# Supplementary material for: Analytical approach of synchronous and asynchronous update schemes applied to solving biological Boolean networks
Source: PLoS One. 2025 Sep 12;20(9):e0319240. doi: 10.1371/journal.pone.0319240 (PMC12431216; doi:10.1371/journal.pone.0319240)
Supplement: S1 Text — This file includes all the codes for the networks used in this work, including their implementation in C#, BoolNet, and SMBL for GINSIM. (DOCX) [file pone.0319240.s001.docx]

# S1 Text. Software appendix

# Analytical approach of synchronous and asynchronous update schemes applied to solving biological Boolean networks

Antonio Bensussen^1^, J. Arturo Arciniega-González^2^, Elena R. Álvarez-Buylla^3^, Juan Carlos Martínez-García^1^*.

^1^Departamento de Control Automático, Cinvestav-IPN, Ciudad de México, Mexico.

^2^Instituto de Investigaciones Biomédicas, Universidad Nacional Autónoma de México, Ciudad de México, Mexico.

^3^Instituto de Ecología, Universidad Nacional Autónoma de México, Ciudad de México, Mexico.

**Content**

[Boolean networks 4](#_Toc196559815)

[Network E.1. 4](#_Toc196559816)

[Network E.2. 4](#_Toc196559817)

[Network E.4. 4](#_Toc196559818)

[Network E.8. 4](#_Toc196559819)

[Network E.11. 4](#_Toc196559820)

[Network E.12. 4](#_Toc196559821)

[Network E.1. 4](#_Toc196559822)

[Network E.13. 5](#_Toc196559823)

[Network E.14. 5](#_Toc196559824)

[Networks of CD4+ T cell differentiation 5](#_Toc196559825)

[Networks of CD8+ T cell differentiation 5](#_Toc196559826)

[Networks of macrophage polarization 6](#_Toc196559827)

[Networks of HSCs differentiation 6](#_Toc196559828)

[Networks of Epithelial-to-Mesenchymal transition in hepatocytes 6](#_Toc196559829)

[Scripts implemented in R 40](#_Toc196559830)

[Calculation of attractors 40](#_Toc196559831)

[Calculation of basins of attraction 42](#_Toc196559832)

[Asynchronous simulation 44](#_Toc196559833)

[Scripts implemented in C# 46](#_Toc196559834)

[Implementation of Theorem 4: Differentiation of CD4+ T cells 46](#_Toc196559835)

[Implementation of Theorem 4: Differentiation of CD8+ T cells 52](#_Toc196559836)

[Implementation of Theorem 4: Differentiation of HSCs 60](#_Toc196559837)

[Implementation of Theorem 4: Differentiation of Macrophages 66](#_Toc196559838)

[Implementation of Theorem 4: Epithelial-to-mesenchymal transition in Hepatocytes 73](#_Toc196559839)

[Scripts implemented in SBML 153](#_Toc196559840)

[Network E.1. 153](#_Toc196559841)

[Network E.2. 155](#_Toc196559842)

[Network E.4. 157](#_Toc196559843)

[Network E.8. 159](#_Toc196559844)

[Network E.11. 161](#_Toc196559845)

[Network E.12. 163](#_Toc196559846)

[Network E.13. 164](#_Toc196559847)

[Network E.14. 167](#_Toc196559848)

[Networks of CD4+ T cell differentiation 168](#_Toc196559849)

[Networks of CD8+ T cell differentiation 193](#_Toc196559850)

[Networks of macrophage polarization 229](#_Toc196559851)

[Networks of HSCs differentiation 258](#_Toc196559852)

# Boolean networks

The networks used in the examples in the main text are presented below, as well as the logical rules of the networks for the differentiation of CD4+ T cells, CD8+ T cells, macrophages, hematopoietic stem cells, and the epithelial-mesenchymal transition in hepatocytes. To use these networks, they must be saved in individual files with the extension ".bnet", in a directory called "networks".

## **Network E.1.**

targets,factors

x1,x2 & x3

x2,x3

x3,x3

## **Network E.2.**

targets, factors

x1, x3

x2, x3

x3, x3

## **Network E.4.**

targets, factors

x1, x1 | x2

x2, !x1 & !x2 | x1 & x2

## **Network E.8.**

targets, factors

x1, x2

x2, x1

## **Network E.11.**

targets, factors

x1, x1 | x2

x2, !x1

## **Network E.12.**

targets, factors

x1, 1

x2, !x2

## **Network E.1.**

targets, factors

Tbet,

## **Network E.13.**

targets, factors

x1, x1

x2, x2 | x3

x3, (x1 | !x3) & (x2 | !x3)

## **Network E.14.**

targets, factors

x1, x1

x2, 1

x3, x1 | !x3

## **Networks of CD4+ T cell differentiation**

targets, factors

Tbet, (IFNy | Tbet) & !(IL4 | GATA3 | IL21 | BCL6 | IL9)

IFNy, (IFNye | ((IFNy | Tbet) & !(GATA3 | TGFB | BCL6)) & !(IL21 | IL4 | IL10 | IL9)

GATA3, ((IL2 & IL4) | GATA3) & !(Tbet | IFNy | TGFB | IL21 | BCL6)

IL2, (IL2e | (IL2 & !FoxP3)) & !(IFNy | IL21 | IL10)

IL4, (IL4e | (GATA3 & (IL2 | IL4)) & !Tbet) & !(IFNy | IL21)

RORγT, (IL21 & TGFB) & !(Tbet | FoxP3 | GATA3 | BCL6)

IL21, (IL21e | IL21 | RORγT | BCL6) & !(IFNy | IL4 | IL10 | IL2 | IL9)

FoxP3, (IL2 & (TGFB | FoxP3)) & !(IL21 | RORγT)

TGFB, (TGFBe | (TGFB | FoxP3) & !IL21)

IL10, IL10e | (IL10 & (IFNy | IL21 | TGFB | GATA3))

BCL6, (IL21) & !(Tbet | IL2 | TGFB)

IL9, (IL4 & ((IL10 & IL2) | TGFB)) & !(IFNy | IL21)

IFNye, IFNye

IL21e, IL21e

TGFBe, TGFBe

IL2e, IL2e

IL4e, IL4e

IL10e, IL10e

## **Networks of CD8+ T cell differentiation**

targets, factors

TBET, ((IFNy | IL12s & !(IL6s | IL4 | IL10)) & !TBET) & !(IL4 | GATA3 | IL6s | FOXO1)

IFNy, ((IFNy | IFN1 & ((IFNy | TBET | EOMES) & mTORC1 & !(GATA3 | TGFB))) & !IL6s & IL4 | IL10)

IL4, ((IL2s & IL4) | EOMES | GATA3) & !(TBET | TGFB | IL6s | IFNy)

RORyT, (IL6s & TGFB) & !(TBET | FOXP3 | GATA3 | FOXO1)

IL10, ((IL10s | EOMES) & (IL10 & (IFNy | IL6s | TGFB | GATA3))) & mTORC1

FOXP3, ((IL2s & IL12s) & TGFB | FOXP3 | IL4 & FOXO1 & !(IL6s | RORyT))

FOXO1, (ROS | FOXO1) & !(mTORC1 | mTORC2)

EOMES, (ROS | EOMES | IFN1 | FOXO1) & !(mTORC2)

mTORC1, (aa | ROS | IL12 | IL12s | Akt) & !(mTORC2 | PD1 | IL15s)

mTORC2, (GFs | ROS) & !mTORC1

ROS, (Glucose | FFAs | Ceramide | EtOH) & SOD

Akt, IFNy | IL4 | IL10 | mTORC2

GLUT1, Akt & EOMES & !FOXO1

GranzymeB, TBET & !(GATA3 | RORyT | FOXP3 | FOXO1)

SOD, ROS & (FOXO1) & !Ceramide

BCL2, (FOXO1) & !ROS

Casp3, FasL & (ROS | Casp3) & !BCL2

## **Networks of macrophage polarization**

targets, factors

IFNy, IFNyInput & !TGFB

IFNB, (IFNBInput | IFNB) & !(TGFB | IFNy)

Tbet, ((IL12 | IL12Input) & (IFNB | IFNy | IL6)) & !(TGFB | GATA3)

iNOS, (IFNy | IFNB) & (IL1B | TNF | IL6 | TLR4) | GMCSF

TNF, (TNFInput | TNF | TLR4 | IL1B | IL6 | Insulin | CeramideInput) & !IL10 & !IL4

TLR4, (CeramideInput | LPS) & !IL4

IL1B, (IL1BInput | IL1B | TNF | TLR4 | IL6) & !IL10 & !IL4

IL6, IL6Input | IL6 | IL1B | TNF | TLR4 | TGFB

IL10, (IL10Input | IL10 | TLR4 | IL6 | TGFB) & !(IFNB | IFNy)

IL4, (IL4Input | GATA3) & !Tbet

IRF4, (IL4 | IL6 | IRF4 | GMCSF) & !(TLR4 | TNF | IL1B)

Arg1, IRF4 & IL4

GATA3, (IL4 | GATA3) & !(Tbet | TGFB)

TGFB, (TGFBInput | TGFB | IL4 | IL6 | IL10) & !(IFNB | IFNy | TLR4 | TNF)

## **Networks of HSCs differentiation**

targets, factors

Runx1, (PU1 | GATA2 | Runx1) & !Ikzf1

Meis1, (Meis1 | Runx1 | PU1) & !Gfi1

HIF1, (!Oxygen & O2 & !(p53 | FOXO3 | AMPK)) & (!Oxygen & Meis1)

FOXO3, ((AMPK & (HIF1 | p53)) & !(AKT | CEBPA))

p53, HIF1 & !(AKT | GATA1 | PU1)

GATA2, (GATA2 | p53) & !(GATA1 | PU1 | Gfi1)

GATA1, (GATA1 | GATA2 | AKT | Runx1) & !(PU1 | p53 | Ikzf1)

PU1, (PU1 | Runx1 | (CEBPA & Ikzf1)) & !(GATA1 | GATA2 | Gfi1)

CEBPA, (PU1 & Runx1) & !Mef2c

Ikzf1, (Mef2c | Ikzf1 | Runx1) & !(CEBPA | PU1 | GATA1)

Gfi1, (Ikzf1 | CEBPA) & !(PU1 | p53)

Mef2c, PU1 & !CEBPA

mTOR, AKT & (!AMPK | !p53)

AMPK, (!AKT | !OXPHOS & !HIF1) & p53

AKT, H2O2 | !(p53 | FOXO3)

H2O2, ((GATA1 | HIF1 | PU1 | OXPHOS | (SOD & O2)) & !Antiox

O2, !SOD & OXPHOS

SOD, p53 | FOXO3

Antiox, p53 | FOXO3

OXPHOS, (mTOR | AKT) & !(FOXO3 | HIF1)

## **Networks of Epithelial-to-Mesenchymal transition in hepatocytes**

targets, factors

EZH2, !EZH2&!BMI1&!E2F&!Bcatenin&YAP1&NFkB&!p53 | !EZH2&!BMI1&!E2F&Bcatenin&!p53 | !EZH2&!BMI1&E2F&!p53 | !EZH2&BMI1&!E2F&!Bcatenin&!YAP1&!NFkB&TGFB&!p53&!p16 | !EZH2&BMI1&!E2F&!Bcatenin&!YAP1&NFkB&!p53&!p16 | !EZH2&BMI1&!E2F&!Bcatenin&YAP1&!NFkB&TGFB&!p53&!p16 | !EZH2&BMI1&!E2F&!Bcatenin&YAP1&NFkB&!p53 | !EZH2&BMI1&!E2F&Bcatenin&!p53 | !EZH2&BMI1&E2F&!p53 | EZH2&!E2F&!Bcatenin&!YAP1&!NFkB&TGFB&!p53&!p16 | EZH2&!E2F&!Bcatenin&!YAP1&NFkB&!p53&!p16 | EZH2&!E2F&!Bcatenin&YAP1&!NFkB&TGFB&!p53&!p16 | EZH2&!E2F&!Bcatenin&YAP1&NFkB&!p53 | EZH2&!E2F&Bcatenin&!p53 | EZH2&E2F&!p53

BMI1, !BMI1&!E2F&!Bcatenin&YAP1&!HNF4A&NFkB&!SNAI1&!ZEB1&!OCT4&!p53 | !BMI1&!E2F&!Bcatenin&YAP1&!HNF4A&NFkB&!SNAI1&ZEB1&!p53 | !BMI1&!E2F&!Bcatenin&YAP1&!HNF4A&NFkB&SNAI1&!p53 | !BMI1&!E2F&!Bcatenin&YAP1&HNF4A&NFkB&!SNAI1&ZEB1&!p53 | !BMI1&!E2F&!Bcatenin&YAP1&HNF4A&NFkB&SNAI1&!p53 | !BMI1&!E2F&Bcatenin&!YAP1&!HNF4A&!NFkB&!SNAI1&!ZEB1&!SOX9&!OCT4&NANOG&SOX2&!p53 | !BMI1&!E2F&Bcatenin&!YAP1&!HNF4A&!NFkB&!SNAI1&!ZEB1&SOX9&!OCT4&SOX2&!p53 | !BMI1&!E2F&Bcatenin&!YAP1&!HNF4A&!NFkB&!SNAI1&ZEB1&!SOX9&NANOG&SOX2&!p53 | !BMI1&!E2F&Bcatenin&!YAP1&!HNF4A&!NFkB&!SNAI1&ZEB1&SOX9&SOX2&!p53 | !BMI1&!E2F&Bcatenin&!YAP1&!HNF4A&!NFkB&SNAI1&SOX2&!p53 | !BMI1&!E2F&Bcatenin&!YAP1&!HNF4A&NFkB&!SNAI1&!ZEB1&!OCT4&SOX2&!p53 | !BMI1&!E2F&Bcatenin&!YAP1&!HNF4A&NFkB&!SNAI1&ZEB1&SOX2&!p53 | !BMI1&!E2F&Bcatenin&!YAP1&!HNF4A&NFkB&SNAI1&SOX2&!p53 | !BMI1&!E2F&Bcatenin&!YAP1&HNF4A&!NFkB&!SNAI1&ZEB1&!SOX9&NANOG&SOX2&!p53 | !BMI1&!E2F&Bcatenin&!YAP1&HNF4A&!NFkB&!SNAI1&ZEB1&SOX9&SOX2&!p53 | !BMI1&!E2F&Bcatenin&!YAP1&HNF4A&!NFkB&SNAI1&SOX2&!p53 | !BMI1&!E2F&Bcatenin&!YAP1&HNF4A&NFkB&!SNAI1&ZEB1&SOX2&!p53 | !BMI1&!E2F&Bcatenin&!YAP1&HNF4A&NFkB&SNAI1&SOX2&!p53 | !BMI1&!E2F&Bcatenin&YAP1&!HNF4A&!NFkB&!SNAI1&!ZEB1&!SOX9&!OCT4&NANOG&!p53 | !BMI1&!E2F&Bcatenin&YAP1&!HNF4A&!NFkB&!SNAI1&!ZEB1&SOX9&!OCT4&!p53 | !BMI1&!E2F&Bcatenin&YAP1&!HNF4A&!NFkB&!SNAI1&ZEB1&!SOX9&NANOG&!p53 | !BMI1&!E2F&Bcatenin&YAP1&!HNF4A&!NFkB&!SNAI1&ZEB1&SOX9&!p53 | !BMI1&!E2F&Bcatenin&YAP1&!HNF4A&!NFkB&SNAI1&!p53 | !BMI1&!E2F&Bcatenin&YAP1&!HNF4A&NFkB&!SNAI1&!ZEB1&!OCT4&!p53 | !BMI1&!E2F&Bcatenin&YAP1&!HNF4A&NFkB&!SNAI1&ZEB1&!p53 | !BMI1&!E2F&Bcatenin&YAP1&!HNF4A&NFkB&SNAI1&!p53 | !BMI1&!E2F&Bcatenin&YAP1&HNF4A&!NFkB&!SNAI1&ZEB1&!SOX9&NANOG&!p53 | !BMI1&!E2F&Bcatenin&YAP1&HNF4A&!NFkB&!SNAI1&ZEB1&SOX9&!p53 | !BMI1&!E2F&Bcatenin&YAP1&HNF4A&!NFkB&SNAI1&!p53 | !BMI1&!E2F&Bcatenin&YAP1&HNF4A&NFkB&!SNAI1&ZEB1&!p53 | !BMI1&!E2F&Bcatenin&YAP1&HNF4A&NFkB&SNAI1&!p53 | !BMI1&E2F&!Bcatenin&!YAP1&!HNF4A&!NFkB&!SNAI1&!ZEB1&!OCT4 | !BMI1&E2F&!Bcatenin&!YAP1&!HNF4A&!NFkB&!SNAI1&!ZEB1&OCT4&SOX2&!p53 | !BMI1&E2F&!Bcatenin&!YAP1&!HNF4A&!NFkB&!SNAI1&ZEB1&!OCT4 | !BMI1&E2F&!Bcatenin&!YAP1&!HNF4A&!NFkB&!SNAI1&ZEB1&OCT4&!p53 | !BMI1&E2F&!Bcatenin&!YAP1&!HNF4A&!NFkB&SNAI1&!ZEB1&!OCT4 | !BMI1&E2F&!Bcatenin&!YAP1&!HNF4A&!NFkB&SNAI1&!ZEB1&OCT4&!p53 | !BMI1&E2F&!Bcatenin&!YAP1&!HNF4A&!NFkB&SNAI1&ZEB1 | !BMI1&E2F&!Bcatenin&!YAP1&!HNF4A&NFkB&!SNAI1&!OCT4 | !BMI1&E2F&!Bcatenin&!YAP1&!HNF4A&NFkB&!SNAI1&OCT4&!p53 | !BMI1&E2F&!Bcatenin&!YAP1&!HNF4A&NFkB&SNAI1&!ZEB1&!OCT4 | !BMI1&E2F&!Bcatenin&!YAP1&!HNF4A&NFkB&SNAI1&!ZEB1&OCT4&!p53 | !BMI1&E2F&!Bcatenin&!YAP1&!HNF4A&NFkB&SNAI1&ZEB1 | !BMI1&E2F&!Bcatenin&!YAP1&HNF4A&!NFkB&!SNAI1&!ZEB1&!TGFB&SOX2&!p53 | !BMI1&E2F&!Bcatenin&!YAP1&HNF4A&!NFkB&!SNAI1&ZEB1&!p53 | !BMI1&E2F&!Bcatenin&!YAP1&HNF4A&!NFkB&SNAI1&!ZEB1&!p53 | !BMI1&E2F&!Bcatenin&!YAP1&HNF4A&!NFkB&SNAI1&ZEB1 | !BMI1&E2F&!Bcatenin&!YAP1&HNF4A&NFkB&!SNAI1&!p53 | !BMI1&E2F&!Bcatenin&!YAP1&HNF4A&NFkB&SNAI1&!ZEB1&!p53 | !BMI1&E2F&!Bcatenin&!YAP1&HNF4A&NFkB&SNAI1&ZEB1 | !BMI1&E2F&!Bcatenin&YAP1&!HNF4A&!SNAI1&!OCT4 | !BMI1&E2F&!Bcatenin&YAP1&!HNF4A&!SNAI1&OCT4&!p53 | !BMI1&E2F&!Bcatenin&YAP1&!HNF4A&SNAI1&!ZEB1&!OCT4 | !BMI1&E2F&!Bcatenin&YAP1&!HNF4A&SNAI1&!ZEB1&OCT4&!p53 | !BMI1&E2F&!Bcatenin&YAP1&!HNF4A&SNAI1&ZEB1 | !BMI1&E2F&!Bcatenin&YAP1&HNF4A&!NFkB&!SNAI1&!ZEB1&!TGFB&!p53 | !BMI1&E2F&!Bcatenin&YAP1&HNF4A&!NFkB&!SNAI1&ZEB1&!p53 | !BMI1&E2F&!Bcatenin&YAP1&HNF4A&!NFkB&SNAI1&!ZEB1&!p53 | !BMI1&E2F&!Bcatenin&YAP1&HNF4A&!NFkB&SNAI1&ZEB1 | !BMI1&E2F&!Bcatenin&YAP1&HNF4A&NFkB&!SNAI1&!p53 | !BMI1&E2F&!Bcatenin&YAP1&HNF4A&NFkB&SNAI1&!ZEB1&!p53 | !BMI1&E2F&!Bcatenin&YAP1&HNF4A&NFkB&SNAI1&ZEB1 | !BMI1&E2F&Bcatenin&!HNF4A&!SNAI1&!OCT4 | !BMI1&E2F&Bcatenin&!HNF4A&!SNAI1&OCT4&!p53 | !BMI1&E2F&Bcatenin&!HNF4A&SNAI1&!ZEB1&!OCT4 | !BMI1&E2F&Bcatenin&!HNF4A&SNAI1&!ZEB1&OCT4&!p53 | !BMI1&E2F&Bcatenin&!HNF4A&SNAI1&ZEB1 | !BMI1&E2F&Bcatenin&HNF4A&!NFkB&!SNAI1&!ZEB1&!TGFB&!p53 | !BMI1&E2F&Bcatenin&HNF4A&!NFkB&!SNAI1&ZEB1&!p53 | !BMI1&E2F&Bcatenin&HNF4A&!NFkB&SNAI1&!ZEB1&!p53 | !BMI1&E2F&Bcatenin&HNF4A&!NFkB&SNAI1&ZEB1 | !BMI1&E2F&Bcatenin&HNF4A&NFkB&!SNAI1&!p53 | !BMI1&E2F&Bcatenin&HNF4A&NFkB&SNAI1&!ZEB1&!p53 | !BMI1&E2F&Bcatenin&HNF4A&NFkB&SNAI1&ZEB1 | BMI1&!E2F&!Bcatenin&YAP1&!HNF4A&NFkB&!p53 | BMI1&!E2F&!Bcatenin&YAP1&HNF4A&NFkB&!SNAI1&!ZEB1&!SOX9&NANOG&!p53 | BMI1&!E2F&!Bcatenin&YAP1&HNF4A&NFkB&!SNAI1&!ZEB1&SOX9&!p53 | BMI1&!E2F&!Bcatenin&YAP1&HNF4A&NFkB&!SNAI1&ZEB1&!p53 | BMI1&!E2F&!Bcatenin&YAP1&HNF4A&NFkB&SNAI1&!p53 | BMI1&!E2F&Bcatenin&!YAP1&!HNF4A&!NFkB&!SNAI1&!SOX9&NANOG&SOX2&!p53 | BMI1&!E2F&Bcatenin&!YAP1&!HNF4A&!NFkB&!SNAI1&SOX9&SOX2&!p53 | BMI1&!E2F&Bcatenin&!YAP1&!HNF4A&!NFkB&SNAI1&SOX2&!p53 | BMI1&!E2F&Bcatenin&!YAP1&!HNF4A&NFkB&SOX2&!p53 | BMI1&!E2F&Bcatenin&!YAP1&HNF4A&!NFkB&!SNAI1&!ZEB1&!TGFB&!SOX9&NANOG&SOX2&!p53 | BMI1&!E2F&Bcatenin&!YAP1&HNF4A&!NFkB&!SNAI1&!ZEB1&!TGFB&SOX9&SOX2&!p53 | BMI1&!E2F&Bcatenin&!YAP1&HNF4A&!NFkB&!SNAI1&ZEB1&!SOX9&NANOG&SOX2&!p53 | BMI1&!E2F&Bcatenin&!YAP1&HNF4A&!NFkB&!SNAI1&ZEB1&SOX9&SOX2&!p53 | BMI1&!E2F&Bcatenin&!YAP1&HNF4A&!NFkB&SNAI1&SOX2&!p53 | BMI1&!E2F&Bcatenin&!YAP1&HNF4A&NFkB&!SNAI1&!ZEB1&!TGFB&!SOX9&NANOG&SOX2&!p53 | BMI1&!E2F&Bcatenin&!YAP1&HNF4A&NFkB&!SNAI1&!ZEB1&!TGFB&SOX9&SOX2&!p53 | BMI1&!E2F&Bcatenin&!YAP1&HNF4A&NFkB&!SNAI1&ZEB1&SOX2&!p53 | BMI1&!E2F&Bcatenin&!YAP1&HNF4A&NFkB&SNAI1&SOX2&!p53 | BMI1&!E2F&Bcatenin&YAP1&!HNF4A&!NFkB&!SNAI1&!SOX9&NANOG&!p53 | BMI1&!E2F&Bcatenin&YAP1&!HNF4A&!NFkB&!SNAI1&SOX9&!p53 | BMI1&!E2F&Bcatenin&YAP1&!HNF4A&!NFkB&SNAI1&!p53 | BMI1&!E2F&Bcatenin&YAP1&!HNF4A&NFkB&!p53 | BMI1&!E2F&Bcatenin&YAP1&HNF4A&!NFkB&!SNAI1&!ZEB1&!TGFB&!SOX9&NANOG&!p53 | BMI1&!E2F&Bcatenin&YAP1&HNF4A&!NFkB&!SNAI1&!ZEB1&!TGFB&SOX9&!p53 | BMI1&!E2F&Bcatenin&YAP1&HNF4A&!NFkB&!SNAI1&ZEB1&!SOX9&NANOG&!p53 | BMI1&!E2F&Bcatenin&YAP1&HNF4A&!NFkB&!SNAI1&ZEB1&SOX9&!p53 | BMI1&!E2F&Bcatenin&YAP1&HNF4A&!NFkB&SNAI1&!p53 | BMI1&!E2F&Bcatenin&YAP1&HNF4A&NFkB&!SNAI1&!ZEB1&!SOX9&NANOG&!p53 | BMI1&!E2F&Bcatenin&YAP1&HNF4A&NFkB&!SNAI1&!ZEB1&SOX9&!p53 | BMI1&!E2F&Bcatenin&YAP1&HNF4A&NFkB&!SNAI1&ZEB1&!p53 | BMI1&!E2F&Bcatenin&YAP1&HNF4A&NFkB&SNAI1&!p53 | BMI1&E2F

E2F, !E2F&!Bcatenin&YAP1&!p21 | !E2F&Bcatenin&!YAP1&!HNF4A&SOX2&!p53&!p21&!RB | !E2F&Bcatenin&!YAP1&HNF4A&!NFkB&!SNAI1&!ZEB1&!TGFB&SOX2&!p53&!p21&!RB | !E2F&Bcatenin&!YAP1&HNF4A&!NFkB&!SNAI1&ZEB1&SOX2&!p53&!p21&!RB | !E2F&Bcatenin&!YAP1&HNF4A&NFkB&!SNAI1&!ZEB1&!TGFB&SOX2&!p53&!p21&!RB | !E2F&Bcatenin&YAP1&!p21 | E2F&!Bcatenin&!YAP1&!HNF4A&!NFkB&!SOX2&!p21&!RB | E2F&!Bcatenin&!YAP1&!HNF4A&!NFkB&SOX2&!p53&!RB | E2F&!Bcatenin&!YAP1&!HNF4A&!NFkB&SOX2&p53&!p21&!RB | E2F&!Bcatenin&!YAP1&!HNF4A&NFkB&!p53&!RB | E2F&!Bcatenin&!YAP1&!HNF4A&NFkB&p53&!p21&!RB | E2F&!Bcatenin&!YAP1&HNF4A&!NFkB&!SNAI1&!ZEB1&!TGFB&!SOX2&!p21&!RB | E2F&!Bcatenin&!YAP1&HNF4A&!NFkB&!SNAI1&!ZEB1&!TGFB&SOX2&!p53&!RB | E2F&!Bcatenin&!YAP1&HNF4A&!NFkB&!SNAI1&!ZEB1&!TGFB&SOX2&p53&!p21&!RB | E2F&!Bcatenin&!YAP1&HNF4A&!NFkB&!SNAI1&!ZEB1&TGFB&!p21&!RB | E2F&!Bcatenin&!YAP1&HNF4A&!NFkB&!SNAI1&ZEB1&!SOX2&!p21&!RB | E2F&!Bcatenin&!YAP1&HNF4A&!NFkB&!SNAI1&ZEB1&SOX2&!p53&!RB | E2F&!Bcatenin&!YAP1&HNF4A&!NFkB&!SNAI1&ZEB1&SOX2&p53&!p21&!RB | E2F&!Bcatenin&!YAP1&HNF4A&!NFkB&SNAI1&SOX2&!p53&!RB | E2F&!Bcatenin&!YAP1&HNF4A&NFkB&!SNAI1&!ZEB1&!p53&!RB | E2F&!Bcatenin&!YAP1&HNF4A&NFkB&!SNAI1&!ZEB1&p53&!p21&!RB | E2F&!Bcatenin&!YAP1&HNF4A&NFkB&!SNAI1&ZEB1&!p53&!RB | E2F&!Bcatenin&!YAP1&HNF4A&NFkB&SNAI1&!p53&!RB | E2F&!Bcatenin&YAP1&!HNF4A&!p53&!p21 | E2F&!Bcatenin&YAP1&!HNF4A&!p53&p21&!RB | E2F&!Bcatenin&YAP1&!HNF4A&p53&!p21 | E2F&!Bcatenin&YAP1&HNF4A&!NFkB&!SNAI1&!ZEB1&!TGFB&!p53&!p21 | E2F&!Bcatenin&YAP1&HNF4A&!NFkB&!SNAI1&!ZEB1&!TGFB&!p53&p21&!RB | E2F&!Bcatenin&YAP1&HNF4A&!NFkB&!SNAI1&!ZEB1&!TGFB&p53&!p21 | E2F&!Bcatenin&YAP1&HNF4A&!NFkB&!SNAI1&!ZEB1&TGFB&!p21 | E2F&!Bcatenin&YAP1&HNF4A&!NFkB&!SNAI1&ZEB1&!p53&!p21 | E2F&!Bcatenin&YAP1&HNF4A&!NFkB&!SNAI1&ZEB1&!p53&p21&!RB | E2F&!Bcatenin&YAP1&HNF4A&!NFkB&!SNAI1&ZEB1&p53&!p21 | E2F&!Bcatenin&YAP1&HNF4A&!NFkB&SNAI1&!p53&!p21 | E2F&!Bcatenin&YAP1&HNF4A&!NFkB&SNAI1&!p53&p21&!RB | E2F&!Bcatenin&YAP1&HNF4A&!NFkB&SNAI1&p53&!p21 | E2F&!Bcatenin&YAP1&HNF4A&NFkB&!p53&!p21 | E2F&!Bcatenin&YAP1&HNF4A&NFkB&!p53&p21&!RB | E2F&!Bcatenin&YAP1&HNF4A&NFkB&p53&!p21 | E2F&Bcatenin&!YAP1&!HNF4A&!p53&!RB | E2F&Bcatenin&!YAP1&!HNF4A&p53&!p21&!RB | E2F&Bcatenin&!YAP1&HNF4A&!NFkB&!SNAI1&!ZEB1&!TGFB&!p53&!RB | E2F&Bcatenin&!YAP1&HNF4A&!NFkB&!SNAI1&!ZEB1&!TGFB&p53&!p21&!RB | E2F&Bcatenin&!YAP1&HNF4A&!NFkB&!SNAI1&!ZEB1&TGFB&!p21&!RB | E2F&Bcatenin&!YAP1&HNF4A&!NFkB&!SNAI1&ZEB1&!p53&!RB | E2F&Bcatenin&!YAP1&HNF4A&!NFkB&!SNAI1&ZEB1&p53&!p21&!RB | E2F&Bcatenin&!YAP1&HNF4A&!NFkB&SNAI1&!p53&!RB | E2F&Bcatenin&!YAP1&HNF4A&NFkB&!SNAI1&!ZEB1&!p53&!RB | E2F&Bcatenin&!YAP1&HNF4A&NFkB&!SNAI1&!ZEB1&p53&!p21&!RB | E2F&Bcatenin&!YAP1&HNF4A&NFkB&!SNAI1&ZEB1&!p53&!RB | E2F&Bcatenin&!YAP1&HNF4A&NFkB&SNAI1&!p53&!RB | E2F&Bcatenin&YAP1&!HNF4A&!p53&!p21 | E2F&Bcatenin&YAP1&!HNF4A&!p53&p21&!RB | E2F&Bcatenin&YAP1&!HNF4A&p53&!p21 | E2F&Bcatenin&YAP1&HNF4A&!NFkB&!SNAI1&!ZEB1&!TGFB&!p53&!p21 | E2F&Bcatenin&YAP1&HNF4A&!NFkB&!SNAI1&!ZEB1&!TGFB&!p53&p21&!RB | E2F&Bcatenin&YAP1&HNF4A&!NFkB&!SNAI1&!ZEB1&!TGFB&p53&!p21 | E2F&Bcatenin&YAP1&HNF4A&!NFkB&!SNAI1&!ZEB1&TGFB&!p21 | E2F&Bcatenin&YAP1&HNF4A&!NFkB&!SNAI1&ZEB1&!p53&!p21 | E2F&Bcatenin&YAP1&HNF4A&!NFkB&!SNAI1&ZEB1&!p53&p21&!RB | E2F&Bcatenin&YAP1&HNF4A&!NFkB&!SNAI1&ZEB1&p53&!p21 | E2F&Bcatenin&YAP1&HNF4A&!NFkB&SNAI1&!p53&!p21 | E2F&Bcatenin&YAP1&HNF4A&!NFkB&SNAI1&!p53&p21&!RB | E2F&Bcatenin&YAP1&HNF4A&!NFkB&SNAI1&p53&!p21 | E2F&Bcatenin&YAP1&HNF4A&NFkB&!p53&!p21 | E2F&Bcatenin&YAP1&HNF4A&NFkB&!p53&p21&!RB | E2F&Bcatenin&YAP1&HNF4A&NFkB&p53&!p21

CyclinD, !E2F&!Bcatenin&YAP1&NFkB&!p53&!p16 | !E2F&Bcatenin&!YAP1&!HNF4A&SOX2&!p53&!p16 | !E2F&Bcatenin&!YAP1&HNF4A&!SNAI1&!ZEB1&!TGFB&SOX2&!p53&!p16 | !E2F&Bcatenin&!YAP1&HNF4A&!SNAI1&ZEB1&SOX2&!p53&!p16 | !E2F&Bcatenin&!YAP1&HNF4A&SNAI1&SOX2&!p53&!p16 | !E2F&Bcatenin&YAP1&!p16 | E2F&!Bcatenin&!YAP1&!HNF4A&!HNF6&!FOXA2&!NFkB&SOX2&!p53&!p16 | E2F&!Bcatenin&!YAP1&!HNF4A&!HNF6&!FOXA2&NFkB&!p16 | E2F&!Bcatenin&!YAP1&!HNF4A&!HNF6&FOXA2&!p16 | E2F&!Bcatenin&!YAP1&!HNF4A&HNF6&!p16 | E2F&!Bcatenin&!YAP1&HNF4A&!HNF6&!FOXA2&!NFkB&!SNAI1&!ZEB1&!TGFB&SOX2&!p53&!p16 | E2F&!Bcatenin&!YAP1&HNF4A&!HNF6&!FOXA2&!NFkB&!SNAI1&ZEB1&SOX2&!p53&!p16 | E2F&!Bcatenin&!YAP1&HNF4A&!HNF6&!FOXA2&!NFkB&SNAI1&SOX2&!p53&!p16 | E2F&!Bcatenin&!YAP1&HNF4A&!HNF6&!FOXA2&NFkB&!p16 | E2F&!Bcatenin&!YAP1&HNF4A&!HNF6&FOXA2&!p16 | E2F&!Bcatenin&!YAP1&HNF4A&HNF6&!p16 | E2F&!Bcatenin&YAP1&!p16 | E2F&Bcatenin&!p16

Bcatenin, !EZH2&!HNF4A&TGFB&!SOX2&!p53 | !EZH2&HNF4A&!ZEB1&SNAI2&TGFB&!SOX2&!p53 | !EZH2&HNF4A&ZEB1&TGFB&!SOX2&!p53 | EZH2&!BMI1&!HNF4A&TGFB&!SOX2&!p53 | EZH2&!BMI1&HNF4A&!ZEB1&SNAI2&TGFB&!SOX2&!p53 | EZH2&!BMI1&HNF4A&ZEB1&TGFB&!SOX2&!p53 | EZH2&BMI1&!ZEB1&!SNAI2&!SOX2&!p53 | EZH2&BMI1&!ZEB1&SNAI2&!p53 | EZH2&BMI1&ZEB1&!p53

RB, !CyclinD | CyclinD&!p16&p21 | CyclinD&p16

p21, !E2F&!Bcatenin&!YAP1&!HNF4A&!FOXA2&!NFkB&!SNAI1&!TGFB&!SOX9&!NANOG&p53 | !E2F&!Bcatenin&!YAP1&!HNF4A&!FOXA2&!NFkB&!SNAI1&!TGFB&!SOX9&NANOG | !E2F&!Bcatenin&!YAP1&!HNF4A&!FOXA2&!NFkB&!SNAI1&!TGFB&SOX9 | !E2F&!Bcatenin&!YAP1&!HNF4A&!FOXA2&!NFkB&!SNAI1&TGFB | !E2F&!Bcatenin&!YAP1&!HNF4A&!FOXA2&!NFkB&SNAI1&!TGFB&!SOX9&p53 | !E2F&!Bcatenin&!YAP1&!HNF4A&!FOXA2&!NFkB&SNAI1&!TGFB&SOX9 | !E2F&!Bcatenin&!YAP1&!HNF4A&!FOXA2&!NFkB&SNAI1&TGFB | !E2F&!Bcatenin&!YAP1&!HNF4A&!FOXA2&NFkB | !E2F&!Bcatenin&!YAP1&!HNF4A&FOXA2 | !E2F&!Bcatenin&!YAP1&HNF4A | !E2F&!Bcatenin&YAP1&!HNF4A&!FOXA2&!NFkB&!SNAI1&!TGFB&!SOX9&!NANOG&p53 | !E2F&!Bcatenin&YAP1&!HNF4A&!FOXA2&!NFkB&!SNAI1&!TGFB&!SOX9&NANOG | !E2F&!Bcatenin&YAP1&!HNF4A&!FOXA2&!NFkB&!SNAI1&!TGFB&SOX9 | !E2F&!Bcatenin&YAP1&!HNF4A&!FOXA2&!NFkB&!SNAI1&TGFB | !E2F&!Bcatenin&YAP1&!HNF4A&!FOXA2&!NFkB&SNAI1&!TGFB&!SOX9&p53 | !E2F&!Bcatenin&YAP1&!HNF4A&!FOXA2&!NFkB&SNAI1&!TGFB&SOX9 | !E2F&!Bcatenin&YAP1&!HNF4A&!FOXA2&!NFkB&SNAI1&TGFB | !E2F&!Bcatenin&YAP1&!HNF4A&!FOXA2&NFkB&p53 | !E2F&!Bcatenin&YAP1&!HNF4A&FOXA2&!NFkB | !E2F&!Bcatenin&YAP1&!HNF4A&FOXA2&NFkB&p53 | !E2F&!Bcatenin&YAP1&HNF4A&!NFkB | !E2F&!Bcatenin&YAP1&HNF4A&NFkB&p53 | !E2F&Bcatenin&p53 | E2F&!Bcatenin&!YAP1&!HNF4A&!FOXA2&!NFkB&!SNAI1&!TGFB&!SOX9&!NANOG&p53 | E2F&!Bcatenin&!YAP1&!HNF4A&!FOXA2&!NFkB&!SNAI1&!TGFB&!SOX9&NANOG&!SOX2 | E2F&!Bcatenin&!YAP1&!HNF4A&!FOXA2&!NFkB&!SNAI1&!TGFB&!SOX9&NANOG&SOX2&p53 | E2F&!Bcatenin&!YAP1&!HNF4A&!FOXA2&!NFkB&!SNAI1&!TGFB&SOX9&!SOX2 | E2F&!Bcatenin&!YAP1&!HNF4A&!FOXA2&!NFkB&!SNAI1&!TGFB&SOX9&SOX2&p53 | E2F&!Bcatenin&!YAP1&!HNF4A&!FOXA2&!NFkB&!SNAI1&TGFB&!SOX2 | E2F&!Bcatenin&!YAP1&!HNF4A&!FOXA2&!NFkB&!SNAI1&TGFB&SOX2&p53 | E2F&!Bcatenin&!YAP1&!HNF4A&!FOXA2&!NFkB&SNAI1&!TGFB&!SOX9&p53 | E2F&!Bcatenin&!YAP1&!HNF4A&!FOXA2&!NFkB&SNAI1&!TGFB&SOX9&!SOX2 | E2F&!Bcatenin&!YAP1&!HNF4A&!FOXA2&!NFkB&SNAI1&!TGFB&SOX9&SOX2&p53 | E2F&!Bcatenin&!YAP1&!HNF4A&!FOXA2&!NFkB&SNAI1&TGFB&!SOX2 | E2F&!Bcatenin&!YAP1&!HNF4A&!FOXA2&!NFkB&SNAI1&TGFB&SOX2&p53 | E2F&!Bcatenin&!YAP1&!HNF4A&!FOXA2&NFkB&p53 | E2F&!Bcatenin&!YAP1&!HNF4A&FOXA2&!NFkB&!SOX2 | E2F&!Bcatenin&!YAP1&!HNF4A&FOXA2&!NFkB&SOX2&p53 | E2F&!Bcatenin&!YAP1&!HNF4A&FOXA2&NFkB&p53 | E2F&!Bcatenin&!YAP1&HNF4A&!NFkB&!SNAI1&!ZEB1&!TGFB&!SOX2 | E2F&!Bcatenin&!YAP1&HNF4A&!NFkB&!SNAI1&!ZEB1&!TGFB&SOX2&p53 | E2F&!Bcatenin&!YAP1&HNF4A&!NFkB&!SNAI1&!ZEB1&TGFB | E2F&!Bcatenin&!YAP1&HNF4A&!NFkB&!SNAI1&ZEB1&!SOX2 | E2F&!Bcatenin&!YAP1&HNF4A&!NFkB&!SNAI1&ZEB1&SOX2&p53 | E2F&!Bcatenin&!YAP1&HNF4A&!NFkB&SNAI1&!SOX2 | E2F&!Bcatenin&!YAP1&HNF4A&!NFkB&SNAI1&SOX2&p53 | E2F&!Bcatenin&!YAP1&HNF4A&NFkB&p53 | E2F&!Bcatenin&YAP1&!HNF4A&p53 | E2F&!Bcatenin&YAP1&HNF4A&!NFkB&!SNAI1&!ZEB1&!TGFB&p53 | E2F&!Bcatenin&YAP1&HNF4A&!NFkB&!SNAI1&!ZEB1&TGFB | E2F&!Bcatenin&YAP1&HNF4A&!NFkB&!SNAI1&ZEB1&p53 | E2F&!Bcatenin&YAP1&HNF4A&!NFkB&SNAI1&p53 | E2F&!Bcatenin&YAP1&HNF4A&NFkB&p53 | E2F&Bcatenin&p53

HNF4A, !E2F&!Bcatenin&!YAP1&!HNF1A&!HNF4A&!HNF6&!FOXA2&!SNAI1&!ZEB1&!SNAI2&!SOX9&!NANOG&SOX2&!p53 | !E2F&!Bcatenin&!YAP1&!HNF1A&!HNF4A&!HNF6&!FOXA2&!SNAI1&!ZEB1&!SNAI2&SOX9&!NANOG&SOX2 | !E2F&!Bcatenin&!YAP1&!HNF1A&!HNF4A&!HNF6&!FOXA2&!SNAI1&ZEB1&!SNAI2&!NANOG&SOX2&!p53 | !E2F&!Bcatenin&!YAP1&!HNF1A&!HNF4A&!HNF6&FOXA2&!SNAI1&!SNAI2 | !E2F&!Bcatenin&!YAP1&!HNF1A&!HNF4A&HNF6&!SNAI1&!SNAI2 | !E2F&!Bcatenin&!YAP1&!HNF1A&HNF4A&!HNF6&!FOXA2&!SNAI1&!SNAI2 | !E2F&!Bcatenin&!YAP1&!HNF1A&HNF4A&!HNF6&FOXA2&!SNAI1 | !E2F&!Bcatenin&!YAP1&!HNF1A&HNF4A&!HNF6&FOXA2&SNAI1&!SNAI2 | !E2F&!Bcatenin&!YAP1&!HNF1A&HNF4A&HNF6&!SNAI1 | !E2F&!Bcatenin&!YAP1&!HNF1A&HNF4A&HNF6&SNAI1&!SNAI2 | !E2F&!Bcatenin&!YAP1&HNF1A&!HNF4A&!HNF6&!FOXA2&!SNAI1&!SNAI2 | !E2F&!Bcatenin&!YAP1&HNF1A&!HNF4A&!HNF6&FOXA2&!NFkB&!SNAI1 | !E2F&!Bcatenin&!YAP1&HNF1A&!HNF4A&!HNF6&FOXA2&!NFkB&SNAI1&!SNAI2 | !E2F&!Bcatenin&!YAP1&HNF1A&!HNF4A&!HNF6&FOXA2&NFkB&!SNAI1&!SNAI2 | !E2F&!Bcatenin&!YAP1&HNF1A&!HNF4A&!HNF6&FOXA2&NFkB&!SNAI1&SNAI2&!p53 | !E2F&!Bcatenin&!YAP1&HNF1A&!HNF4A&!HNF6&FOXA2&NFkB&SNAI1&!SNAI2&!p53 | !E2F&!Bcatenin&!YAP1&HNF1A&!HNF4A&HNF6&!NFkB&!SNAI1 | !E2F&!Bcatenin&!YAP1&HNF1A&!HNF4A&HNF6&!NFkB&SNAI1&!SNAI2 | !E2F&!Bcatenin&!YAP1&HNF1A&!HNF4A&HNF6&NFkB&!SNAI1&!SNAI2 | !E2F&!Bcatenin&!YAP1&HNF1A&!HNF4A&HNF6&NFkB&!SNAI1&SNAI2&!p53 | !E2F&!Bcatenin&!YAP1&HNF1A&!HNF4A&HNF6&NFkB&SNAI1&!SNAI2&!p53 | !E2F&!Bcatenin&!YAP1&HNF1A&HNF4A&!SNAI1 | !E2F&!Bcatenin&!YAP1&HNF1A&HNF4A&SNAI1&!SNAI2 | !E2F&!Bcatenin&YAP1&!HNF1A&!HNF4A&!HNF6&!FOXA2&!NFkB&!SNAI1&!ZEB1&!SNAI2&!SOX9&!NANOG&SOX2&!p53 | !E2F&!Bcatenin&YAP1&!HNF1A&!HNF4A&!HNF6&!FOXA2&!NFkB&!SNAI1&!ZEB1&!SNAI2&SOX9&!NANOG&SOX2 | !E2F&!Bcatenin&YAP1&!HNF1A&!HNF4A&!HNF6&!FOXA2&!NFkB&!SNAI1&ZEB1&!SNAI2&!NANOG&SOX2&!p53 | !E2F&!Bcatenin&YAP1&!HNF1A&!HNF4A&!HNF6&FOXA2&!NFkB&!SNAI1&!SNAI2 | !E2F&!Bcatenin&YAP1&!HNF1A&!HNF4A&!HNF6&FOXA2&NFkB&!SNAI1&!SNAI2&!p53 | !E2F&!Bcatenin&YAP1&!HNF1A&!HNF4A&HNF6&!NFkB&!SNAI1&!SNAI2 | !E2F&!Bcatenin&YAP1&!HNF1A&!HNF4A&HNF6&NFkB&!SNAI1&!SNAI2&!p53 | !E2F&!Bcatenin&YAP1&!HNF1A&HNF4A&!HNF6&!FOXA2&!NFkB&!SNAI1&!SNAI2 | !E2F&!Bcatenin&YAP1&!HNF1A&HNF4A&!HNF6&!FOXA2&NFkB&!SNAI1&!SNAI2&!p53 | !E2F&!Bcatenin&YAP1&!HNF1A&HNF4A&!HNF6&FOXA2&!SNAI1 | !E2F&!Bcatenin&YAP1&!HNF1A&HNF4A&!HNF6&FOXA2&SNAI1&!SNAI2 | !E2F&!Bcatenin&YAP1&!HNF1A&HNF4A&HNF6&!SNAI1 | !E2F&!Bcatenin&YAP1&!HNF1A&HNF4A&HNF6&SNAI1&!SNAI2 | !E2F&!Bcatenin&YAP1&HNF1A&!HNF4A&!NFkB&!SNAI1&!SNAI2 | !E2F&!Bcatenin&YAP1&HNF1A&!HNF4A&NFkB&!SNAI1&!SNAI2&!p53 | !E2F&!Bcatenin&YAP1&HNF1A&HNF4A&!SNAI1 | !E2F&!Bcatenin&YAP1&HNF1A&HNF4A&SNAI1&!SNAI2 | !E2F&Bcatenin&!YAP1&!HNF1A&!HNF4A&!HNF6&!FOXA2&!SNAI1&!ZEB1&!SNAI2&SOX9&!NANOG&SOX2&p53 | !E2F&Bcatenin&!YAP1&!HNF1A&!HNF4A&!HNF6&FOXA2&!SNAI1&!SNAI2 | !E2F&Bcatenin&!YAP1&!HNF1A&!HNF4A&HNF6&!SNAI1&!SNAI2 | !E2F&Bcatenin&!YAP1&!HNF1A&HNF4A&!HNF6&!FOXA2&!SNAI1&!SNAI2 | !E2F&Bcatenin&!YAP1&!HNF1A&HNF4A&!HNF6&FOXA2&!SNAI1 | !E2F&Bcatenin&!YAP1&!HNF1A&HNF4A&!HNF6&FOXA2&SNAI1&!SNAI2 | !E2F&Bcatenin&!YAP1&!HNF1A&HNF4A&HNF6&!SNAI1 | !E2F&Bcatenin&!YAP1&!HNF1A&HNF4A&HNF6&SNAI1&!SNAI2 | !E2F&Bcatenin&!YAP1&HNF1A&!HNF4A&!HNF6&!FOXA2&!SNAI1&!SNAI2 | !E2F&Bcatenin&!YAP1&HNF1A&!HNF4A&!HNF6&FOXA2&!NFkB&!SNAI1 | !E2F&Bcatenin&!YAP1&HNF1A&!HNF4A&!HNF6&FOXA2&!NFkB&SNAI1&!SNAI2 | !E2F&Bcatenin&!YAP1&HNF1A&!HNF4A&!HNF6&FOXA2&NFkB&!SNAI1&!SNAI2 | !E2F&Bcatenin&!YAP1&HNF1A&!HNF4A&!HNF6&FOXA2&NFkB&!SNAI1&SNAI2&!p53 | !E2F&Bcatenin&!YAP1&HNF1A&!HNF4A&!HNF6&FOXA2&NFkB&SNAI1&!SNAI2&!p53 | !E2F&Bcatenin&!YAP1&HNF1A&!HNF4A&HNF6&!NFkB&!SNAI1 | !E2F&Bcatenin&!YAP1&HNF1A&!HNF4A&HNF6&!NFkB&SNAI1&!SNAI2 | !E2F&Bcatenin&!YAP1&HNF1A&!HNF4A&HNF6&NFkB&!SNAI1&!SNAI2 | !E2F&Bcatenin&!YAP1&HNF1A&!HNF4A&HNF6&NFkB&!SNAI1&SNAI2&!p53 | !E2F&Bcatenin&!YAP1&HNF1A&!HNF4A&HNF6&NFkB&SNAI1&!SNAI2&!p53 | !E2F&Bcatenin&!YAP1&HNF1A&HNF4A&!SNAI1 | !E2F&Bcatenin&!YAP1&HNF1A&HNF4A&SNAI1&!SNAI2 | !E2F&Bcatenin&YAP1&!HNF1A&!HNF4A&!HNF6&!FOXA2&!NFkB&!SNAI1&!ZEB1&!SNAI2&SOX9&!NANOG&SOX2&p53 | !E2F&Bcatenin&YAP1&!HNF1A&!HNF4A&!HNF6&FOXA2&!NFkB&!SNAI1&!SNAI2 | !E2F&Bcatenin&YAP1&!HNF1A&!HNF4A&!HNF6&FOXA2&NFkB&!SNAI1&!SNAI2&!p53 | !E2F&Bcatenin&YAP1&!HNF1A&!HNF4A&HNF6&!NFkB&!SNAI1&!SNAI2 | !E2F&Bcatenin&YAP1&!HNF1A&!HNF4A&HNF6&NFkB&!SNAI1&!SNAI2&!p53 | !E2F&Bcatenin&YAP1&!HNF1A&HNF4A&!HNF6&!FOXA2&!NFkB&!SNAI1&!SNAI2 | !E2F&Bcatenin&YAP1&!HNF1A&HNF4A&!HNF6&!FOXA2&NFkB&!SNAI1&!SNAI2&!p53 | !E2F&Bcatenin&YAP1&!HNF1A&HNF4A&!HNF6&FOXA2&!SNAI1 | !E2F&Bcatenin&YAP1&!HNF1A&HNF4A&!HNF6&FOXA2&SNAI1&!SNAI2 | !E2F&Bcatenin&YAP1&!HNF1A&HNF4A&HNF6&!SNAI1 | !E2F&Bcatenin&YAP1&!HNF1A&HNF4A&HNF6&SNAI1&!SNAI2 | !E2F&Bcatenin&YAP1&HNF1A&!HNF4A&!NFkB&!SNAI1&!SNAI2 | !E2F&Bcatenin&YAP1&HNF1A&!HNF4A&NFkB&!SNAI1&!SNAI2&!p53 | !E2F&Bcatenin&YAP1&HNF1A&HNF4A&!SNAI1 | !E2F&Bcatenin&YAP1&HNF1A&HNF4A&SNAI1&!SNAI2 | E2F&!YAP1&!HNF1A&!HNF4A&!HNF6&!FOXA2&!SNAI1&!ZEB1&!SNAI2&SOX9&!NANOG&SOX2&p53 | E2F&!YAP1&!HNF1A&!HNF4A&!HNF6&FOXA2&!SNAI1&!SNAI2 | E2F&!YAP1&!HNF1A&!HNF4A&HNF6&!SNAI1&!SNAI2 | E2F&!YAP1&!HNF1A&HNF4A&!HNF6&!FOXA2&!SNAI1&!SNAI2 | E2F&!YAP1&!HNF1A&HNF4A&!HNF6&FOXA2&!SNAI1 | E2F&!YAP1&!HNF1A&HNF4A&!HNF6&FOXA2&SNAI1&!SNAI2 | E2F&!YAP1&!HNF1A&HNF4A&HNF6&!SNAI1 | E2F&!YAP1&!HNF1A&HNF4A&HNF6&SNAI1&!SNAI2 | E2F&!YAP1&HNF1A&!HNF4A&!HNF6&!FOXA2&!SNAI1&!SNAI2 | E2F&!YAP1&HNF1A&!HNF4A&!HNF6&FOXA2&!NFkB&!SNAI1 | E2F&!YAP1&HNF1A&!HNF4A&!HNF6&FOXA2&!NFkB&SNAI1&!SNAI2 | E2F&!YAP1&HNF1A&!HNF4A&!HNF6&FOXA2&NFkB&!SNAI1&!SNAI2 | E2F&!YAP1&HNF1A&!HNF4A&!HNF6&FOXA2&NFkB&!SNAI1&SNAI2&!p53 | E2F&!YAP1&HNF1A&!HNF4A&!HNF6&FOXA2&NFkB&SNAI1&!SNAI2&!p53 | E2F&!YAP1&HNF1A&!HNF4A&HNF6&!NFkB&!SNAI1 | E2F&!YAP1&HNF1A&!HNF4A&HNF6&!NFkB&SNAI1&!SNAI2 | E2F&!YAP1&HNF1A&!HNF4A&HNF6&NFkB&!SNAI1&!SNAI2 | E2F&!YAP1&HNF1A&!HNF4A&HNF6&NFkB&!SNAI1&SNAI2&!p53 | E2F&!YAP1&HNF1A&!HNF4A&HNF6&NFkB&SNAI1&!SNAI2&!p53 | E2F&!YAP1&HNF1A&HNF4A&!SNAI1 | E2F&!YAP1&HNF1A&HNF4A&SNAI1&!SNAI2 | E2F&YAP1&!HNF1A&!HNF4A&!HNF6&!FOXA2&!NFkB&!SNAI1&!ZEB1&!SNAI2&SOX9&!NANOG&SOX2&p53 | E2F&YAP1&!HNF1A&!HNF4A&!HNF6&FOXA2&!NFkB&!SNAI1&!SNAI2 | E2F&YAP1&!HNF1A&!HNF4A&!HNF6&FOXA2&NFkB&!SNAI1&!SNAI2&!p53 | E2F&YAP1&!HNF1A&!HNF4A&HNF6&!NFkB&!SNAI1&!SNAI2 | E2F&YAP1&!HNF1A&!HNF4A&HNF6&NFkB&!SNAI1&!SNAI2&!p53 | E2F&YAP1&!HNF1A&HNF4A&!HNF6&!FOXA2&!NFkB&!SNAI1&!SNAI2 | E2F&YAP1&!HNF1A&HNF4A&!HNF6&!FOXA2&NFkB&!SNAI1&!SNAI2&!p53 | E2F&YAP1&!HNF1A&HNF4A&!HNF6&FOXA2&!SNAI1 | E2F&YAP1&!HNF1A&HNF4A&!HNF6&FOXA2&SNAI1&!SNAI2 | E2F&YAP1&!HNF1A&HNF4A&HNF6&!SNAI1 | E2F&YAP1&!HNF1A&HNF4A&HNF6&SNAI1&!SNAI2 | E2F&YAP1&HNF1A&!HNF4A&!NFkB&!SNAI1&!SNAI2 | E2F&YAP1&HNF1A&!HNF4A&NFkB&!SNAI1&!SNAI2&!p53 | E2F&YAP1&HNF1A&HNF4A&!SNAI1 | E2F&YAP1&HNF1A&HNF4A&SNAI1&!SNAI2

HNF6, !HNF1A&HNF4A&!HNF6&!SNAI1&!SOX9&!OCT4&!NANOG&!SOX2 | !HNF1A&HNF4A&!HNF6&!SNAI1&!SOX9&!OCT4&!NANOG&SOX2&p53 | !HNF1A&HNF4A&!HNF6&!SNAI1&!SOX9&!OCT4&NANOG | !HNF1A&HNF4A&!HNF6&!SNAI1&!SOX9&OCT4&!NANOG&p53 | !HNF1A&HNF4A&!HNF6&!SNAI1&!SOX9&OCT4&NANOG&!SOX2 | !HNF1A&HNF4A&!HNF6&!SNAI1&SOX9&!OCT4 | !HNF1A&HNF4A&!HNF6&!SNAI1&SOX9&OCT4&!NANOG | !HNF1A&HNF4A&!HNF6&!SNAI1&SOX9&OCT4&NANOG&!SOX2 | !HNF1A&HNF4A&!HNF6&SNAI1&!SOX9&!OCT4&!NANOG&!SOX2 | !HNF1A&HNF4A&!HNF6&SNAI1&SOX9&!OCT4 | !HNF1A&HNF4A&!HNF6&SNAI1&SOX9&OCT4&!NANOG | !HNF1A&HNF4A&!HNF6&SNAI1&SOX9&OCT4&NANOG&!SOX2 | !HNF1A&HNF4A&HNF6&!OCT4 | !HNF1A&HNF4A&HNF6&OCT4&!NANOG | !HNF1A&HNF4A&HNF6&OCT4&NANOG&!SOX2 | HNF1A&!HNF6&!SNAI1&!SOX9&!OCT4&!NANOG&!SOX2 | HNF1A&!HNF6&!SNAI1&!SOX9&!OCT4&!NANOG&SOX2&p53 | HNF1A&!HNF6&!SNAI1&!SOX9&!OCT4&NANOG | HNF1A&!HNF6&!SNAI1&!SOX9&OCT4&!NANOG&p53 | HNF1A&!HNF6&!SNAI1&!SOX9&OCT4&NANOG&!SOX2 | HNF1A&!HNF6&!SNAI1&SOX9&!OCT4 | HNF1A&!HNF6&!SNAI1&SOX9&OCT4&!NANOG | HNF1A&!HNF6&!SNAI1&SOX9&OCT4&NANOG&!SOX2 | HNF1A&!HNF6&SNAI1&!SOX9&!OCT4&!NANOG&!SOX2 | HNF1A&!HNF6&SNAI1&SOX9&!OCT4 | HNF1A&!HNF6&SNAI1&SOX9&OCT4&!NANOG | HNF1A&!HNF6&SNAI1&SOX9&OCT4&NANOG&!SOX2 | HNF1A&HNF6&!OCT4 | HNF1A&HNF6&OCT4&!NANOG | HNF1A&HNF6&OCT4&NANOG&!SOX2

HNF1A, !HNF1A&!HNF4A&FOXA2&!NFkB&!SNAI1 | !HNF1A&HNF4A&!FOXA2&!SNAI1 | !HNF1A&HNF4A&FOXA2 | HNF1A&!HNF4A&!NFkB&!SNAI1 | HNF1A&HNF4A

FOXA2, !Bcatenin&!HNF4A&HNF6&!FOXA2&SOX9&!SOX2 | !Bcatenin&!HNF4A&HNF6&FOXA2&!SOX2 | !Bcatenin&HNF4A&!HNF6&!FOXA2&!ZEB1&!SNAI2&!TGFB&SOX9&!SOX2 | !Bcatenin&HNF4A&!HNF6&!FOXA2&!ZEB1&SNAI2&SOX9&!SOX2 | !Bcatenin&HNF4A&!HNF6&!FOXA2&ZEB1&SOX9&!SOX2 | !Bcatenin&HNF4A&!HNF6&FOXA2&!ZEB1&!SNAI2&!TGFB&!SOX2 | !Bcatenin&HNF4A&!HNF6&FOXA2&!ZEB1&SNAI2&!SOX2 | !Bcatenin&HNF4A&!HNF6&FOXA2&ZEB1&!SOX2 | !Bcatenin&HNF4A&HNF6&!FOXA2&!ZEB1&!SNAI2&!TGFB&!SOX2 | !Bcatenin&HNF4A&HNF6&!FOXA2&!ZEB1&SNAI2&!TGFB&!SOX2 | !Bcatenin&HNF4A&HNF6&!FOXA2&!ZEB1&SNAI2&TGFB&SOX9&!SOX2 | !Bcatenin&HNF4A&HNF6&!FOXA2&ZEB1&!TGFB&!SOX2 | !Bcatenin&HNF4A&HNF6&!FOXA2&ZEB1&TGFB&SOX9&!SOX2 | !Bcatenin&HNF4A&HNF6&FOXA2&!ZEB1&!SNAI2&!TGFB&!SOX2 | !Bcatenin&HNF4A&HNF6&FOXA2&!ZEB1&SNAI2&!SOX2 | !Bcatenin&HNF4A&HNF6&FOXA2&ZEB1&!SOX2 | Bcatenin&!HNF4A&HNF6&!SOX2 | Bcatenin&HNF4A&!ZEB1&!SNAI2&!TGFB&!SOX2 | Bcatenin&HNF4A&!ZEB1&SNAI2&!SOX2 | Bcatenin&HNF4A&ZEB1&!SOX2

NFkB, !YAP1&!HNF1A | !YAP1&HNF1A&!HNF4A | !YAP1&HNF1A&HNF4A&!FOXA2&!NFkB&!SNAI1&!ZEB1&!TGFB&!SOX9 | !YAP1&HNF1A&HNF4A&!FOXA2&!NFkB&!SNAI1&!ZEB1&!TGFB&SOX9&!p53 | !YAP1&HNF1A&HNF4A&!FOXA2&!NFkB&!SNAI1&!ZEB1&TGFB | !YAP1&HNF1A&HNF4A&!FOXA2&!NFkB&!SNAI1&ZEB1 | !YAP1&HNF1A&HNF4A&!FOXA2&!NFkB&SNAI1 | !YAP1&HNF1A&HNF4A&!FOXA2&NFkB | !YAP1&HNF1A&HNF4A&FOXA2&!NFkB&!SNAI1&!ZEB1&!TGFB&!SOX9 | !YAP1&HNF1A&HNF4A&FOXA2&!NFkB&!SNAI1&!ZEB1&!TGFB&SOX9&!p53 | !YAP1&HNF1A&HNF4A&FOXA2&!NFkB&!SNAI1&!ZEB1&TGFB | !YAP1&HNF1A&HNF4A&FOXA2&!NFkB&!SNAI1&ZEB1 | !YAP1&HNF1A&HNF4A&FOXA2&!NFkB&SNAI1 | !YAP1&HNF1A&HNF4A&FOXA2&NFkB&!SNAI1&!ZEB1&!SOX9 | !YAP1&HNF1A&HNF4A&FOXA2&NFkB&!SNAI1&!ZEB1&SOX9&!p53 | !YAP1&HNF1A&HNF4A&FOXA2&NFkB&!SNAI1&ZEB1 | !YAP1&HNF1A&HNF4A&FOXA2&NFkB&SNAI1 | YAP1

SNAI1, !E2F&!Bcatenin&!YAP1&!HNF1A&!HNF4A&!NFkB&!SNAI1&!ZEB1&TGFB&!p53 | !E2F&!Bcatenin&!YAP1&!HNF1A&!HNF4A&!NFkB&!SNAI1&ZEB1&TGFB | !E2F&!Bcatenin&!YAP1&!HNF1A&!HNF4A&!NFkB&SNAI1&!SNAI2&TGFB | !E2F&!Bcatenin&!YAP1&!HNF1A&!HNF4A&!NFkB&SNAI1&SNAI2&TGFB&!NANOG&!SOX2 | !E2F&!Bcatenin&!YAP1&!HNF1A&!HNF4A&!NFkB&SNAI1&SNAI2&TGFB&NANOG | !E2F&!Bcatenin&!YAP1&!HNF1A&!HNF4A&NFkB | !E2F&!Bcatenin&!YAP1&!HNF1A&HNF4A&!NFkB&!SNAI1&ZEB1&TGFB | !E2F&!Bcatenin&!YAP1&!HNF1A&HNF4A&!NFkB&SNAI1&!SNAI2&TGFB | !E2F&!Bcatenin&!YAP1&!HNF1A&HNF4A&!NFkB&SNAI1&SNAI2&TGFB&!NANOG&!SOX2 | !E2F&!Bcatenin&!YAP1&!HNF1A&HNF4A&!NFkB&SNAI1&SNAI2&TGFB&NANOG | !E2F&!Bcatenin&!YAP1&!HNF1A&HNF4A&NFkB&!SNAI1&ZEB1 | !E2F&!Bcatenin&!YAP1&!HNF1A&HNF4A&NFkB&SNAI1 | !E2F&!Bcatenin&!YAP1&HNF1A&!HNF4A&!NFkB&!SNAI1&!ZEB1&TGFB&!p53 | !E2F&!Bcatenin&!YAP1&HNF1A&!HNF4A&!NFkB&!SNAI1&ZEB1&TGFB | !E2F&!Bcatenin&!YAP1&HNF1A&!HNF4A&!NFkB&SNAI1&!SNAI2&TGFB | !E2F&!Bcatenin&!YAP1&HNF1A&!HNF4A&!NFkB&SNAI1&SNAI2&TGFB&!NANOG&!SOX2 | !E2F&!Bcatenin&!YAP1&HNF1A&!HNF4A&!NFkB&SNAI1&SNAI2&TGFB&NANOG | !E2F&!Bcatenin&!YAP1&HNF1A&!HNF4A&NFkB&!SNAI1&!ZEB1&!p53 | !E2F&!Bcatenin&!YAP1&HNF1A&!HNF4A&NFkB&!SNAI1&ZEB1 | !E2F&!Bcatenin&!YAP1&HNF1A&!HNF4A&NFkB&SNAI1&!SNAI2 | !E2F&!Bcatenin&!YAP1&HNF1A&!HNF4A&NFkB&SNAI1&SNAI2&!NANOG&!SOX2 | !E2F&!Bcatenin&!YAP1&HNF1A&!HNF4A&NFkB&SNAI1&SNAI2&NANOG | !E2F&!Bcatenin&YAP1&!HNF1A&!HNF4A&!NFkB&!SNAI1&!ZEB1&!OCT4&!NANOG&!p53 | !E2F&!Bcatenin&YAP1&!HNF1A&!HNF4A&!NFkB&!SNAI1&!ZEB1&!OCT4&NANOG | !E2F&!Bcatenin&YAP1&!HNF1A&!HNF4A&!NFkB&!SNAI1&!ZEB1&OCT4 | !E2F&!Bcatenin&YAP1&!HNF1A&!HNF4A&!NFkB&!SNAI1&ZEB1 | !E2F&!Bcatenin&YAP1&!HNF1A&!HNF4A&!NFkB&SNAI1&!SNAI2 | !E2F&!Bcatenin&YAP1&!HNF1A&!HNF4A&!NFkB&SNAI1&SNAI2&!OCT4&!NANOG&!SOX2 | !E2F&!Bcatenin&YAP1&!HNF1A&!HNF4A&!NFkB&SNAI1&SNAI2&!OCT4&NANOG | !E2F&!Bcatenin&YAP1&!HNF1A&!HNF4A&!NFkB&SNAI1&SNAI2&OCT4 | !E2F&!Bcatenin&YAP1&!HNF1A&!HNF4A&NFkB | !E2F&!Bcatenin&YAP1&!HNF1A&HNF4A&!NFkB&!SNAI1&!ZEB1&!OCT4&NANOG | !E2F&!Bcatenin&YAP1&!HNF1A&HNF4A&!NFkB&!SNAI1&!ZEB1&OCT4 | !E2F&!Bcatenin&YAP1&!HNF1A&HNF4A&!NFkB&!SNAI1&ZEB1 | !E2F&!Bcatenin&YAP1&!HNF1A&HNF4A&!NFkB&SNAI1&!SNAI2 | !E2F&!Bcatenin&YAP1&!HNF1A&HNF4A&!NFkB&SNAI1&SNAI2&!OCT4&!NANOG&!SOX2 | !E2F&!Bcatenin&YAP1&!HNF1A&HNF4A&!NFkB&SNAI1&SNAI2&!OCT4&NANOG | !E2F&!Bcatenin&YAP1&!HNF1A&HNF4A&!NFkB&SNAI1&SNAI2&OCT4 | !E2F&!Bcatenin&YAP1&!HNF1A&HNF4A&NFkB&!SNAI1&!ZEB1&!TGFB&!OCT4&NANOG | !E2F&!Bcatenin&YAP1&!HNF1A&HNF4A&NFkB&!SNAI1&!ZEB1&!TGFB&OCT4 | !E2F&!Bcatenin&YAP1&!HNF1A&HNF4A&NFkB&!SNAI1&!ZEB1&TGFB | !E2F&!Bcatenin&YAP1&!HNF1A&HNF4A&NFkB&!SNAI1&ZEB1 | !E2F&!Bcatenin&YAP1&!HNF1A&HNF4A&NFkB&SNAI1 | !E2F&!Bcatenin&YAP1&HNF1A&!HNF4A&!NFkB&!SNAI1&!ZEB1&!OCT4&!NANOG&!p53 | !E2F&!Bcatenin&YAP1&HNF1A&!HNF4A&!NFkB&!SNAI1&!ZEB1&!OCT4&NANOG | !E2F&!Bcatenin&YAP1&HNF1A&!HNF4A&!NFkB&!SNAI1&!ZEB1&OCT4 | !E2F&!Bcatenin&YAP1&HNF1A&!HNF4A&!NFkB&!SNAI1&ZEB1 | !E2F&!Bcatenin&YAP1&HNF1A&!HNF4A&!NFkB&SNAI1&!SNAI2 | !E2F&!Bcatenin&YAP1&HNF1A&!HNF4A&!NFkB&SNAI1&SNAI2&!OCT4&!NANOG&!SOX2 | !E2F&!Bcatenin&YAP1&HNF1A&!HNF4A&!NFkB&SNAI1&SNAI2&!OCT4&NANOG | !E2F&!Bcatenin&YAP1&HNF1A&!HNF4A&!NFkB&SNAI1&SNAI2&OCT4 | !E2F&!Bcatenin&YAP1&HNF1A&!HNF4A&NFkB&!SNAI1&!ZEB1&!TGFB&!OCT4&!NANOG&!p53 | !E2F&!Bcatenin&YAP1&HNF1A&!HNF4A&NFkB&!SNAI1&!ZEB1&!TGFB&!OCT4&NANOG | !E2F&!Bcatenin&YAP1&HNF1A&!HNF4A&NFkB&!SNAI1&!ZEB1&!TGFB&OCT4 | !E2F&!Bcatenin&YAP1&HNF1A&!HNF4A&NFkB&!SNAI1&!ZEB1&TGFB | !E2F&!Bcatenin&YAP1&HNF1A&!HNF4A&NFkB&!SNAI1&ZEB1 | !E2F&!Bcatenin&YAP1&HNF1A&!HNF4A&NFkB&SNAI1&!SNAI2 | !E2F&!Bcatenin&YAP1&HNF1A&!HNF4A&NFkB&SNAI1&SNAI2&!TGFB&!OCT4&!NANOG&!SOX2 | !E2F&!Bcatenin&YAP1&HNF1A&!HNF4A&NFkB&SNAI1&SNAI2&!TGFB&!OCT4&!NANOG&SOX2&!p53 | !E2F&!Bcatenin&YAP1&HNF1A&!HNF4A&NFkB&SNAI1&SNAI2&!TGFB&!OCT4&NANOG | !E2F&!Bcatenin&YAP1&HNF1A&!HNF4A&NFkB&SNAI1&SNAI2&!TGFB&OCT4 | !E2F&!Bcatenin&YAP1&HNF1A&!HNF4A&NFkB&SNAI1&SNAI2&TGFB | !E2F&!Bcatenin&YAP1&HNF1A&HNF4A&NFkB&TGFB | !E2F&Bcatenin&!YAP1&!HNF1A&!HNF4A&!NFkB&!SNAI1&!ZEB1&!TGFB&SOX2&!p53 | !E2F&Bcatenin&!YAP1&!HNF1A&!HNF4A&!NFkB&!SNAI1&!ZEB1&TGFB&!p53 | !E2F&Bcatenin&!YAP1&!HNF1A&!HNF4A&!NFkB&!SNAI1&ZEB1&!TGFB&SOX2&!p53 | !E2F&Bcatenin&!YAP1&!HNF1A&!HNF4A&!NFkB&!SNAI1&ZEB1&TGFB | !E2F&Bcatenin&!YAP1&!HNF1A&!HNF4A&!NFkB&SNAI1&!SNAI2&!TGFB&SOX2&!p53 | !E2F&Bcatenin&!YAP1&!HNF1A&!HNF4A&!NFkB&SNAI1&!SNAI2&TGFB | !E2F&Bcatenin&!YAP1&!HNF1A&!HNF4A&!NFkB&SNAI1&SNAI2&!TGFB&SOX2&!p53 | !E2F&Bcatenin&!YAP1&!HNF1A&!HNF4A&!NFkB&SNAI1&SNAI2&TGFB&!NANOG&!SOX2 | !E2F&Bcatenin&!YAP1&!HNF1A&!HNF4A&!NFkB&SNAI1&SNAI2&TGFB&!NANOG&SOX2&!p53 | !E2F&Bcatenin&!YAP1&!HNF1A&!HNF4A&!NFkB&SNAI1&SNAI2&TGFB&NANOG | !E2F&Bcatenin&!YAP1&!HNF1A&!HNF4A&NFkB | !E2F&Bcatenin&!YAP1&!HNF1A&HNF4A&!NFkB&!SNAI1&!ZEB1&!TGFB&!OCT4&NANOG&SOX2&!p53 | !E2F&Bcatenin&!YAP1&!HNF1A&HNF4A&!NFkB&!SNAI1&!ZEB1&!TGFB&OCT4&SOX2&!p53 | !E2F&Bcatenin&!YAP1&!HNF1A&HNF4A&!NFkB&!SNAI1&ZEB1&!TGFB&SOX2&!p53 | !E2F&Bcatenin&!YAP1&!HNF1A&HNF4A&!NFkB&!SNAI1&ZEB1&TGFB | !E2F&Bcatenin&!YAP1&!HNF1A&HNF4A&!NFkB&SNAI1&!SNAI2&!TGFB&SOX2&!p53 | !E2F&Bcatenin&!YAP1&!HNF1A&HNF4A&!NFkB&SNAI1&!SNAI2&TGFB | !E2F&Bcatenin&!YAP1&!HNF1A&HNF4A&!NFkB&SNAI1&SNAI2&!TGFB&SOX2&!p53 | !E2F&Bcatenin&!YAP1&!HNF1A&HNF4A&!NFkB&SNAI1&SNAI2&TGFB&!NANOG&!SOX2 | !E2F&Bcatenin&!YAP1&!HNF1A&HNF4A&!NFkB&SNAI1&SNAI2&TGFB&!NANOG&SOX2&!p53 | !E2F&Bcatenin&!YAP1&!HNF1A&HNF4A&!NFkB&SNAI1&SNAI2&TGFB&NANOG | !E2F&Bcatenin&!YAP1&!HNF1A&HNF4A&NFkB&!SNAI1&!ZEB1&!TGFB&!OCT4&NANOG&SOX2&!p53 | !E2F&Bcatenin&!YAP1&!HNF1A&HNF4A&NFkB&!SNAI1&!ZEB1&!TGFB&OCT4&SOX2&!p53 | !E2F&Bcatenin&!YAP1&!HNF1A&HNF4A&NFkB&!SNAI1&ZEB1 | !E2F&Bcatenin&!YAP1&!HNF1A&HNF4A&NFkB&SNAI1 | !E2F&Bcatenin&!YAP1&HNF1A&!HNF4A&!NFkB&!SNAI1&!ZEB1&!TGFB&SOX2&!p53 | !E2F&Bcatenin&!YAP1&HNF1A&!HNF4A&!NFkB&!SNAI1&!ZEB1&TGFB&!p53 | !E2F&Bcatenin&!YAP1&HNF1A&!HNF4A&!NFkB&!SNAI1&ZEB1&!TGFB&SOX2&!p53 | !E2F&Bcatenin&!YAP1&HNF1A&!HNF4A&!NFkB&!SNAI1&ZEB1&TGFB | !E2F&Bcatenin&!YAP1&HNF1A&!HNF4A&!NFkB&SNAI1&!SNAI2&!TGFB&SOX2&!p53 | !E2F&Bcatenin&!YAP1&HNF1A&!HNF4A&!NFkB&SNAI1&!SNAI2&TGFB | !E2F&Bcatenin&!YAP1&HNF1A&!HNF4A&!NFkB&SNAI1&SNAI2&!TGFB&SOX2&!p53 | !E2F&Bcatenin&!YAP1&HNF1A&!HNF4A&!NFkB&SNAI1&SNAI2&TGFB&!NANOG&!SOX2 | !E2F&Bcatenin&!YAP1&HNF1A&!HNF4A&!NFkB&SNAI1&SNAI2&TGFB&!NANOG&SOX2&!p53 | !E2F&Bcatenin&!YAP1&HNF1A&!HNF4A&!NFkB&SNAI1&SNAI2&TGFB&NANOG | !E2F&Bcatenin&!YAP1&HNF1A&!HNF4A&NFkB&!SNAI1&!ZEB1&!p53 | !E2F&Bcatenin&!YAP1&HNF1A&!HNF4A&NFkB&!SNAI1&ZEB1 | !E2F&Bcatenin&!YAP1&HNF1A&!HNF4A&NFkB&SNAI1&!SNAI2 | !E2F&Bcatenin&!YAP1&HNF1A&!HNF4A&NFkB&SNAI1&SNAI2&!NANOG&!SOX2 | !E2F&Bcatenin&!YAP1&HNF1A&!HNF4A&NFkB&SNAI1&SNAI2&!NANOG&SOX2&!p53 | !E2F&Bcatenin&!YAP1&HNF1A&!HNF4A&NFkB&SNAI1&SNAI2&NANOG | !E2F&Bcatenin&!YAP1&HNF1A&HNF4A&NFkB&!SNAI1&ZEB1&TGFB&SOX2&!p53 | !E2F&Bcatenin&!YAP1&HNF1A&HNF4A&NFkB&SNAI1&TGFB&SOX2&!p53 | !E2F&Bcatenin&YAP1&!HNF1A&!HNF4A&!NFkB&!SNAI1&!ZEB1&!OCT4&!NANOG&!p53 | !E2F&Bcatenin&YAP1&!HNF1A&!HNF4A&!NFkB&!SNAI1&!ZEB1&!OCT4&NANOG | !E2F&Bcatenin&YAP1&!HNF1A&!HNF4A&!NFkB&!SNAI1&!ZEB1&OCT4 | !E2F&Bcatenin&YAP1&!HNF1A&!HNF4A&!NFkB&!SNAI1&ZEB1 | !E2F&Bcatenin&YAP1&!HNF1A&!HNF4A&!NFkB&SNAI1&!SNAI2 | !E2F&Bcatenin&YAP1&!HNF1A&!HNF4A&!NFkB&SNAI1&SNAI2&!OCT4&!NANOG&!SOX2 | !E2F&Bcatenin&YAP1&!HNF1A&!HNF4A&!NFkB&SNAI1&SNAI2&!OCT4&!NANOG&SOX2&!p53 | !E2F&Bcatenin&YAP1&!HNF1A&!HNF4A&!NFkB&SNAI1&SNAI2&!OCT4&NANOG | !E2F&Bcatenin&YAP1&!HNF1A&!HNF4A&!NFkB&SNAI1&SNAI2&OCT4 | !E2F&Bcatenin&YAP1&!HNF1A&!HNF4A&NFkB | !E2F&Bcatenin&YAP1&!HNF1A&HNF4A&!NFkB&!SNAI1&!ZEB1&!OCT4&NANOG | !E2F&Bcatenin&YAP1&!HNF1A&HNF4A&!NFkB&!SNAI1&!ZEB1&OCT4 | !E2F&Bcatenin&YAP1&!HNF1A&HNF4A&!NFkB&!SNAI1&ZEB1 | !E2F&Bcatenin&YAP1&!HNF1A&HNF4A&!NFkB&SNAI1&!SNAI2 | !E2F&Bcatenin&YAP1&!HNF1A&HNF4A&!NFkB&SNAI1&SNAI2&!OCT4&!NANOG&!SOX2 | !E2F&Bcatenin&YAP1&!HNF1A&HNF4A&!NFkB&SNAI1&SNAI2&!OCT4&!NANOG&SOX2&!p53 | !E2F&Bcatenin&YAP1&!HNF1A&HNF4A&!NFkB&SNAI1&SNAI2&!OCT4&NANOG | !E2F&Bcatenin&YAP1&!HNF1A&HNF4A&!NFkB&SNAI1&SNAI2&OCT4 | !E2F&Bcatenin&YAP1&!HNF1A&HNF4A&NFkB&!SNAI1&!ZEB1&!TGFB&!OCT4&NANOG | !E2F&Bcatenin&YAP1&!HNF1A&HNF4A&NFkB&!SNAI1&!ZEB1&!TGFB&OCT4 | !E2F&Bcatenin&YAP1&!HNF1A&HNF4A&NFkB&!SNAI1&!ZEB1&TGFB | !E2F&Bcatenin&YAP1&!HNF1A&HNF4A&NFkB&!SNAI1&ZEB1 | !E2F&Bcatenin&YAP1&!HNF1A&HNF4A&NFkB&SNAI1 | !E2F&Bcatenin&YAP1&HNF1A&!HNF4A&!NFkB&!SNAI1&!ZEB1&!OCT4&!NANOG&!p53 | !E2F&Bcatenin&YAP1&HNF1A&!HNF4A&!NFkB&!SNAI1&!ZEB1&!OCT4&NANOG | !E2F&Bcatenin&YAP1&HNF1A&!HNF4A&!NFkB&!SNAI1&!ZEB1&OCT4 | !E2F&Bcatenin&YAP1&HNF1A&!HNF4A&!NFkB&!SNAI1&ZEB1 | !E2F&Bcatenin&YAP1&HNF1A&!HNF4A&!NFkB&SNAI1&!SNAI2 | !E2F&Bcatenin&YAP1&HNF1A&!HNF4A&!NFkB&SNAI1&SNAI2&!OCT4&!NANOG&!SOX2 | !E2F&Bcatenin&YAP1&HNF1A&!HNF4A&!NFkB&SNAI1&SNAI2&!OCT4&!NANOG&SOX2&!p53 | !E2F&Bcatenin&YAP1&HNF1A&!HNF4A&!NFkB&SNAI1&SNAI2&!OCT4&NANOG | !E2F&Bcatenin&YAP1&HNF1A&!HNF4A&!NFkB&SNAI1&SNAI2&OCT4 | !E2F&Bcatenin&YAP1&HNF1A&!HNF4A&NFkB&!SNAI1&!ZEB1&!TGFB&!OCT4&!NANOG&!p53 | !E2F&Bcatenin&YAP1&HNF1A&!HNF4A&NFkB&!SNAI1&!ZEB1&!TGFB&!OCT4&NANOG | !E2F&Bcatenin&YAP1&HNF1A&!HNF4A&NFkB&!SNAI1&!ZEB1&!TGFB&OCT4 | !E2F&Bcatenin&YAP1&HNF1A&!HNF4A&NFkB&!SNAI1&!ZEB1&TGFB | !E2F&Bcatenin&YAP1&HNF1A&!HNF4A&NFkB&!SNAI1&ZEB1 | !E2F&Bcatenin&YAP1&HNF1A&!HNF4A&NFkB&SNAI1&!SNAI2 | !E2F&Bcatenin&YAP1&HNF1A&!HNF4A&NFkB&SNAI1&SNAI2&!TGFB&!OCT4&!NANOG&!SOX2 | !E2F&Bcatenin&YAP1&HNF1A&!HNF4A&NFkB&SNAI1&SNAI2&!TGFB&!OCT4&!NANOG&SOX2&!p53 | !E2F&Bcatenin&YAP1&HNF1A&!HNF4A&NFkB&SNAI1&SNAI2&!TGFB&!OCT4&NANOG | !E2F&Bcatenin&YAP1&HNF1A&!HNF4A&NFkB&SNAI1&SNAI2&!TGFB&OCT4 | !E2F&Bcatenin&YAP1&HNF1A&!HNF4A&NFkB&SNAI1&SNAI2&TGFB | !E2F&Bcatenin&YAP1&HNF1A&HNF4A&NFkB&TGFB | E2F&!Bcatenin&!YAP1&!HNF1A&!HNF4A&!NFkB&!SNAI1&!ZEB1&!TGFB&SOX2&!p53 | E2F&!Bcatenin&!YAP1&!HNF1A&!HNF4A&!NFkB&!SNAI1&!ZEB1&TGFB&!p53 | E2F&!Bcatenin&!YAP1&!HNF1A&!HNF4A&!NFkB&!SNAI1&ZEB1&!TGFB&SOX2&!p53 | E2F&!Bcatenin&!YAP1&!HNF1A&!HNF4A&!NFkB&!SNAI1&ZEB1&TGFB | E2F&!Bcatenin&!YAP1&!HNF1A&!HNF4A&!NFkB&SNAI1&!SNAI2&!TGFB&SOX2&!p53 | E2F&!Bcatenin&!YAP1&!HNF1A&!HNF4A&!NFkB&SNAI1&!SNAI2&TGFB | E2F&!Bcatenin&!YAP1&!HNF1A&!HNF4A&!NFkB&SNAI1&SNAI2&!TGFB&SOX2&!p53 | E2F&!Bcatenin&!YAP1&!HNF1A&!HNF4A&!NFkB&SNAI1&SNAI2&TGFB&!NANOG&!SOX2 | E2F&!Bcatenin&!YAP1&!HNF1A&!HNF4A&!NFkB&SNAI1&SNAI2&TGFB&!NANOG&SOX2&!p53 | E2F&!Bcatenin&!YAP1&!HNF1A&!HNF4A&!NFkB&SNAI1&SNAI2&TGFB&NANOG | E2F&!Bcatenin&!YAP1&!HNF1A&!HNF4A&NFkB | E2F&!Bcatenin&!YAP1&!HNF1A&HNF4A&!NFkB&!SNAI1&!ZEB1&!TGFB&!OCT4&NANOG&SOX2&!p53 | E2F&!Bcatenin&!YAP1&!HNF1A&HNF4A&!NFkB&!SNAI1&!ZEB1&!TGFB&OCT4&SOX2&!p53 | E2F&!Bcatenin&!YAP1&!HNF1A&HNF4A&!NFkB&!SNAI1&ZEB1&!TGFB&SOX2&!p53 | E2F&!Bcatenin&!YAP1&!HNF1A&HNF4A&!NFkB&!SNAI1&ZEB1&TGFB | E2F&!Bcatenin&!YAP1&!HNF1A&HNF4A&!NFkB&SNAI1&!SNAI2&!TGFB&SOX2&!p53 | E2F&!Bcatenin&!YAP1&!HNF1A&HNF4A&!NFkB&SNAI1&!SNAI2&TGFB | E2F&!Bcatenin&!YAP1&!HNF1A&HNF4A&!NFkB&SNAI1&SNAI2&!TGFB&SOX2&!p53 | E2F&!Bcatenin&!YAP1&!HNF1A&HNF4A&!NFkB&SNAI1&SNAI2&TGFB&!NANOG&!SOX2 | E2F&!Bcatenin&!YAP1&!HNF1A&HNF4A&!NFkB&SNAI1&SNAI2&TGFB&!NANOG&SOX2&!p53 | E2F&!Bcatenin&!YAP1&!HNF1A&HNF4A&!NFkB&SNAI1&SNAI2&TGFB&NANOG | E2F&!Bcatenin&!YAP1&!HNF1A&HNF4A&NFkB&!SNAI1&!ZEB1&!TGFB&!OCT4&NANOG&!p53 | E2F&!Bcatenin&!YAP1&!HNF1A&HNF4A&NFkB&!SNAI1&!ZEB1&!TGFB&OCT4&!p53 | E2F&!Bcatenin&!YAP1&!HNF1A&HNF4A&NFkB&!SNAI1&!ZEB1&TGFB&!p53 | E2F&!Bcatenin&!YAP1&!HNF1A&HNF4A&NFkB&!SNAI1&ZEB1 | E2F&!Bcatenin&!YAP1&!HNF1A&HNF4A&NFkB&SNAI1 | E2F&!Bcatenin&!YAP1&HNF1A&!HNF4A&!NFkB&!SNAI1&!ZEB1&!TGFB&SOX2&!p53 | E2F&!Bcatenin&!YAP1&HNF1A&!HNF4A&!NFkB&!SNAI1&!ZEB1&TGFB&!p53 | E2F&!Bcatenin&!YAP1&HNF1A&!HNF4A&!NFkB&!SNAI1&ZEB1&!TGFB&SOX2&!p53 | E2F&!Bcatenin&!YAP1&HNF1A&!HNF4A&!NFkB&!SNAI1&ZEB1&TGFB | E2F&!Bcatenin&!YAP1&HNF1A&!HNF4A&!NFkB&SNAI1&!SNAI2&!TGFB&SOX2&!p53 | E2F&!Bcatenin&!YAP1&HNF1A&!HNF4A&!NFkB&SNAI1&!SNAI2&TGFB | E2F&!Bcatenin&!YAP1&HNF1A&!HNF4A&!NFkB&SNAI1&SNAI2&!TGFB&SOX2&!p53 | E2F&!Bcatenin&!YAP1&HNF1A&!HNF4A&!NFkB&SNAI1&SNAI2&TGFB&!NANOG&!SOX2 | E2F&!Bcatenin&!YAP1&HNF1A&!HNF4A&!NFkB&SNAI1&SNAI2&TGFB&!NANOG&SOX2&!p53 | E2F&!Bcatenin&!YAP1&HNF1A&!HNF4A&!NFkB&SNAI1&SNAI2&TGFB&NANOG | E2F&!Bcatenin&!YAP1&HNF1A&!HNF4A&NFkB&!SNAI1&!ZEB1&!p53 | E2F&!Bcatenin&!YAP1&HNF1A&!HNF4A&NFkB&!SNAI1&ZEB1 | E2F&!Bcatenin&!YAP1&HNF1A&!HNF4A&NFkB&SNAI1&!SNAI2 | E2F&!Bcatenin&!YAP1&HNF1A&!HNF4A&NFkB&SNAI1&SNAI2&!NANOG&!SOX2 | E2F&!Bcatenin&!YAP1&HNF1A&!HNF4A&NFkB&SNAI1&SNAI2&!NANOG&SOX2&!p53 | E2F&!Bcatenin&!YAP1&HNF1A&!HNF4A&NFkB&SNAI1&SNAI2&NANOG | E2F&!Bcatenin&!YAP1&HNF1A&HNF4A&NFkB&TGFB&!p53 | E2F&!Bcatenin&YAP1&!HNF1A&!HNF4A&!NFkB&!SNAI1&!ZEB1&!OCT4&!NANOG&!p53 | E2F&!Bcatenin&YAP1&!HNF1A&!HNF4A&!NFkB&!SNAI1&!ZEB1&!OCT4&NANOG | E2F&!Bcatenin&YAP1&!HNF1A&!HNF4A&!NFkB&!SNAI1&!ZEB1&OCT4 | E2F&!Bcatenin&YAP1&!HNF1A&!HNF4A&!NFkB&!SNAI1&ZEB1 | E2F&!Bcatenin&YAP1&!HNF1A&!HNF4A&!NFkB&SNAI1&!SNAI2 | E2F&!Bcatenin&YAP1&!HNF1A&!HNF4A&!NFkB&SNAI1&SNAI2&!OCT4&!NANOG&!SOX2 | E2F&!Bcatenin&YAP1&!HNF1A&!HNF4A&!NFkB&SNAI1&SNAI2&!OCT4&!NANOG&SOX2&!p53 | E2F&!Bcatenin&YAP1&!HNF1A&!HNF4A&!NFkB&SNAI1&SNAI2&!OCT4&NANOG | E2F&!Bcatenin&YAP1&!HNF1A&!HNF4A&!NFkB&SNAI1&SNAI2&OCT4 | E2F&!Bcatenin&YAP1&!HNF1A&!HNF4A&NFkB | E2F&!Bcatenin&YAP1&!HNF1A&HNF4A&!NFkB&!SNAI1&!ZEB1&!OCT4&NANOG | E2F&!Bcatenin&YAP1&!HNF1A&HNF4A&!NFkB&!SNAI1&!ZEB1&OCT4 | E2F&!Bcatenin&YAP1&!HNF1A&HNF4A&!NFkB&!SNAI1&ZEB1 | E2F&!Bcatenin&YAP1&!HNF1A&HNF4A&!NFkB&SNAI1&!SNAI2 | E2F&!Bcatenin&YAP1&!HNF1A&HNF4A&!NFkB&SNAI1&SNAI2&!OCT4&!NANOG&!SOX2 | E2F&!Bcatenin&YAP1&!HNF1A&HNF4A&!NFkB&SNAI1&SNAI2&!OCT4&!NANOG&SOX2&!p53 | E2F&!Bcatenin&YAP1&!HNF1A&HNF4A&!NFkB&SNAI1&SNAI2&!OCT4&NANOG | E2F&!Bcatenin&YAP1&!HNF1A&HNF4A&!NFkB&SNAI1&SNAI2&OCT4 | E2F&!Bcatenin&YAP1&!HNF1A&HNF4A&NFkB&!SNAI1&!ZEB1&!TGFB&!OCT4&NANOG | E2F&!Bcatenin&YAP1&!HNF1A&HNF4A&NFkB&!SNAI1&!ZEB1&!TGFB&OCT4 | E2F&!Bcatenin&YAP1&!HNF1A&HNF4A&NFkB&!SNAI1&!ZEB1&TGFB | E2F&!Bcatenin&YAP1&!HNF1A&HNF4A&NFkB&!SNAI1&ZEB1 | E2F&!Bcatenin&YAP1&!HNF1A&HNF4A&NFkB&SNAI1 | E2F&!Bcatenin&YAP1&HNF1A&!HNF4A&!NFkB&!SNAI1&!ZEB1&!OCT4&!NANOG&!p53 | E2F&!Bcatenin&YAP1&HNF1A&!HNF4A&!NFkB&!SNAI1&!ZEB1&!OCT4&NANOG | E2F&!Bcatenin&YAP1&HNF1A&!HNF4A&!NFkB&!SNAI1&!ZEB1&OCT4 | E2F&!Bcatenin&YAP1&HNF1A&!HNF4A&!NFkB&!SNAI1&ZEB1 | E2F&!Bcatenin&YAP1&HNF1A&!HNF4A&!NFkB&SNAI1&!SNAI2 | E2F&!Bcatenin&YAP1&HNF1A&!HNF4A&!NFkB&SNAI1&SNAI2&!OCT4&!NANOG&!SOX2 | E2F&!Bcatenin&YAP1&HNF1A&!HNF4A&!NFkB&SNAI1&SNAI2&!OCT4&!NANOG&SOX2&!p53 | E2F&!Bcatenin&YAP1&HNF1A&!HNF4A&!NFkB&SNAI1&SNAI2&!OCT4&NANOG | E2F&!Bcatenin&YAP1&HNF1A&!HNF4A&!NFkB&SNAI1&SNAI2&OCT4 | E2F&!Bcatenin&YAP1&HNF1A&!HNF4A&NFkB&!SNAI1&!ZEB1&!TGFB&!OCT4&!NANOG&!p53 | E2F&!Bcatenin&YAP1&HNF1A&!HNF4A&NFkB&!SNAI1&!ZEB1&!TGFB&!OCT4&NANOG | E2F&!Bcatenin&YAP1&HNF1A&!HNF4A&NFkB&!SNAI1&!ZEB1&!TGFB&OCT4 | E2F&!Bcatenin&YAP1&HNF1A&!HNF4A&NFkB&!SNAI1&!ZEB1&TGFB | E2F&!Bcatenin&YAP1&HNF1A&!HNF4A&NFkB&!SNAI1&ZEB1 | E2F&!Bcatenin&YAP1&HNF1A&!HNF4A&NFkB&SNAI1&!SNAI2 | E2F&!Bcatenin&YAP1&HNF1A&!HNF4A&NFkB&SNAI1&SNAI2&!TGFB&!OCT4&!NANOG&!SOX2 | E2F&!Bcatenin&YAP1&HNF1A&!HNF4A&NFkB&SNAI1&SNAI2&!TGFB&!OCT4&!NANOG&SOX2&!p53 | E2F&!Bcatenin&YAP1&HNF1A&!HNF4A&NFkB&SNAI1&SNAI2&!TGFB&!OCT4&NANOG | E2F&!Bcatenin&YAP1&HNF1A&!HNF4A&NFkB&SNAI1&SNAI2&!TGFB&OCT4 | E2F&!Bcatenin&YAP1&HNF1A&!HNF4A&NFkB&SNAI1&SNAI2&TGFB | E2F&!Bcatenin&YAP1&HNF1A&HNF4A&NFkB&TGFB | E2F&Bcatenin&!YAP1&!HNF1A&!HNF4A&!NFkB&!SNAI1&!ZEB1&!p53 | E2F&Bcatenin&!YAP1&!HNF1A&!HNF4A&!NFkB&!SNAI1&ZEB1&!TGFB&!p53 | E2F&Bcatenin&!YAP1&!HNF1A&!HNF4A&!NFkB&!SNAI1&ZEB1&TGFB | E2F&Bcatenin&!YAP1&!HNF1A&!HNF4A&!NFkB&SNAI1&!SNAI2&!TGFB&!p53 | E2F&Bcatenin&!YAP1&!HNF1A&!HNF4A&!NFkB&SNAI1&!SNAI2&TGFB | E2F&Bcatenin&!YAP1&!HNF1A&!HNF4A&!NFkB&SNAI1&SNAI2&!TGFB&!p53 | E2F&Bcatenin&!YAP1&!HNF1A&!HNF4A&!NFkB&SNAI1&SNAI2&TGFB&!NANOG&!SOX2 | E2F&Bcatenin&!YAP1&!HNF1A&!HNF4A&!NFkB&SNAI1&SNAI2&TGFB&!NANOG&SOX2&!p53 | E2F&Bcatenin&!YAP1&!HNF1A&!HNF4A&!NFkB&SNAI1&SNAI2&TGFB&NANOG | E2F&Bcatenin&!YAP1&!HNF1A&!HNF4A&NFkB | E2F&Bcatenin&!YAP1&!HNF1A&HNF4A&!NFkB&!SNAI1&!ZEB1&!TGFB&!OCT4&NANOG&!p53 | E2F&Bcatenin&!YAP1&!HNF1A&HNF4A&!NFkB&!SNAI1&!ZEB1&!TGFB&OCT4&!p53 | E2F&Bcatenin&!YAP1&!HNF1A&HNF4A&!NFkB&!SNAI1&ZEB1&!TGFB&!p53 | E2F&Bcatenin&!YAP1&!HNF1A&HNF4A&!NFkB&!SNAI1&ZEB1&TGFB | E2F&Bcatenin&!YAP1&!HNF1A&HNF4A&!NFkB&SNAI1&!SNAI2&!TGFB&!p53 | E2F&Bcatenin&!YAP1&!HNF1A&HNF4A&!NFkB&SNAI1&!SNAI2&TGFB | E2F&Bcatenin&!YAP1&!HNF1A&HNF4A&!NFkB&SNAI1&SNAI2&!TGFB&!p53 | E2F&Bcatenin&!YAP1&!HNF1A&HNF4A&!NFkB&SNAI1&SNAI2&TGFB&!NANOG&!SOX2 | E2F&Bcatenin&!YAP1&!HNF1A&HNF4A&!NFkB&SNAI1&SNAI2&TGFB&!NANOG&SOX2&!p53 | E2F&Bcatenin&!YAP1&!HNF1A&HNF4A&!NFkB&SNAI1&SNAI2&TGFB&NANOG | E2F&Bcatenin&!YAP1&!HNF1A&HNF4A&NFkB&!SNAI1&!ZEB1&!TGFB&!OCT4&NANOG&!p53 | E2F&Bcatenin&!YAP1&!HNF1A&HNF4A&NFkB&!SNAI1&!ZEB1&!TGFB&OCT4&!p53 | E2F&Bcatenin&!YAP1&!HNF1A&HNF4A&NFkB&!SNAI1&!ZEB1&TGFB&!p53 | E2F&Bcatenin&!YAP1&!HNF1A&HNF4A&NFkB&!SNAI1&ZEB1 | E2F&Bcatenin&!YAP1&!HNF1A&HNF4A&NFkB&SNAI1 | E2F&Bcatenin&!YAP1&HNF1A&!HNF4A&!NFkB&!SNAI1&!ZEB1&!p53 | E2F&Bcatenin&!YAP1&HNF1A&!HNF4A&!NFkB&!SNAI1&ZEB1&!TGFB&!p53 | E2F&Bcatenin&!YAP1&HNF1A&!HNF4A&!NFkB&!SNAI1&ZEB1&TGFB | E2F&Bcatenin&!YAP1&HNF1A&!HNF4A&!NFkB&SNAI1&!SNAI2&!TGFB&!p53 | E2F&Bcatenin&!YAP1&HNF1A&!HNF4A&!NFkB&SNAI1&!SNAI2&TGFB | E2F&Bcatenin&!YAP1&HNF1A&!HNF4A&!NFkB&SNAI1&SNAI2&!TGFB&!p53 | E2F&Bcatenin&!YAP1&HNF1A&!HNF4A&!NFkB&SNAI1&SNAI2&TGFB&!NANOG&!SOX2 | E2F&Bcatenin&!YAP1&HNF1A&!HNF4A&!NFkB&SNAI1&SNAI2&TGFB&!NANOG&SOX2&!p53 | E2F&Bcatenin&!YAP1&HNF1A&!HNF4A&!NFkB&SNAI1&SNAI2&TGFB&NANOG | E2F&Bcatenin&!YAP1&HNF1A&!HNF4A&NFkB&!SNAI1&!ZEB1&!p53 | E2F&Bcatenin&!YAP1&HNF1A&!HNF4A&NFkB&!SNAI1&ZEB1 | E2F&Bcatenin&!YAP1&HNF1A&!HNF4A&NFkB&SNAI1&!SNAI2 | E2F&Bcatenin&!YAP1&HNF1A&!HNF4A&NFkB&SNAI1&SNAI2&!NANOG&!SOX2 | E2F&Bcatenin&!YAP1&HNF1A&!HNF4A&NFkB&SNAI1&SNAI2&!NANOG&SOX2&!p53 | E2F&Bcatenin&!YAP1&HNF1A&!HNF4A&NFkB&SNAI1&SNAI2&NANOG | E2F&Bcatenin&!YAP1&HNF1A&HNF4A&NFkB&TGFB&!p53 | E2F&Bcatenin&YAP1&!HNF1A&!HNF4A&!NFkB&!SNAI1&!ZEB1&!OCT4&!NANOG&!p53 | E2F&Bcatenin&YAP1&!HNF1A&!HNF4A&!NFkB&!SNAI1&!ZEB1&!OCT4&NANOG | E2F&Bcatenin&YAP1&!HNF1A&!HNF4A&!NFkB&!SNAI1&!ZEB1&OCT4 | E2F&Bcatenin&YAP1&!HNF1A&!HNF4A&!NFkB&!SNAI1&ZEB1 | E2F&Bcatenin&YAP1&!HNF1A&!HNF4A&!NFkB&SNAI1&!SNAI2 | E2F&Bcatenin&YAP1&!HNF1A&!HNF4A&!NFkB&SNAI1&SNAI2&!OCT4&!NANOG&!SOX2 | E2F&Bcatenin&YAP1&!HNF1A&!HNF4A&!NFkB&SNAI1&SNAI2&!OCT4&!NANOG&SOX2&!p53 | E2F&Bcatenin&YAP1&!HNF1A&!HNF4A&!NFkB&SNAI1&SNAI2&!OCT4&NANOG | E2F&Bcatenin&YAP1&!HNF1A&!HNF4A&!NFkB&SNAI1&SNAI2&OCT4 | E2F&Bcatenin&YAP1&!HNF1A&!HNF4A&NFkB | E2F&Bcatenin&YAP1&!HNF1A&HNF4A&!NFkB&!SNAI1&!ZEB1&!OCT4&NANOG | E2F&Bcatenin&YAP1&!HNF1A&HNF4A&!NFkB&!SNAI1&!ZEB1&OCT4 | E2F&Bcatenin&YAP1&!HNF1A&HNF4A&!NFkB&!SNAI1&ZEB1 | E2F&Bcatenin&YAP1&!HNF1A&HNF4A&!NFkB&SNAI1&!SNAI2 | E2F&Bcatenin&YAP1&!HNF1A&HNF4A&!NFkB&SNAI1&SNAI2&!OCT4&!NANOG&!SOX2 | E2F&Bcatenin&YAP1&!HNF1A&HNF4A&!NFkB&SNAI1&SNAI2&!OCT4&!NANOG&SOX2&!p53 | E2F&Bcatenin&YAP1&!HNF1A&HNF4A&!NFkB&SNAI1&SNAI2&!OCT4&NANOG | E2F&Bcatenin&YAP1&!HNF1A&HNF4A&!NFkB&SNAI1&SNAI2&OCT4 | E2F&Bcatenin&YAP1&!HNF1A&HNF4A&NFkB&!SNAI1&!ZEB1&!TGFB&!OCT4&NANOG | E2F&Bcatenin&YAP1&!HNF1A&HNF4A&NFkB&!SNAI1&!ZEB1&!TGFB&OCT4 | E2F&Bcatenin&YAP1&!HNF1A&HNF4A&NFkB&!SNAI1&!ZEB1&TGFB | E2F&Bcatenin&YAP1&!HNF1A&HNF4A&NFkB&!SNAI1&ZEB1 | E2F&Bcatenin&YAP1&!HNF1A&HNF4A&NFkB&SNAI1 | E2F&Bcatenin&YAP1&HNF1A&!HNF4A&!NFkB&!SNAI1&!ZEB1&!OCT4&!NANOG&!p53 | E2F&Bcatenin&YAP1&HNF1A&!HNF4A&!NFkB&!SNAI1&!ZEB1&!OCT4&NANOG | E2F&Bcatenin&YAP1&HNF1A&!HNF4A&!NFkB&!SNAI1&!ZEB1&OCT4 | E2F&Bcatenin&YAP1&HNF1A&!HNF4A&!NFkB&!SNAI1&ZEB1 | E2F&Bcatenin&YAP1&HNF1A&!HNF4A&!NFkB&SNAI1&!SNAI2 | E2F&Bcatenin&YAP1&HNF1A&!HNF4A&!NFkB&SNAI1&SNAI2&!OCT4&!NANOG&!SOX2 | E2F&Bcatenin&YAP1&HNF1A&!HNF4A&!NFkB&SNAI1&SNAI2&!OCT4&!NANOG&SOX2&!p53 | E2F&Bcatenin&YAP1&HNF1A&!HNF4A&!NFkB&SNAI1&SNAI2&!OCT4&NANOG | E2F&Bcatenin&YAP1&HNF1A&!HNF4A&!NFkB&SNAI1&SNAI2&OCT4 | E2F&Bcatenin&YAP1&HNF1A&!HNF4A&NFkB&!SNAI1&!ZEB1&!TGFB&!OCT4&!NANOG&!p53 | E2F&Bcatenin&YAP1&HNF1A&!HNF4A&NFkB&!SNAI1&!ZEB1&!TGFB&!OCT4&NANOG | E2F&Bcatenin&YAP1&HNF1A&!HNF4A&NFkB&!SNAI1&!ZEB1&!TGFB&OCT4 | E2F&Bcatenin&YAP1&HNF1A&!HNF4A&NFkB&!SNAI1&!ZEB1&TGFB | E2F&Bcatenin&YAP1&HNF1A&!HNF4A&NFkB&!SNAI1&ZEB1 | E2F&Bcatenin&YAP1&HNF1A&!HNF4A&NFkB&SNAI1&!SNAI2 | E2F&Bcatenin&YAP1&HNF1A&!HNF4A&NFkB&SNAI1&SNAI2&!TGFB&!OCT4&!NANOG&!SOX2 | E2F&Bcatenin&YAP1&HNF1A&!HNF4A&NFkB&SNAI1&SNAI2&!TGFB&!OCT4&!NANOG&SOX2&!p53 | E2F&Bcatenin&YAP1&HNF1A&!HNF4A&NFkB&SNAI1&SNAI2&!TGFB&!OCT4&NANOG | E2F&Bcatenin&YAP1&HNF1A&!HNF4A&NFkB&SNAI1&SNAI2&!TGFB&OCT4 | E2F&Bcatenin&YAP1&HNF1A&!HNF4A&NFkB&SNAI1&SNAI2&TGFB | E2F&Bcatenin&YAP1&HNF1A&HNF4A&NFkB&TGFB

SNAI2, !BMI1&!Bcatenin&!YAP1&!HNF1A&!HNF4A&!NFkB&!SNAI1&!ZEB1&!SNAI2&SOX9&!OCT4&!SOX2 | !BMI1&!Bcatenin&!YAP1&!HNF1A&!HNF4A&!NFkB&!SNAI1&!ZEB1&SNAI2&!OCT4 | !BMI1&!Bcatenin&!YAP1&!HNF1A&!HNF4A&!NFkB&!SNAI1&ZEB1&!SNAI2&SOX9&!OCT4 | !BMI1&!Bcatenin&!YAP1&!HNF1A&!HNF4A&!NFkB&!SNAI1&ZEB1&!SNAI2&SOX9&OCT4&!p53 | !BMI1&!Bcatenin&!YAP1&!HNF1A&!HNF4A&!NFkB&!SNAI1&ZEB1&SNAI2&!OCT4 | !BMI1&!Bcatenin&!YAP1&!HNF1A&!HNF4A&!NFkB&!SNAI1&ZEB1&SNAI2&OCT4&!p53 | !BMI1&!Bcatenin&!YAP1&!HNF1A&!HNF4A&!NFkB&SNAI1&!ZEB1&!SNAI2&!OCT4&!SOX2 | !BMI1&!Bcatenin&!YAP1&!HNF1A&!HNF4A&!NFkB&SNAI1&!ZEB1&!SNAI2&OCT4&!SOX2&!p53 | !BMI1&!Bcatenin&!YAP1&!HNF1A&!HNF4A&!NFkB&SNAI1&!ZEB1&SNAI2&!OCT4 | !BMI1&!Bcatenin&!YAP1&!HNF1A&!HNF4A&!NFkB&SNAI1&!ZEB1&SNAI2&OCT4&!p53 | !BMI1&!Bcatenin&!YAP1&!HNF1A&!HNF4A&!NFkB&SNAI1&ZEB1 | !BMI1&!Bcatenin&!YAP1&!HNF1A&!HNF4A&NFkB&!SNAI1&!ZEB1&!SNAI2&!OCT4&!SOX2 | !BMI1&!Bcatenin&!YAP1&!HNF1A&!HNF4A&NFkB&!SNAI1&!ZEB1&SNAI2&!OCT4 | !BMI1&!Bcatenin&!YAP1&!HNF1A&!HNF4A&NFkB&!SNAI1&ZEB1&!OCT4 | !BMI1&!Bcatenin&!YAP1&!HNF1A&!HNF4A&NFkB&!SNAI1&ZEB1&OCT4&!p53 | !BMI1&!Bcatenin&!YAP1&!HNF1A&!HNF4A&NFkB&SNAI1&!ZEB1&!SNAI2&!OCT4&!SOX2 | !BMI1&!Bcatenin&!YAP1&!HNF1A&!HNF4A&NFkB&SNAI1&!ZEB1&!SNAI2&OCT4&!SOX2&!p53 | !BMI1&!Bcatenin&!YAP1&!HNF1A&!HNF4A&NFkB&SNAI1&!ZEB1&SNAI2&!OCT4 | !BMI1&!Bcatenin&!YAP1&!HNF1A&!HNF4A&NFkB&SNAI1&!ZEB1&SNAI2&OCT4&!p53 | !BMI1&!Bcatenin&!YAP1&!HNF1A&!HNF4A&NFkB&SNAI1&ZEB1 | !BMI1&!Bcatenin&!YAP1&HNF1A&!HNF4A&!FOXA2&!NFkB&!SNAI1&!ZEB1&!SNAI2&SOX9&!OCT4&!SOX2 | !BMI1&!Bcatenin&!YAP1&HNF1A&!HNF4A&!FOXA2&!NFkB&!SNAI1&!ZEB1&SNAI2&!OCT4 | !BMI1&!Bcatenin&!YAP1&HNF1A&!HNF4A&!FOXA2&!NFkB&!SNAI1&ZEB1&!SNAI2&SOX9&!OCT4 | !BMI1&!Bcatenin&!YAP1&HNF1A&!HNF4A&!FOXA2&!NFkB&!SNAI1&ZEB1&!SNAI2&SOX9&OCT4&!p53 | !BMI1&!Bcatenin&!YAP1&HNF1A&!HNF4A&!FOXA2&!NFkB&!SNAI1&ZEB1&SNAI2&!OCT4 | !BMI1&!Bcatenin&!YAP1&HNF1A&!HNF4A&!FOXA2&!NFkB&!SNAI1&ZEB1&SNAI2&OCT4&!p53 | !BMI1&!Bcatenin&!YAP1&HNF1A&!HNF4A&!FOXA2&!NFkB&SNAI1&!ZEB1&!SNAI2&!OCT4&!SOX2 | !BMI1&!Bcatenin&!YAP1&HNF1A&!HNF4A&!FOXA2&!NFkB&SNAI1&!ZEB1&!SNAI2&OCT4&!SOX2&!p53 | !BMI1&!Bcatenin&!YAP1&HNF1A&!HNF4A&!FOXA2&!NFkB&SNAI1&!ZEB1&SNAI2&!OCT4 | !BMI1&!Bcatenin&!YAP1&HNF1A&!HNF4A&!FOXA2&!NFkB&SNAI1&!ZEB1&SNAI2&OCT4&!p53 | !BMI1&!Bcatenin&!YAP1&HNF1A&!HNF4A&!FOXA2&!NFkB&SNAI1&ZEB1 | !BMI1&!Bcatenin&!YAP1&HNF1A&!HNF4A&!FOXA2&NFkB&!SNAI1&!ZEB1&!SNAI2&!OCT4&!SOX2 | !BMI1&!Bcatenin&!YAP1&HNF1A&!HNF4A&!FOXA2&NFkB&!SNAI1&!ZEB1&SNAI2&!OCT4 | !BMI1&!Bcatenin&!YAP1&HNF1A&!HNF4A&!FOXA2&NFkB&!SNAI1&ZEB1&!OCT4 | !BMI1&!Bcatenin&!YAP1&HNF1A&!HNF4A&!FOXA2&NFkB&!SNAI1&ZEB1&OCT4&!p53 | !BMI1&!Bcatenin&!YAP1&HNF1A&!HNF4A&!FOXA2&NFkB&SNAI1&!ZEB1&!SNAI2&!OCT4&!SOX2 | !BMI1&!Bcatenin&!YAP1&HNF1A&!HNF4A&!FOXA2&NFkB&SNAI1&!ZEB1&!SNAI2&OCT4&!SOX2&!p53 | !BMI1&!Bcatenin&!YAP1&HNF1A&!HNF4A&!FOXA2&NFkB&SNAI1&!ZEB1&SNAI2&!OCT4 | !BMI1&!Bcatenin&!YAP1&HNF1A&!HNF4A&!FOXA2&NFkB&SNAI1&!ZEB1&SNAI2&OCT4&!p53 | !BMI1&!Bcatenin&!YAP1&HNF1A&!HNF4A&!FOXA2&NFkB&SNAI1&ZEB1 | !BMI1&!Bcatenin&YAP1&!HNF1A&!HNF4A&!NFkB&!SNAI1&!ZEB1&!SNAI2&SOX9&!OCT4&!SOX2 | !BMI1&!Bcatenin&YAP1&!HNF1A&!HNF4A&!NFkB&!SNAI1&!ZEB1&SNAI2&!OCT4 | !BMI1&!Bcatenin&YAP1&!HNF1A&!HNF4A&!NFkB&!SNAI1&ZEB1&!SNAI2&SOX9&!OCT4 | !BMI1&!Bcatenin&YAP1&!HNF1A&!HNF4A&!NFkB&!SNAI1&ZEB1&!SNAI2&SOX9&OCT4&!p53 | !BMI1&!Bcatenin&YAP1&!HNF1A&!HNF4A&!NFkB&!SNAI1&ZEB1&SNAI2&!OCT4 | !BMI1&!Bcatenin&YAP1&!HNF1A&!HNF4A&!NFkB&!SNAI1&ZEB1&SNAI2&OCT4&!p53 | !BMI1&!Bcatenin&YAP1&!HNF1A&!HNF4A&!NFkB&SNAI1&!ZEB1&!SNAI2&!OCT4&!SOX2 | !BMI1&!Bcatenin&YAP1&!HNF1A&!HNF4A&!NFkB&SNAI1&!ZEB1&!SNAI2&OCT4&!SOX2&!p53 | !BMI1&!Bcatenin&YAP1&!HNF1A&!HNF4A&!NFkB&SNAI1&!ZEB1&SNAI2&!OCT4 | !BMI1&!Bcatenin&YAP1&!HNF1A&!HNF4A&!NFkB&SNAI1&!ZEB1&SNAI2&OCT4&!p53 | !BMI1&!Bcatenin&YAP1&!HNF1A&!HNF4A&!NFkB&SNAI1&ZEB1 | !BMI1&!Bcatenin&YAP1&!HNF1A&!HNF4A&NFkB&!SNAI1&!ZEB1&!SNAI2&!OCT4&!SOX2 | !BMI1&!Bcatenin&YAP1&!HNF1A&!HNF4A&NFkB&!SNAI1&!ZEB1&SNAI2&!OCT4 | !BMI1&!Bcatenin&YAP1&!HNF1A&!HNF4A&NFkB&!SNAI1&ZEB1&!OCT4 | !BMI1&!Bcatenin&YAP1&!HNF1A&!HNF4A&NFkB&!SNAI1&ZEB1&OCT4&!p53 | !BMI1&!Bcatenin&YAP1&!HNF1A&!HNF4A&NFkB&SNAI1&!ZEB1&!SNAI2&!OCT4&!SOX2 | !BMI1&!Bcatenin&YAP1&!HNF1A&!HNF4A&NFkB&SNAI1&!ZEB1&!SNAI2&OCT4&!SOX2&!p53 | !BMI1&!Bcatenin&YAP1&!HNF1A&!HNF4A&NFkB&SNAI1&!ZEB1&SNAI2&!OCT4 | !BMI1&!Bcatenin&YAP1&!HNF1A&!HNF4A&NFkB&SNAI1&!ZEB1&SNAI2&OCT4&!p53 | !BMI1&!Bcatenin&YAP1&!HNF1A&!HNF4A&NFkB&SNAI1&ZEB1 | !BMI1&!Bcatenin&YAP1&!HNF1A&HNF4A&!FOXA2&!NFkB&!SNAI1&ZEB1&SNAI2&!p53 | !BMI1&!Bcatenin&YAP1&!HNF1A&HNF4A&!FOXA2&!NFkB&SNAI1&!ZEB1&SNAI2&!p53 | !BMI1&!Bcatenin&YAP1&!HNF1A&HNF4A&!FOXA2&!NFkB&SNAI1&ZEB1&!SNAI2&NANOG | !BMI1&!Bcatenin&YAP1&!HNF1A&HNF4A&!FOXA2&!NFkB&SNAI1&ZEB1&SNAI2 | !BMI1&!Bcatenin&YAP1&!HNF1A&HNF4A&!FOXA2&NFkB&!SNAI1&ZEB1&!p53 | !BMI1&!Bcatenin&YAP1&!HNF1A&HNF4A&!FOXA2&NFkB&SNAI1&!ZEB1&SNAI2&!p53 | !BMI1&!Bcatenin&YAP1&!HNF1A&HNF4A&!FOXA2&NFkB&SNAI1&ZEB1 | !BMI1&!Bcatenin&YAP1&!HNF1A&HNF4A&FOXA2&!NFkB&!SNAI1&ZEB1&SNAI2&NANOG&!p53 | !BMI1&!Bcatenin&YAP1&!HNF1A&HNF4A&FOXA2&!NFkB&SNAI1&!ZEB1&SNAI2&NANOG&!p53 | !BMI1&!Bcatenin&YAP1&!HNF1A&HNF4A&FOXA2&!NFkB&SNAI1&ZEB1&NANOG | !BMI1&!Bcatenin&YAP1&!HNF1A&HNF4A&FOXA2&NFkB&!SNAI1&ZEB1&NANOG&!p53 | !BMI1&!Bcatenin&YAP1&!HNF1A&HNF4A&FOXA2&NFkB&SNAI1&!ZEB1&SNAI2&NANOG&!p53 | !BMI1&!Bcatenin&YAP1&!HNF1A&HNF4A&FOXA2&NFkB&SNAI1&ZEB1&NANOG | !BMI1&!Bcatenin&YAP1&HNF1A&!HNF4A&!FOXA2&!NFkB&!SNAI1&!ZEB1&!SNAI2&SOX9&!OCT4&!SOX2 | !BMI1&!Bcatenin&YAP1&HNF1A&!HNF4A&!FOXA2&!NFkB&!SNAI1&!ZEB1&SNAI2&!OCT4 | !BMI1&!Bcatenin&YAP1&HNF1A&!HNF4A&!FOXA2&!NFkB&!SNAI1&ZEB1&!SNAI2&SOX9&!OCT4 | !BMI1&!Bcatenin&YAP1&HNF1A&!HNF4A&!FOXA2&!NFkB&!SNAI1&ZEB1&!SNAI2&SOX9&OCT4&!p53 | !BMI1&!Bcatenin&YAP1&HNF1A&!HNF4A&!FOXA2&!NFkB&!SNAI1&ZEB1&SNAI2&!OCT4 | !BMI1&!Bcatenin&YAP1&HNF1A&!HNF4A&!FOXA2&!NFkB&!SNAI1&ZEB1&SNAI2&OCT4&!p53 | !BMI1&!Bcatenin&YAP1&HNF1A&!HNF4A&!FOXA2&!NFkB&SNAI1&!ZEB1&!SNAI2&!OCT4&!SOX2 | !BMI1&!Bcatenin&YAP1&HNF1A&!HNF4A&!FOXA2&!NFkB&SNAI1&!ZEB1&!SNAI2&OCT4&!SOX2&!p53 | !BMI1&!Bcatenin&YAP1&HNF1A&!HNF4A&!FOXA2&!NFkB&SNAI1&!ZEB1&SNAI2&!OCT4 | !BMI1&!Bcatenin&YAP1&HNF1A&!HNF4A&!FOXA2&!NFkB&SNAI1&!ZEB1&SNAI2&OCT4&!p53 | !BMI1&!Bcatenin&YAP1&HNF1A&!HNF4A&!FOXA2&!NFkB&SNAI1&ZEB1 | !BMI1&!Bcatenin&YAP1&HNF1A&!HNF4A&!FOXA2&NFkB&!SNAI1&!ZEB1&!SNAI2&!OCT4&!SOX2 | !BMI1&!Bcatenin&YAP1&HNF1A&!HNF4A&!FOXA2&NFkB&!SNAI1&!ZEB1&SNAI2&!OCT4 | !BMI1&!Bcatenin&YAP1&HNF1A&!HNF4A&!FOXA2&NFkB&!SNAI1&ZEB1&!OCT4 | !BMI1&!Bcatenin&YAP1&HNF1A&!HNF4A&!FOXA2&NFkB&!SNAI1&ZEB1&OCT4&!p53 | !BMI1&!Bcatenin&YAP1&HNF1A&!HNF4A&!FOXA2&NFkB&SNAI1&!ZEB1&!SNAI2&!OCT4&!SOX2 | !BMI1&!Bcatenin&YAP1&HNF1A&!HNF4A&!FOXA2&NFkB&SNAI1&!ZEB1&!SNAI2&OCT4&!SOX2&!p53 | !BMI1&!Bcatenin&YAP1&HNF1A&!HNF4A&!FOXA2&NFkB&SNAI1&!ZEB1&SNAI2&!OCT4 | !BMI1&!Bcatenin&YAP1&HNF1A&!HNF4A&!FOXA2&NFkB&SNAI1&!ZEB1&SNAI2&OCT4&!p53 | !BMI1&!Bcatenin&YAP1&HNF1A&!HNF4A&!FOXA2&NFkB&SNAI1&ZEB1 | !BMI1&!Bcatenin&YAP1&HNF1A&!HNF4A&FOXA2&!NFkB&!SNAI1&!ZEB1&SNAI2&!OCT4&NANOG | !BMI1&!Bcatenin&YAP1&HNF1A&!HNF4A&FOXA2&!NFkB&!SNAI1&ZEB1&SNAI2&!OCT4&NANOG | !BMI1&!Bcatenin&YAP1&HNF1A&!HNF4A&FOXA2&!NFkB&!SNAI1&ZEB1&SNAI2&OCT4&NANOG&!p53 | !BMI1&!Bcatenin&YAP1&HNF1A&!HNF4A&FOXA2&!NFkB&SNAI1&!ZEB1&!SNAI2&!OCT4&NANOG&!SOX2 | !BMI1&!Bcatenin&YAP1&HNF1A&!HNF4A&FOXA2&!NFkB&SNAI1&!ZEB1&!SNAI2&OCT4&NANOG&!SOX2&!p53 | !BMI1&!Bcatenin&YAP1&HNF1A&!HNF4A&FOXA2&!NFkB&SNAI1&!ZEB1&SNAI2&!OCT4&NANOG | !BMI1&!Bcatenin&YAP1&HNF1A&!HNF4A&FOXA2&!NFkB&SNAI1&!ZEB1&SNAI2&OCT4&NANOG&!p53 | !BMI1&!Bcatenin&YAP1&HNF1A&!HNF4A&FOXA2&!NFkB&SNAI1&ZEB1&NANOG | !BMI1&!Bcatenin&YAP1&HNF1A&!HNF4A&FOXA2&NFkB&!SNAI1&!ZEB1&!SNAI2&!OCT4&NANOG&!SOX2&!p53 | !BMI1&!Bcatenin&YAP1&HNF1A&!HNF4A&FOXA2&NFkB&!SNAI1&!ZEB1&SNAI2&!OCT4&NANOG | !BMI1&!Bcatenin&YAP1&HNF1A&!HNF4A&FOXA2&NFkB&!SNAI1&ZEB1&!OCT4&NANOG | !BMI1&!Bcatenin&YAP1&HNF1A&!HNF4A&FOXA2&NFkB&!SNAI1&ZEB1&OCT4&NANOG&!p53 | !BMI1&!Bcatenin&YAP1&HNF1A&!HNF4A&FOXA2&NFkB&SNAI1&!ZEB1&!SNAI2&!OCT4&NANOG&!SOX2 | !BMI1&!Bcatenin&YAP1&HNF1A&!HNF4A&FOXA2&NFkB&SNAI1&!ZEB1&!SNAI2&OCT4&NANOG&!SOX2&!p53 | !BMI1&!Bcatenin&YAP1&HNF1A&!HNF4A&FOXA2&NFkB&SNAI1&!ZEB1&SNAI2&!OCT4&NANOG | !BMI1&!Bcatenin&YAP1&HNF1A&!HNF4A&FOXA2&NFkB&SNAI1&!ZEB1&SNAI2&OCT4&NANOG&!p53 | !BMI1&!Bcatenin&YAP1&HNF1A&!HNF4A&FOXA2&NFkB&SNAI1&ZEB1&NANOG | !BMI1&Bcatenin&!HNF1A&!HNF4A&!NFkB&!SNAI1&!ZEB1&!SNAI2&SOX9&!OCT4&!SOX2 | !BMI1&Bcatenin&!HNF1A&!HNF4A&!NFkB&!SNAI1&!ZEB1&SNAI2&!OCT4 | !BMI1&Bcatenin&!HNF1A&!HNF4A&!NFkB&!SNAI1&ZEB1&!SNAI2&SOX9&!OCT4 | !BMI1&Bcatenin&!HNF1A&!HNF4A&!NFkB&!SNAI1&ZEB1&!SNAI2&SOX9&OCT4&!p53 | !BMI1&Bcatenin&!HNF1A&!HNF4A&!NFkB&!SNAI1&ZEB1&SNAI2&!OCT4 | !BMI1&Bcatenin&!HNF1A&!HNF4A&!NFkB&!SNAI1&ZEB1&SNAI2&OCT4&!p53 | !BMI1&Bcatenin&!HNF1A&!HNF4A&!NFkB&SNAI1&!ZEB1&!SNAI2&!OCT4&!SOX2 | !BMI1&Bcatenin&!HNF1A&!HNF4A&!NFkB&SNAI1&!ZEB1&!SNAI2&OCT4&!SOX2&!p53 | !BMI1&Bcatenin&!HNF1A&!HNF4A&!NFkB&SNAI1&!ZEB1&SNAI2&!OCT4 | !BMI1&Bcatenin&!HNF1A&!HNF4A&!NFkB&SNAI1&!ZEB1&SNAI2&OCT4&!p53 | !BMI1&Bcatenin&!HNF1A&!HNF4A&!NFkB&SNAI1&ZEB1 | !BMI1&Bcatenin&!HNF1A&!HNF4A&NFkB&!SNAI1&!ZEB1&!SNAI2&!OCT4&!SOX2 | !BMI1&Bcatenin&!HNF1A&!HNF4A&NFkB&!SNAI1&!ZEB1&SNAI2&!OCT4 | !BMI1&Bcatenin&!HNF1A&!HNF4A&NFkB&!SNAI1&ZEB1&!OCT4 | !BMI1&Bcatenin&!HNF1A&!HNF4A&NFkB&!SNAI1&ZEB1&OCT4&!p53 | !BMI1&Bcatenin&!HNF1A&!HNF4A&NFkB&SNAI1&!ZEB1&!SNAI2&!OCT4&!SOX2 | !BMI1&Bcatenin&!HNF1A&!HNF4A&NFkB&SNAI1&!ZEB1&!SNAI2&OCT4&!SOX2&!p53 | !BMI1&Bcatenin&!HNF1A&!HNF4A&NFkB&SNAI1&!ZEB1&SNAI2&!OCT4 | !BMI1&Bcatenin&!HNF1A&!HNF4A&NFkB&SNAI1&!ZEB1&SNAI2&OCT4&!p53 | !BMI1&Bcatenin&!HNF1A&!HNF4A&NFkB&SNAI1&ZEB1 | !BMI1&Bcatenin&!HNF1A&HNF4A&!FOXA2&!NFkB&!SNAI1&ZEB1&SNAI2&!p53 | !BMI1&Bcatenin&!HNF1A&HNF4A&!FOXA2&!NFkB&SNAI1&!ZEB1&SNAI2&!p53 | !BMI1&Bcatenin&!HNF1A&HNF4A&!FOXA2&!NFkB&SNAI1&ZEB1&!SNAI2&NANOG | !BMI1&Bcatenin&!HNF1A&HNF4A&!FOXA2&!NFkB&SNAI1&ZEB1&SNAI2 | !BMI1&Bcatenin&!HNF1A&HNF4A&!FOXA2&NFkB&!SNAI1&ZEB1&!p53 | !BMI1&Bcatenin&!HNF1A&HNF4A&!FOXA2&NFkB&SNAI1&!ZEB1&SNAI2&!p53 | !BMI1&Bcatenin&!HNF1A&HNF4A&!FOXA2&NFkB&SNAI1&ZEB1 | !BMI1&Bcatenin&!HNF1A&HNF4A&FOXA2&!NFkB&!SNAI1&ZEB1&SNAI2&NANOG&!p53 | !BMI1&Bcatenin&!HNF1A&HNF4A&FOXA2&!NFkB&SNAI1&!ZEB1&SNAI2&NANOG&!p53 | !BMI1&Bcatenin&!HNF1A&HNF4A&FOXA2&!NFkB&SNAI1&ZEB1&NANOG | !BMI1&Bcatenin&!HNF1A&HNF4A&FOXA2&NFkB&!SNAI1&ZEB1&NANOG&!p53 | !BMI1&Bcatenin&!HNF1A&HNF4A&FOXA2&NFkB&SNAI1&!ZEB1&SNAI2&NANOG&!p53 | !BMI1&Bcatenin&!HNF1A&HNF4A&FOXA2&NFkB&SNAI1&ZEB1&NANOG | !BMI1&Bcatenin&HNF1A&!HNF4A&!FOXA2&!NFkB&!SNAI1&!ZEB1&!SNAI2&SOX9&!OCT4&!SOX2 | !BMI1&Bcatenin&HNF1A&!HNF4A&!FOXA2&!NFkB&!SNAI1&!ZEB1&SNAI2&!OCT4 | !BMI1&Bcatenin&HNF1A&!HNF4A&!FOXA2&!NFkB&!SNAI1&ZEB1&!SNAI2&SOX9&!OCT4 | !BMI1&Bcatenin&HNF1A&!HNF4A&!FOXA2&!NFkB&!SNAI1&ZEB1&!SNAI2&SOX9&OCT4&!p53 | !BMI1&Bcatenin&HNF1A&!HNF4A&!FOXA2&!NFkB&!SNAI1&ZEB1&SNAI2&!OCT4 | !BMI1&Bcatenin&HNF1A&!HNF4A&!FOXA2&!NFkB&!SNAI1&ZEB1&SNAI2&OCT4&!p53 | !BMI1&Bcatenin&HNF1A&!HNF4A&!FOXA2&!NFkB&SNAI1&!ZEB1&!SNAI2&!OCT4&!SOX2 | !BMI1&Bcatenin&HNF1A&!HNF4A&!FOXA2&!NFkB&SNAI1&!ZEB1&!SNAI2&OCT4&!SOX2&!p53 | !BMI1&Bcatenin&HNF1A&!HNF4A&!FOXA2&!NFkB&SNAI1&!ZEB1&SNAI2&!OCT4 | !BMI1&Bcatenin&HNF1A&!HNF4A&!FOXA2&!NFkB&SNAI1&!ZEB1&SNAI2&OCT4&!p53 | !BMI1&Bcatenin&HNF1A&!HNF4A&!FOXA2&!NFkB&SNAI1&ZEB1 | !BMI1&Bcatenin&HNF1A&!HNF4A&!FOXA2&NFkB&!SNAI1&!ZEB1&!SNAI2&!OCT4&!SOX2 | !BMI1&Bcatenin&HNF1A&!HNF4A&!FOXA2&NFkB&!SNAI1&!ZEB1&SNAI2&!OCT4 | !BMI1&Bcatenin&HNF1A&!HNF4A&!FOXA2&NFkB&!SNAI1&ZEB1&!OCT4 | !BMI1&Bcatenin&HNF1A&!HNF4A&!FOXA2&NFkB&!SNAI1&ZEB1&OCT4&!p53 | !BMI1&Bcatenin&HNF1A&!HNF4A&!FOXA2&NFkB&SNAI1&!ZEB1&!SNAI2&!OCT4&!SOX2 | !BMI1&Bcatenin&HNF1A&!HNF4A&!FOXA2&NFkB&SNAI1&!ZEB1&!SNAI2&OCT4&!SOX2&!p53 | !BMI1&Bcatenin&HNF1A&!HNF4A&!FOXA2&NFkB&SNAI1&!ZEB1&SNAI2&!OCT4 | !BMI1&Bcatenin&HNF1A&!HNF4A&!FOXA2&NFkB&SNAI1&!ZEB1&SNAI2&OCT4&!p53 | !BMI1&Bcatenin&HNF1A&!HNF4A&!FOXA2&NFkB&SNAI1&ZEB1 | !BMI1&Bcatenin&HNF1A&!HNF4A&FOXA2&!NFkB&!SNAI1&!ZEB1&SNAI2&!OCT4&NANOG | !BMI1&Bcatenin&HNF1A&!HNF4A&FOXA2&!NFkB&!SNAI1&ZEB1&SNAI2&!OCT4&NANOG | !BMI1&Bcatenin&HNF1A&!HNF4A&FOXA2&!NFkB&!SNAI1&ZEB1&SNAI2&OCT4&NANOG&!p53 | !BMI1&Bcatenin&HNF1A&!HNF4A&FOXA2&!NFkB&SNAI1&!ZEB1&!SNAI2&!OCT4&NANOG&!SOX2 | !BMI1&Bcatenin&HNF1A&!HNF4A&FOXA2&!NFkB&SNAI1&!ZEB1&!SNAI2&OCT4&NANOG&!SOX2&!p53 | !BMI1&Bcatenin&HNF1A&!HNF4A&FOXA2&!NFkB&SNAI1&!ZEB1&SNAI2&!OCT4&NANOG | !BMI1&Bcatenin&HNF1A&!HNF4A&FOXA2&!NFkB&SNAI1&!ZEB1&SNAI2&OCT4&NANOG&!p53 | !BMI1&Bcatenin&HNF1A&!HNF4A&FOXA2&!NFkB&SNAI1&ZEB1&NANOG | !BMI1&Bcatenin&HNF1A&!HNF4A&FOXA2&NFkB&!SNAI1&!ZEB1&!SNAI2&!OCT4&NANOG&!SOX2&!p53 | !BMI1&Bcatenin&HNF1A&!HNF4A&FOXA2&NFkB&!SNAI1&!ZEB1&SNAI2&!OCT4&NANOG | !BMI1&Bcatenin&HNF1A&!HNF4A&FOXA2&NFkB&!SNAI1&ZEB1&!OCT4&NANOG | !BMI1&Bcatenin&HNF1A&!HNF4A&FOXA2&NFkB&!SNAI1&ZEB1&OCT4&NANOG&!p53 | !BMI1&Bcatenin&HNF1A&!HNF4A&FOXA2&NFkB&SNAI1&!ZEB1&!SNAI2&!OCT4&NANOG&!SOX2 | !BMI1&Bcatenin&HNF1A&!HNF4A&FOXA2&NFkB&SNAI1&!ZEB1&!SNAI2&OCT4&NANOG&!SOX2&!p53 | !BMI1&Bcatenin&HNF1A&!HNF4A&FOXA2&NFkB&SNAI1&!ZEB1&SNAI2&!OCT4&NANOG | !BMI1&Bcatenin&HNF1A&!HNF4A&FOXA2&NFkB&SNAI1&!ZEB1&SNAI2&OCT4&NANOG&!p53 | !BMI1&Bcatenin&HNF1A&!HNF4A&FOXA2&NFkB&SNAI1&ZEB1&NANOG | BMI1&!Bcatenin&!YAP1&!HNF1A&!HNF4A&!NFkB&!SNAI1&!ZEB1&!SNAI2&SOX9&!SOX2 | BMI1&!Bcatenin&!YAP1&!HNF1A&!HNF4A&!NFkB&!SNAI1&!ZEB1&SNAI2 | BMI1&!Bcatenin&!YAP1&!HNF1A&!HNF4A&!NFkB&!SNAI1&ZEB1&!SNAI2&SOX9 | BMI1&!Bcatenin&!YAP1&!HNF1A&!HNF4A&!NFkB&!SNAI1&ZEB1&SNAI2 | BMI1&!Bcatenin&!YAP1&!HNF1A&!HNF4A&!NFkB&SNAI1&!ZEB1&!SNAI2&!SOX2 | BMI1&!Bcatenin&!YAP1&!HNF1A&!HNF4A&!NFkB&SNAI1&!ZEB1&SNAI2 | BMI1&!Bcatenin&!YAP1&!HNF1A&!HNF4A&!NFkB&SNAI1&ZEB1 | BMI1&!Bcatenin&!YAP1&!HNF1A&!HNF4A&NFkB&!ZEB1&!SNAI2&!SOX2 | BMI1&!Bcatenin&!YAP1&!HNF1A&!HNF4A&NFkB&!ZEB1&SNAI2 | BMI1&!Bcatenin&!YAP1&!HNF1A&!HNF4A&NFkB&ZEB1 | BMI1&!Bcatenin&!YAP1&HNF1A&!HNF4A&!FOXA2&!NFkB&!SNAI1&!ZEB1&!SNAI2&SOX9&!SOX2 | BMI1&!Bcatenin&!YAP1&HNF1A&!HNF4A&!FOXA2&!NFkB&!SNAI1&!ZEB1&SNAI2 | BMI1&!Bcatenin&!YAP1&HNF1A&!HNF4A&!FOXA2&!NFkB&!SNAI1&ZEB1&!SNAI2&SOX9 | BMI1&!Bcatenin&!YAP1&HNF1A&!HNF4A&!FOXA2&!NFkB&!SNAI1&ZEB1&SNAI2 | BMI1&!Bcatenin&!YAP1&HNF1A&!HNF4A&!FOXA2&!NFkB&SNAI1&!ZEB1&!SNAI2&!SOX2 | BMI1&!Bcatenin&!YAP1&HNF1A&!HNF4A&!FOXA2&!NFkB&SNAI1&!ZEB1&SNAI2 | BMI1&!Bcatenin&!YAP1&HNF1A&!HNF4A&!FOXA2&!NFkB&SNAI1&ZEB1 | BMI1&!Bcatenin&!YAP1&HNF1A&!HNF4A&!FOXA2&NFkB&!ZEB1&!SNAI2&!SOX2 | BMI1&!Bcatenin&!YAP1&HNF1A&!HNF4A&!FOXA2&NFkB&!ZEB1&SNAI2 | BMI1&!Bcatenin&!YAP1&HNF1A&!HNF4A&!FOXA2&NFkB&ZEB1 | BMI1&!Bcatenin&YAP1&!HNF1A&!HNF4A&!NFkB&!SNAI1&!ZEB1&!SNAI2&SOX9&!SOX2 | BMI1&!Bcatenin&YAP1&!HNF1A&!HNF4A&!NFkB&!SNAI1&!ZEB1&SNAI2 | BMI1&!Bcatenin&YAP1&!HNF1A&!HNF4A&!NFkB&!SNAI1&ZEB1&!SNAI2&SOX9 | BMI1&!Bcatenin&YAP1&!HNF1A&!HNF4A&!NFkB&!SNAI1&ZEB1&SNAI2 | BMI1&!Bcatenin&YAP1&!HNF1A&!HNF4A&!NFkB&SNAI1&!ZEB1&!SNAI2&!SOX2 | BMI1&!Bcatenin&YAP1&!HNF1A&!HNF4A&!NFkB&SNAI1&!ZEB1&SNAI2 | BMI1&!Bcatenin&YAP1&!HNF1A&!HNF4A&!NFkB&SNAI1&ZEB1 | BMI1&!Bcatenin&YAP1&!HNF1A&!HNF4A&NFkB&!ZEB1&!SNAI2&!SOX2 | BMI1&!Bcatenin&YAP1&!HNF1A&!HNF4A&NFkB&!ZEB1&SNAI2 | BMI1&!Bcatenin&YAP1&!HNF1A&!HNF4A&NFkB&ZEB1 | BMI1&!Bcatenin&YAP1&!HNF1A&HNF4A&!FOXA2&!NFkB&!SNAI1&SNAI2 | BMI1&!Bcatenin&YAP1&!HNF1A&HNF4A&!FOXA2&!NFkB&SNAI1&!ZEB1&SNAI2 | BMI1&!Bcatenin&YAP1&!HNF1A&HNF4A&!FOXA2&!NFkB&SNAI1&ZEB1&!SNAI2&NANOG | BMI1&!Bcatenin&YAP1&!HNF1A&HNF4A&!FOXA2&!NFkB&SNAI1&ZEB1&SNAI2 | BMI1&!Bcatenin&YAP1&!HNF1A&HNF4A&!FOXA2&NFkB&!ZEB1&SNAI2 | BMI1&!Bcatenin&YAP1&!HNF1A&HNF4A&!FOXA2&NFkB&ZEB1 | BMI1&!Bcatenin&YAP1&!HNF1A&HNF4A&FOXA2&!NFkB&!SNAI1&SNAI2&NANOG | BMI1&!Bcatenin&YAP1&!HNF1A&HNF4A&FOXA2&!NFkB&SNAI1&!ZEB1&SNAI2&NANOG | BMI1&!Bcatenin&YAP1&!HNF1A&HNF4A&FOXA2&!NFkB&SNAI1&ZEB1&NANOG | BMI1&!Bcatenin&YAP1&!HNF1A&HNF4A&FOXA2&NFkB&!ZEB1&SNAI2&NANOG | BMI1&!Bcatenin&YAP1&!HNF1A&HNF4A&FOXA2&NFkB&ZEB1&NANOG | BMI1&!Bcatenin&YAP1&HNF1A&!HNF4A&!FOXA2&!NFkB&!SNAI1&!ZEB1&!SNAI2&SOX9&!SOX2 | BMI1&!Bcatenin&YAP1&HNF1A&!HNF4A&!FOXA2&!NFkB&!SNAI1&!ZEB1&SNAI2 | BMI1&!Bcatenin&YAP1&HNF1A&!HNF4A&!FOXA2&!NFkB&!SNAI1&ZEB1&!SNAI2&SOX9 | BMI1&!Bcatenin&YAP1&HNF1A&!HNF4A&!FOXA2&!NFkB&!SNAI1&ZEB1&SNAI2 | BMI1&!Bcatenin&YAP1&HNF1A&!HNF4A&!FOXA2&!NFkB&SNAI1&!ZEB1&!SNAI2&!SOX2 | BMI1&!Bcatenin&YAP1&HNF1A&!HNF4A&!FOXA2&!NFkB&SNAI1&!ZEB1&SNAI2 | BMI1&!Bcatenin&YAP1&HNF1A&!HNF4A&!FOXA2&!NFkB&SNAI1&ZEB1 | BMI1&!Bcatenin&YAP1&HNF1A&!HNF4A&!FOXA2&NFkB&!ZEB1&!SNAI2&!SOX2 | BMI1&!Bcatenin&YAP1&HNF1A&!HNF4A&!FOXA2&NFkB&!ZEB1&SNAI2 | BMI1&!Bcatenin&YAP1&HNF1A&!HNF4A&!FOXA2&NFkB&ZEB1 | BMI1&!Bcatenin&YAP1&HNF1A&!HNF4A&FOXA2&!NFkB&!SNAI1&SNAI2&NANOG | BMI1&!Bcatenin&YAP1&HNF1A&!HNF4A&FOXA2&!NFkB&SNAI1&!ZEB1&!SNAI2&NANOG&!SOX2 | BMI1&!Bcatenin&YAP1&HNF1A&!HNF4A&FOXA2&!NFkB&SNAI1&!ZEB1&SNAI2&NANOG | BMI1&!Bcatenin&YAP1&HNF1A&!HNF4A&FOXA2&!NFkB&SNAI1&ZEB1&NANOG | BMI1&!Bcatenin&YAP1&HNF1A&!HNF4A&FOXA2&NFkB&!SNAI1&!ZEB1&!SNAI2&NANOG&!SOX2&!p53 | BMI1&!Bcatenin&YAP1&HNF1A&!HNF4A&FOXA2&NFkB&!SNAI1&!ZEB1&SNAI2&NANOG | BMI1&!Bcatenin&YAP1&HNF1A&!HNF4A&FOXA2&NFkB&!SNAI1&ZEB1&NANOG | BMI1&!Bcatenin&YAP1&HNF1A&!HNF4A&FOXA2&NFkB&SNAI1&!ZEB1&!SNAI2&NANOG&!SOX2 | BMI1&!Bcatenin&YAP1&HNF1A&!HNF4A&FOXA2&NFkB&SNAI1&!ZEB1&SNAI2&NANOG | BMI1&!Bcatenin&YAP1&HNF1A&!HNF4A&FOXA2&NFkB&SNAI1&ZEB1&NANOG | BMI1&Bcatenin&!HNF1A&!HNF4A&!NFkB&!SNAI1&!ZEB1&!SNAI2&SOX9&!SOX2 | BMI1&Bcatenin&!HNF1A&!HNF4A&!NFkB&!SNAI1&!ZEB1&SNAI2 | BMI1&Bcatenin&!HNF1A&!HNF4A&!NFkB&!SNAI1&ZEB1&!SNAI2&SOX9 | BMI1&Bcatenin&!HNF1A&!HNF4A&!NFkB&!SNAI1&ZEB1&SNAI2 | BMI1&Bcatenin&!HNF1A&!HNF4A&!NFkB&SNAI1&!ZEB1&!SNAI2&!SOX2 | BMI1&Bcatenin&!HNF1A&!HNF4A&!NFkB&SNAI1&!ZEB1&SNAI2 | BMI1&Bcatenin&!HNF1A&!HNF4A&!NFkB&SNAI1&ZEB1 | BMI1&Bcatenin&!HNF1A&!HNF4A&NFkB&!ZEB1&!SNAI2&!SOX2 | BMI1&Bcatenin&!HNF1A&!HNF4A&NFkB&!ZEB1&SNAI2 | BMI1&Bcatenin&!HNF1A&!HNF4A&NFkB&ZEB1 | BMI1&Bcatenin&!HNF1A&HNF4A&!FOXA2&!NFkB&!SNAI1&SNAI2 | BMI1&Bcatenin&!HNF1A&HNF4A&!FOXA2&!NFkB&SNAI1&!ZEB1&SNAI2 | BMI1&Bcatenin&!HNF1A&HNF4A&!FOXA2&!NFkB&SNAI1&ZEB1&!SNAI2&NANOG | BMI1&Bcatenin&!HNF1A&HNF4A&!FOXA2&!NFkB&SNAI1&ZEB1&SNAI2 | BMI1&Bcatenin&!HNF1A&HNF4A&!FOXA2&NFkB&!ZEB1&SNAI2 | BMI1&Bcatenin&!HNF1A&HNF4A&!FOXA2&NFkB&ZEB1 | BMI1&Bcatenin&!HNF1A&HNF4A&FOXA2&!NFkB&!SNAI1&SNAI2&NANOG | BMI1&Bcatenin&!HNF1A&HNF4A&FOXA2&!NFkB&SNAI1&!ZEB1&SNAI2&NANOG | BMI1&Bcatenin&!HNF1A&HNF4A&FOXA2&!NFkB&SNAI1&ZEB1&NANOG | BMI1&Bcatenin&!HNF1A&HNF4A&FOXA2&NFkB&!ZEB1&SNAI2&NANOG | BMI1&Bcatenin&!HNF1A&HNF4A&FOXA2&NFkB&ZEB1&NANOG | BMI1&Bcatenin&HNF1A&!HNF4A&!FOXA2&!NFkB&!SNAI1&!ZEB1&!SNAI2&SOX9&!SOX2 | BMI1&Bcatenin&HNF1A&!HNF4A&!FOXA2&!NFkB&!SNAI1&!ZEB1&SNAI2 | BMI1&Bcatenin&HNF1A&!HNF4A&!FOXA2&!NFkB&!SNAI1&ZEB1&!SNAI2&SOX9 | BMI1&Bcatenin&HNF1A&!HNF4A&!FOXA2&!NFkB&!SNAI1&ZEB1&SNAI2 | BMI1&Bcatenin&HNF1A&!HNF4A&!FOXA2&!NFkB&SNAI1&!ZEB1&!SNAI2&!SOX2 | BMI1&Bcatenin&HNF1A&!HNF4A&!FOXA2&!NFkB&SNAI1&!ZEB1&SNAI2 | BMI1&Bcatenin&HNF1A&!HNF4A&!FOXA2&!NFkB&SNAI1&ZEB1 | BMI1&Bcatenin&HNF1A&!HNF4A&!FOXA2&NFkB&!ZEB1&!SNAI2&!SOX2 | BMI1&Bcatenin&HNF1A&!HNF4A&!FOXA2&NFkB&!ZEB1&SNAI2 | BMI1&Bcatenin&HNF1A&!HNF4A&!FOXA2&NFkB&ZEB1 | BMI1&Bcatenin&HNF1A&!HNF4A&FOXA2&!NFkB&!SNAI1&SNAI2&NANOG | BMI1&Bcatenin&HNF1A&!HNF4A&FOXA2&!NFkB&SNAI1&!ZEB1&!SNAI2&NANOG&!SOX2 | BMI1&Bcatenin&HNF1A&!HNF4A&FOXA2&!NFkB&SNAI1&!ZEB1&SNAI2&NANOG | BMI1&Bcatenin&HNF1A&!HNF4A&FOXA2&!NFkB&SNAI1&ZEB1&NANOG | BMI1&Bcatenin&HNF1A&!HNF4A&FOXA2&NFkB&!SNAI1&!ZEB1&!SNAI2&NANOG&!SOX2&!p53 | BMI1&Bcatenin&HNF1A&!HNF4A&FOXA2&NFkB&!SNAI1&!ZEB1&SNAI2&NANOG | BMI1&Bcatenin&HNF1A&!HNF4A&FOXA2&NFkB&!SNAI1&ZEB1&NANOG | BMI1&Bcatenin&HNF1A&!HNF4A&FOXA2&NFkB&SNAI1&!ZEB1&!SNAI2&NANOG&!SOX2 | BMI1&Bcatenin&HNF1A&!HNF4A&FOXA2&NFkB&SNAI1&!ZEB1&SNAI2&NANOG | BMI1&Bcatenin&HNF1A&!HNF4A&FOXA2&NFkB&SNAI1&ZEB1&NANOG

ZEB1, !BMI1&!E2F&!Bcatenin&!YAP1&!HNF1A&!HNF4A&!NFkB&!SNAI1&SNAI2 | !BMI1&!E2F&!Bcatenin&!YAP1&!HNF1A&!HNF4A&!NFkB&SNAI1 | !BMI1&!E2F&!Bcatenin&!YAP1&!HNF1A&!HNF4A&NFkB&!SNAI1&!ZEB1&!SNAI2&!SOX9 | !BMI1&!E2F&!Bcatenin&!YAP1&!HNF1A&!HNF4A&NFkB&!SNAI1&!ZEB1&!SNAI2&SOX9&!OCT4 | !BMI1&!E2F&!Bcatenin&!YAP1&!HNF1A&!HNF4A&NFkB&!SNAI1&!ZEB1&!SNAI2&SOX9&OCT4&!NANOG&!SOX2 | !BMI1&!E2F&!Bcatenin&!YAP1&!HNF1A&!HNF4A&NFkB&!SNAI1&!ZEB1&!SNAI2&SOX9&OCT4&!NANOG&SOX2&!p53 | !BMI1&!E2F&!Bcatenin&!YAP1&!HNF1A&!HNF4A&NFkB&!SNAI1&!ZEB1&!SNAI2&SOX9&OCT4&NANOG | !BMI1&!E2F&!Bcatenin&!YAP1&!HNF1A&!HNF4A&NFkB&!SNAI1&!ZEB1&SNAI2 | !BMI1&!E2F&!Bcatenin&!YAP1&!HNF1A&!HNF4A&NFkB&!SNAI1&ZEB1 | !BMI1&!E2F&!Bcatenin&!YAP1&!HNF1A&!HNF4A&NFkB&SNAI1 | !BMI1&!E2F&!Bcatenin&!YAP1&!HNF1A&HNF4A&!NFkB&!SNAI1&SNAI2 | !BMI1&!E2F&!Bcatenin&!YAP1&!HNF1A&HNF4A&!NFkB&SNAI1 | !BMI1&!E2F&!Bcatenin&!YAP1&!HNF1A&HNF4A&NFkB&!SNAI1&!ZEB1&!SNAI2&!SOX9&!NANOG&!SOX2 | !BMI1&!E2F&!Bcatenin&!YAP1&!HNF1A&HNF4A&NFkB&!SNAI1&!ZEB1&!SNAI2&!SOX9&!NANOG&SOX2&p53 | !BMI1&!E2F&!Bcatenin&!YAP1&!HNF1A&HNF4A&NFkB&!SNAI1&!ZEB1&!SNAI2&!SOX9&NANOG | !BMI1&!E2F&!Bcatenin&!YAP1&!HNF1A&HNF4A&NFkB&!SNAI1&!ZEB1&!SNAI2&SOX9&!NANOG&!SOX2 | !BMI1&!E2F&!Bcatenin&!YAP1&!HNF1A&HNF4A&NFkB&!SNAI1&!ZEB1&!SNAI2&SOX9&NANOG | !BMI1&!E2F&!Bcatenin&!YAP1&!HNF1A&HNF4A&NFkB&!SNAI1&!ZEB1&SNAI2 | !BMI1&!E2F&!Bcatenin&!YAP1&!HNF1A&HNF4A&NFkB&!SNAI1&ZEB1 | !BMI1&!E2F&!Bcatenin&!YAP1&!HNF1A&HNF4A&NFkB&SNAI1 | !BMI1&!E2F&!Bcatenin&YAP1&!HNF1A&!HNF4A&!NFkB&!SNAI1&SNAI2 | !BMI1&!E2F&!Bcatenin&YAP1&!HNF1A&!HNF4A&!NFkB&SNAI1 | !BMI1&!E2F&!Bcatenin&YAP1&!HNF1A&!HNF4A&NFkB&!SNAI1&!ZEB1&!SNAI2&!SOX9 | !BMI1&!E2F&!Bcatenin&YAP1&!HNF1A&!HNF4A&NFkB&!SNAI1&!ZEB1&!SNAI2&SOX9&!OCT4 | !BMI1&!E2F&!Bcatenin&YAP1&!HNF1A&!HNF4A&NFkB&!SNAI1&!ZEB1&!SNAI2&SOX9&OCT4&!NANOG&!SOX2 | !BMI1&!E2F&!Bcatenin&YAP1&!HNF1A&!HNF4A&NFkB&!SNAI1&!ZEB1&!SNAI2&SOX9&OCT4&!NANOG&SOX2&!p53 | !BMI1&!E2F&!Bcatenin&YAP1&!HNF1A&!HNF4A&NFkB&!SNAI1&!ZEB1&!SNAI2&SOX9&OCT4&NANOG | !BMI1&!E2F&!Bcatenin&YAP1&!HNF1A&!HNF4A&NFkB&!SNAI1&!ZEB1&SNAI2 | !BMI1&!E2F&!Bcatenin&YAP1&!HNF1A&!HNF4A&NFkB&!SNAI1&ZEB1 | !BMI1&!E2F&!Bcatenin&YAP1&!HNF1A&!HNF4A&NFkB&SNAI1 | !BMI1&!E2F&!Bcatenin&YAP1&!HNF1A&HNF4A&!NFkB&!SNAI1&SNAI2 | !BMI1&!E2F&!Bcatenin&YAP1&!HNF1A&HNF4A&!NFkB&SNAI1 | !BMI1&!E2F&!Bcatenin&YAP1&!HNF1A&HNF4A&NFkB&!SNAI1&!ZEB1&!SNAI2&!SOX9 | !BMI1&!E2F&!Bcatenin&YAP1&!HNF1A&HNF4A&NFkB&!SNAI1&!ZEB1&!SNAI2&SOX9&!NANOG&!SOX2 | !BMI1&!E2F&!Bcatenin&YAP1&!HNF1A&HNF4A&NFkB&!SNAI1&!ZEB1&!SNAI2&SOX9&!NANOG&SOX2&!p53 | !BMI1&!E2F&!Bcatenin&YAP1&!HNF1A&HNF4A&NFkB&!SNAI1&!ZEB1&!SNAI2&SOX9&NANOG | !BMI1&!E2F&!Bcatenin&YAP1&!HNF1A&HNF4A&NFkB&!SNAI1&!ZEB1&SNAI2 | !BMI1&!E2F&!Bcatenin&YAP1&!HNF1A&HNF4A&NFkB&!SNAI1&ZEB1 | !BMI1&!E2F&!Bcatenin&YAP1&!HNF1A&HNF4A&NFkB&SNAI1 | !BMI1&!E2F&Bcatenin&!YAP1&!HNF1A&!HNF4A&!SNAI1&!ZEB1&!SNAI2&!SOX9 | !BMI1&!E2F&Bcatenin&!YAP1&!HNF1A&!HNF4A&!SNAI1&!ZEB1&!SNAI2&SOX9&!OCT4 | !BMI1&!E2F&Bcatenin&!YAP1&!HNF1A&!HNF4A&!SNAI1&!ZEB1&!SNAI2&SOX9&OCT4&!NANOG&!SOX2 | !BMI1&!E2F&Bcatenin&!YAP1&!HNF1A&!HNF4A&!SNAI1&!ZEB1&!SNAI2&SOX9&OCT4&!NANOG&SOX2&!p53 | !BMI1&!E2F&Bcatenin&!YAP1&!HNF1A&!HNF4A&!SNAI1&!ZEB1&!SNAI2&SOX9&OCT4&NANOG | !BMI1&!E2F&Bcatenin&!YAP1&!HNF1A&!HNF4A&!SNAI1&!ZEB1&SNAI2 | !BMI1&!E2F&Bcatenin&!YAP1&!HNF1A&!HNF4A&!SNAI1&ZEB1 | !BMI1&!E2F&Bcatenin&!YAP1&!HNF1A&!HNF4A&SNAI1 | !BMI1&!E2F&Bcatenin&!YAP1&!HNF1A&HNF4A&!SNAI1&!ZEB1&!SNAI2&!TGFB&!SOX9 | !BMI1&!E2F&Bcatenin&!YAP1&!HNF1A&HNF4A&!SNAI1&!ZEB1&!SNAI2&!TGFB&SOX9&!NANOG&!SOX2 | !BMI1&!E2F&Bcatenin&!YAP1&!HNF1A&HNF4A&!SNAI1&!ZEB1&!SNAI2&!TGFB&SOX9&!NANOG&SOX2&!p53 | !BMI1&!E2F&Bcatenin&!YAP1&!HNF1A&HNF4A&!SNAI1&!ZEB1&!SNAI2&!TGFB&SOX9&NANOG | !BMI1&!E2F&Bcatenin&!YAP1&!HNF1A&HNF4A&!SNAI1&!ZEB1&!SNAI2&TGFB&!SOX9&!NANOG&!SOX2 | !BMI1&!E2F&Bcatenin&!YAP1&!HNF1A&HNF4A&!SNAI1&!ZEB1&!SNAI2&TGFB&!SOX9&!NANOG&SOX2&p53 | !BMI1&!E2F&Bcatenin&!YAP1&!HNF1A&HNF4A&!SNAI1&!ZEB1&!SNAI2&TGFB&!SOX9&NANOG | !BMI1&!E2F&Bcatenin&!YAP1&!HNF1A&HNF4A&!SNAI1&!ZEB1&!SNAI2&TGFB&SOX9&!NANOG&!SOX2 | !BMI1&!E2F&Bcatenin&!YAP1&!HNF1A&HNF4A&!SNAI1&!ZEB1&!SNAI2&TGFB&SOX9&NANOG | !BMI1&!E2F&Bcatenin&!YAP1&!HNF1A&HNF4A&!SNAI1&!ZEB1&SNAI2 | !BMI1&!E2F&Bcatenin&!YAP1&!HNF1A&HNF4A&!SNAI1&ZEB1 | !BMI1&!E2F&Bcatenin&!YAP1&!HNF1A&HNF4A&SNAI1 | !BMI1&!E2F&Bcatenin&YAP1&!HNF1A&!HNF4A&!SNAI1&!ZEB1&!SNAI2&!SOX9 | !BMI1&!E2F&Bcatenin&YAP1&!HNF1A&!HNF4A&!SNAI1&!ZEB1&!SNAI2&SOX9&!OCT4 | !BMI1&!E2F&Bcatenin&YAP1&!HNF1A&!HNF4A&!SNAI1&!ZEB1&!SNAI2&SOX9&OCT4&!NANOG&!SOX2 | !BMI1&!E2F&Bcatenin&YAP1&!HNF1A&!HNF4A&!SNAI1&!ZEB1&!SNAI2&SOX9&OCT4&!NANOG&SOX2&!p53 | !BMI1&!E2F&Bcatenin&YAP1&!HNF1A&!HNF4A&!SNAI1&!ZEB1&!SNAI2&SOX9&OCT4&NANOG | !BMI1&!E2F&Bcatenin&YAP1&!HNF1A&!HNF4A&!SNAI1&!ZEB1&SNAI2 | !BMI1&!E2F&Bcatenin&YAP1&!HNF1A&!HNF4A&!SNAI1&ZEB1 | !BMI1&!E2F&Bcatenin&YAP1&!HNF1A&!HNF4A&SNAI1 | !BMI1&!E2F&Bcatenin&YAP1&!HNF1A&HNF4A&!NFkB&!SNAI1&!ZEB1&!SNAI2&!TGFB&!SOX9 | !BMI1&!E2F&Bcatenin&YAP1&!HNF1A&HNF4A&!NFkB&!SNAI1&!ZEB1&!SNAI2&!TGFB&SOX9&!NANOG&!SOX2 | !BMI1&!E2F&Bcatenin&YAP1&!HNF1A&HNF4A&!NFkB&!SNAI1&!ZEB1&!SNAI2&!TGFB&SOX9&!NANOG&SOX2&!p53 | !BMI1&!E2F&Bcatenin&YAP1&!HNF1A&HNF4A&!NFkB&!SNAI1&!ZEB1&!SNAI2&!TGFB&SOX9&NANOG | !BMI1&!E2F&Bcatenin&YAP1&!HNF1A&HNF4A&!NFkB&!SNAI1&!ZEB1&!SNAI2&TGFB&!SOX9&!NANOG&!SOX2 | !BMI1&!E2F&Bcatenin&YAP1&!HNF1A&HNF4A&!NFkB&!SNAI1&!ZEB1&!SNAI2&TGFB&!SOX9&!NANOG&SOX2&p53 | !BMI1&!E2F&Bcatenin&YAP1&!HNF1A&HNF4A&!NFkB&!SNAI1&!ZEB1&!SNAI2&TGFB&!SOX9&NANOG | !BMI1&!E2F&Bcatenin&YAP1&!HNF1A&HNF4A&!NFkB&!SNAI1&!ZEB1&!SNAI2&TGFB&SOX9&!NANOG&!SOX2 | !BMI1&!E2F&Bcatenin&YAP1&!HNF1A&HNF4A&!NFkB&!SNAI1&!ZEB1&!SNAI2&TGFB&SOX9&NANOG | !BMI1&!E2F&Bcatenin&YAP1&!HNF1A&HNF4A&!NFkB&!SNAI1&!ZEB1&SNAI2 | !BMI1&!E2F&Bcatenin&YAP1&!HNF1A&HNF4A&!NFkB&!SNAI1&ZEB1 | !BMI1&!E2F&Bcatenin&YAP1&!HNF1A&HNF4A&!NFkB&SNAI1 | !BMI1&!E2F&Bcatenin&YAP1&!HNF1A&HNF4A&NFkB&!SNAI1&!ZEB1&!SNAI2&!SOX9 | !BMI1&!E2F&Bcatenin&YAP1&!HNF1A&HNF4A&NFkB&!SNAI1&!ZEB1&!SNAI2&SOX9&!NANOG&!SOX2 | !BMI1&!E2F&Bcatenin&YAP1&!HNF1A&HNF4A&NFkB&!SNAI1&!ZEB1&!SNAI2&SOX9&!NANOG&SOX2&!p53 | !BMI1&!E2F&Bcatenin&YAP1&!HNF1A&HNF4A&NFkB&!SNAI1&!ZEB1&!SNAI2&SOX9&NANOG | !BMI1&!E2F&Bcatenin&YAP1&!HNF1A&HNF4A&NFkB&!SNAI1&!ZEB1&SNAI2 | !BMI1&!E2F&Bcatenin&YAP1&!HNF1A&HNF4A&NFkB&!SNAI1&ZEB1 | !BMI1&!E2F&Bcatenin&YAP1&!HNF1A&HNF4A&NFkB&SNAI1 | !BMI1&E2F&!Bcatenin&!HNF1A&!HNF4A&!NFkB&!SNAI1&SNAI2 | !BMI1&E2F&!Bcatenin&!HNF1A&!HNF4A&!NFkB&SNAI1 | !BMI1&E2F&!Bcatenin&!HNF1A&!HNF4A&NFkB&!SNAI1&!ZEB1&!SNAI2&!SOX9 | !BMI1&E2F&!Bcatenin&!HNF1A&!HNF4A&NFkB&!SNAI1&!ZEB1&!SNAI2&SOX9&!OCT4 | !BMI1&E2F&!Bcatenin&!HNF1A&!HNF4A&NFkB&!SNAI1&!ZEB1&!SNAI2&SOX9&OCT4&!NANOG&!SOX2 | !BMI1&E2F&!Bcatenin&!HNF1A&!HNF4A&NFkB&!SNAI1&!ZEB1&!SNAI2&SOX9&OCT4&!NANOG&SOX2&!p53 | !BMI1&E2F&!Bcatenin&!HNF1A&!HNF4A&NFkB&!SNAI1&!ZEB1&!SNAI2&SOX9&OCT4&NANOG | !BMI1&E2F&!Bcatenin&!HNF1A&!HNF4A&NFkB&!SNAI1&!ZEB1&SNAI2 | !BMI1&E2F&!Bcatenin&!HNF1A&!HNF4A&NFkB&!SNAI1&ZEB1 | !BMI1&E2F&!Bcatenin&!HNF1A&!HNF4A&NFkB&SNAI1 | !BMI1&E2F&!Bcatenin&!HNF1A&HNF4A&!NFkB&!SNAI1&SNAI2 | !BMI1&E2F&!Bcatenin&!HNF1A&HNF4A&!NFkB&SNAI1 | !BMI1&E2F&!Bcatenin&!HNF1A&HNF4A&NFkB&!SNAI1&!ZEB1&!SNAI2&!SOX9 | !BMI1&E2F&!Bcatenin&!HNF1A&HNF4A&NFkB&!SNAI1&!ZEB1&!SNAI2&SOX9&!NANOG&!SOX2 | !BMI1&E2F&!Bcatenin&!HNF1A&HNF4A&NFkB&!SNAI1&!ZEB1&!SNAI2&SOX9&!NANOG&SOX2&!p53 | !BMI1&E2F&!Bcatenin&!HNF1A&HNF4A&NFkB&!SNAI1&!ZEB1&!SNAI2&SOX9&NANOG | !BMI1&E2F&!Bcatenin&!HNF1A&HNF4A&NFkB&!SNAI1&!ZEB1&SNAI2 | !BMI1&E2F&!Bcatenin&!HNF1A&HNF4A&NFkB&!SNAI1&ZEB1 | !BMI1&E2F&!Bcatenin&!HNF1A&HNF4A&NFkB&SNAI1 | !BMI1&E2F&Bcatenin&!HNF1A&!HNF4A&!SNAI1&!ZEB1&!SNAI2&!SOX9 | !BMI1&E2F&Bcatenin&!HNF1A&!HNF4A&!SNAI1&!ZEB1&!SNAI2&SOX9&!OCT4 | !BMI1&E2F&Bcatenin&!HNF1A&!HNF4A&!SNAI1&!ZEB1&!SNAI2&SOX9&OCT4&!NANOG&!SOX2 | !BMI1&E2F&Bcatenin&!HNF1A&!HNF4A&!SNAI1&!ZEB1&!SNAI2&SOX9&OCT4&!NANOG&SOX2&!p53 | !BMI1&E2F&Bcatenin&!HNF1A&!HNF4A&!SNAI1&!ZEB1&!SNAI2&SOX9&OCT4&NANOG | !BMI1&E2F&Bcatenin&!HNF1A&!HNF4A&!SNAI1&!ZEB1&SNAI2 | !BMI1&E2F&Bcatenin&!HNF1A&!HNF4A&!SNAI1&ZEB1 | !BMI1&E2F&Bcatenin&!HNF1A&!HNF4A&SNAI1 | !BMI1&E2F&Bcatenin&!HNF1A&HNF4A&!NFkB&!SNAI1&!ZEB1&!SNAI2&!TGFB&!SOX9 | !BMI1&E2F&Bcatenin&!HNF1A&HNF4A&!NFkB&!SNAI1&!ZEB1&!SNAI2&!TGFB&SOX9&!NANOG&!SOX2 | !BMI1&E2F&Bcatenin&!HNF1A&HNF4A&!NFkB&!SNAI1&!ZEB1&!SNAI2&!TGFB&SOX9&!NANOG&SOX2&!p53 | !BMI1&E2F&Bcatenin&!HNF1A&HNF4A&!NFkB&!SNAI1&!ZEB1&!SNAI2&!TGFB&SOX9&NANOG | !BMI1&E2F&Bcatenin&!HNF1A&HNF4A&!NFkB&!SNAI1&!ZEB1&!SNAI2&TGFB&!SOX9&!NANOG&!SOX2 | !BMI1&E2F&Bcatenin&!HNF1A&HNF4A&!NFkB&!SNAI1&!ZEB1&!SNAI2&TGFB&!SOX9&!NANOG&SOX2&p53 | !BMI1&E2F&Bcatenin&!HNF1A&HNF4A&!NFkB&!SNAI1&!ZEB1&!SNAI2&TGFB&!SOX9&NANOG | !BMI1&E2F&Bcatenin&!HNF1A&HNF4A&!NFkB&!SNAI1&!ZEB1&!SNAI2&TGFB&SOX9&!NANOG&!SOX2 | !BMI1&E2F&Bcatenin&!HNF1A&HNF4A&!NFkB&!SNAI1&!ZEB1&!SNAI2&TGFB&SOX9&NANOG | !BMI1&E2F&Bcatenin&!HNF1A&HNF4A&!NFkB&!SNAI1&!ZEB1&SNAI2 | !BMI1&E2F&Bcatenin&!HNF1A&HNF4A&!NFkB&!SNAI1&ZEB1 | !BMI1&E2F&Bcatenin&!HNF1A&HNF4A&!NFkB&SNAI1 | !BMI1&E2F&Bcatenin&!HNF1A&HNF4A&NFkB&!SNAI1&!ZEB1&!SNAI2&!SOX9 | !BMI1&E2F&Bcatenin&!HNF1A&HNF4A&NFkB&!SNAI1&!ZEB1&!SNAI2&SOX9&!NANOG&!SOX2 | !BMI1&E2F&Bcatenin&!HNF1A&HNF4A&NFkB&!SNAI1&!ZEB1&!SNAI2&SOX9&!NANOG&SOX2&!p53 | !BMI1&E2F&Bcatenin&!HNF1A&HNF4A&NFkB&!SNAI1&!ZEB1&!SNAI2&SOX9&NANOG | !BMI1&E2F&Bcatenin&!HNF1A&HNF4A&NFkB&!SNAI1&!ZEB1&SNAI2 | !BMI1&E2F&Bcatenin&!HNF1A&HNF4A&NFkB&!SNAI1&ZEB1 | !BMI1&E2F&Bcatenin&!HNF1A&HNF4A&NFkB&SNAI1 | BMI1&!Bcatenin&!HNF1A&!NFkB&!SNAI1&SNAI2 | BMI1&!Bcatenin&!HNF1A&!NFkB&SNAI1 | BMI1&!Bcatenin&!HNF1A&NFkB | BMI1&Bcatenin&!HNF1A

YAP1, Bcatenin&!HNF4A&SOX2&!p53

TGFB, !E2F&!Bcatenin&YAP1&!HNF4A&!NFkB&!SNAI1&!ZEB1&!SOX9&!NANOG&!SOX2&!p53 | !E2F&!Bcatenin&YAP1&!HNF4A&!NFkB&!SNAI1&!ZEB1&SOX9&!NANOG&!SOX2 | !E2F&!Bcatenin&YAP1&!HNF4A&!NFkB&!SNAI1&!ZEB1&SOX9&NANOG&p53 | !E2F&!Bcatenin&YAP1&!HNF4A&!NFkB&!SNAI1&ZEB1&!NANOG&!SOX2&!p53 | !E2F&!Bcatenin&YAP1&!HNF4A&!NFkB&SNAI1&!NANOG&!SOX2 | !E2F&!Bcatenin&YAP1&!HNF4A&!NFkB&SNAI1&NANOG | !E2F&!Bcatenin&YAP1&!HNF4A&NFkB&!SNAI1&!ZEB1&!SOX9&!NANOG&!p53 | !E2F&!Bcatenin&YAP1&!HNF4A&NFkB&!SNAI1&!ZEB1&SOX9&!NANOG&!SOX2 | !E2F&!Bcatenin&YAP1&!HNF4A&NFkB&!SNAI1&!ZEB1&SOX9&!NANOG&SOX2&!p53 | !E2F&!Bcatenin&YAP1&!HNF4A&NFkB&!SNAI1&!ZEB1&SOX9&NANOG&p53 | !E2F&!Bcatenin&YAP1&!HNF4A&NFkB&!SNAI1&ZEB1&!NANOG&!p53 | !E2F&!Bcatenin&YAP1&!HNF4A&NFkB&SNAI1&!NANOG&!SOX2 | !E2F&!Bcatenin&YAP1&!HNF4A&NFkB&SNAI1&!NANOG&SOX2&!p53 | !E2F&!Bcatenin&YAP1&!HNF4A&NFkB&SNAI1&NANOG | !E2F&!Bcatenin&YAP1&HNF4A&!NFkB&!SNAI1&!ZEB1&!SOX9&!NANOG&!SOX2&!p53 | !E2F&!Bcatenin&YAP1&HNF4A&!NFkB&!SNAI1&!ZEB1&SOX9&!NANOG&!SOX2 | !E2F&!Bcatenin&YAP1&HNF4A&!NFkB&!SNAI1&!ZEB1&SOX9&NANOG | !E2F&!Bcatenin&YAP1&HNF4A&!NFkB&!SNAI1&ZEB1&!NANOG&!SOX2&!p53 | !E2F&!Bcatenin&YAP1&HNF4A&!NFkB&SNAI1&!NANOG&!SOX2 | !E2F&!Bcatenin&YAP1&HNF4A&!NFkB&SNAI1&NANOG | !E2F&!Bcatenin&YAP1&HNF4A&NFkB&!SNAI1&!ZEB1&!SOX9&!NANOG&!p53 | !E2F&!Bcatenin&YAP1&HNF4A&NFkB&!SNAI1&!ZEB1&SOX9&!NANOG&!SOX2 | !E2F&!Bcatenin&YAP1&HNF4A&NFkB&!SNAI1&!ZEB1&SOX9&!NANOG&SOX2&!p53 | !E2F&!Bcatenin&YAP1&HNF4A&NFkB&!SNAI1&!ZEB1&SOX9&NANOG | !E2F&!Bcatenin&YAP1&HNF4A&NFkB&!SNAI1&ZEB1&!NANOG&!p53 | !E2F&!Bcatenin&YAP1&HNF4A&NFkB&SNAI1&!NANOG&!SOX2 | !E2F&!Bcatenin&YAP1&HNF4A&NFkB&SNAI1&!NANOG&SOX2&!p53 | !E2F&!Bcatenin&YAP1&HNF4A&NFkB&SNAI1&NANOG | !E2F&Bcatenin&YAP1&!HNF4A&!SNAI1&!ZEB1&!SOX9&!NANOG&!p53 | !E2F&Bcatenin&YAP1&!HNF4A&!SNAI1&!ZEB1&SOX9&!NANOG&!SOX2 | !E2F&Bcatenin&YAP1&!HNF4A&!SNAI1&!ZEB1&SOX9&!NANOG&SOX2&!p53 | !E2F&Bcatenin&YAP1&!HNF4A&!SNAI1&!ZEB1&SOX9&NANOG&p53 | !E2F&Bcatenin&YAP1&!HNF4A&!SNAI1&ZEB1&!NANOG&!p53 | !E2F&Bcatenin&YAP1&!HNF4A&SNAI1&!NANOG&!SOX2 | !E2F&Bcatenin&YAP1&!HNF4A&SNAI1&!NANOG&SOX2&!p53 | !E2F&Bcatenin&YAP1&!HNF4A&SNAI1&NANOG | !E2F&Bcatenin&YAP1&HNF4A&!NFkB&!SNAI1&!ZEB1&!TGFB&!SOX9&!NANOG&!p53 | !E2F&Bcatenin&YAP1&HNF4A&!NFkB&!SNAI1&!ZEB1&!TGFB&SOX9&!NANOG&!SOX2 | !E2F&Bcatenin&YAP1&HNF4A&!NFkB&!SNAI1&!ZEB1&!TGFB&SOX9&!NANOG&SOX2&!p53 | !E2F&Bcatenin&YAP1&HNF4A&!NFkB&!SNAI1&!ZEB1&!TGFB&SOX9&NANOG | !E2F&Bcatenin&YAP1&HNF4A&!NFkB&!SNAI1&!ZEB1&TGFB&!SOX9&!NANOG&!SOX2&!p53 | !E2F&Bcatenin&YAP1&HNF4A&!NFkB&!SNAI1&!ZEB1&TGFB&SOX9&!NANOG&!SOX2 | !E2F&Bcatenin&YAP1&HNF4A&!NFkB&!SNAI1&!ZEB1&TGFB&SOX9&NANOG | !E2F&Bcatenin&YAP1&HNF4A&!NFkB&!SNAI1&ZEB1&!NANOG&!p53 | !E2F&Bcatenin&YAP1&HNF4A&!NFkB&SNAI1&!NANOG&!SOX2 | !E2F&Bcatenin&YAP1&HNF4A&!NFkB&SNAI1&!NANOG&SOX2&!p53 | !E2F&Bcatenin&YAP1&HNF4A&!NFkB&SNAI1&NANOG | !E2F&Bcatenin&YAP1&HNF4A&NFkB&!SNAI1&!ZEB1&!SOX9&!NANOG&!p53 | !E2F&Bcatenin&YAP1&HNF4A&NFkB&!SNAI1&!ZEB1&SOX9&!NANOG&!SOX2 | !E2F&Bcatenin&YAP1&HNF4A&NFkB&!SNAI1&!ZEB1&SOX9&!NANOG&SOX2&!p53 | !E2F&Bcatenin&YAP1&HNF4A&NFkB&!SNAI1&!ZEB1&SOX9&NANOG | !E2F&Bcatenin&YAP1&HNF4A&NFkB&!SNAI1&ZEB1&!NANOG&!p53 | !E2F&Bcatenin&YAP1&HNF4A&NFkB&SNAI1&!NANOG&!SOX2 | !E2F&Bcatenin&YAP1&HNF4A&NFkB&SNAI1&!NANOG&SOX2&!p53 | !E2F&Bcatenin&YAP1&HNF4A&NFkB&SNAI1&NANOG | E2F&YAP1&!HNF4A&!SNAI1&!ZEB1&!SOX9&!NANOG&!p53 | E2F&YAP1&!HNF4A&!SNAI1&!ZEB1&SOX9&!NANOG&!SOX2 | E2F&YAP1&!HNF4A&!SNAI1&!ZEB1&SOX9&!NANOG&SOX2&!p53 | E2F&YAP1&!HNF4A&!SNAI1&!ZEB1&SOX9&NANOG&p53 | E2F&YAP1&!HNF4A&!SNAI1&ZEB1&!NANOG&!p53 | E2F&YAP1&!HNF4A&SNAI1&!NANOG&!SOX2 | E2F&YAP1&!HNF4A&SNAI1&!NANOG&SOX2&!p53 | E2F&YAP1&!HNF4A&SNAI1&NANOG | E2F&YAP1&HNF4A&!NFkB&!SNAI1&!ZEB1&!TGFB&!SOX9&!NANOG&!p53 | E2F&YAP1&HNF4A&!NFkB&!SNAI1&!ZEB1&!TGFB&SOX9&!NANOG&!SOX2 | E2F&YAP1&HNF4A&!NFkB&!SNAI1&!ZEB1&!TGFB&SOX9&!NANOG&SOX2&!p53 | E2F&YAP1&HNF4A&!NFkB&!SNAI1&!ZEB1&!TGFB&SOX9&NANOG | E2F&YAP1&HNF4A&!NFkB&!SNAI1&!ZEB1&TGFB&!SOX9&!NANOG&!SOX2&!p53 | E2F&YAP1&HNF4A&!NFkB&!SNAI1&!ZEB1&TGFB&SOX9&!NANOG&!SOX2 | E2F&YAP1&HNF4A&!NFkB&!SNAI1&!ZEB1&TGFB&SOX9&NANOG | E2F&YAP1&HNF4A&!NFkB&!SNAI1&ZEB1&!NANOG&!p53 | E2F&YAP1&HNF4A&!NFkB&SNAI1&!NANOG&!SOX2 | E2F&YAP1&HNF4A&!NFkB&SNAI1&!NANOG&SOX2&!p53 | E2F&YAP1&HNF4A&!NFkB&SNAI1&NANOG | E2F&YAP1&HNF4A&NFkB&!SNAI1&!ZEB1&!SOX9&!NANOG&!p53 | E2F&YAP1&HNF4A&NFkB&!SNAI1&!ZEB1&SOX9&!NANOG&!SOX2 | E2F&YAP1&HNF4A&NFkB&!SNAI1&!ZEB1&SOX9&!NANOG&SOX2&!p53 | E2F&YAP1&HNF4A&NFkB&!SNAI1&!ZEB1&SOX9&NANOG | E2F&YAP1&HNF4A&NFkB&!SNAI1&ZEB1&!NANOG&!p53 | E2F&YAP1&HNF4A&NFkB&SNAI1&!NANOG&!SOX2 | E2F&YAP1&HNF4A&NFkB&SNAI1&!NANOG&SOX2&!p53 | E2F&YAP1&HNF4A&NFkB&SNAI1&NANOG

OCT4, !YAP1&NANOG&SOX2&!p53&!RB | YAP1&!HNF4A&!SNAI1&!OCT4&NANOG&!p53 | YAP1&!HNF4A&!SNAI1&OCT4&!NANOG&SOX2&!p53 | YAP1&!HNF4A&!SNAI1&OCT4&NANOG&!p53 | YAP1&!HNF4A&SNAI1&!OCT4&NANOG&SOX2&!p53&!RB | YAP1&!HNF4A&SNAI1&OCT4&!NANOG&SOX2&!p53 | YAP1&!HNF4A&SNAI1&OCT4&NANOG&!p53 | YAP1&HNF4A&!SNAI1&!ZEB1&!SOX9&!OCT4&NANOG&!p53 | YAP1&HNF4A&!SNAI1&!ZEB1&!SOX9&OCT4&!NANOG&SOX2&!p53 | YAP1&HNF4A&!SNAI1&!ZEB1&!SOX9&OCT4&NANOG&!p53 | YAP1&HNF4A&!SNAI1&!ZEB1&SOX9&!OCT4&NANOG&SOX2&!p53&!RB | YAP1&HNF4A&!SNAI1&!ZEB1&SOX9&OCT4&!NANOG&SOX2&!p53 | YAP1&HNF4A&!SNAI1&!ZEB1&SOX9&OCT4&NANOG&!p53 | YAP1&HNF4A&!SNAI1&ZEB1&!OCT4&NANOG&!p53 | YAP1&HNF4A&!SNAI1&ZEB1&OCT4&!NANOG&SOX2&!p53 | YAP1&HNF4A&!SNAI1&ZEB1&OCT4&NANOG&!p53 | YAP1&HNF4A&SNAI1&!OCT4&NANOG&SOX2&!p53&!RB | YAP1&HNF4A&SNAI1&OCT4&!NANOG&SOX2&!p53 | YAP1&HNF4A&SNAI1&OCT4&NANOG&!p53

SOX2, !BMI1&!E2F&!Bcatenin&!YAP1&OCT4&NANOG&SOX2&!p53&!p21&!RB | !BMI1&!E2F&!Bcatenin&YAP1&!HNF4A&!OCT4&NANOG&!p53&!p21&!RB | !BMI1&!E2F&!Bcatenin&YAP1&!HNF4A&OCT4&!p53&!p21&!RB | !BMI1&!E2F&!Bcatenin&YAP1&HNF4A&!NFkB&!SNAI1&!ZEB1&OCT4&NANOG&SOX2&!p53&!p21&!RB | !BMI1&!E2F&!Bcatenin&YAP1&HNF4A&!NFkB&!SNAI1&ZEB1&!OCT4&NANOG&!p53&!p21&!RB | !BMI1&!E2F&!Bcatenin&YAP1&HNF4A&!NFkB&!SNAI1&ZEB1&OCT4&!p53&!p21&!RB | !BMI1&!E2F&!Bcatenin&YAP1&HNF4A&!NFkB&SNAI1&!OCT4&NANOG&!p53&!p21&!RB | !BMI1&!E2F&!Bcatenin&YAP1&HNF4A&!NFkB&SNAI1&OCT4&!p53&!p21&!RB | !BMI1&!E2F&!Bcatenin&YAP1&HNF4A&NFkB&!SNAI1&!ZEB1&!OCT4&NANOG&SOX2&!p53&!p21&!RB | !BMI1&!E2F&!Bcatenin&YAP1&HNF4A&NFkB&!SNAI1&!ZEB1&OCT4&NANOG&!p53&!p21&!RB | !BMI1&!E2F&!Bcatenin&YAP1&HNF4A&NFkB&!SNAI1&ZEB1&!OCT4&NANOG&!p53&!p21&!RB | !BMI1&!E2F&!Bcatenin&YAP1&HNF4A&NFkB&!SNAI1&ZEB1&OCT4&!p53&!p21&!RB | !BMI1&!E2F&!Bcatenin&YAP1&HNF4A&NFkB&SNAI1&!OCT4&NANOG&!p53&!p21&!RB | !BMI1&!E2F&!Bcatenin&YAP1&HNF4A&NFkB&SNAI1&OCT4&!p53&!p21&!RB | !BMI1&!E2F&Bcatenin&!YAP1&!HNF4A&!OCT4&NANOG&!p53&!p21&!RB | !BMI1&!E2F&Bcatenin&!YAP1&!HNF4A&OCT4&!NANOG&!p53&!p21&!RB | !BMI1&!E2F&Bcatenin&!YAP1&!HNF4A&OCT4&NANOG&!p53&!p21 | !BMI1&!E2F&Bcatenin&!YAP1&HNF4A&!SNAI1&!ZEB1&!TGFB&!OCT4&NANOG&SOX2&!p53&!p21&!RB | !BMI1&!E2F&Bcatenin&!YAP1&HNF4A&!SNAI1&!ZEB1&!TGFB&OCT4&NANOG&!p53&!p21 | !BMI1&!E2F&Bcatenin&!YAP1&HNF4A&!SNAI1&!ZEB1&TGFB&OCT4&NANOG&!p53&!p21 | !BMI1&!E2F&Bcatenin&!YAP1&HNF4A&!SNAI1&ZEB1&!OCT4&NANOG&!p53&!p21&!RB | !BMI1&!E2F&Bcatenin&!YAP1&HNF4A&!SNAI1&ZEB1&OCT4&!NANOG&!p53&!p21&!RB | !BMI1&!E2F&Bcatenin&!YAP1&HNF4A&!SNAI1&ZEB1&OCT4&NANOG&!p53&!p21 | !BMI1&!E2F&Bcatenin&!YAP1&HNF4A&SNAI1&!OCT4&NANOG&!p53&!p21&!RB | !BMI1&!E2F&Bcatenin&!YAP1&HNF4A&SNAI1&OCT4&!NANOG&!p53&!p21&!RB | !BMI1&!E2F&Bcatenin&!YAP1&HNF4A&SNAI1&OCT4&NANOG&!p53&!p21 | !BMI1&!E2F&Bcatenin&YAP1&!HNF4A&!OCT4&NANOG&!p53&!p21&!RB | !BMI1&!E2F&Bcatenin&YAP1&!HNF4A&OCT4&!NANOG&!p53&!p21&!RB | !BMI1&!E2F&Bcatenin&YAP1&!HNF4A&OCT4&NANOG&!p53&!p21 | !BMI1&!E2F&Bcatenin&YAP1&HNF4A&!NFkB&!SNAI1&!ZEB1&!TGFB&!OCT4&NANOG&SOX2&!p53&!p21&!RB | !BMI1&!E2F&Bcatenin&YAP1&HNF4A&!NFkB&!SNAI1&!ZEB1&!TGFB&OCT4&NANOG&!p53&!p21 | !BMI1&!E2F&Bcatenin&YAP1&HNF4A&!NFkB&!SNAI1&!ZEB1&TGFB&OCT4&NANOG&!p53&!p21 | !BMI1&!E2F&Bcatenin&YAP1&HNF4A&!NFkB&!SNAI1&ZEB1&!OCT4&NANOG&!p53&!p21&!RB | !BMI1&!E2F&Bcatenin&YAP1&HNF4A&!NFkB&!SNAI1&ZEB1&OCT4&!NANOG&!p53&!p21&!RB | !BMI1&!E2F&Bcatenin&YAP1&HNF4A&!NFkB&!SNAI1&ZEB1&OCT4&NANOG&!p53&!p21 | !BMI1&!E2F&Bcatenin&YAP1&HNF4A&!NFkB&SNAI1&!OCT4&NANOG&!p53&!p21&!RB | !BMI1&!E2F&Bcatenin&YAP1&HNF4A&!NFkB&SNAI1&OCT4&!NANOG&!p53&!p21&!RB | !BMI1&!E2F&Bcatenin&YAP1&HNF4A&!NFkB&SNAI1&OCT4&NANOG&!p53&!p21 | !BMI1&!E2F&Bcatenin&YAP1&HNF4A&NFkB&!SNAI1&!ZEB1&!OCT4&NANOG&SOX2&!p53&!p21&!RB | !BMI1&!E2F&Bcatenin&YAP1&HNF4A&NFkB&!SNAI1&!ZEB1&OCT4&NANOG&!p53&!p21 | !BMI1&!E2F&Bcatenin&YAP1&HNF4A&NFkB&!SNAI1&ZEB1&!OCT4&NANOG&!p53&!p21&!RB | !BMI1&!E2F&Bcatenin&YAP1&HNF4A&NFkB&!SNAI1&ZEB1&OCT4&!NANOG&!p53&!p21&!RB | !BMI1&!E2F&Bcatenin&YAP1&HNF4A&NFkB&!SNAI1&ZEB1&OCT4&NANOG&!p53&!p21 | !BMI1&!E2F&Bcatenin&YAP1&HNF4A&NFkB&SNAI1&!OCT4&NANOG&!p53&!p21&!RB | !BMI1&!E2F&Bcatenin&YAP1&HNF4A&NFkB&SNAI1&OCT4&!NANOG&!p53&!p21&!RB | !BMI1&!E2F&Bcatenin&YAP1&HNF4A&NFkB&SNAI1&OCT4&NANOG&!p53&!p21 | !BMI1&E2F&!Bcatenin&!YAP1&!HNF4A&!NFkB&NANOG&SOX2&!p53&!p21&!RB | !BMI1&E2F&!Bcatenin&!YAP1&!HNF4A&NFkB&!OCT4&NANOG&SOX2&!p53&!p21&!RB | !BMI1&E2F&!Bcatenin&!YAP1&!HNF4A&NFkB&OCT4&NANOG&!p53&!p21&!RB | !BMI1&E2F&!Bcatenin&!YAP1&HNF4A&!NFkB&!SNAI1&!ZEB1&!TGFB&NANOG&SOX2&!p53&!p21&!RB | !BMI1&E2F&!Bcatenin&!YAP1&HNF4A&!NFkB&!SNAI1&!ZEB1&TGFB&OCT4&NANOG&SOX2&!p53&!p21&!RB | !BMI1&E2F&!Bcatenin&!YAP1&HNF4A&!NFkB&!SNAI1&ZEB1&NANOG&SOX2&!p53&!p21&!RB | !BMI1&E2F&!Bcatenin&!YAP1&HNF4A&!NFkB&SNAI1&NANOG&SOX2&!p53&!p21&!RB | !BMI1&E2F&!Bcatenin&!YAP1&HNF4A&NFkB&!OCT4&NANOG&SOX2&!p53&!p21&!RB | !BMI1&E2F&!Bcatenin&!YAP1&HNF4A&NFkB&OCT4&NANOG&!p53&!p21&!RB | !BMI1&E2F&!Bcatenin&YAP1&!HNF4A&!OCT4&NANOG&!p53&!p21&!RB | !BMI1&E2F&!Bcatenin&YAP1&!HNF4A&OCT4&!p53&!p21&!RB | !BMI1&E2F&!Bcatenin&YAP1&HNF4A&!NFkB&!SNAI1&!ZEB1&!TGFB&!OCT4&NANOG&SOX2&!p53&!p21&!RB | !BMI1&E2F&!Bcatenin&YAP1&HNF4A&!NFkB&!SNAI1&!ZEB1&!TGFB&OCT4&NANOG&!p53&!p21&!RB | !BMI1&E2F&!Bcatenin&YAP1&HNF4A&!NFkB&!SNAI1&!ZEB1&TGFB&OCT4&NANOG&SOX2&!p53&!p21&!RB | !BMI1&E2F&!Bcatenin&YAP1&HNF4A&!NFkB&!SNAI1&ZEB1&!OCT4&NANOG&!p53&!p21&!RB | !BMI1&E2F&!Bcatenin&YAP1&HNF4A&!NFkB&!SNAI1&ZEB1&OCT4&!p53&!p21&!RB | !BMI1&E2F&!Bcatenin&YAP1&HNF4A&!NFkB&SNAI1&!OCT4&NANOG&!p53&!p21&!RB | !BMI1&E2F&!Bcatenin&YAP1&HNF4A&!NFkB&SNAI1&OCT4&!p53&!p21&!RB | !BMI1&E2F&!Bcatenin&YAP1&HNF4A&NFkB&!SNAI1&!ZEB1&!OCT4&NANOG&SOX2&!p53&!p21&!RB | !BMI1&E2F&!Bcatenin&YAP1&HNF4A&NFkB&!SNAI1&!ZEB1&OCT4&NANOG&!p53&!p21&!RB | !BMI1&E2F&!Bcatenin&YAP1&HNF4A&NFkB&!SNAI1&ZEB1&!OCT4&NANOG&!p53&!p21&!RB | !BMI1&E2F&!Bcatenin&YAP1&HNF4A&NFkB&!SNAI1&ZEB1&OCT4&!p53&!p21&!RB | !BMI1&E2F&!Bcatenin&YAP1&HNF4A&NFkB&SNAI1&!OCT4&NANOG&!p53&!p21&!RB | !BMI1&E2F&!Bcatenin&YAP1&HNF4A&NFkB&SNAI1&OCT4&!p53&!p21&!RB | !BMI1&E2F&Bcatenin&!HNF4A&!OCT4&NANOG&!p53&!p21&!RB | !BMI1&E2F&Bcatenin&!HNF4A&OCT4&!NANOG&!p53&!p21&!RB | !BMI1&E2F&Bcatenin&!HNF4A&OCT4&NANOG&!p53&!p21 | !BMI1&E2F&Bcatenin&HNF4A&!NFkB&!SNAI1&!ZEB1&!TGFB&!OCT4&NANOG&SOX2&!p53&!p21&!RB | !BMI1&E2F&Bcatenin&HNF4A&!NFkB&!SNAI1&!ZEB1&!TGFB&OCT4&NANOG&!p53&!p21 | !BMI1&E2F&Bcatenin&HNF4A&!NFkB&!SNAI1&!ZEB1&TGFB&OCT4&NANOG&!p53&!p21 | !BMI1&E2F&Bcatenin&HNF4A&!NFkB&!SNAI1&ZEB1&!OCT4&NANOG&!p53&!p21&!RB | !BMI1&E2F&Bcatenin&HNF4A&!NFkB&!SNAI1&ZEB1&OCT4&!NANOG&!p53&!p21&!RB | !BMI1&E2F&Bcatenin&HNF4A&!NFkB&!SNAI1&ZEB1&OCT4&NANOG&!p53&!p21 | !BMI1&E2F&Bcatenin&HNF4A&!NFkB&SNAI1&!OCT4&NANOG&!p53&!p21&!RB | !BMI1&E2F&Bcatenin&HNF4A&!NFkB&SNAI1&OCT4&!NANOG&!p53&!p21&!RB | !BMI1&E2F&Bcatenin&HNF4A&!NFkB&SNAI1&OCT4&NANOG&!p53&!p21 | !BMI1&E2F&Bcatenin&HNF4A&NFkB&!SNAI1&!ZEB1&!OCT4&NANOG&SOX2&!p53&!p21&!RB | !BMI1&E2F&Bcatenin&HNF4A&NFkB&!SNAI1&!ZEB1&OCT4&NANOG&!p53&!p21 | !BMI1&E2F&Bcatenin&HNF4A&NFkB&!SNAI1&ZEB1&!OCT4&NANOG&!p53&!p21&!RB | !BMI1&E2F&Bcatenin&HNF4A&NFkB&!SNAI1&ZEB1&OCT4&!NANOG&!p53&!p21&!RB | !BMI1&E2F&Bcatenin&HNF4A&NFkB&!SNAI1&ZEB1&OCT4&NANOG&!p53&!p21 | !BMI1&E2F&Bcatenin&HNF4A&NFkB&SNAI1&!OCT4&NANOG&!p53&!p21&!RB | !BMI1&E2F&Bcatenin&HNF4A&NFkB&SNAI1&OCT4&!NANOG&!p53&!p21&!RB | !BMI1&E2F&Bcatenin&HNF4A&NFkB&SNAI1&OCT4&NANOG&!p53&!p21 | BMI1&!E2F&!Bcatenin&!YAP1&OCT4&NANOG&SOX2&!p53&!p21&!RB | BMI1&!E2F&!Bcatenin&YAP1&!OCT4&NANOG&!p53&!p21&!RB | BMI1&!E2F&!Bcatenin&YAP1&OCT4&!p53&!p21&!RB | BMI1&!E2F&Bcatenin&!OCT4&NANOG&!p53&!p21&!RB | BMI1&!E2F&Bcatenin&OCT4&!NANOG&!p53&!p21&!RB | BMI1&!E2F&Bcatenin&OCT4&NANOG&!p53&!p21 | BMI1&E2F&!Bcatenin&!YAP1&!HNF4A&!NFkB&NANOG&SOX2&!p53&!p21&!RB | BMI1&E2F&!Bcatenin&!YAP1&!HNF4A&NFkB&!OCT4&NANOG&SOX2&!p53&!p21&!RB | BMI1&E2F&!Bcatenin&!YAP1&!HNF4A&NFkB&OCT4&NANOG&!p53&!p21&!RB | BMI1&E2F&!Bcatenin&!YAP1&HNF4A&!NFkB&!SNAI1&!ZEB1&!TGFB&NANOG&SOX2&!p53&!p21&!RB | BMI1&E2F&!Bcatenin&!YAP1&HNF4A&!NFkB&!SNAI1&!ZEB1&TGFB&OCT4&NANOG&SOX2&!p53&!p21&!RB | BMI1&E2F&!Bcatenin&!YAP1&HNF4A&!NFkB&!SNAI1&ZEB1&NANOG&SOX2&!p53&!p21&!RB | BMI1&E2F&!Bcatenin&!YAP1&HNF4A&!NFkB&SNAI1&NANOG&SOX2&!p53&!p21&!RB | BMI1&E2F&!Bcatenin&!YAP1&HNF4A&NFkB&!OCT4&NANOG&SOX2&!p53&!p21&!RB | BMI1&E2F&!Bcatenin&!YAP1&HNF4A&NFkB&OCT4&NANOG&!p53&!p21&!RB | BMI1&E2F&!Bcatenin&YAP1&!OCT4&NANOG&!p53&!p21&!RB | BMI1&E2F&!Bcatenin&YAP1&OCT4&!p53&!p21&!RB | BMI1&E2F&Bcatenin&!OCT4&NANOG&!p53&!p21&!RB | BMI1&E2F&Bcatenin&OCT4&!NANOG&!p53&!p21&!RB | BMI1&E2F&Bcatenin&OCT4&NANOG&!p53&!p21

NANOG, !E2F&!Bcatenin&!NFkB&!SNAI1&!TGFB&!OCT4&NANOG&SOX2&!p53 | !E2F&!Bcatenin&!NFkB&!SNAI1&!TGFB&OCT4&NANOG&!p53 | !E2F&!Bcatenin&!NFkB&!SNAI1&TGFB&!OCT4&NANOG&SOX2&!p53 | !E2F&!Bcatenin&!NFkB&!SNAI1&TGFB&OCT4&!NANOG&SOX2&!p53 | !E2F&!Bcatenin&!NFkB&!SNAI1&TGFB&OCT4&NANOG&!p53 | !E2F&!Bcatenin&!NFkB&SNAI1&TGFB&OCT4&SOX2&!p53 | !E2F&!Bcatenin&NFkB&!OCT4&NANOG&SOX2&!p53 | !E2F&!Bcatenin&NFkB&OCT4&!NANOG&SOX2&!p53 | !E2F&!Bcatenin&NFkB&OCT4&NANOG&!p53 | !E2F&Bcatenin&!HNF4A&!NFkB&!SNAI1&!OCT4&NANOG&SOX2&!p53 | !E2F&Bcatenin&!HNF4A&!NFkB&!SNAI1&OCT4&!NANOG&SOX2&!p53 | !E2F&Bcatenin&!HNF4A&!NFkB&!SNAI1&OCT4&NANOG&!p53 | !E2F&Bcatenin&!HNF4A&!NFkB&SNAI1&TGFB&OCT4&SOX2&!p53 | !E2F&Bcatenin&!HNF4A&NFkB&!OCT4&NANOG&SOX2&!p53 | !E2F&Bcatenin&!HNF4A&NFkB&OCT4&!NANOG&SOX2&!p53 | !E2F&Bcatenin&!HNF4A&NFkB&OCT4&NANOG&!p53 | !E2F&Bcatenin&HNF4A&!NFkB&!SNAI1&!ZEB1&!TGFB&!OCT4&NANOG&SOX2&!p53 | !E2F&Bcatenin&HNF4A&!NFkB&!SNAI1&!ZEB1&!TGFB&OCT4&NANOG&!p53 | !E2F&Bcatenin&HNF4A&!NFkB&!SNAI1&!ZEB1&TGFB&!OCT4&NANOG&SOX2&!p53 | !E2F&Bcatenin&HNF4A&!NFkB&!SNAI1&!ZEB1&TGFB&OCT4&!NANOG&SOX2&!p53 | !E2F&Bcatenin&HNF4A&!NFkB&!SNAI1&!ZEB1&TGFB&OCT4&NANOG&!p53 | !E2F&Bcatenin&HNF4A&!NFkB&!SNAI1&ZEB1&!OCT4&NANOG&SOX2&!p53 | !E2F&Bcatenin&HNF4A&!NFkB&!SNAI1&ZEB1&OCT4&!NANOG&SOX2&!p53 | !E2F&Bcatenin&HNF4A&!NFkB&!SNAI1&ZEB1&OCT4&NANOG&!p53 | !E2F&Bcatenin&HNF4A&!NFkB&SNAI1&TGFB&OCT4&SOX2&!p53 | !E2F&Bcatenin&HNF4A&NFkB&!OCT4&NANOG&SOX2&!p53 | !E2F&Bcatenin&HNF4A&NFkB&OCT4&!NANOG&SOX2&!p53 | !E2F&Bcatenin&HNF4A&NFkB&OCT4&NANOG&!p53 | E2F&!HNF4A&!NFkB&!SNAI1&!OCT4&NANOG&SOX2&!p53 | E2F&!HNF4A&!NFkB&!SNAI1&OCT4&!NANOG&SOX2&!p53 | E2F&!HNF4A&!NFkB&!SNAI1&OCT4&NANOG&!p53 | E2F&!HNF4A&!NFkB&SNAI1&TGFB&OCT4&SOX2&!p53 | E2F&!HNF4A&NFkB&!OCT4&NANOG&SOX2&!p53 | E2F&!HNF4A&NFkB&OCT4&!NANOG&SOX2&!p53 | E2F&!HNF4A&NFkB&OCT4&NANOG&!p53 | E2F&HNF4A&!NFkB&!SNAI1&!ZEB1&!TGFB&!OCT4&NANOG&SOX2&!p53 | E2F&HNF4A&!NFkB&!SNAI1&!ZEB1&!TGFB&OCT4&NANOG&!p53 | E2F&HNF4A&!NFkB&!SNAI1&!ZEB1&TGFB&!OCT4&NANOG&SOX2&!p53 | E2F&HNF4A&!NFkB&!SNAI1&!ZEB1&TGFB&OCT4&!NANOG&SOX2&!p53 | E2F&HNF4A&!NFkB&!SNAI1&!ZEB1&TGFB&OCT4&NANOG&!p53 | E2F&HNF4A&!NFkB&!SNAI1&ZEB1&!OCT4&NANOG&SOX2&!p53 | E2F&HNF4A&!NFkB&!SNAI1&ZEB1&OCT4&!NANOG&SOX2&!p53 | E2F&HNF4A&!NFkB&!SNAI1&ZEB1&OCT4&NANOG&!p53 | E2F&HNF4A&!NFkB&SNAI1&TGFB&OCT4&SOX2&!p53 | E2F&HNF4A&NFkB&!OCT4&NANOG&SOX2&!p53 | E2F&HNF4A&NFkB&OCT4&!NANOG&SOX2&!p53 | E2F&HNF4A&NFkB&OCT4&NANOG&!p53

SOX9, YAP1&!FOXA2&!NFkB&SOX9 | YAP1&!FOXA2&NFkB | YAP1&FOXA2

p16, !EZH2&!BMI1&!E2F | !EZH2&!BMI1&E2F&!Bcatenin&!YAP1&!HNF4A&!NFkB&!SOX2 | !EZH2&!BMI1&E2F&!Bcatenin&!YAP1&!HNF4A&!NFkB&SOX2&p53 | !EZH2&!BMI1&E2F&!Bcatenin&!YAP1&!HNF4A&NFkB&p53 | !EZH2&!BMI1&E2F&!Bcatenin&!YAP1&HNF4A&!NFkB&!SNAI1&!ZEB1&!TGFB&!SOX2 | !EZH2&!BMI1&E2F&!Bcatenin&!YAP1&HNF4A&!NFkB&!SNAI1&!ZEB1&!TGFB&SOX2&p53 | !EZH2&!BMI1&E2F&!Bcatenin&!YAP1&HNF4A&!NFkB&!SNAI1&!ZEB1&TGFB | !EZH2&!BMI1&E2F&!Bcatenin&!YAP1&HNF4A&!NFkB&!SNAI1&ZEB1&!SOX2 | !EZH2&!BMI1&E2F&!Bcatenin&!YAP1&HNF4A&!NFkB&!SNAI1&ZEB1&SOX2&p53 | !EZH2&!BMI1&E2F&!Bcatenin&!YAP1&HNF4A&!NFkB&SNAI1&!SOX2 | !EZH2&!BMI1&E2F&!Bcatenin&!YAP1&HNF4A&!NFkB&SNAI1&SOX2&p53 | !EZH2&!BMI1&E2F&!Bcatenin&!YAP1&HNF4A&NFkB&p53 | !EZH2&!BMI1&E2F&!Bcatenin&YAP1&!HNF4A&p53 | !EZH2&!BMI1&E2F&!Bcatenin&YAP1&HNF4A&!NFkB&!SNAI1&!ZEB1&!TGFB&p53 | !EZH2&!BMI1&E2F&!Bcatenin&YAP1&HNF4A&!NFkB&!SNAI1&!ZEB1&TGFB | !EZH2&!BMI1&E2F&!Bcatenin&YAP1&HNF4A&!NFkB&!SNAI1&ZEB1&p53 | !EZH2&!BMI1&E2F&!Bcatenin&YAP1&HNF4A&!NFkB&SNAI1&p53 | !EZH2&!BMI1&E2F&!Bcatenin&YAP1&HNF4A&NFkB&p53 | !EZH2&!BMI1&E2F&Bcatenin&!HNF4A&p53 | !EZH2&!BMI1&E2F&Bcatenin&HNF4A&!NFkB&!SNAI1&!ZEB1&!TGFB&p53 | !EZH2&!BMI1&E2F&Bcatenin&HNF4A&!NFkB&!SNAI1&!ZEB1&TGFB | !EZH2&!BMI1&E2F&Bcatenin&HNF4A&!NFkB&!SNAI1&ZEB1&p53 | !EZH2&!BMI1&E2F&Bcatenin&HNF4A&!NFkB&SNAI1&p53 | !EZH2&!BMI1&E2F&Bcatenin&HNF4A&NFkB&p53 | !EZH2&BMI1&!E2F&!SNAI2 | !EZH2&BMI1&E2F&!Bcatenin&!YAP1&!HNF4A&!NFkB&!SNAI2&!SOX2 | !EZH2&BMI1&E2F&!Bcatenin&!YAP1&!HNF4A&!NFkB&!SNAI2&SOX2&p53 | !EZH2&BMI1&E2F&!Bcatenin&!YAP1&!HNF4A&NFkB&!SNAI2&p53 | !EZH2&BMI1&E2F&!Bcatenin&!YAP1&HNF4A&!NFkB&!SNAI1&!ZEB1&!SNAI2&!TGFB&!SOX2 | !EZH2&BMI1&E2F&!Bcatenin&!YAP1&HNF4A&!NFkB&!SNAI1&!ZEB1&!SNAI2&!TGFB&SOX2&p53 | !EZH2&BMI1&E2F&!Bcatenin&!YAP1&HNF4A&!NFkB&!SNAI1&!ZEB1&!SNAI2&TGFB | !EZH2&BMI1&E2F&!Bcatenin&!YAP1&HNF4A&!NFkB&!SNAI1&ZEB1&!SNAI2&!SOX2 | !EZH2&BMI1&E2F&!Bcatenin&!YAP1&HNF4A&!NFkB&!SNAI1&ZEB1&!SNAI2&SOX2&p53 | !EZH2&BMI1&E2F&!Bcatenin&!YAP1&HNF4A&!NFkB&SNAI1&!SNAI2&!SOX2 | !EZH2&BMI1&E2F&!Bcatenin&!YAP1&HNF4A&!NFkB&SNAI1&!SNAI2&SOX2&p53 | !EZH2&BMI1&E2F&!Bcatenin&!YAP1&HNF4A&NFkB&!SNAI2&p53 | !EZH2&BMI1&E2F&!Bcatenin&YAP1&!HNF4A&!SNAI2&p53 | !EZH2&BMI1&E2F&!Bcatenin&YAP1&HNF4A&!NFkB&!SNAI1&!ZEB1&!SNAI2&!TGFB&p53 | !EZH2&BMI1&E2F&!Bcatenin&YAP1&HNF4A&!NFkB&!SNAI1&!ZEB1&!SNAI2&TGFB | !EZH2&BMI1&E2F&!Bcatenin&YAP1&HNF4A&!NFkB&!SNAI1&ZEB1&!SNAI2&p53 | !EZH2&BMI1&E2F&!Bcatenin&YAP1&HNF4A&!NFkB&SNAI1&!SNAI2&p53 | !EZH2&BMI1&E2F&!Bcatenin&YAP1&HNF4A&NFkB&!SNAI2&p53 | !EZH2&BMI1&E2F&Bcatenin&!HNF4A&!SNAI2&p53 | !EZH2&BMI1&E2F&Bcatenin&HNF4A&!NFkB&!SNAI1&!ZEB1&!SNAI2&!TGFB&p53 | !EZH2&BMI1&E2F&Bcatenin&HNF4A&!NFkB&!SNAI1&!ZEB1&!SNAI2&TGFB | !EZH2&BMI1&E2F&Bcatenin&HNF4A&!NFkB&!SNAI1&ZEB1&!SNAI2&p53 | !EZH2&BMI1&E2F&Bcatenin&HNF4A&!NFkB&SNAI1&!SNAI2&p53 | !EZH2&BMI1&E2F&Bcatenin&HNF4A&NFkB&!SNAI2&p53

p53, !EZH2&!BMI1&!E2F&!HNF4A&!HNF6&!NFkB&!SNAI1&!NANOG&p53 | !EZH2&!BMI1&!E2F&!HNF4A&!HNF6&!NFkB&!SNAI1&NANOG | !EZH2&!BMI1&!E2F&!HNF4A&!HNF6&!NFkB&SNAI1&p53 | !EZH2&!BMI1&!E2F&!HNF4A&!HNF6&NFkB&!SNAI1&!OCT4 | !EZH2&!BMI1&!E2F&!HNF4A&!HNF6&NFkB&!SNAI1&OCT4&!NANOG&p53 | !EZH2&!BMI1&!E2F&!HNF4A&!HNF6&NFkB&!SNAI1&OCT4&NANOG | !EZH2&!BMI1&!E2F&!HNF4A&!HNF6&NFkB&SNAI1&!OCT4 | !EZH2&!BMI1&!E2F&!HNF4A&!HNF6&NFkB&SNAI1&OCT4&p53 | !EZH2&!BMI1&!E2F&!HNF4A&HNF6 | !EZH2&!BMI1&!E2F&HNF4A&!HNF6&!NFkB&!SNAI1&!ZEB1&!SOX9&!NANOG&p53 | !EZH2&!BMI1&!E2F&HNF4A&!HNF6&!NFkB&!SNAI1&!ZEB1&!SOX9&NANOG | !EZH2&!BMI1&!E2F&HNF4A&!HNF6&!NFkB&!SNAI1&!ZEB1&SOX9&p53 | !EZH2&!BMI1&!E2F&HNF4A&!HNF6&!NFkB&!SNAI1&ZEB1&!NANOG&p53 | !EZH2&!BMI1&!E2F&HNF4A&!HNF6&!NFkB&!SNAI1&ZEB1&NANOG | !EZH2&!BMI1&!E2F&HNF4A&!HNF6&!NFkB&SNAI1&p53 | !EZH2&!BMI1&!E2F&HNF4A&!HNF6&NFkB&!SNAI1&!ZEB1&!SOX9&!NANOG&p53 | !EZH2&!BMI1&!E2F&HNF4A&!HNF6&NFkB&!SNAI1&!ZEB1&!SOX9&NANOG | !EZH2&!BMI1&!E2F&HNF4A&!HNF6&NFkB&!SNAI1&!ZEB1&SOX9&p53 | !EZH2&!BMI1&!E2F&HNF4A&!HNF6&NFkB&!SNAI1&ZEB1&!OCT4 | !EZH2&!BMI1&!E2F&HNF4A&!HNF6&NFkB&!SNAI1&ZEB1&OCT4&!NANOG&p53 | !EZH2&!BMI1&!E2F&HNF4A&!HNF6&NFkB&!SNAI1&ZEB1&OCT4&NANOG | !EZH2&!BMI1&!E2F&HNF4A&!HNF6&NFkB&SNAI1&!OCT4 | !EZH2&!BMI1&!E2F&HNF4A&!HNF6&NFkB&SNAI1&OCT4&p53 | !EZH2&!BMI1&!E2F&HNF4A&HNF6 | !EZH2&!BMI1&E2F&!Bcatenin&!YAP1&!HNF4A&!HNF6&!NFkB&!SNAI1&!TGFB&!NANOG&p53 | !EZH2&!BMI1&E2F&!Bcatenin&!YAP1&!HNF4A&!HNF6&!NFkB&!SNAI1&!TGFB&NANOG | !EZH2&!BMI1&E2F&!Bcatenin&!YAP1&!HNF4A&!HNF6&!NFkB&!SNAI1&TGFB&!NANOG&p53 | !EZH2&!BMI1&E2F&!Bcatenin&!YAP1&!HNF4A&!HNF6&!NFkB&!SNAI1&TGFB&NANOG&!SOX2 | !EZH2&!BMI1&E2F&!Bcatenin&!YAP1&!HNF4A&!HNF6&!NFkB&!SNAI1&TGFB&NANOG&SOX2&!p53&p16 | !EZH2&!BMI1&E2F&!Bcatenin&!YAP1&!HNF4A&!HNF6&!NFkB&!SNAI1&TGFB&NANOG&SOX2&p53 | !EZH2&!BMI1&E2F&!Bcatenin&!YAP1&!HNF4A&!HNF6&!NFkB&SNAI1&p53 | !EZH2&!BMI1&E2F&!Bcatenin&!YAP1&!HNF4A&!HNF6&NFkB&!SNAI1&!OCT4&!p53&p16 | !EZH2&!BMI1&E2F&!Bcatenin&!YAP1&!HNF4A&!HNF6&NFkB&!SNAI1&!OCT4&p53 | !EZH2&!BMI1&E2F&!Bcatenin&!YAP1&!HNF4A&!HNF6&NFkB&!SNAI1&OCT4&!NANOG&p53 | !EZH2&!BMI1&E2F&!Bcatenin&!YAP1&!HNF4A&!HNF6&NFkB&!SNAI1&OCT4&NANOG&!p53&p16 | !EZH2&!BMI1&E2F&!Bcatenin&!YAP1&!HNF4A&!HNF6&NFkB&!SNAI1&OCT4&NANOG&p53 | !EZH2&!BMI1&E2F&!Bcatenin&!YAP1&!HNF4A&!HNF6&NFkB&SNAI1&!OCT4&!p53&p16 | !EZH2&!BMI1&E2F&!Bcatenin&!YAP1&!HNF4A&!HNF6&NFkB&SNAI1&!OCT4&p53 | !EZH2&!BMI1&E2F&!Bcatenin&!YAP1&!HNF4A&!HNF6&NFkB&SNAI1&OCT4&p53 | !EZH2&!BMI1&E2F&!Bcatenin&!YAP1&!HNF4A&HNF6&!NFkB&!TGFB | !EZH2&!BMI1&E2F&!Bcatenin&!YAP1&!HNF4A&HNF6&!NFkB&TGFB&!SOX2 | !EZH2&!BMI1&E2F&!Bcatenin&!YAP1&!HNF4A&HNF6&!NFkB&TGFB&SOX2&!p53&p16 | !EZH2&!BMI1&E2F&!Bcatenin&!YAP1&!HNF4A&HNF6&!NFkB&TGFB&SOX2&p53 | !EZH2&!BMI1&E2F&!Bcatenin&!YAP1&!HNF4A&HNF6&NFkB&!p53&p16 | !EZH2&!BMI1&E2F&!Bcatenin&!YAP1&!HNF4A&HNF6&NFkB&p53 | !EZH2&!BMI1&E2F&!Bcatenin&!YAP1&HNF4A&!HNF6&!NFkB&!SNAI1&!ZEB1&!SOX9&!NANOG&p53 | !EZH2&!BMI1&E2F&!Bcatenin&!YAP1&HNF4A&!HNF6&!NFkB&!SNAI1&!ZEB1&!SOX9&NANOG | !EZH2&!BMI1&E2F&!Bcatenin&!YAP1&HNF4A&!HNF6&!NFkB&!SNAI1&!ZEB1&SOX9&p53 | !EZH2&!BMI1&E2F&!Bcatenin&!YAP1&HNF4A&!HNF6&!NFkB&!SNAI1&ZEB1&!TGFB&!NANOG&p53 | !EZH2&!BMI1&E2F&!Bcatenin&!YAP1&HNF4A&!HNF6&!NFkB&!SNAI1&ZEB1&!TGFB&NANOG | !EZH2&!BMI1&E2F&!Bcatenin&!YAP1&HNF4A&!HNF6&!NFkB&!SNAI1&ZEB1&TGFB&!NANOG&p53 | !EZH2&!BMI1&E2F&!Bcatenin&!YAP1&HNF4A&!HNF6&!NFkB&!SNAI1&ZEB1&TGFB&NANOG&!SOX2 | !EZH2&!BMI1&E2F&!Bcatenin&!YAP1&HNF4A&!HNF6&!NFkB&!SNAI1&ZEB1&TGFB&NANOG&SOX2&!p53&p16 | !EZH2&!BMI1&E2F&!Bcatenin&!YAP1&HNF4A&!HNF6&!NFkB&!SNAI1&ZEB1&TGFB&NANOG&SOX2&p53 | !EZH2&!BMI1&E2F&!Bcatenin&!YAP1&HNF4A&!HNF6&!NFkB&SNAI1&p53 | !EZH2&!BMI1&E2F&!Bcatenin&!YAP1&HNF4A&!HNF6&NFkB&!SNAI1&!ZEB1&!SOX9&!NANOG&p53 | !EZH2&!BMI1&E2F&!Bcatenin&!YAP1&HNF4A&!HNF6&NFkB&!SNAI1&!ZEB1&!SOX9&NANOG&!p53&p16 | !EZH2&!BMI1&E2F&!Bcatenin&!YAP1&HNF4A&!HNF6&NFkB&!SNAI1&!ZEB1&!SOX9&NANOG&p53 | !EZH2&!BMI1&E2F&!Bcatenin&!YAP1&HNF4A&!HNF6&NFkB&!SNAI1&!ZEB1&SOX9&p53 | !EZH2&!BMI1&E2F&!Bcatenin&!YAP1&HNF4A&!HNF6&NFkB&!SNAI1&ZEB1&!OCT4&!p53&p16 | !EZH2&!BMI1&E2F&!Bcatenin&!YAP1&HNF4A&!HNF6&NFkB&!SNAI1&ZEB1&!OCT4&p53 | !EZH2&!BMI1&E2F&!Bcatenin&!YAP1&HNF4A&!HNF6&NFkB&!SNAI1&ZEB1&OCT4&!NANOG&p53 | !EZH2&!BMI1&E2F&!Bcatenin&!YAP1&HNF4A&!HNF6&NFkB&!SNAI1&ZEB1&OCT4&NANOG&!p53&p16 | !EZH2&!BMI1&E2F&!Bcatenin&!YAP1&HNF4A&!HNF6&NFkB&!SNAI1&ZEB1&OCT4&NANOG&p53 | !EZH2&!BMI1&E2F&!Bcatenin&!YAP1&HNF4A&!HNF6&NFkB&SNAI1&!OCT4&!p53&p16 | !EZH2&!BMI1&E2F&!Bcatenin&!YAP1&HNF4A&!HNF6&NFkB&SNAI1&!OCT4&p53 | !EZH2&!BMI1&E2F&!Bcatenin&!YAP1&HNF4A&!HNF6&NFkB&SNAI1&OCT4&p53 | !EZH2&!BMI1&E2F&!Bcatenin&!YAP1&HNF4A&HNF6&!NFkB&!SNAI1&!ZEB1 | !EZH2&!BMI1&E2F&!Bcatenin&!YAP1&HNF4A&HNF6&!NFkB&!SNAI1&ZEB1&!TGFB | !EZH2&!BMI1&E2F&!Bcatenin&!YAP1&HNF4A&HNF6&!NFkB&!SNAI1&ZEB1&TGFB&!SOX2 | !EZH2&!BMI1&E2F&!Bcatenin&!YAP1&HNF4A&HNF6&!NFkB&!SNAI1&ZEB1&TGFB&SOX2&!p53&p16 | !EZH2&!BMI1&E2F&!Bcatenin&!YAP1&HNF4A&HNF6&!NFkB&!SNAI1&ZEB1&TGFB&SOX2&p53 | !EZH2&!BMI1&E2F&!Bcatenin&!YAP1&HNF4A&HNF6&!NFkB&SNAI1&!TGFB | !EZH2&!BMI1&E2F&!Bcatenin&!YAP1&HNF4A&HNF6&!NFkB&SNAI1&TGFB&!SOX2 | !EZH2&!BMI1&E2F&!Bcatenin&!YAP1&HNF4A&HNF6&!NFkB&SNAI1&TGFB&SOX2&!p53&p16 | !EZH2&!BMI1&E2F&!Bcatenin&!YAP1&HNF4A&HNF6&!NFkB&SNAI1&TGFB&SOX2&p53 | !EZH2&!BMI1&E2F&!Bcatenin&!YAP1&HNF4A&HNF6&NFkB&!p53&p16 | !EZH2&!BMI1&E2F&!Bcatenin&!YAP1&HNF4A&HNF6&NFkB&p53 | !EZH2&!BMI1&E2F&!Bcatenin&YAP1&!HNF4A&!HNF6&!NFkB&!SNAI1&!TGFB&!NANOG&p53 | !EZH2&!BMI1&E2F&!Bcatenin&YAP1&!HNF4A&!HNF6&!NFkB&!SNAI1&!TGFB&NANOG | !EZH2&!BMI1&E2F&!Bcatenin&YAP1&!HNF4A&!HNF6&!NFkB&!SNAI1&TGFB&!NANOG&p53 | !EZH2&!BMI1&E2F&!Bcatenin&YAP1&!HNF4A&!HNF6&!NFkB&!SNAI1&TGFB&NANOG&!p53&p16 | !EZH2&!BMI1&E2F&!Bcatenin&YAP1&!HNF4A&!HNF6&!NFkB&!SNAI1&TGFB&NANOG&p53 | !EZH2&!BMI1&E2F&!Bcatenin&YAP1&!HNF4A&!HNF6&!NFkB&SNAI1&p53 | !EZH2&!BMI1&E2F&!Bcatenin&YAP1&!HNF4A&!HNF6&NFkB&!SNAI1&!OCT4&!p53&p16 | !EZH2&!BMI1&E2F&!Bcatenin&YAP1&!HNF4A&!HNF6&NFkB&!SNAI1&!OCT4&p53 | !EZH2&!BMI1&E2F&!Bcatenin&YAP1&!HNF4A&!HNF6&NFkB&!SNAI1&OCT4&!NANOG&p53 | !EZH2&!BMI1&E2F&!Bcatenin&YAP1&!HNF4A&!HNF6&NFkB&!SNAI1&OCT4&NANOG&!p53&p16 | !EZH2&!BMI1&E2F&!Bcatenin&YAP1&!HNF4A&!HNF6&NFkB&!SNAI1&OCT4&NANOG&p53 | !EZH2&!BMI1&E2F&!Bcatenin&YAP1&!HNF4A&!HNF6&NFkB&SNAI1&!OCT4&!p53&p16 | !EZH2&!BMI1&E2F&!Bcatenin&YAP1&!HNF4A&!HNF6&NFkB&SNAI1&!OCT4&p53 | !EZH2&!BMI1&E2F&!Bcatenin&YAP1&!HNF4A&!HNF6&NFkB&SNAI1&OCT4&p53 | !EZH2&!BMI1&E2F&!Bcatenin&YAP1&!HNF4A&HNF6&!NFkB&!TGFB | !EZH2&!BMI1&E2F&!Bcatenin&YAP1&!HNF4A&HNF6&!NFkB&TGFB&!p53&p16 | !EZH2&!BMI1&E2F&!Bcatenin&YAP1&!HNF4A&HNF6&!NFkB&TGFB&p53 | !EZH2&!BMI1&E2F&!Bcatenin&YAP1&!HNF4A&HNF6&NFkB&!p53&p16 | !EZH2&!BMI1&E2F&!Bcatenin&YAP1&!HNF4A&HNF6&NFkB&p53 | !EZH2&!BMI1&E2F&!Bcatenin&YAP1&HNF4A&!HNF6&!NFkB&!SNAI1&!ZEB1&!SOX9&!NANOG&p53 | !EZH2&!BMI1&E2F&!Bcatenin&YAP1&HNF4A&!HNF6&!NFkB&!SNAI1&!ZEB1&!SOX9&NANOG | !EZH2&!BMI1&E2F&!Bcatenin&YAP1&HNF4A&!HNF6&!NFkB&!SNAI1&!ZEB1&SOX9&p53 | !EZH2&!BMI1&E2F&!Bcatenin&YAP1&HNF4A&!HNF6&!NFkB&!SNAI1&ZEB1&!TGFB&!NANOG&p53 | !EZH2&!BMI1&E2F&!Bcatenin&YAP1&HNF4A&!HNF6&!NFkB&!SNAI1&ZEB1&!TGFB&NANOG | !EZH2&!BMI1&E2F&!Bcatenin&YAP1&HNF4A&!HNF6&!NFkB&!SNAI1&ZEB1&TGFB&!NANOG&p53 | !EZH2&!BMI1&E2F&!Bcatenin&YAP1&HNF4A&!HNF6&!NFkB&!SNAI1&ZEB1&TGFB&NANOG&!p53&p16 | !EZH2&!BMI1&E2F&!Bcatenin&YAP1&HNF4A&!HNF6&!NFkB&!SNAI1&ZEB1&TGFB&NANOG&p53 | !EZH2&!BMI1&E2F&!Bcatenin&YAP1&HNF4A&!HNF6&!NFkB&SNAI1&p53 | !EZH2&!BMI1&E2F&!Bcatenin&YAP1&HNF4A&!HNF6&NFkB&!SNAI1&!ZEB1&!SOX9&!NANOG&p53 | !EZH2&!BMI1&E2F&!Bcatenin&YAP1&HNF4A&!HNF6&NFkB&!SNAI1&!ZEB1&!SOX9&NANOG&!p53&p16 | !EZH2&!BMI1&E2F&!Bcatenin&YAP1&HNF4A&!HNF6&NFkB&!SNAI1&!ZEB1&!SOX9&NANOG&p53 | !EZH2&!BMI1&E2F&!Bcatenin&YAP1&HNF4A&!HNF6&NFkB&!SNAI1&!ZEB1&SOX9&p53 | !EZH2&!BMI1&E2F&!Bcatenin&YAP1&HNF4A&!HNF6&NFkB&!SNAI1&ZEB1&!OCT4&!p53&p16 | !EZH2&!BMI1&E2F&!Bcatenin&YAP1&HNF4A&!HNF6&NFkB&!SNAI1&ZEB1&!OCT4&p53 | !EZH2&!BMI1&E2F&!Bcatenin&YAP1&HNF4A&!HNF6&NFkB&!SNAI1&ZEB1&OCT4&!NANOG&p53 | !EZH2&!BMI1&E2F&!Bcatenin&YAP1&HNF4A&!HNF6&NFkB&!SNAI1&ZEB1&OCT4&NANOG&!p53&p16 | !EZH2&!BMI1&E2F&!Bcatenin&YAP1&HNF4A&!HNF6&NFkB&!SNAI1&ZEB1&OCT4&NANOG&p53 | !EZH2&!BMI1&E2F&!Bcatenin&YAP1&HNF4A&!HNF6&NFkB&SNAI1&!OCT4&!p53&p16 | !EZH2&!BMI1&E2F&!Bcatenin&YAP1&HNF4A&!HNF6&NFkB&SNAI1&!OCT4&p53 | !EZH2&!BMI1&E2F&!Bcatenin&YAP1&HNF4A&!HNF6&NFkB&SNAI1&OCT4&p53 | !EZH2&!BMI1&E2F&!Bcatenin&YAP1&HNF4A&HNF6&!NFkB&!SNAI1&!ZEB1 | !EZH2&!BMI1&E2F&!Bcatenin&YAP1&HNF4A&HNF6&!NFkB&!SNAI1&ZEB1&!TGFB | !EZH2&!BMI1&E2F&!Bcatenin&YAP1&HNF4A&HNF6&!NFkB&!SNAI1&ZEB1&TGFB&!p53&p16 | !EZH2&!BMI1&E2F&!Bcatenin&YAP1&HNF4A&HNF6&!NFkB&!SNAI1&ZEB1&TGFB&p53 | !EZH2&!BMI1&E2F&!Bcatenin&YAP1&HNF4A&HNF6&!NFkB&SNAI1&!TGFB | !EZH2&!BMI1&E2F&!Bcatenin&YAP1&HNF4A&HNF6&!NFkB&SNAI1&TGFB&!p53&p16 | !EZH2&!BMI1&E2F&!Bcatenin&YAP1&HNF4A&HNF6&!NFkB&SNAI1&TGFB&p53 | !EZH2&!BMI1&E2F&!Bcatenin&YAP1&HNF4A&HNF6&NFkB&!p53&p16 | !EZH2&!BMI1&E2F&!Bcatenin&YAP1&HNF4A&HNF6&NFkB&p53 | !EZH2&!BMI1&E2F&Bcatenin&!HNF4A&!HNF6&!NFkB&!SNAI1&!TGFB&!NANOG&p53 | !EZH2&!BMI1&E2F&Bcatenin&!HNF4A&!HNF6&!NFkB&!SNAI1&!TGFB&NANOG | !EZH2&!BMI1&E2F&Bcatenin&!HNF4A&!HNF6&!NFkB&!SNAI1&TGFB&!NANOG&p53 | !EZH2&!BMI1&E2F&Bcatenin&!HNF4A&!HNF6&!NFkB&!SNAI1&TGFB&NANOG&!p53&p16 | !EZH2&!BMI1&E2F&Bcatenin&!HNF4A&!HNF6&!NFkB&!SNAI1&TGFB&NANOG&p53 | !EZH2&!BMI1&E2F&Bcatenin&!HNF4A&!HNF6&!NFkB&SNAI1&p53 | !EZH2&!BMI1&E2F&Bcatenin&!HNF4A&!HNF6&NFkB&!SNAI1&!OCT4&!p53&p16 | !EZH2&!BMI1&E2F&Bcatenin&!HNF4A&!HNF6&NFkB&!SNAI1&!OCT4&p53 | !EZH2&!BMI1&E2F&Bcatenin&!HNF4A&!HNF6&NFkB&!SNAI1&OCT4&!NANOG&p53 | !EZH2&!BMI1&E2F&Bcatenin&!HNF4A&!HNF6&NFkB&!SNAI1&OCT4&NANOG&!p53&p16 | !EZH2&!BMI1&E2F&Bcatenin&!HNF4A&!HNF6&NFkB&!SNAI1&OCT4&NANOG&p53 | !EZH2&!BMI1&E2F&Bcatenin&!HNF4A&!HNF6&NFkB&SNAI1&!OCT4&!p53&p16 | !EZH2&!BMI1&E2F&Bcatenin&!HNF4A&!HNF6&NFkB&SNAI1&!OCT4&p53 | !EZH2&!BMI1&E2F&Bcatenin&!HNF4A&!HNF6&NFkB&SNAI1&OCT4&p53 | !EZH2&!BMI1&E2F&Bcatenin&!HNF4A&HNF6&!NFkB&!TGFB | !EZH2&!BMI1&E2F&Bcatenin&!HNF4A&HNF6&!NFkB&TGFB&!p53&p16 | !EZH2&!BMI1&E2F&Bcatenin&!HNF4A&HNF6&!NFkB&TGFB&p53 | !EZH2&!BMI1&E2F&Bcatenin&!HNF4A&HNF6&NFkB&!p53&p16 | !EZH2&!BMI1&E2F&Bcatenin&!HNF4A&HNF6&NFkB&p53 | !EZH2&!BMI1&E2F&Bcatenin&HNF4A&!HNF6&!NFkB&!SNAI1&!ZEB1&!SOX9&!NANOG&p53 | !EZH2&!BMI1&E2F&Bcatenin&HNF4A&!HNF6&!NFkB&!SNAI1&!ZEB1&!SOX9&NANOG | !EZH2&!BMI1&E2F&Bcatenin&HNF4A&!HNF6&!NFkB&!SNAI1&!ZEB1&SOX9&p53 | !EZH2&!BMI1&E2F&Bcatenin&HNF4A&!HNF6&!NFkB&!SNAI1&ZEB1&!TGFB&!NANOG&p53 | !EZH2&!BMI1&E2F&Bcatenin&HNF4A&!HNF6&!NFkB&!SNAI1&ZEB1&!TGFB&NANOG | !EZH2&!BMI1&E2F&Bcatenin&HNF4A&!HNF6&!NFkB&!SNAI1&ZEB1&TGFB&!NANOG&p53 | !EZH2&!BMI1&E2F&Bcatenin&HNF4A&!HNF6&!NFkB&!SNAI1&ZEB1&TGFB&NANOG&!p53&p16 | !EZH2&!BMI1&E2F&Bcatenin&HNF4A&!HNF6&!NFkB&!SNAI1&ZEB1&TGFB&NANOG&p53 | !EZH2&!BMI1&E2F&Bcatenin&HNF4A&!HNF6&!NFkB&SNAI1&p53 | !EZH2&!BMI1&E2F&Bcatenin&HNF4A&!HNF6&NFkB&!SNAI1&!ZEB1&!SOX9&!NANOG&p53 | !EZH2&!BMI1&E2F&Bcatenin&HNF4A&!HNF6&NFkB&!SNAI1&!ZEB1&!SOX9&NANOG&!p53&p16 | !EZH2&!BMI1&E2F&Bcatenin&HNF4A&!HNF6&NFkB&!SNAI1&!ZEB1&!SOX9&NANOG&p53 | !EZH2&!BMI1&E2F&Bcatenin&HNF4A&!HNF6&NFkB&!SNAI1&!ZEB1&SOX9&p53 | !EZH2&!BMI1&E2F&Bcatenin&HNF4A&!HNF6&NFkB&!SNAI1&ZEB1&!OCT4&!p53&p16 | !EZH2&!BMI1&E2F&Bcatenin&HNF4A&!HNF6&NFkB&!SNAI1&ZEB1&!OCT4&p53 | !EZH2&!BMI1&E2F&Bcatenin&HNF4A&!HNF6&NFkB&!SNAI1&ZEB1&OCT4&!NANOG&p53 | !EZH2&!BMI1&E2F&Bcatenin&HNF4A&!HNF6&NFkB&!SNAI1&ZEB1&OCT4&NANOG&!p53&p16 | !EZH2&!BMI1&E2F&Bcatenin&HNF4A&!HNF6&NFkB&!SNAI1&ZEB1&OCT4&NANOG&p53 | !EZH2&!BMI1&E2F&Bcatenin&HNF4A&!HNF6&NFkB&SNAI1&!OCT4&!p53&p16 | !EZH2&!BMI1&E2F&Bcatenin&HNF4A&!HNF6&NFkB&SNAI1&!OCT4&p53 | !EZH2&!BMI1&E2F&Bcatenin&HNF4A&!HNF6&NFkB&SNAI1&OCT4&p53 | !EZH2&!BMI1&E2F&Bcatenin&HNF4A&HNF6&!NFkB&!SNAI1&!ZEB1 | !EZH2&!BMI1&E2F&Bcatenin&HNF4A&HNF6&!NFkB&!SNAI1&ZEB1&!TGFB | !EZH2&!BMI1&E2F&Bcatenin&HNF4A&HNF6&!NFkB&!SNAI1&ZEB1&TGFB&!p53&p16 | !EZH2&!BMI1&E2F&Bcatenin&HNF4A&HNF6&!NFkB&!SNAI1&ZEB1&TGFB&p53 | !EZH2&!BMI1&E2F&Bcatenin&HNF4A&HNF6&!NFkB&SNAI1&!TGFB | !EZH2&!BMI1&E2F&Bcatenin&HNF4A&HNF6&!NFkB&SNAI1&TGFB&!p53&p16 | !EZH2&!BMI1&E2F&Bcatenin&HNF4A&HNF6&!NFkB&SNAI1&TGFB&p53 | !EZH2&!BMI1&E2F&Bcatenin&HNF4A&HNF6&NFkB&!p53&p16 | !EZH2&!BMI1&E2F&Bcatenin&HNF4A&HNF6&NFkB&p53 | !EZH2&BMI1&!HNF4A&!HNF6&!NFkB&!SNAI1&!TGFB&!NANOG&p53 | !EZH2&BMI1&!HNF4A&!HNF6&!NFkB&!SNAI1&!TGFB&NANOG | !EZH2&BMI1&!HNF4A&!HNF6&!NFkB&!SNAI1&TGFB&!NANOG&p53 | !EZH2&BMI1&!HNF4A&!HNF6&!NFkB&!SNAI1&TGFB&NANOG&!p53&p16 | !EZH2&BMI1&!HNF4A&!HNF6&!NFkB&!SNAI1&TGFB&NANOG&p53 | !EZH2&BMI1&!HNF4A&!HNF6&!NFkB&SNAI1&p53 | !EZH2&BMI1&!HNF4A&!HNF6&NFkB&!SNAI1&!OCT4&!p53&p16 | !EZH2&BMI1&!HNF4A&!HNF6&NFkB&!SNAI1&!OCT4&p53 | !EZH2&BMI1&!HNF4A&!HNF6&NFkB&!SNAI1&OCT4&!NANOG&p53 | !EZH2&BMI1&!HNF4A&!HNF6&NFkB&!SNAI1&OCT4&NANOG&!p53&p16 | !EZH2&BMI1&!HNF4A&!HNF6&NFkB&!SNAI1&OCT4&NANOG&p53 | !EZH2&BMI1&!HNF4A&!HNF6&NFkB&SNAI1&!OCT4&!p53&p16 | !EZH2&BMI1&!HNF4A&!HNF6&NFkB&SNAI1&!OCT4&p53 | !EZH2&BMI1&!HNF4A&!HNF6&NFkB&SNAI1&OCT4&p53 | !EZH2&BMI1&!HNF4A&HNF6&!NFkB&!TGFB | !EZH2&BMI1&!HNF4A&HNF6&!NFkB&TGFB&!p53&p16 | !EZH2&BMI1&!HNF4A&HNF6&!NFkB&TGFB&p53 | !EZH2&BMI1&!HNF4A&HNF6&NFkB&!p53&p16 | !EZH2&BMI1&!HNF4A&HNF6&NFkB&p53 | !EZH2&BMI1&HNF4A&!HNF6&!NFkB&!SNAI1&!ZEB1&!TGFB&!SOX9&!NANOG&p53 | !EZH2&BMI1&HNF4A&!HNF6&!NFkB&!SNAI1&!ZEB1&!TGFB&!SOX9&NANOG | !EZH2&BMI1&HNF4A&!HNF6&!NFkB&!SNAI1&!ZEB1&!TGFB&SOX9&p53 | !EZH2&BMI1&HNF4A&!HNF6&!NFkB&!SNAI1&!ZEB1&TGFB&!SOX9&!NANOG&p53 | !EZH2&BMI1&HNF4A&!HNF6&!NFkB&!SNAI1&!ZEB1&TGFB&!SOX9&NANOG&!p53&p16 | !EZH2&BMI1&HNF4A&!HNF6&!NFkB&!SNAI1&!ZEB1&TGFB&!SOX9&NANOG&p53 | !EZH2&BMI1&HNF4A&!HNF6&!NFkB&!SNAI1&!ZEB1&TGFB&SOX9&p53 | !EZH2&BMI1&HNF4A&!HNF6&!NFkB&!SNAI1&ZEB1&!TGFB&!NANOG&p53 | !EZH2&BMI1&HNF4A&!HNF6&!NFkB&!SNAI1&ZEB1&!TGFB&NANOG | !EZH2&BMI1&HNF4A&!HNF6&!NFkB&!SNAI1&ZEB1&TGFB&!NANOG&p53 | !EZH2&BMI1&HNF4A&!HNF6&!NFkB&!SNAI1&ZEB1&TGFB&NANOG&!p53&p16 | !EZH2&BMI1&HNF4A&!HNF6&!NFkB&!SNAI1&ZEB1&TGFB&NANOG&p53 | !EZH2&BMI1&HNF4A&!HNF6&!NFkB&SNAI1&p53 | !EZH2&BMI1&HNF4A&!HNF6&NFkB&!SNAI1&!ZEB1&!SOX9&!NANOG&p53 | !EZH2&BMI1&HNF4A&!HNF6&NFkB&!SNAI1&!ZEB1&!SOX9&NANOG&!p53&p16 | !EZH2&BMI1&HNF4A&!HNF6&NFkB&!SNAI1&!ZEB1&!SOX9&NANOG&p53 | !EZH2&BMI1&HNF4A&!HNF6&NFkB&!SNAI1&!ZEB1&SOX9&p53 | !EZH2&BMI1&HNF4A&!HNF6&NFkB&!SNAI1&ZEB1&!OCT4&!p53&p16 | !EZH2&BMI1&HNF4A&!HNF6&NFkB&!SNAI1&ZEB1&!OCT4&p53 | !EZH2&BMI1&HNF4A&!HNF6&NFkB&!SNAI1&ZEB1&OCT4&!NANOG&p53 | !EZH2&BMI1&HNF4A&!HNF6&NFkB&!SNAI1&ZEB1&OCT4&NANOG&!p53&p16 | !EZH2&BMI1&HNF4A&!HNF6&NFkB&!SNAI1&ZEB1&OCT4&NANOG&p53 | !EZH2&BMI1&HNF4A&!HNF6&NFkB&SNAI1&!OCT4&!p53&p16 | !EZH2&BMI1&HNF4A&!HNF6&NFkB&SNAI1&!OCT4&p53 | !EZH2&BMI1&HNF4A&!HNF6&NFkB&SNAI1&OCT4&p53 | !EZH2&BMI1&HNF4A&HNF6&!NFkB&!TGFB | !EZH2&BMI1&HNF4A&HNF6&!NFkB&TGFB&!p53&p16 | !EZH2&BMI1&HNF4A&HNF6&!NFkB&TGFB&p53 | !EZH2&BMI1&HNF4A&HNF6&NFkB&!p53&p16 | !EZH2&BMI1&HNF4A&HNF6&NFkB&p53 | EZH2&!HNF4A&!HNF6&!NFkB&!SNAI1&!TGFB&!NANOG&p53 | EZH2&!HNF4A&!HNF6&!NFkB&!SNAI1&!TGFB&NANOG | EZH2&!HNF4A&!HNF6&!NFkB&!SNAI1&TGFB&!NANOG&p53 | EZH2&!HNF4A&!HNF6&!NFkB&!SNAI1&TGFB&NANOG&!p53&p16 | EZH2&!HNF4A&!HNF6&!NFkB&!SNAI1&TGFB&NANOG&p53 | EZH2&!HNF4A&!HNF6&!NFkB&SNAI1&p53 | EZH2&!HNF4A&!HNF6&NFkB&!SNAI1&!OCT4&!p53&p16 | EZH2&!HNF4A&!HNF6&NFkB&!SNAI1&!OCT4&p53 | EZH2&!HNF4A&!HNF6&NFkB&!SNAI1&OCT4&!NANOG&p53 | EZH2&!HNF4A&!HNF6&NFkB&!SNAI1&OCT4&NANOG&!p53&p16 | EZH2&!HNF4A&!HNF6&NFkB&!SNAI1&OCT4&NANOG&p53 | EZH2&!HNF4A&!HNF6&NFkB&SNAI1&!OCT4&!p53&p16 | EZH2&!HNF4A&!HNF6&NFkB&SNAI1&!OCT4&p53 | EZH2&!HNF4A&!HNF6&NFkB&SNAI1&OCT4&p53 | EZH2&!HNF4A&HNF6&!NFkB&!TGFB | EZH2&!HNF4A&HNF6&!NFkB&TGFB&!p53&p16 | EZH2&!HNF4A&HNF6&!NFkB&TGFB&p53 | EZH2&!HNF4A&HNF6&NFkB&!p53&p16 | EZH2&!HNF4A&HNF6&NFkB&p53 | EZH2&HNF4A&!HNF6&!NFkB&!SNAI1&!ZEB1&!TGFB&!SOX9&!NANOG&p53 | EZH2&HNF4A&!HNF6&!NFkB&!SNAI1&!ZEB1&!TGFB&!SOX9&NANOG | EZH2&HNF4A&!HNF6&!NFkB&!SNAI1&!ZEB1&!TGFB&SOX9&p53 | EZH2&HNF4A&!HNF6&!NFkB&!SNAI1&!ZEB1&TGFB&!SOX9&!NANOG&p53 | EZH2&HNF4A&!HNF6&!NFkB&!SNAI1&!ZEB1&TGFB&!SOX9&NANOG&!p53&p16 | EZH2&HNF4A&!HNF6&!NFkB&!SNAI1&!ZEB1&TGFB&!SOX9&NANOG&p53 | EZH2&HNF4A&!HNF6&!NFkB&!SNAI1&!ZEB1&TGFB&SOX9&p53 | EZH2&HNF4A&!HNF6&!NFkB&!SNAI1&ZEB1&!TGFB&!NANOG&p53 | EZH2&HNF4A&!HNF6&!NFkB&!SNAI1&ZEB1&!TGFB&NANOG | EZH2&HNF4A&!HNF6&!NFkB&!SNAI1&ZEB1&TGFB&!NANOG&p53 | EZH2&HNF4A&!HNF6&!NFkB&!SNAI1&ZEB1&TGFB&NANOG&!p53&p16 | EZH2&HNF4A&!HNF6&!NFkB&!SNAI1&ZEB1&TGFB&NANOG&p53 | EZH2&HNF4A&!HNF6&!NFkB&SNAI1&p53 | EZH2&HNF4A&!HNF6&NFkB&!SNAI1&!ZEB1&!SOX9&!NANOG&p53 | EZH2&HNF4A&!HNF6&NFkB&!SNAI1&!ZEB1&!SOX9&NANOG&!p53&p16 | EZH2&HNF4A&!HNF6&NFkB&!SNAI1&!ZEB1&!SOX9&NANOG&p53 | EZH2&HNF4A&!HNF6&NFkB&!SNAI1&!ZEB1&SOX9&p53 | EZH2&HNF4A&!HNF6&NFkB&!SNAI1&ZEB1&!OCT4&!p53&p16 | EZH2&HNF4A&!HNF6&NFkB&!SNAI1&ZEB1&!OCT4&p53 | EZH2&HNF4A&!HNF6&NFkB&!SNAI1&ZEB1&OCT4&!NANOG&p53 | EZH2&HNF4A&!HNF6&NFkB&!SNAI1&ZEB1&OCT4&NANOG&!p53&p16 | EZH2&HNF4A&!HNF6&NFkB&!SNAI1&ZEB1&OCT4&NANOG&p53 | EZH2&HNF4A&!HNF6&NFkB&SNAI1&!OCT4&!p53&p16 | EZH2&HNF4A&!HNF6&NFkB&SNAI1&!OCT4&p53 | EZH2&HNF4A&!HNF6&NFkB&SNAI1&OCT4&p53 | EZH2&HNF4A&HNF6&!NFkB&!TGFB | EZH2&HNF4A&HNF6&!NFkB&TGFB&!p53&p16 | EZH2&HNF4A&HNF6&!NFkB&TGFB&p53 | EZH2&HNF4A&HNF6&NFkB&!p53&p16 | EZH2&HNF4A&HNF6&NFkB&p53

# Scripts implemented in R

The following scripts are used to automatically calculate the attractors of the networks shown above, as well as their basins of attraction. These scripts should be saved in separate files with the extension ".R", and run in R. Remember that it is important to have the networks saved in a special directory called "networks".

## **Calculation of attractors**

# This script reads a network from a text file, calculates the attractors and

# and plots the results. The network is defined by the

# rules of the Boolean network and the attractors are calculated using the

# synchronous update method. The attractors are then

# plotted using the plotAttractors function from the BoolNet package.

# Load the BoolNet package

library(BoolNet)

#### ---- Network of T-CD4+ lymphocytes differentiation ---- ####

TCD4 <- loadNetwork(file = "networks/network_TCD4.bnet")

TCD4_attractors <- getAttractors(network = TCD4,

                                 genesOFF = c("IFNye", "IL21e", "TGFBe", "IL2e", "IL4e", "IL10e"),

                                 canonical =  TRUE,

                                 type = "synchronous",

                                 method = "exhaustive")

plotAttractors(attractorInfo = TCD4_attractors,

               title = "Differentiation of T-CD4+ lymphocytes",

               onColor = "black",

               offColor = "white",

               borderColor = "grey", eps = 0.1)

#### ---- Network of T-CD8+ lymphocytes differentiation ---- ####

TCD8 <- loadNetwork(file = "networks/network_TCD8.bnet")

TCD8_attractors <- getAttractors(network = TCD8,

                                 genesOFF = c("IL12s", "IL6s", "GATA3", "IFN1", "TGFB", "IL2s", "IL10s", "IL12", "PD1", "IL15s", "GFs", "Glucose", "FFAs", "Ceramide", "EtOH", "FasL"),

                                 canonical =  TRUE,

                                 type = "synchronous",

                                 method = "sat.exhaustive")

plotAttractors(attractorInfo = TCD8_attractors,

               title = "Differentiation of T CD8+ lymphocytes",

               onColor = "black",

               offColor = "white",

               borderColor = "grey", eps = 0.1)

#### ---- Red de macrofagos ---- ####

macrophages <- loadNetwork(file = "networks/network_macrophages.bnet")

macrophages_attractors <- getAttractors(network = macrophages,

                                 genesOFF = c("IFNyInput", "IFNBInput", "IL12Input", "GMCSF", "TNFInput", "Insulin", "CeramideInput", "LPS", "IL1BInput", "IL6Input", "IL10Input", "IL4Input", "TGFBInput"),

                                 canonical =  TRUE,

                                 type = "synchronous",

                                 method = "exhaustive")

plotAttractors(attractorInfo = macrophages_attractors,

               title = "Differentiation of macrofagues",

               onColor = "black",

               offColor = "white",

               borderColor = "grey", eps = 0.1)

#### ---- Network of hematopoietic system differentiation ---- ####

hematopoietic <- loadNetwork(file = "networks/network_HSCs.bnet")

## Case: Without oxygen

hematopoietic_attrs <- getAttractors(network = hematopoietic,

                                 genesOFF = c("Oxygen"),

                                 canonical =  TRUE,

                                 type = "synchronous",

                                 method = "exhaustive")

plotAttractors(attractorInfo = hematopoietic_attrs,

               title = "Differentiation of hematopoietic cells without oxygen",

               onColor = "black",

               offColor = "white",

               borderColor = "grey", eps = 0.1)

## Case: With oxygen

hematopoietic_attrs <- getAttractors(network = hematopoietic,

                                 genesON = c("Oxygen"),

                                 canonical =  TRUE,

                                 type = "synchronous",

                                 method = "exhaustive")

plotAttractors(attractorInfo = hematopoietic_attrs,

               title = "Differentiation of hematopoietic cells with oxygen",

               onColor = "black",

               offColor = "white",

               borderColor = "grey", eps = 0.1)

#### ---- Network of EMT in hepatocytes ---- ####

EMT <- loadNetwork(file = "networks/network_HCC.bnet")

EMT_attractors <- getAttractors(network = EMT,

                                 canonical =  TRUE,

                                 type = "synchronous",

                                 method = "exhaustive")

plotAttractors(attractorInfo = EMT,

               title = "Epithelial-to-mesenchymal transition in Hepatocytes",

               onColor = "black",

               offColor = "white",

               borderColor = "grey", eps = 0.1)

## **Calculation of basins of attraction**

# This script reads a set of Boolean networks from a folder and calculates

# the attractors and basins of attraction of each network. The results are

# saved in a text file.

#### Automatization ####

# Load the BoolNet package

library(BoolNet)

# Define the folder path where the networks are stonet

networks_directory <- "networks"

# We list all the files in the folder that have the extension .bnet and we store them in a vector

files <- list.files(path = networks_directory, pattern = "^red[0-9]{1,2}\\.bnet$", full.names = TRUE)

# We apply the loadNetwork function to each file in the vector to load the networks

# and store them in a list

networks <- lapply(files, loadNetwork)

# We define a function that calculates the attractors and basins of attraction of a network

# This function receives a network as an argument and returns a data frame with the attractors

basins_of_attraction <- function(net) {

  # Calculate attractors

  net_attractors <- getAttractors(net)

  ## -- Calculate the basin of attraction for each attractos -- ##

  # Create an empty data frame to store the results with the

  # columns Attractor and BasinSize

  results <- data.frame(

    Attractor = integer(),

    BasinSize = integer()

  )

  # For each attractor, calculate the basin of attraction and store the size

  # in the data frame

  for (i in 1:length(net_attractors$attractors)) {

    basin <- getBasinOfAttraction(net_attractors, i)

    results <- rbind(results, data.frame(

      Attractor = i,

      BasinSize = nrow(basin)

    ))

  }

  # Return the results

  return(results)

}

# We apply the function to each network in the list

all_nets_results <- lapply(networks, basins_of_attraction)

# We save the results in a text file

output_file <- "results_basins_of_attraction.txt"

con <- file(output_file, "w")

# We write the results of each network in the file with tabulation as separator

for (i in seq_along(all_nets_results)) {

  cat("results of the nets:", files[i], "\n", file = con, sep = "\t")

  write.table(all_nets_results[[i]], file = con, sep = "\t", row.names = FALSE, col.names = TRUE, quote = FALSE)

  cat("\n", file = con)  # We add a blank line between networks

}

close(con)

# We print the path of the file where the results were saved

cat("results saved in:", output_file, "\n")

## **Asynchronous simulation**

# This script reads a network from a text file, simulates it with asynchronous

# update, and compares two different update schemes. The network is defined by

# the rules of the Boolean network and the update schemes are defined by the

# periods of update for each gene.

# Load the BoolNet package

library(BoolNet)

# Definition of the rules of the Boolean network

rules <- "

targets, factors

x1, x2

x2, x1

"

# For reproducibility, we save the rules in a temporary file and load the network

temp_file <- tempfile(fileext = ".bnet")

writeLines(rules, temp_file)

# Load the network from the temporary file using the loadNetwork function

network <- loadNetwork(temp_file)

print(network) # We print the network to verify the rules

# Define a function to simulate the network with asynchronous update

# The function takes the network and the periods of update for each gene

# as arguments

asynchronous_update <- function(network, periods) {

  # Extract the genes from the network object

  genes <- network$genes

  # Define the maximum number of steps for the simulation

  # until reaching a stable state

  steps <- max(periods) * 5

  # Select a random initial state for the network

  initial_state <- generateState(network, specs = c(x1 = 0, x2 = 1))

  # Set the initial state as the current state

  current_state <- initial_state

  # Print the initial state to verify the simulation

  cat("Initial state:", current_state, "\n\n")

  # Simulate the network by updating the genes asynchronously based on the periods

  # defined for each gene

  for (i in 1:steps) {

    cat("Step:", i, "\n")

    if (i %% periods["x1"] == 0) {

      # We update the state of gene x1 based on the state transition function

      # Note: The update is asynchronous, so the order of updates may vary

      current_state["x1"] <- stateTransition(network, current_state)["x1"]

      cat("x1 updated ->", current_state["x1"], "\n")

    }

    if (i %% periods["x2"] == 0) {

      # We update the state of gene x2 based on the state transition function

      # Note: The update is asynchronous, so the order of updates may vary

      current_state["x2"] <- stateTransition(network, current_state)["x2"]

      cat("x2 updated ->", current_state["x2"], "\n")

    }

    # We print the current state after each update

    cat("Current state:", current_state, "\n\n")

  }

}

# Simulate the first scheme: x1 every 2 steps, x2 every 3 steps

cat("### First update scheme ###\n")

first_period <- c(x1 = 2, x2 = 3)

asynchronous_update(network, first_period)

# Simulate the second scheme: x1 every 3 steps, x2 every 2 steps

cat("### Second update scheme ###\n")

second_period <- c(x1 = 3, x2 = 2)

asynchronous_update(network, second_period)

# Delete the temporary file

unlink(temp_file)

# Scripts implemented in C#

The following scripts are written in C# and must be saved as individual files to run in Visual Studio Code or Visual Studio.

## **Implementation of Theorem 4: Differentiation of CD4+ T cells**

// See https://aka.ms/new-console-template for more information

//Step 1: Defining state space

using System.Diagnostics;

Stopwatch stopWatch = new Stopwatch();

stopWatch.Start();

List<string> intialConditions = new List<string>();

List<string> InputsZ = new List<string>();

List<string> Output = new List<string>();

List<string> OutputsZ = new List<string>();

List<string> FixedPoints = new List<string>();

//StreamWriter outcomeModel = new StreamWriter(filepath);

int numberNodes = 12;

int maxN = (int)Math.Pow(2, numberNodes);

int n = 0;

//Step 2: Creating all initial states of the network

for (int i = 0; i < maxN; i++)

{

n = i;

string bin = Convert.ToString(n, 2).PadLeft(numberNodes, '0');

//Console.WriteLine(bin);

intialConditions.Add(bin);

string z1 = Convert.ToString(n);

InputsZ.Add(z1);

//Console.WriteLine(z1);

}

Console.WriteLine("Next step:");

//Step 3: Evaluating the network

for (int h = 0; h < maxN; h++)

{

string sentence = intialConditions[h];

char[] charArr = sentence.ToCharArray();

int[] xS = Array.ConvertAll(charArr, c => (int)Char.GetNumericValue(c));

int IFNGe = 0;

int IL12e = 0;

int IL2e = 0;

int IL4e = 0;

int TGFBe = 0;

int IL10e = 0;

int IL6e = 0;

int IL27e = 0;

int INSULIN = 0;

int Ceramide = 0;

List<int> TBET = new List<int>();

List<int> IFNG = new List<int>();

List<int> GATA3 = new List<int>();

List<int> IL2 = new List<int>();

List<int> IL4 = new List<int>();

List<int> RORGT = new List<int>();

List<int> IL6 = new List<int>();

List<int> FOXP3 = new List<int>();

List<int> TGFB = new List<int>();

List<int> IL10 = new List<int>();

List<int> PU1 = new List<int>();

List<int> IL9 = new List<int>();

TBET.Add(xS[0]);

IFNG.Add(xS[1]);

GATA3.Add(xS[2]);

IL2.Add(xS[3]);

IL4.Add(xS[4]);

RORGT.Add(xS[5]);

IL6.Add(xS[6]);

FOXP3.Add(xS[7]);

TGFB.Add(xS[8]);

IL10.Add(xS[9]);

PU1.Add(xS[10]);

IL9.Add(xS[11]);

//Logic rule for TBET

if (((IFNG[0] == 1 || (

IL12e == 1 && !(

IL6[0] == 1 ||

IL4[0] == 1 ||

IL10[0] == 1))) ||

TBET[0] == 1) && !(

IL4[0] == 1 ||

GATA3[0] == 1 ||

IL6[0] == 1)) { TBET[0] = 1; }

else { TBET[0] = 0; }

//Logic rule for IFNG

if ((IFNGe == 1 || ((

IFNG[0] == 1 ||

TBET[0] == 1) && !(

GATA3[0] == 1 ||

TGFB[0] == 1))) && !(

IL6[0] == 1 ||

IL4[0] == 1 ||

IL10[0] == 1 || Ceramide == 1)) { IFNG[0] = 1; }

else { IFNG[0] = 0; }

//Logic rule for GATA3

if (((IL2[0] == 1 &&

IL4[0] == 1) ||

GATA3[0] == 1) && !(

TBET[0] == 1 ||

TGFB[0] == 1 ||

IL6[0] == 1 ||

IFNG[0] == 1 ||

PU1[0] == 1)) { GATA3[0] = 1; }

else { GATA3[0] = 0; }

//Logic rule for IL2

if ((IL2e == 1 || (

IL2[0] == 1 &&

FOXP3[0] == 0)) && !(

IFNG[0] == 1 ||

IL6[0] == 1 || (

IL10[0] == 1 &&

FOXP3[0] == 0))) { IL2[0] = 1; }

else { IL2[0] = 0; }

//Logic rule for IL4

if ((IL4e == 1 || (

GATA3[0] == 1 && (

IL2[0] == 1 ||

IL4[0] == 1) &&

TBET[0] == 0)) && !(

IFNG[0] == 1 ||

IL6[0] == 1 ||

PU1[0] == 1)) { IL4[0] = 1; }

else { IL4[0] = 0; }

//Logic rule for RORGT

if ((IL6[0] == 1 &&

TGFB[0] == 1) && !(

TBET[0] == 1 ||

FOXP3[0] == 1 ||

GATA3[0] == 1)) { RORGT[0] = 1; }

else { RORGT[0] = 0; }

//Logic rule for IL6

if ((IL6e == 1 ||

IL6[0] == 1 ||

RORGT[0] == 1) && !(

IFNG[0] == 1 ||

IL4[0] == 1 ||

IL10[0] == 1 ||

IL2[0] == 1)) { IL6[0] = 1; }

else { IL6[0] = 0; }

//Logic rule for FOXP3

if ((IL2[0] == 1 && (Ceramide == 1 ||

TGFB[0] == 1 ||

FOXP3[0] == 1)) && !(

IL6[0] == 1 ||

RORGT[0] == 1 ||

PU1[0] == 1)) { FOXP3[0] = 1; }

else { FOXP3[0] = 0; }

//Logic rule for TGFB

if (TGFBe == 1 || ((

TGFB[0] == 1 ||

FOXP3[0] == 1) &&

IL6[0] == 0)) { TGFB[0] = 1; }

else { TGFB[0] = 0; }

//Logic rule for IL10

if (IL10e == 1 || (

IL10[0] == 1 && (

IFNG[0] == 1 ||

IL6[0] == 1 ||

TGFB[0] == 1 ||

GATA3[0] == 1 ||

IL27e == 1) &&

!(INSULIN == 1 || Ceramide == 1))) { IL10[0] = 1; }

else { IL10[0] = 0; }

//Logic rule for PU1

if ((IL6[0] == 1 ||

TGFB[0] == 1 ||

PU1[0] == 1) &&

(IL2[0] == 0)) { PU1[0] = 1; }

else { PU1[0] = 0; }

//Logic rule for IL9

if (((IL4[0] == 1 ||

IL4e == 1) &&

PU1[0] == 1) &&

TBET[0] == 0) { IL9[0] = 1; }

else { IL9[0] = 0; }

var node1 = TBET.Select(x => Convert.ToString(x)).ToList();

var node2 = IFNG.Select(x => Convert.ToString(x)).ToList();

var node3 = GATA3.Select(x => Convert.ToString(x)).ToList();

var node4 = IL2.Select(x => Convert.ToString(x)).ToList();

var node5 = IL4.Select(x => Convert.ToString(x)).ToList();

var node6 = RORGT.Select(x => Convert.ToString(x)).ToList();

var node7 = IL6.Select(x => Convert.ToString(x)).ToList();

var node8 = FOXP3.Select(x => Convert.ToString(x)).ToList();

var node9 = TGFB.Select(x => Convert.ToString(x)).ToList();

var node10 = IL10.Select(x => Convert.ToString(x)).ToList();

var node11 = PU1.Select(x => Convert.ToString(x)).ToList();

var node12 = IL9.Select(x => Convert.ToString(x)).ToList();

List<string> Nodes = new List<string>();

for (int w = 0; w < 1; w++)

{

Nodes.Add(node1[w] +

node2[w] +

node3[w] +

node4[w] +

node5[w] +

node6[w] +

node7[w] +

node8[w] +

node9[w] +

node10[w] +

node11[w] +

node12[w]);

Output.Add(node1[w] +

node2[w] +

node3[w] +

node4[w] +

node5[w] +

node6[w] +

node7[w] +

node8[w] +

node9[w] +

node10[w] +

node11[w] +

node12[w]);

}

}

//Step 4: Finding all fixed-point attractors

for (int k = 0; k < intialConditions.Count; k++)

{

if (intialConditions[k] == Output[k])

{

FixedPoints.Add(intialConditions[k]);

}

}

Console.WriteLine("Fixed-points in:");

foreach (string s in FixedPoints)

Console.WriteLine(s);

stopWatch.Stop();

// Get the elapsed time as a TimeSpan value.

TimeSpan ts = stopWatch.Elapsed;

// Format and display the TimeSpan value.

string elapsedTime = String.Format("{0:00}:{1:00}:{2:00}.{3:00}",

ts.Hours, ts.Minutes, ts.Seconds,

ts.Milliseconds / 10);

Console.WriteLine("RunTime " + elapsedTime);

## **Implementation of Theorem 4: Differentiation of CD8+ T cells**

//Step 1: Defining state space

using System.Diagnostics;

Stopwatch stopWatch = new Stopwatch();

stopWatch.Start();

List<string> intialConditions = new List<string>();

List<string> InputsZ = new List<string>();

List<string> Output = new List<string>();

List<string> OutputsZ = new List<string>();

List<string> FixedPoints = new List<string>();

//StreamWriter outcomeModel = new StreamWriter(filepath);

int numberNodes = 18;

int maxN = (int)Math.Pow(2, numberNodes);

int n = 0;

//Step 2: Creating all initial states of the network

for (int i = 0; i < maxN; i++)

{

n = i;

string bin = Convert.ToString(n, 2).PadLeft(numberNodes, '0');

//Console.WriteLine(bin);

intialConditions.Add(bin);

string z1 = Convert.ToString(n);

InputsZ.Add(z1);

//Console.WriteLine(z1);

}

Console.WriteLine("Next step:");

//Step 3: Evaluating the network

for (int h = 0; h < maxN; h++)

{

string sentence = intialConditions[h];

char[] charArr = sentence.ToCharArray();

int[] xS = Array.ConvertAll(charArr, c => (int)Char.GetNumericValue(c));

int IL6e = 0;

int IFNGe = 0;

int IL2e = 0;

int IL4e = 0;

int IL10e = 0;

int TGFB = 0;

int IL12e = 0;

int IFNI = 0;

int aa = 0;

int GFs = 0;

int Glucose = 0;

int PD1 = 0;

int FFAs = 0;

int IL15e = 0;

int Ceramide = 0;

int EtOH = 0;

int FasL = 0;

List<int> TBET = new List<int>();

List<int> IFNG = new List<int>();

List<int> GATA3 = new List<int>();

List<int> IL4 = new List<int>();

List<int> RORGT = new List<int>();

List<int> IL10 = new List<int>();

List<int> FOXP3 = new List<int>();

List<int> FOXO1 = new List<int>();

List<int> EOMES = new List<int>();

List<int> mTORC1 = new List<int>();

List<int> mTORC2 = new List<int>();

List<int> ROS = new List<int>();

List<int> AKT = new List<int>();

List<int> GLUT1 = new List<int>();

List<int> GranzymeB = new List<int>();

List<int> SOD = new List<int>();

List<int> BCL2 = new List<int>();

List<int> CASP3 = new List<int>();

TBET.Add(xS[0]);

IFNG.Add(xS[1]);

GATA3.Add(xS[2]);

IL4.Add(xS[3]);

RORGT.Add(xS[4]);

IL10.Add(xS[5]);

FOXP3.Add(xS[6]);

FOXO1.Add(xS[7]);

EOMES.Add(xS[8]);

mTORC1.Add(xS[9]);

mTORC2.Add(xS[10]);

ROS.Add(xS[11]);

AKT.Add(xS[12]);

GLUT1.Add(xS[13]);

GranzymeB.Add(xS[14]);

SOD.Add(xS[15]);

BCL2.Add(xS[16]);

CASP3.Add(xS[17]);

//Logic rule for TBET

if (((IFNG[0] == 1 || IFNI == 1 || (

IL12e == 1 && !(

IL6e == 1 ||

IL4[0] == 1 ||

IL10[0] == 1))) ||

TBET[0] == 1) && !(

IL4[0] == 1 ||

GATA3[0] == 1 ||

IL6e == 1 ||

FOXO1[0] == 1)) { TBET[0] = 1; }

else { TBET[0] = 0; }

//Logic rule for IFNG

if ((IFNGe == 1 || IFNI == 1 || ((

IFNG[0] == 1 ||

TBET[0] == 1 ||

EOMES[0] == 1) &&

mTORC1[0] == 1 && !(

GATA3[0] == 1 ||

TGFB == 1))) && !(

IL6e == 1 ||

IL4[0] == 1 ||

IL10[0] == 1)) { IFNG[0] = 1; }

else { IFNG[0] = 0; }

//Logic rule for GATA3

if (((IL2e == 1 &&

IL4[0] == 1) ||

EOMES[0] == 1 ||

GATA3[0] == 1) && !(

TBET[0] == 1 ||

TGFB == 1 ||

IL6e == 1 ||

IFNG[0] == 1)) { GATA3[0] = 1; }

else { GATA3[0] = 0; }

//Logic rule for IL4

if ((IL4e == 1 || (

GATA3[0] == 1 && (

IL2e == 1 ||

IL4[0] == 1) &&

TBET[0] == 0)) && !(

IFNG[0] == 1 ||

IL6e == 1)) { IL4[0] = 1; }

else { IL4[0] = 0; }

//Logic rule for RORGT

if ((IL6e == 1 &&

TGFB == 1) && !(

TBET[0] == 1 ||

FOXP3[0] == 1 ||

GATA3[0] == 1 ||

FOXO1[0] == 1)) { RORGT[0] = 1; }

else { RORGT[0] = 0; }

//Logic rule for IL10

if ((IL10e == 1 ||

EOMES[0] == 1 || (

IL10[0] == 1 && (

IFNG[0] == 1 ||

IL6e == 1 ||

TGFB == 1 ||

GATA3[0] == 1))) &&

mTORC1[0] == 1) { IL10[0] = 1; }

else { IL10[0] = 0; }

//Logic rule for FOXP3

if (((IL2e == 1 ||

IL12e == 1) && (

TGFB == 1 ||

FOXP3[0] == 1 ||

IL4[0] == 1 || (

FOXO1[0] == 1))) && !(

IL6e == 1 ||

RORGT[0] == 1)) { FOXP3[0] = 1; }

else { FOXP3[0] = 0; }

//Logic rule for FOXO1

if ((ROS[0] == 1 ||

FOXO1[0] == 1) && !(

mTORC1[0] == 1 ||

mTORC2[0] == 1)) { FOXO1[0] = 1; }

else { FOXO1[0] = 0; }

//Logic rule for EOMES

if ((ROS[0] == 1 ||

EOMES[0] == 1 ||

IFNI == 1 ||

FOXO1[0] == 1) &&

mTORC2[0] == 0) { EOMES[0] = 1; }

else { EOMES[0] = 0; }

//Logic rule for mTORC1

if ((aa == 1 ||

AKT[0] == 1 ||

ROS[0] == 1 ||

IL12e == 1 ||

IL2e == 1) && !(

mTORC2[0] == 1 ||

PD1 == 1 ||

IL15e == 1)) { mTORC1[0] = 1; }

else { mTORC1[0] = 0; }

//Logic rule for mTORC2

if ((GFs == 1 ||

ROS[0] == 1) &&

mTORC1[0] == 0) { mTORC2[0] = 1; }

else { mTORC2[0] = 0; }

//Logic rule for ROS

if ((Glucose == 1 ||

FFAs == 1 ||

Ceramide == 1 ||

EtOH == 1) &&

SOD[0] == 0) { ROS[0] = 1; }

else { ROS[0] = 0; }

//Logic rule for AKT

if ((IFNG[0] == 1 ||

IL4[0] == 1 ||

IL10[0] == 1 ||

mTORC2[0] == 1)) { AKT[0] = 1; }

else { AKT[0] = 0; }

//Logic rule for GLUT1

if (AKT[0] == 1 &&

EOMES[0] == 1 &&

FOXO1[0] == 0) { GLUT1[0] = 1; }

else { GLUT1[0] = 0; }

//Logic rule for GranzymeB

if (TBET[0] == 1 && !(

GATA3[0] == 1 ||

RORGT[0] == 1 ||

FOXP3[0] == 1 ||

FOXO1[0] == 1)) { GranzymeB[0] = 1; }

else { GranzymeB[0] = 0; }

//Logic rule for SOD

if (ROS[0] == 1 && (

FOXO1[0] == 1) &&

Ceramide == 0) { SOD[0] = 1; }

else { SOD[0] = 0; }

//Logic rule for BCL2

if ((FOXO1[0] == 1) &&

ROS[0] == 0) { BCL2[0] = 1; }

else { BCL2[0] = 0; }

//Logic rule for CASP3

if (FasL == 1 && (

ROS[0] == 1 ||

CASP3[0] == 1) &&

BCL2[0] == 0) { CASP3[0] = 1; }

else { CASP3[0] = 0; }

var node1 = TBET.Select(x => Convert.ToString(x)).ToList();

var node2 = IFNG.Select(x => Convert.ToString(x)).ToList();

var node3 = GATA3.Select(x => Convert.ToString(x)).ToList();

var node4 = IL4.Select(x => Convert.ToString(x)).ToList();

var node5 = RORGT.Select(x => Convert.ToString(x)).ToList();

var node6 = IL10.Select(x => Convert.ToString(x)).ToList();

var node7 = FOXP3.Select(x => Convert.ToString(x)).ToList();

var node8 = FOXO1.Select(x => Convert.ToString(x)).ToList();

var node9 = EOMES.Select(x => Convert.ToString(x)).ToList();

var node10 = mTORC1.Select(x => Convert.ToString(x)).ToList();

var node11 = mTORC2.Select(x => Convert.ToString(x)).ToList();

var node12 = ROS.Select(x => Convert.ToString(x)).ToList();

var node13 = AKT.Select(x => Convert.ToString(x)).ToList();

var node14 = GLUT1.Select(x => Convert.ToString(x)).ToList();

var node15 = GranzymeB.Select(x => Convert.ToString(x)).ToList();

var node16 = SOD.Select(x => Convert.ToString(x)).ToList();

var node17 = BCL2.Select(x => Convert.ToString(x)).ToList();

var node18 = CASP3.Select(x => Convert.ToString(x)).ToList();

List<string> Nodes = new List<string>();

for (int w = 0; w < 1; w++)

{

Nodes.Add(node1[w] +

node2[w] +

node3[w] +

node4[w] +

node5[w] +

node6[w] +

node7[w] +

node8[w] +

node9[w] +

node10[w] +

node11[w] +

node12[w] +

node13[w] +

node14[w] +

node15[w] +

node16[w] +

node17[w] +

node18[w]);

Output.Add(node1[w] +

node2[w] +

node3[w] +

node4[w] +

node5[w] +

node6[w] +

node7[w] +

node8[w] +

node9[w] +

node10[w] +

node11[w] +

node12[w] +

node13[w] +

node14[w] +

node15[w] +

node16[w] +

node17[w] +

node18[w]);

}

}

//Step 4: Finding all fixed-point attractors

for (int k = 0; k < intialConditions.Count; k++)

{

if (intialConditions[k] == Output[k])

{

FixedPoints.Add(intialConditions[k]);

}

}

Console.WriteLine("Fixed-points in:");

foreach (string s in FixedPoints)

Console.WriteLine(s);

stopWatch.Stop();

// Get the elapsed time as a TimeSpan value.

TimeSpan ts = stopWatch.Elapsed;

// Format and display the TimeSpan value.

string elapsedTime = String.Format("{0:00}:{1:00}:{2:00}.{3:00}",

ts.Hours, ts.Minutes, ts.Seconds,

ts.Milliseconds / 10);

Console.WriteLine("RunTime " + elapsedTime);

## **Implementation of Theorem 4: Differentiation of HSCs**

//Step 1: Defining state space

using System.Diagnostics;

Stopwatch stopWatch = new Stopwatch();

stopWatch.Start();

List<string> intialConditions = new List<string>();

List<string> InputsZ = new List<string>();

List<string> Output = new List<string>();

List<string> OutputsZ = new List<string>();

List<string> FixedPoints = new List<string>();

//StreamWriter outcomeModel = new StreamWriter(filepath);

int numberNodes = 20;

int maxN = (int)Math.Pow(2, numberNodes);

int n = 0;

//Step 2: Creating all initial states of the network

for (int i = 0; i < maxN; i++)

{

n = i;

string bin = Convert.ToString(n, 2).PadLeft(numberNodes, '0');

//Console.WriteLine(bin);

intialConditions.Add(bin);

string z1 = Convert.ToString(n);

InputsZ.Add(z1);

//Console.WriteLine(z1);

}

Console.WriteLine("Next step:");

//Step 3: Evaluating the network

for (int h = 0; h < maxN; h++)

{

string sentence = intialConditions[h];

char[] charArr = sentence.ToCharArray();

int[] xS = Array.ConvertAll(charArr, c => (int)Char.GetNumericValue(c));

int oxigeno = 01;

List<int> runx1 = new List<int>();

List<int> meis1 = new List<int>();

List<int> hif1 = new List<int>();

List<int> foxo3 = new List<int>();

List<int> p53 = new List<int>();

List<int> gata2 = new List<int>();

List<int> gata1 = new List<int>();

List<int> pu1 = new List<int>();

List<int> cebpa = new List<int>();

List<int> ikzf1 = new List<int>();

List<int> gfi1 = new List<int>();

List<int> mef2c = new List<int>();

List<int> mtor = new List<int>();

List<int> ampk = new List<int>();

List<int> akt = new List<int>();

List<int> h2o2 = new List<int>();

List<int> o2 = new List<int>();

List<int> sod = new List<int>();

List<int> antiox = new List<int>();

List<int> oxphos = new List<int>();

runx1.Add(xS[0]);

meis1.Add(xS[1]);

hif1.Add(xS[2]);

foxo3.Add(xS[3]);

p53.Add(xS[4]);

gata2.Add(xS[5]);

gata1.Add(xS[6]);

pu1.Add(xS[7]);

cebpa.Add(xS[8]);

ikzf1.Add(xS[9]);

gfi1.Add(xS[10]);

mef2c.Add(xS[11]);

mtor.Add(xS[12]);

ampk.Add(xS[13]);

akt.Add(xS[14]);

h2o2.Add(xS[15]);

o2.Add(xS[16]);

sod.Add(xS[17]);

antiox.Add(xS[18]);

oxphos.Add(xS[19]);

//Logic rule for runx1

if ((pu1[0] == 1 || gata2[0] == 1 || runx1[0] == 1) && !(ikzf1[0] == 1/*|| mef2c[0] == 1*/)) { runx1[0] = 1; }

else { runx1[0] = 0; }

//Logic rule for meis1

if ((meis1[0] == 1 || runx1[0] == 1 || pu1[0] == 1) && gfi1[0] == 0) { meis1[0] = 1; }

else { meis1[0] = 0; }

//Logic rule for hif1

if ((oxigeno == 0 && o2[0] == 1 && !(p53[0] == 1 || foxo3[0] == 1 || ampk[0] == 1)) || (oxigeno == 0 && meis1[0] == 1)) { hif1[0] = 1; }

else { hif1[0] = 0; }

//Logic rule for foxo3

if (((ampk[0] == 1 && (hif1[0] == 1 || p53[0] == 1)) && !(akt[0] == 1 || cebpa[0] == 1))) { foxo3[0] = 1; }

else { foxo3[0] = 0; }

//Logic rule for p53

if (hif1[0] == 1 && !(akt[0] == 1 || gata1[0] == 1 || pu1[0] == 1)) { p53[0] = 1; }

else { p53[0] = 0; }

//Logic rule for gata2

if ((gata2[0] == 1 || p53[0] == 1) && !(gata1[0] == 1 || pu1[0] == 1 || gfi1[0] == 1)) { gata2[0] = 1; }

else { gata2[0] = 0; }

//Logic rule for gata1

if ((gata1[0] == 1 || gata2[0] == 1 || akt[0] == 1 || runx1[0] == 1) && !(pu1[0] == 1 || p53[0] == 1 || ikzf1[0] == 1)) { gata1[0] = 1; }

else { gata1[0] = 0; }

//Logic rule for pu1

if ((pu1[0] == 1 || runx1[0] == 1 || (cebpa[0] == 1 && ikzf1[0] == 1)) && !(gata1[0] == 1 || gata2[0] == 1 || gfi1[0] == 1)) { pu1[0] = 1; }

else { pu1[0] = 0; }

//Logic rule for cebpa

if ((pu1[0] == 1 && runx1[0] == 1) && mef2c[0] == 0) { cebpa[0] = 1; }

else { cebpa[0] = 0; }

//Logic rule for ikzf1

if ((mef2c[0] == 1 || ikzf1[0] == 1 || runx1[0] == 1) && !(cebpa[0] == 1 || pu1[0] == 1 || gata1[0] == 1)) { ikzf1[0] = 1; }

else { ikzf1[0] = 0; }

//Logic rule for gfi1

if ((ikzf1[0] == 1 || cebpa[0] == 1) && !(pu1[0] == 1 || p53[0] == 1)) { gfi1[0] = 1; }

else { gfi1[0] = 0; }

//Logic rule for mef2c

if (pu1[0] == 1 && cebpa[0] == 0) { mef2c[0] = 1; }

else { mef2c[0] = 0; }

//Logic rule for mtor

if (akt[0] == 1 && (ampk[0] == 0 && p53[0] == 0)) { mtor[0] = 1; }

else { mtor[0] = 0; }

//Logic rule for ampk

if ((akt[0] == 0 || oxphos[0] == 0 || hif1[0] == 0) && (p53[0] == 1)) { ampk[0] = 1; }

else { ampk[0] = 0; }

//Logic rule for akt

if ((h2o2[0] == 1) || !(p53[0] == 1 || foxo3[0] == 1 /*|| gata2[0] == 1*/)) { akt[0] = 1; }

else { akt[0] = 0; }

//Logic rule for h2o2

if ((sod[0] == 1 && o2[0] == 1 & antiox[0] == 0) | ((gata1[0] == 1 || hif1[0] == 1 || pu1[0] == 1) && antiox[0] == 0) || (oxphos[0] == 1 && antiox[0] == 0)) { h2o2[0] = 1; }

else { h2o2[0] = 0; }

//Logic rule for o2

if (sod[0] == 0 || oxphos[0] == 1) { o2[0] = 1; }

else { o2[0] = 0; }

//Logic rule for sod

if (p53[0] == 1 || foxo3[0] == 1) { sod[0] = 1; }

else { sod[0] = 0; }

//Logic rule for antiox

if (p53[0] == 1 || foxo3[0] == 1) { antiox[0] = 1; }

else { antiox[0] = 0; }

//Logic rule for oxphos

if ((mtor[0] == 1 || akt[0] == 1) && !(foxo3[0] == 1 || hif1[0] == 1)) { oxphos[0] = 1; }

else { oxphos[0] = 0; }

var node1 = runx1.Select(x => Convert.ToString(x)).ToList();

var node2 = meis1.Select(x => Convert.ToString(x)).ToList();

var node3 = hif1.Select(x => Convert.ToString(x)).ToList();

var node4 = foxo3.Select(x => Convert.ToString(x)).ToList();

var node5 = p53.Select(x => Convert.ToString(x)).ToList();

var node6 = gata2.Select(x => Convert.ToString(x)).ToList();

var node7 = gata1.Select(x => Convert.ToString(x)).ToList();

var node8 = pu1.Select(x => Convert.ToString(x)).ToList();

var node9 = cebpa.Select(x => Convert.ToString(x)).ToList();

var node10 = ikzf1.Select(x => Convert.ToString(x)).ToList();

var node11 = gfi1.Select(x => Convert.ToString(x)).ToList();

var node12 = mef2c.Select(x => Convert.ToString(x)).ToList();

var node13 = mtor.Select(x => Convert.ToString(x)).ToList();

var node14 = ampk.Select(x => Convert.ToString(x)).ToList();

var node15 = akt.Select(x => Convert.ToString(x)).ToList();

var node16 = h2o2.Select(x => Convert.ToString(x)).ToList();

var node17 = o2.Select(x => Convert.ToString(x)).ToList();

var node18 = sod.Select(x => Convert.ToString(x)).ToList();

var node19 = antiox.Select(x => Convert.ToString(x)).ToList();

var node20 = oxphos.Select(x => Convert.ToString(x)).ToList();

List<string> Nodes = new List<string>();

for (int w = 0; w < 1; w++)

{

Nodes.Add(node1[w] +

node2[w] +

node3[w] +

node4[w] +

node5[w] +

node6[w] +

node7[w] +

node8[w] +

node9[w] +

node10[w] +

node11[w] +

node12[w] +

node13[w] +

node14[w] +

node15[w] +

node16[w] +

node17[w] +

node18[w] +

node19[w] +

node20[w]);

Output.Add(node1[w] +

node2[w] +

node3[w] +

node4[w] +

node5[w] +

node6[w] +

node7[w] +

node8[w] +

node9[w] +

node10[w] +

node11[w] +

node12[w] +

node13[w] +

node14[w] +

node15[w] +

node16[w] +

node17[w] +

node18[w] +

node19[w] +

node20[w]);

}

}

//Step 4: Finding all fixed-point attractors

for (int k = 0; k < intialConditions.Count; k++)

{

if (intialConditions[k] == Output[k])

{

FixedPoints.Add(intialConditions[k]);

}

}

Console.WriteLine("Fixed-points in:");

foreach (string s in FixedPoints)

Console.WriteLine(s);

stopWatch.Stop();

// Get the elapsed time as a TimeSpan value.

TimeSpan ts = stopWatch.Elapsed;

// Format and display the TimeSpan value.

string elapsedTime = String.Format("{0:00}:{1:00}:{2:00}.{3:00}",

ts.Hours, ts.Minutes, ts.Seconds,

ts.Milliseconds / 10);

Console.WriteLine("RunTime " + elapsedTime);

## **Implementation of Theorem 4: Differentiation of Macrophages**

//Step 1: Defining state space

using System.Diagnostics;

Stopwatch stopWatch = new Stopwatch();

stopWatch.Start();

List<string> intialConditions = new List<string>();

List<string> InputsZ = new List<string>();

List<string> Output = new List<string>();

List<string> OutputsZ = new List<string>();

List<string> FixedPoints = new List<string>();

//StreamWriter outcomeModel = new StreamWriter(filepath);

int numberNodes = 15;

int maxN = (int)Math.Pow(2, numberNodes);

int n = 0;

//Step 2: Creating all initial states of the network

for (int i = 0; i < maxN; i++)

{

n = i;

string bin = Convert.ToString(n, 2).PadLeft(numberNodes, '0');

//Console.WriteLine(bin);

intialConditions.Add(bin);

string z1 = Convert.ToString(n);

InputsZ.Add(z1);

//Console.WriteLine(z1);

}

Console.WriteLine("Next step:");

//Step 3: Evaluating the network

for (int h = 0; h < maxN; h++)

{

string sentence = intialConditions[h];

char[] charArr = sentence.ToCharArray();

int[] xS = Array.ConvertAll(charArr, c => (int)Char.GetNumericValue(c));

// Cellular inputs

int IFNGe = 0;

int IL12e = 0;

int IFNBe = 0;

int IL4e = 0;

int TGFBe = 0;

int IL10e = 0;

int IL6e = 0;

int LPS = 0;

int TNFe = 0;

int IL1Be = 0;

int GMCSF = 0;

int Ceramide = 0;

int Insulin = 0;

List<int> IFNG = new List<int>();

List<int> IFNB = new List<int>();

List<int> IL12 = new List<int>();

List<int> TBET = new List<int>();

List<int> INOS = new List<int>();

List<int> TNF = new List<int>();

List<int> TLR4 = new List<int>();

List<int> IL1B = new List<int>();

List<int> IL6 = new List<int>();

List<int> IL10 = new List<int>();

List<int> IL4 = new List<int>();

List<int> IRF4 = new List<int>();

List<int> ARG1 = new List<int>();

List<int> GATA3 = new List<int>();

List<int> TGFB = new List<int>();

IFNG.Add(xS[0]);

IFNB.Add(xS[1]);

IL12.Add(xS[2]);

TBET.Add(xS[3]);

INOS.Add(xS[4]);

TNF.Add(xS[5]);

TLR4.Add(xS[6]);

IL1B.Add(xS[7]);

IL6.Add(xS[8]);

IL10.Add(xS[9]);

IL4.Add(xS[10]);

IRF4.Add(xS[11]);

ARG1.Add(xS[12]);

GATA3.Add(xS[13]);

TGFB.Add(xS[14]);

//Logic rule for IFNG

if (IFNGe == 1 &&

TGFB[0] == 0) { IFNG[0] = 1; }

else { IFNG[0] = 0; }

//Logic rule for IFNB

if ((IFNBe == 1 ||

IFNB[0] == 1) && !(

TGFB[0] == 1 ||

IFNG[0] == 1)) { IFNB[0] = 1; }

else { IFNB[0] = 0; }

//Logic rule for IL12

if (TBET[0] == 1 &&

GATA3[0] == 0) { IL12[0] = 1; }

else { IL12[0] = 0; }

//Logic rule for TBET

if (((IL12[0] == 1 ||

IL12e == 1) && (

IFNG[0] == 1 ||

IFNB[0] == 1 ||

IL6[0] == 1)) && !(

GATA3[0] == 1 ||

TGFB[0] == 1)) { TBET[0] = 1; }

else { TBET[0] = 0; }

//Logic rule for INOS

if ((IFNG[0] == 1 ||

IFNB[0] == 1) && (

IL1B[0] == 1 ||

TNF[0] == 1 ||

IL6[0] == 1 ||

TLR4[0] == 1) || GMCSF == 1) { INOS[0] = 1; }

else { INOS[0] = 0; }

//Logic rule for TNF

if ((TNFe == 1 || Insulin == 1 || Ceramide == 1 ||

TNF[0] == 1 ||

TLR4[0] == 1 ||

IL1B[0] == 1 ||

IL6[0] == 1) &&

IL10[0] == 0 &&

IL4[0] == 0) { TNF[0] = 1; }

else { TNF[0] = 0; }

//Logic rule for TLR4

if ((LPS == 1 || Ceramide == 1) &&

IL4[0] == 0) { TLR4[0] = 1; }

else { TLR4[0] = 0; }

//Logic rule for IL1B

if ((IL1Be == 1 ||

IL1B[0] == 1 ||

IL6[0] == 1 ||

TNF[0] == 1 ||

TLR4[0] == 1) &&

IL10[0] == 0 &&

IL4[0] == 0) { IL1B[0] = 1; }

else { IL1B[0] = 0; }

//Logic rule for IL6

if (IL6e == 1 ||

IL6[0] == 1 ||

IL1B[0] == 1 ||

TNF[0] == 1 ||

TLR4[0] == 1 ||

TGFB[0] == 1) { IL6[0] = 1; }

else { IL6[0] = 0; }

//Logic rule for IL10

if ((IL10e == 1 ||

IL10[0] == 1 ||

IL6[0] == 1 ||

TGFB[0] == 1 ||

TLR4[0] == 1) && !(

(IFNG[0] == 1 ||

IFNB[0] == 1))) { IL10[0] = 1; }

else { IL10[0] = 0; }

//Logic rule for IL4

if ((IL4e == 1 ||

GATA3[0] == 1) &&

TBET[0] == 0) { IL4[0] = 1; }

else { IL4[0] = 0; }

//Logic rule for IRF4

if ((IL4[0] == 1 ||

IL6[0] == 1 ||

IRF4[0] == 1 || GMCSF == 1) && !(

TLR4[0] == 1 ||

TNF[0] == 1 ||

IL1B[0] == 1)) { IRF4[0] = 1; }

else { IRF4[0] = 0; }

//Logic rule for ARG1

if (IRF4[0] == 1 &&

IL4[0] == 1) { ARG1[0] = 1; }

else { ARG1[0] = 0; }

//Logic rule for GATA3

if ((GATA3[0] == 1 ||

IL4[0] == 1) && !(

TBET[0] == 1 ||

TGFB[0] == 1)) { GATA3[0] = 1; }

else { GATA3[0] = 0; }

//Logic rule for TGFB

if ((TGFBe == 1 ||

TGFB[0] == 1 ||

IL4[0] == 1 ||

IL6[0] == 1 ||

IL10[0] == 1) && !(

IFNG[0] == 1 ||

IFNB[0] == 1 ||

TLR4[0] == 1 ||

TNF[0] == 1)) { TGFB[0] = 1; }

else { TGFB[0] = 0; }

var node1 = IFNG.Select(x => Convert.ToString(x)).ToList();

var node2 = IFNB.Select(x => Convert.ToString(x)).ToList();

var node3 = IL12.Select(x => Convert.ToString(x)).ToList();

var node4 = TBET.Select(x => Convert.ToString(x)).ToList();

var node5 = INOS.Select(x => Convert.ToString(x)).ToList();

var node6 = TNF.Select(x => Convert.ToString(x)).ToList();

var node7 = TLR4.Select(x => Convert.ToString(x)).ToList();

var node8 = IL1B.Select(x => Convert.ToString(x)).ToList();

var node9 = IL6.Select(x => Convert.ToString(x)).ToList();

var node10 = IL10.Select(x => Convert.ToString(x)).ToList();

var node11 = IL4.Select(x => Convert.ToString(x)).ToList();

var node12 = IRF4.Select(x => Convert.ToString(x)).ToList();

var node13 = ARG1.Select(x => Convert.ToString(x)).ToList();

var node14 = GATA3.Select(x => Convert.ToString(x)).ToList();

var node15 = TGFB.Select(x => Convert.ToString(x)).ToList();

List<string> Nodes = new List<string>();

for (int w = 0; w < 1; w++)

{

Nodes.Add(node1[w] +

node2[w] +

node3[w] +

node4[w] +

node5[w] +

node6[w] +

node7[w] +

node8[w] +

node9[w] +

node10[w] +

node11[w] +

node12[w] +

node13[w] +

node14[w] +

node15[w]);

Output.Add(node1[w] +

node2[w] +

node3[w] +

node4[w] +

node5[w] +

node6[w] +

node7[w] +

node8[w] +

node9[w] +

node10[w] +

node11[w] +

node12[w] +

node13[w] +

node14[w] +

node15[w]);

}

}

//Step 4: Finding all fixed-point attractors

for (int k = 0; k < intialConditions.Count; k++)

{

if (intialConditions[k] == Output[k])

{

FixedPoints.Add(intialConditions[k]);

}

}

Console.WriteLine("Fixed-points in:");

foreach (string s in FixedPoints)

Console.WriteLine(s);

stopWatch.Stop();

// Get the elapsed time as a TimeSpan value.

TimeSpan ts = stopWatch.Elapsed;

// Format and display the TimeSpan value.

string elapsedTime = String.Format("{0:00}:{1:00}:{2:00}.{3:00}",

ts.Hours, ts.Minutes, ts.Seconds,

ts.Milliseconds / 10);

Console.WriteLine("RunTime " + elapsedTime);

## **Implementation of Theorem 4: Epithelial-to-mesenchymal transition in Hepatocytes**

  // See https://aka.ms/new-console-template for more information

using System.Diagnostics;

Stopwatch stopWatch = new Stopwatch();

stopWatch.Start();

List<string> intialConditions = new List<string>();

List<string> InputsZ = new List<string>();

List<string> Output = new List<string>();

List<string> OutputsZ = new List<string>();

List<string> FixedPoints = new List<string>();

//StreamWriter outcomeModel = new StreamWriter(filepath);

int numberNodes = 23;

int maxN = (int)Math.Pow(2, numberNodes);

int n = 0;

//Step 2: Creating all initial states of the network

for (int i = 0; i < maxN; i++)

{

n = i;

string bin = Convert.ToString(n, 2).PadLeft(numberNodes, '0');

//Console.WriteLine(bin);

intialConditions.Add(bin);

string z1 = Convert.ToString(n);

InputsZ.Add(z1);

//Console.WriteLine(z1);

}

Console.WriteLine("Next step:");

//Step 3: Evaluating the network

for (int h = 0; h < maxN; h++)

{

string sentence = intialConditions[h];

char[] charArr = sentence.ToCharArray();

int[] xS = Array.ConvertAll(charArr, c => (int)Char.GetNumericValue(c));

List<int> EZH2 = new List<int>();

List<int> BMI1 = new List<int>();

List<int> E2F = new List<int>();

List<int> CyclinD = new List<int>();

List<int> Bcatenin = new List<int>();

List<int> RB = new List<int>();

List<int> p21 = new List<int>();

List<int> HNF4A = new List<int>();

List<int> HNF6 = new List<int>();

List<int> HNF1A = new List<int>();

List<int> FOXA2 = new List<int>();

List<int> NFkB = new List<int>();

List<int> SNAI1 = new List<int>();

List<int> SNAI2 = new List<int>();

List<int> ZEB1 = new List<int>();

List<int> YAP1 = new List<int>();

List<int> TGFB = new List<int>();

List<int> OCT4 = new List<int>();

List<int> SOX2 = new List<int>();

List<int> NANOG = new List<int>();

List<int> SOX9 = new List<int>();

List<int> p16 = new List<int>();

List<int> p53 = new List<int>();

EZH2.Add(xS[0]);

BMI1.Add(xS[1]);

E2F.Add(xS[2]);

CyclinD.Add(xS[3]);

Bcatenin.Add(xS[4]);

RB.Add(xS[5]);

p21.Add(xS[6]);

HNF4A.Add(xS[7]);

HNF6.Add(xS[8]);

HNF1A.Add(xS[9]);

FOXA2.Add(xS[10]);

NFkB.Add(xS[11]);

SNAI1.Add(xS[12]);

SNAI2.Add(xS[13]);

ZEB1.Add(xS[14]);

YAP1.Add(xS[15]);

TGFB.Add(xS[16]);

OCT4.Add(xS[17]);

SOX2.Add(xS[18]);

NANOG.Add(xS[19]);

SOX9.Add(xS[20]);

p16.Add(xS[21]);

p53.Add(xS[22]);

//Logic rule for EZH2

if (EZH2[0] == 0 && BMI1[0] == 0 && E2F[0] == 0 && Bcatenin[0] == 0 && YAP1[0] == 1 && NFkB[0] == 1 && p53[0] == 0 || EZH2[0] == 0 && BMI1[0] == 0 && E2F[0] == 0 && Bcatenin[0] == 1 && p53[0] == 0 || EZH2[0] == 0 && BMI1[0] == 0 && E2F[0] == 1 && p53[0] == 0 || EZH2[0] == 0 && BMI1[0] == 1 && E2F[0] == 0 && Bcatenin[0] == 0 && YAP1[0] == 0 && NFkB[0] == 0 && TGFB[0] == 1 && p53[0] == 0 && p16[0] == 0 || EZH2[0] == 0 && BMI1[0] == 1 && E2F[0] == 0 && Bcatenin[0] == 0 && YAP1[0] == 0 && NFkB[0] == 1 && p53[0] == 0 && p16[0] == 0 || EZH2[0] == 0 && BMI1[0] == 1 && E2F[0] == 0 && Bcatenin[0] == 0 && YAP1[0] == 1 && NFkB[0] == 0 && TGFB[0] == 1 && p53[0] == 0 && p16[0] == 0 || EZH2[0] == 0 && BMI1[0] == 1 && E2F[0] == 0 && Bcatenin[0] == 0 && YAP1[0] == 1 && NFkB[0] == 1 && p53[0] == 0 || EZH2[0] == 0 && BMI1[0] == 1 && E2F[0] == 0 && Bcatenin[0] == 1 && p53[0] == 0 || EZH2[0] == 0 && BMI1[0] == 1 && E2F[0] == 1 && p53[0] == 0 || EZH2[0] == 1 && E2F[0] == 0 && Bcatenin[0] == 0 && YAP1[0] == 0 && NFkB[0] == 0 && TGFB[0] == 1 && p53[0] == 0 && p16[0] == 0 || EZH2[0] == 1 && E2F[0] == 0 && Bcatenin[0] == 0 && YAP1[0] == 0 && NFkB[0] == 1 && p53[0] == 0 && p16[0] == 0 || EZH2[0] == 1 && E2F[0] == 0 && Bcatenin[0] == 0 && YAP1[0] == 1 && NFkB[0] == 0 && TGFB[0] == 1 && p53[0] == 0 && p16[0] == 0 || EZH2[0] == 1 && E2F[0] == 0 && Bcatenin[0] == 0 && YAP1[0] == 1 && NFkB[0] == 1 && p53[0] == 0 || EZH2[0] == 1 && E2F[0] == 0 && Bcatenin[0] == 1 && p53[0] == 0 || EZH2[0] == 1 && E2F[0] == 1 && p53[0] == 0) { EZH2[0] = 1; }

else { EZH2[0] = 0; }

//Logic rule for BMI1

if (BMI1[0] == 0 && E2F[0] == 0 && Bcatenin[0] == 0 && YAP1[0] == 1 && HNF4A[0] == 0 && NFkB[0] == 1 && SNAI1[0] == 0 && ZEB1[0] == 0 && OCT4[0] == 0 && p53[0] == 0 || BMI1[0] == 0 && E2F[0] == 0 && Bcatenin[0] == 0 && YAP1[0] == 1 && HNF4A[0] == 0 && NFkB[0] == 1 && SNAI1[0] == 0 && ZEB1[0] == 1 && p53[0] == 0 || BMI1[0] == 0 && E2F[0] == 0 && Bcatenin[0] == 0 && YAP1[0] == 1 && HNF4A[0] == 0 && NFkB[0] == 1 && SNAI1[0] == 1 && p53[0] == 0 || BMI1[0] == 0 && E2F[0] == 0 && Bcatenin[0] == 0 && YAP1[0] == 1 && HNF4A[0] == 1 && NFkB[0] == 1 && SNAI1[0] == 0 && ZEB1[0] == 1 && p53[0] == 0 || BMI1[0] == 0 && E2F[0] == 0 && Bcatenin[0] == 0 && YAP1[0] == 1 && HNF4A[0] == 1 && NFkB[0] == 1 && SNAI1[0] == 1 && p53[0] == 0 || BMI1[0] == 0 && E2F[0] == 0 && Bcatenin[0] == 1 && YAP1[0] == 0 && HNF4A[0] == 0 && NFkB[0] == 0 && SNAI1[0] == 0 && ZEB1[0] == 0 && SOX9[0] == 0 && OCT4[0] == 0 && NANOG[0] == 1 && SOX2[0] == 1 && p53[0] == 0 || BMI1[0] == 0 && E2F[0] == 0 && Bcatenin[0] == 1 && YAP1[0] == 0 && HNF4A[0] == 0 && NFkB[0] == 0 && SNAI1[0] == 0 && ZEB1[0] == 0 && SOX9[0] == 1 && OCT4[0] == 0 && SOX2[0] == 1 && p53[0] == 0 || BMI1[0] == 0 && E2F[0] == 0 && Bcatenin[0] == 1 && YAP1[0] == 0 && HNF4A[0] == 0 && NFkB[0] == 0 && SNAI1[0] == 0 && ZEB1[0] == 1 && SOX9[0] == 0 && NANOG[0] == 1 && SOX2[0] == 1 && p53[0] == 0 || BMI1[0] == 0 && E2F[0] == 0 && Bcatenin[0] == 1 && YAP1[0] == 0 && HNF4A[0] == 0 && NFkB[0] == 0 && SNAI1[0] == 0 && ZEB1[0] == 1 && SOX9[0] == 1 && SOX2[0] == 1 && p53[0] == 0 || BMI1[0] == 0 && E2F[0] == 0 && Bcatenin[0] == 1 && YAP1[0] == 0 && HNF4A[0] == 0 && NFkB[0] == 0 && SNAI1[0] == 1 && SOX2[0] == 1 && p53[0] == 0 || BMI1[0] == 0 && E2F[0] == 0 && Bcatenin[0] == 1 && YAP1[0] == 0 && HNF4A[0] == 0 && NFkB[0] == 1 && SNAI1[0] == 0 && ZEB1[0] == 0 && OCT4[0] == 0 && SOX2[0] == 1 && p53[0] == 0 || BMI1[0] == 0 && E2F[0] == 0 && Bcatenin[0] == 1 && YAP1[0] == 0 && HNF4A[0] == 0 && NFkB[0] == 1 && SNAI1[0] == 0 && ZEB1[0] == 1 && SOX2[0] == 1 && p53[0] == 0 || BMI1[0] == 0 && E2F[0] == 0 && Bcatenin[0] == 1 && YAP1[0] == 0 && HNF4A[0] == 0 && NFkB[0] == 1 && SNAI1[0] == 1 && SOX2[0] == 1 && p53[0] == 0 || BMI1[0] == 0 && E2F[0] == 0 && Bcatenin[0] == 1 && YAP1[0] == 0 && HNF4A[0] == 1 && NFkB[0] == 0 && SNAI1[0] == 0 && ZEB1[0] == 1 && SOX9[0] == 0 && NANOG[0] == 1 && SOX2[0] == 1 && p53[0] == 0 || BMI1[0] == 0 && E2F[0] == 0 && Bcatenin[0] == 1 && YAP1[0] == 0 && HNF4A[0] == 1 && NFkB[0] == 0 && SNAI1[0] == 0 && ZEB1[0] == 1 && SOX9[0] == 1 && SOX2[0] == 1 && p53[0] == 0 || BMI1[0] == 0 && E2F[0] == 0 && Bcatenin[0] == 1 && YAP1[0] == 0 && HNF4A[0] == 1 && NFkB[0] == 0 && SNAI1[0] == 1 && SOX2[0] == 1 && p53[0] == 0 || BMI1[0] == 0 && E2F[0] == 0 && Bcatenin[0] == 1 && YAP1[0] == 0 && HNF4A[0] == 1 && NFkB[0] == 1 && SNAI1[0] == 0 && ZEB1[0] == 1 && SOX2[0] == 1 && p53[0] == 0 || BMI1[0] == 0 && E2F[0] == 0 && Bcatenin[0] == 1 && YAP1[0] == 0 && HNF4A[0] == 1 && NFkB[0] == 1 && SNAI1[0] == 1 && SOX2[0] == 1 && p53[0] == 0 || BMI1[0] == 0 && E2F[0] == 0 && Bcatenin[0] == 1 && YAP1[0] == 1 && HNF4A[0] == 0 && NFkB[0] == 0 && SNAI1[0] == 0 && ZEB1[0] == 0 && SOX9[0] == 0 && OCT4[0] == 0 && NANOG[0] == 1 && p53[0] == 0 || BMI1[0] == 0 && E2F[0] == 0 && Bcatenin[0] == 1 && YAP1[0] == 1 && HNF4A[0] == 0 && NFkB[0] == 0 && SNAI1[0] == 0 && ZEB1[0] == 0 && SOX9[0] == 1 && OCT4[0] == 0 && p53[0] == 0 || BMI1[0] == 0 && E2F[0] == 0 && Bcatenin[0] == 1 && YAP1[0] == 1 && HNF4A[0] == 0 && NFkB[0] == 0 && SNAI1[0] == 0 && ZEB1[0] == 1 && SOX9[0] == 0 && NANOG[0] == 1 && p53[0] == 0 || BMI1[0] == 0 && E2F[0] == 0 && Bcatenin[0] == 1 && YAP1[0] == 1 && HNF4A[0] == 0 && NFkB[0] == 0 && SNAI1[0] == 0 && ZEB1[0] == 1 && SOX9[0] == 1 && p53[0] == 0 || BMI1[0] == 0 && E2F[0] == 0 && Bcatenin[0] == 1 && YAP1[0] == 1 && HNF4A[0] == 0 && NFkB[0] == 0 && SNAI1[0] == 1 && p53[0] == 0 || BMI1[0] == 0 && E2F[0] == 0 && Bcatenin[0] == 1 && YAP1[0] == 1 && HNF4A[0] == 0 && NFkB[0] == 1 && SNAI1[0] == 0 && ZEB1[0] == 0 && OCT4[0] == 0 && p53[0] == 0 || BMI1[0] == 0 && E2F[0] == 0 && Bcatenin[0] == 1 && YAP1[0] == 1 && HNF4A[0] == 0 && NFkB[0] == 1 && SNAI1[0] == 0 && ZEB1[0] == 1 && p53[0] == 0 || BMI1[0] == 0 && E2F[0] == 0 && Bcatenin[0] == 1 && YAP1[0] == 1 && HNF4A[0] == 0 && NFkB[0] == 1 && SNAI1[0] == 1 && p53[0] == 0 || BMI1[0] == 0 && E2F[0] == 0 && Bcatenin[0] == 1 && YAP1[0] == 1 && HNF4A[0] == 1 && NFkB[0] == 0 && SNAI1[0] == 0 && ZEB1[0] == 1 && SOX9[0] == 0 && NANOG[0] == 1 && p53[0] == 0 || BMI1[0] == 0 && E2F[0] == 0 && Bcatenin[0] == 1 && YAP1[0] == 1 && HNF4A[0] == 1 && NFkB[0] == 0 && SNAI1[0] == 0 && ZEB1[0] == 1 && SOX9[0] == 1 && p53[0] == 0 || BMI1[0] == 0 && E2F[0] == 0 && Bcatenin[0] == 1 && YAP1[0] == 1 && HNF4A[0] == 1 && NFkB[0] == 0 && SNAI1[0] == 1 && p53[0] == 0 || BMI1[0] == 0 && E2F[0] == 0 && Bcatenin[0] == 1 && YAP1[0] == 1 && HNF4A[0] == 1 && NFkB[0] == 1 && SNAI1[0] == 0 && ZEB1[0] == 1 && p53[0] == 0 || BMI1[0] == 0 && E2F[0] == 0 && Bcatenin[0] == 1 && YAP1[0] == 1 && HNF4A[0] == 1 && NFkB[0] == 1 && SNAI1[0] == 1 && p53[0] == 0 || BMI1[0] == 0 && E2F[0] == 1 && Bcatenin[0] == 0 && YAP1[0] == 0 && HNF4A[0] == 0 && NFkB[0] == 0 && SNAI1[0] == 0 && ZEB1[0] == 0 && OCT4[0] == 0 || BMI1[0] == 0 && E2F[0] == 1 && Bcatenin[0] == 0 && YAP1[0] == 0 && HNF4A[0] == 0 && NFkB[0] == 0 && SNAI1[0] == 0 && ZEB1[0] == 0 && OCT4[0] == 1 && SOX2[0] == 1 && p53[0] == 0 || BMI1[0] == 0 && E2F[0] == 1 && Bcatenin[0] == 0 && YAP1[0] == 0 && HNF4A[0] == 0 && NFkB[0] == 0 && SNAI1[0] == 0 && ZEB1[0] == 1 && OCT4[0] == 0 || BMI1[0] == 0 && E2F[0] == 1 && Bcatenin[0] == 0 && YAP1[0] == 0 && HNF4A[0] == 0 && NFkB[0] == 0 && SNAI1[0] == 0 && ZEB1[0] == 1 && OCT4[0] == 1 && p53[0] == 0 || BMI1[0] == 0 && E2F[0] == 1 && Bcatenin[0] == 0 && YAP1[0] == 0 && HNF4A[0] == 0 && NFkB[0] == 0 && SNAI1[0] == 1 && ZEB1[0] == 0 && OCT4[0] == 0 || BMI1[0] == 0 && E2F[0] == 1 && Bcatenin[0] == 0 && YAP1[0] == 0 && HNF4A[0] == 0 && NFkB[0] == 0 && SNAI1[0] == 1 && ZEB1[0] == 0 && OCT4[0] == 1 && p53[0] == 0 || BMI1[0] == 0 && E2F[0] == 1 && Bcatenin[0] == 0 && YAP1[0] == 0 && HNF4A[0] == 0 && NFkB[0] == 0 && SNAI1[0] == 1 && ZEB1[0] == 1 || BMI1[0] == 0 && E2F[0] == 1 && Bcatenin[0] == 0 && YAP1[0] == 0 && HNF4A[0] == 0 && NFkB[0] == 1 && SNAI1[0] == 0 && OCT4[0] == 0 || BMI1[0] == 0 && E2F[0] == 1 && Bcatenin[0] == 0 && YAP1[0] == 0 && HNF4A[0] == 0 && NFkB[0] == 1 && SNAI1[0] == 0 && OCT4[0] == 1 && p53[0] == 0 || BMI1[0] == 0 && E2F[0] == 1 && Bcatenin[0] == 0 && YAP1[0] == 0 && HNF4A[0] == 0 && NFkB[0] == 1 && SNAI1[0] == 1 && ZEB1[0] == 0 && OCT4[0] == 0 || BMI1[0] == 0 && E2F[0] == 1 && Bcatenin[0] == 0 && YAP1[0] == 0 && HNF4A[0] == 0 && NFkB[0] == 1 && SNAI1[0] == 1 && ZEB1[0] == 0 && OCT4[0] == 1 && p53[0] == 0 || BMI1[0] == 0 && E2F[0] == 1 && Bcatenin[0] == 0 && YAP1[0] == 0 && HNF4A[0] == 0 && NFkB[0] == 1 && SNAI1[0] == 1 && ZEB1[0] == 1 || BMI1[0] == 0 && E2F[0] == 1 && Bcatenin[0] == 0 && YAP1[0] == 0 && HNF4A[0] == 1 && NFkB[0] == 0 && SNAI1[0] == 0 && ZEB1[0] == 0 && TGFB[0] == 0 && SOX2[0] == 1 && p53[0] == 0 || BMI1[0] == 0 && E2F[0] == 1 && Bcatenin[0] == 0 && YAP1[0] == 0 && HNF4A[0] == 1 && NFkB[0] == 0 && SNAI1[0] == 0 && ZEB1[0] == 1 && p53[0] == 0 || BMI1[0] == 0 && E2F[0] == 1 && Bcatenin[0] == 0 && YAP1[0] == 0 && HNF4A[0] == 1 && NFkB[0] == 0 && SNAI1[0] == 1 && ZEB1[0] == 0 && p53[0] == 0 || BMI1[0] == 0 && E2F[0] == 1 && Bcatenin[0] == 0 && YAP1[0] == 0 && HNF4A[0] == 1 && NFkB[0] == 0 && SNAI1[0] == 1 && ZEB1[0] == 1 || BMI1[0] == 0 && E2F[0] == 1 && Bcatenin[0] == 0 && YAP1[0] == 0 && HNF4A[0] == 1 && NFkB[0] == 1 && SNAI1[0] == 0 && p53[0] == 0 || BMI1[0] == 0 && E2F[0] == 1 && Bcatenin[0] == 0 && YAP1[0] == 0 && HNF4A[0] == 1 && NFkB[0] == 1 && SNAI1[0] == 1 && ZEB1[0] == 0 && p53[0] == 0 || BMI1[0] == 0 && E2F[0] == 1 && Bcatenin[0] == 0 && YAP1[0] == 0 && HNF4A[0] == 1 && NFkB[0] == 1 && SNAI1[0] == 1 && ZEB1[0] == 1 || BMI1[0] == 0 && E2F[0] == 1 && Bcatenin[0] == 0 && YAP1[0] == 1 && HNF4A[0] == 0 && SNAI1[0] == 0 && OCT4[0] == 0 || BMI1[0] == 0 && E2F[0] == 1 && Bcatenin[0] == 0 && YAP1[0] == 1 && HNF4A[0] == 0 && SNAI1[0] == 0 && OCT4[0] == 1 && p53[0] == 0 || BMI1[0] == 0 && E2F[0] == 1 && Bcatenin[0] == 0 && YAP1[0] == 1 && HNF4A[0] == 0 && SNAI1[0] == 1 && ZEB1[0] == 0 && OCT4[0] == 0 || BMI1[0] == 0 && E2F[0] == 1 && Bcatenin[0] == 0 && YAP1[0] == 1 && HNF4A[0] == 0 && SNAI1[0] == 1 && ZEB1[0] == 0 && OCT4[0] == 1 && p53[0] == 0 || BMI1[0] == 0 && E2F[0] == 1 && Bcatenin[0] == 0 && YAP1[0] == 1 && HNF4A[0] == 0 && SNAI1[0] == 1 && ZEB1[0] == 1 || BMI1[0] == 0 && E2F[0] == 1 && Bcatenin[0] == 0 && YAP1[0] == 1 && HNF4A[0] == 1 && NFkB[0] == 0 && SNAI1[0] == 0 && ZEB1[0] == 0 && TGFB[0] == 0 && p53[0] == 0 || BMI1[0] == 0 && E2F[0] == 1 && Bcatenin[0] == 0 && YAP1[0] == 1 && HNF4A[0] == 1 && NFkB[0] == 0 && SNAI1[0] == 0 && ZEB1[0] == 1 && p53[0] == 0 || BMI1[0] == 0 && E2F[0] == 1 && Bcatenin[0] == 0 && YAP1[0] == 1 && HNF4A[0] == 1 && NFkB[0] == 0 && SNAI1[0] == 1 && ZEB1[0] == 0 && p53[0] == 0 || BMI1[0] == 0 && E2F[0] == 1 && Bcatenin[0] == 0 && YAP1[0] == 1 && HNF4A[0] == 1 && NFkB[0] == 0 && SNAI1[0] == 1 && ZEB1[0] == 1 || BMI1[0] == 0 && E2F[0] == 1 && Bcatenin[0] == 0 && YAP1[0] == 1 && HNF4A[0] == 1 && NFkB[0] == 1 && SNAI1[0] == 0 && p53[0] == 0 || BMI1[0] == 0 && E2F[0] == 1 && Bcatenin[0] == 0 && YAP1[0] == 1 && HNF4A[0] == 1 && NFkB[0] == 1 && SNAI1[0] == 1 && ZEB1[0] == 0 && p53[0] == 0 || BMI1[0] == 0 && E2F[0] == 1 && Bcatenin[0] == 0 && YAP1[0] == 1 && HNF4A[0] == 1 && NFkB[0] == 1 && SNAI1[0] == 1 && ZEB1[0] == 1 || BMI1[0] == 0 && E2F[0] == 1 && Bcatenin[0] == 1 && HNF4A[0] == 0 && SNAI1[0] == 0 && OCT4[0] == 0 || BMI1[0] == 0 && E2F[0] == 1 && Bcatenin[0] == 1 && HNF4A[0] == 0 && SNAI1[0] == 0 && OCT4[0] == 1 && p53[0] == 0 || BMI1[0] == 0 && E2F[0] == 1 && Bcatenin[0] == 1 && HNF4A[0] == 0 && SNAI1[0] == 1 && ZEB1[0] == 0 && OCT4[0] == 0 || BMI1[0] == 0 && E2F[0] == 1 && Bcatenin[0] == 1 && HNF4A[0] == 0 && SNAI1[0] == 1 && ZEB1[0] == 0 && OCT4[0] == 1 && p53[0] == 0 || BMI1[0] == 0 && E2F[0] == 1 && Bcatenin[0] == 1 && HNF4A[0] == 0 && SNAI1[0] == 1 && ZEB1[0] == 1 || BMI1[0] == 0 && E2F[0] == 1 && Bcatenin[0] == 1 && HNF4A[0] == 1 && NFkB[0] == 0 && SNAI1[0] == 0 && ZEB1[0] == 0 && TGFB[0] == 0 && p53[0] == 0 || BMI1[0] == 0 && E2F[0] == 1 && Bcatenin[0] == 1 && HNF4A[0] == 1 && NFkB[0] == 0 && SNAI1[0] == 0 && ZEB1[0] == 1 && p53[0] == 0 || BMI1[0] == 0 && E2F[0] == 1 && Bcatenin[0] == 1 && HNF4A[0] == 1 && NFkB[0] == 0 && SNAI1[0] == 1 && ZEB1[0] == 0 && p53[0] == 0 || BMI1[0] == 0 && E2F[0] == 1 && Bcatenin[0] == 1 && HNF4A[0] == 1 && NFkB[0] == 0 && SNAI1[0] == 1 && ZEB1[0] == 1 || BMI1[0] == 0 && E2F[0] == 1 && Bcatenin[0] == 1 && HNF4A[0] == 1 && NFkB[0] == 1 && SNAI1[0] == 0 && p53[0] == 0 || BMI1[0] == 0 && E2F[0] == 1 && Bcatenin[0] == 1 && HNF4A[0] == 1 && NFkB[0] == 1 && SNAI1[0] == 1 && ZEB1[0] == 0 && p53[0] == 0 || BMI1[0] == 0 && E2F[0] == 1 && Bcatenin[0] == 1 && HNF4A[0] == 1 && NFkB[0] == 1 && SNAI1[0] == 1 && ZEB1[0] == 1 || BMI1[0] == 1 && E2F[0] == 0 && Bcatenin[0] == 0 && YAP1[0] == 1 && HNF4A[0] == 0 && NFkB[0] == 1 && p53[0] == 0 || BMI1[0] == 1 && E2F[0] == 0 && Bcatenin[0] == 0 && YAP1[0] == 1 && HNF4A[0] == 1 && NFkB[0] == 1 && SNAI1[0] == 0 && ZEB1[0] == 0 && SOX9[0] == 0 && NANOG[0] == 1 && p53[0] == 0 || BMI1[0] == 1 && E2F[0] == 0 && Bcatenin[0] == 0 && YAP1[0] == 1 && HNF4A[0] == 1 && NFkB[0] == 1 && SNAI1[0] == 0 && ZEB1[0] == 0 && SOX9[0] == 1 && p53[0] == 0 || BMI1[0] == 1 && E2F[0] == 0 && Bcatenin[0] == 0 && YAP1[0] == 1 && HNF4A[0] == 1 && NFkB[0] == 1 && SNAI1[0] == 0 && ZEB1[0] == 1 && p53[0] == 0 || BMI1[0] == 1 && E2F[0] == 0 && Bcatenin[0] == 0 && YAP1[0] == 1 && HNF4A[0] == 1 && NFkB[0] == 1 && SNAI1[0] == 1 && p53[0] == 0 || BMI1[0] == 1 && E2F[0] == 0 && Bcatenin[0] == 1 && YAP1[0] == 0 && HNF4A[0] == 0 && NFkB[0] == 0 && SNAI1[0] == 0 && SOX9[0] == 0 && NANOG[0] == 1 && SOX2[0] == 1 && p53[0] == 0 || BMI1[0] == 1 && E2F[0] == 0 && Bcatenin[0] == 1 && YAP1[0] == 0 && HNF4A[0] == 0 && NFkB[0] == 0 && SNAI1[0] == 0 && SOX9[0] == 1 && SOX2[0] == 1 && p53[0] == 0 || BMI1[0] == 1 && E2F[0] == 0 && Bcatenin[0] == 1 && YAP1[0] == 0 && HNF4A[0] == 0 && NFkB[0] == 0 && SNAI1[0] == 1 && SOX2[0] == 1 && p53[0] == 0 || BMI1[0] == 1 && E2F[0] == 0 && Bcatenin[0] == 1 && YAP1[0] == 0 && HNF4A[0] == 0 && NFkB[0] == 1 && SOX2[0] == 1 && p53[0] == 0 || BMI1[0] == 1 && E2F[0] == 0 && Bcatenin[0] == 1 && YAP1[0] == 0 && HNF4A[0] == 1 && NFkB[0] == 0 && SNAI1[0] == 0 && ZEB1[0] == 0 && TGFB[0] == 0 && SOX9[0] == 0 && NANOG[0] == 1 && SOX2[0] == 1 && p53[0] == 0 || BMI1[0] == 1 && E2F[0] == 0 && Bcatenin[0] == 1 && YAP1[0] == 0 && HNF4A[0] == 1 && NFkB[0] == 0 && SNAI1[0] == 0 && ZEB1[0] == 0 && TGFB[0] == 0 && SOX9[0] == 1 && SOX2[0] == 1 && p53[0] == 0 || BMI1[0] == 1 && E2F[0] == 0 && Bcatenin[0] == 1 && YAP1[0] == 0 && HNF4A[0] == 1 && NFkB[0] == 0 && SNAI1[0] == 0 && ZEB1[0] == 1 && SOX9[0] == 0 && NANOG[0] == 1 && SOX2[0] == 1 && p53[0] == 0 || BMI1[0] == 1 && E2F[0] == 0 && Bcatenin[0] == 1 && YAP1[0] == 0 && HNF4A[0] == 1 && NFkB[0] == 0 && SNAI1[0] == 0 && ZEB1[0] == 1 && SOX9[0] == 1 && SOX2[0] == 1 && p53[0] == 0 || BMI1[0] == 1 && E2F[0] == 0 && Bcatenin[0] == 1 && YAP1[0] == 0 && HNF4A[0] == 1 && NFkB[0] == 0 && SNAI1[0] == 1 && SOX2[0] == 1 && p53[0] == 0 || BMI1[0] == 1 && E2F[0] == 0 && Bcatenin[0] == 1 && YAP1[0] == 0 && HNF4A[0] == 1 && NFkB[0] == 1 && SNAI1[0] == 0 && ZEB1[0] == 0 && TGFB[0] == 0 && SOX9[0] == 0 && NANOG[0] == 1 && SOX2[0] == 1 && p53[0] == 0 || BMI1[0] == 1 && E2F[0] == 0 && Bcatenin[0] == 1 && YAP1[0] == 0 && HNF4A[0] == 1 && NFkB[0] == 1 && SNAI1[0] == 0 && ZEB1[0] == 0 && TGFB[0] == 0 && SOX9[0] == 1 && SOX2[0] == 1 && p53[0] == 0 || BMI1[0] == 1 && E2F[0] == 0 && Bcatenin[0] == 1 && YAP1[0] == 0 && HNF4A[0] == 1 && NFkB[0] == 1 && SNAI1[0] == 0 && ZEB1[0] == 1 && SOX2[0] == 1 && p53[0] == 0 || BMI1[0] == 1 && E2F[0] == 0 && Bcatenin[0] == 1 && YAP1[0] == 0 && HNF4A[0] == 1 && NFkB[0] == 1 && SNAI1[0] == 1 && SOX2[0] == 1 && p53[0] == 0 || BMI1[0] == 1 && E2F[0] == 0 && Bcatenin[0] == 1 && YAP1[0] == 1 && HNF4A[0] == 0 && NFkB[0] == 0 && SNAI1[0] == 0 && SOX9[0] == 0 && NANOG[0] == 1 && p53[0] == 0 || BMI1[0] == 1 && E2F[0] == 0 && Bcatenin[0] == 1 && YAP1[0] == 1 && HNF4A[0] == 0 && NFkB[0] == 0 && SNAI1[0] == 0 && SOX9[0] == 1 && p53[0] == 0 || BMI1[0] == 1 && E2F[0] == 0 && Bcatenin[0] == 1 && YAP1[0] == 1 && HNF4A[0] == 0 && NFkB[0] == 0 && SNAI1[0] == 1 && p53[0] == 0 || BMI1[0] == 1 && E2F[0] == 0 && Bcatenin[0] == 1 && YAP1[0] == 1 && HNF4A[0] == 0 && NFkB[0] == 1 && p53[0] == 0 || BMI1[0] == 1 && E2F[0] == 0 && Bcatenin[0] == 1 && YAP1[0] == 1 && HNF4A[0] == 1 && NFkB[0] == 0 && SNAI1[0] == 0 && ZEB1[0] == 0 && TGFB[0] == 0 && SOX9[0] == 0 && NANOG[0] == 1 && p53[0] == 0 || BMI1[0] == 1 && E2F[0] == 0 && Bcatenin[0] == 1 && YAP1[0] == 1 && HNF4A[0] == 1 && NFkB[0] == 0 && SNAI1[0] == 0 && ZEB1[0] == 0 && TGFB[0] == 0 && SOX9[0] == 1 && p53[0] == 0 || BMI1[0] == 1 && E2F[0] == 0 && Bcatenin[0] == 1 && YAP1[0] == 1 && HNF4A[0] == 1 && NFkB[0] == 0 && SNAI1[0] == 0 && ZEB1[0] == 1 && SOX9[0] == 0 && NANOG[0] == 1 && p53[0] == 0 || BMI1[0] == 1 && E2F[0] == 0 && Bcatenin[0] == 1 && YAP1[0] == 1 && HNF4A[0] == 1 && NFkB[0] == 0 && SNAI1[0] == 0 && ZEB1[0] == 1 && SOX9[0] == 1 && p53[0] == 0 || BMI1[0] == 1 && E2F[0] == 0 && Bcatenin[0] == 1 && YAP1[0] == 1 && HNF4A[0] == 1 && NFkB[0] == 0 && SNAI1[0] == 1 && p53[0] == 0 || BMI1[0] == 1 && E2F[0] == 0 && Bcatenin[0] == 1 && YAP1[0] == 1 && HNF4A[0] == 1 && NFkB[0] == 1 && SNAI1[0] == 0 && ZEB1[0] == 0 && SOX9[0] == 0 && NANOG[0] == 1 && p53[0] == 0 || BMI1[0] == 1 && E2F[0] == 0 && Bcatenin[0] == 1 && YAP1[0] == 1 && HNF4A[0] == 1 && NFkB[0] == 1 && SNAI1[0] == 0 && ZEB1[0] == 0 && SOX9[0] == 1 && p53[0] == 0 || BMI1[0] == 1 && E2F[0] == 0 && Bcatenin[0] == 1 && YAP1[0] == 1 && HNF4A[0] == 1 && NFkB[0] == 1 && SNAI1[0] == 0 && ZEB1[0] == 1 && p53[0] == 0 || BMI1[0] == 1 && E2F[0] == 0 && Bcatenin[0] == 1 && YAP1[0] == 1 && HNF4A[0] == 1 && NFkB[0] == 1 && SNAI1[0] == 1 && p53[0] == 0 || BMI1[0] == 1 && E2F[0] == 1) { BMI1[0] = 1; }

else { BMI1[0] = 0; }

//Logic rule for E2F

if (E2F[0] == 0 && Bcatenin[0] == 0 && YAP1[0] == 1 && p21[0] == 0 || E2F[0] == 0 && Bcatenin[0] == 1 && YAP1[0] == 0 && HNF4A[0] == 0 && SOX2[0] == 1 && p53[0] == 0 && p21[0] == 0 && RB[0] == 0 || E2F[0] == 0 && Bcatenin[0] == 1 && YAP1[0] == 0 && HNF4A[0] == 1 && NFkB[0] == 0 && SNAI1[0] == 0 && ZEB1[0] == 0 && TGFB[0] == 0 && SOX2[0] == 1 && p53[0] == 0 && p21[0] == 0 && RB[0] == 0 || E2F[0] == 0 && Bcatenin[0] == 1 && YAP1[0] == 0 && HNF4A[0] == 1 && NFkB[0] == 0 && SNAI1[0] == 0 && ZEB1[0] == 1 && SOX2[0] == 1 && p53[0] == 0 && p21[0] == 0 && RB[0] == 0 || E2F[0] == 0 && Bcatenin[0] == 1 && YAP1[0] == 0 && HNF4A[0] == 1 && NFkB[0] == 1 && SNAI1[0] == 0 && ZEB1[0] == 0 && TGFB[0] == 0 && SOX2[0] == 1 && p53[0] == 0 && p21[0] == 0 && RB[0] == 0 || E2F[0] == 0 && Bcatenin[0] == 1 && YAP1[0] == 1 && p21[0] == 0 || E2F[0] == 1 && Bcatenin[0] == 0 && YAP1[0] == 0 && HNF4A[0] == 0 && NFkB[0] == 0 && SOX2[0] == 0 && p21[0] == 0 && RB[0] == 0 || E2F[0] == 1 && Bcatenin[0] == 0 && YAP1[0] == 0 && HNF4A[0] == 0 && NFkB[0] == 0 && SOX2[0] == 1 && p53[0] == 0 && RB[0] == 0 || E2F[0] == 1 && Bcatenin[0] == 0 && YAP1[0] == 0 && HNF4A[0] == 0 && NFkB[0] == 0 && SOX2[0] == 1 && p53[0] == 1 && p21[0] == 0 && RB[0] == 0 || E2F[0] == 1 && Bcatenin[0] == 0 && YAP1[0] == 0 && HNF4A[0] == 0 && NFkB[0] == 1 && p53[0] == 0 && RB[0] == 0 || E2F[0] == 1 && Bcatenin[0] == 0 && YAP1[0] == 0 && HNF4A[0] == 0 && NFkB[0] == 1 && p53[0] == 1 && p21[0] == 0 && RB[0] == 0 || E2F[0] == 1 && Bcatenin[0] == 0 && YAP1[0] == 0 && HNF4A[0] == 1 && NFkB[0] == 0 && SNAI1[0] == 0 && ZEB1[0] == 0 && TGFB[0] == 0 && SOX2[0] == 0 && p21[0] == 0 && RB[0] == 0 || E2F[0] == 1 && Bcatenin[0] == 0 && YAP1[0] == 0 && HNF4A[0] == 1 && NFkB[0] == 0 && SNAI1[0] == 0 && ZEB1[0] == 0 && TGFB[0] == 0 && SOX2[0] == 1 && p53[0] == 0 && RB[0] == 0 || E2F[0] == 1 && Bcatenin[0] == 0 && YAP1[0] == 0 && HNF4A[0] == 1 && NFkB[0] == 0 && SNAI1[0] == 0 && ZEB1[0] == 0 && TGFB[0] == 0 && SOX2[0] == 1 && p53[0] == 1 && p21[0] == 0 && RB[0] == 0 || E2F[0] == 1 && Bcatenin[0] == 0 && YAP1[0] == 0 && HNF4A[0] == 1 && NFkB[0] == 0 && SNAI1[0] == 0 && ZEB1[0] == 0 && TGFB[0] == 1 && p21[0] == 0 && RB[0] == 0 || E2F[0] == 1 && Bcatenin[0] == 0 && YAP1[0] == 0 && HNF4A[0] == 1 && NFkB[0] == 0 && SNAI1[0] == 0 && ZEB1[0] == 1 && SOX2[0] == 0 && p21[0] == 0 && RB[0] == 0 || E2F[0] == 1 && Bcatenin[0] == 0 && YAP1[0] == 0 && HNF4A[0] == 1 && NFkB[0] == 0 && SNAI1[0] == 0 && ZEB1[0] == 1 && SOX2[0] == 1 && p53[0] == 0 && RB[0] == 0 || E2F[0] == 1 && Bcatenin[0] == 0 && YAP1[0] == 0 && HNF4A[0] == 1 && NFkB[0] == 0 && SNAI1[0] == 0 && ZEB1[0] == 1 && SOX2[0] == 1 && p53[0] == 1 && p21[0] == 0 && RB[0] == 0 || E2F[0] == 1 && Bcatenin[0] == 0 && YAP1[0] == 0 && HNF4A[0] == 1 && NFkB[0] == 0 && SNAI1[0] == 1 && SOX2[0] == 1 && p53[0] == 0 && RB[0] == 0 || E2F[0] == 1 && Bcatenin[0] == 0 && YAP1[0] == 0 && HNF4A[0] == 1 && NFkB[0] == 1 && SNAI1[0] == 0 && ZEB1[0] == 0 && p53[0] == 0 && RB[0] == 0 || E2F[0] == 1 && Bcatenin[0] == 0 && YAP1[0] == 0 && HNF4A[0] == 1 && NFkB[0] == 1 && SNAI1[0] == 0 && ZEB1[0] == 0 && p53[0] == 1 && p21[0] == 0 && RB[0] == 0 || E2F[0] == 1 && Bcatenin[0] == 0 && YAP1[0] == 0 && HNF4A[0] == 1 && NFkB[0] == 1 && SNAI1[0] == 0 && ZEB1[0] == 1 && p53[0] == 0 && RB[0] == 0 || E2F[0] == 1 && Bcatenin[0] == 0 && YAP1[0] == 0 && HNF4A[0] == 1 && NFkB[0] == 1 && SNAI1[0] == 1 && p53[0] == 0 && RB[0] == 0 || E2F[0] == 1 && Bcatenin[0] == 0 && YAP1[0] == 1 && HNF4A[0] == 0 && p53[0] == 0 && p21[0] == 0 || E2F[0] == 1 && Bcatenin[0] == 0 && YAP1[0] == 1 && HNF4A[0] == 0 && p53[0] == 0 && p21[0] == 1 && RB[0] == 0 || E2F[0] == 1 && Bcatenin[0] == 0 && YAP1[0] == 1 && HNF4A[0] == 0 && p53[0] == 1 && p21[0] == 0 || E2F[0] == 1 && Bcatenin[0] == 0 && YAP1[0] == 1 && HNF4A[0] == 1 && NFkB[0] == 0 && SNAI1[0] == 0 && ZEB1[0] == 0 && TGFB[0] == 0 && p53[0] == 0 && p21[0] == 0 || E2F[0] == 1 && Bcatenin[0] == 0 && YAP1[0] == 1 && HNF4A[0] == 1 && NFkB[0] == 0 && SNAI1[0] == 0 && ZEB1[0] == 0 && TGFB[0] == 0 && p53[0] == 0 && p21[0] == 1 && RB[0] == 0 || E2F[0] == 1 && Bcatenin[0] == 0 && YAP1[0] == 1 && HNF4A[0] == 1 && NFkB[0] == 0 && SNAI1[0] == 0 && ZEB1[0] == 0 && TGFB[0] == 0 && p53[0] == 1 && p21[0] == 0 || E2F[0] == 1 && Bcatenin[0] == 0 && YAP1[0] == 1 && HNF4A[0] == 1 && NFkB[0] == 0 && SNAI1[0] == 0 && ZEB1[0] == 0 && TGFB[0] == 1 && p21[0] == 0 || E2F[0] == 1 && Bcatenin[0] == 0 && YAP1[0] == 1 && HNF4A[0] == 1 && NFkB[0] == 0 && SNAI1[0] == 0 && ZEB1[0] == 1 && p53[0] == 0 && p21[0] == 0 || E2F[0] == 1 && Bcatenin[0] == 0 && YAP1[0] == 1 && HNF4A[0] == 1 && NFkB[0] == 0 && SNAI1[0] == 0 && ZEB1[0] == 1 && p53[0] == 0 && p21[0] == 1 && RB[0] == 0 || E2F[0] == 1 && Bcatenin[0] == 0 && YAP1[0] == 1 && HNF4A[0] == 1 && NFkB[0] == 0 && SNAI1[0] == 0 && ZEB1[0] == 1 && p53[0] == 1 && p21[0] == 0 || E2F[0] == 1 && Bcatenin[0] == 0 && YAP1[0] == 1 && HNF4A[0] == 1 && NFkB[0] == 0 && SNAI1[0] == 1 && p53[0] == 0 && p21[0] == 0 || E2F[0] == 1 && Bcatenin[0] == 0 && YAP1[0] == 1 && HNF4A[0] == 1 && NFkB[0] == 0 && SNAI1[0] == 1 && p53[0] == 0 && p21[0] == 1 && RB[0] == 0 || E2F[0] == 1 && Bcatenin[0] == 0 && YAP1[0] == 1 && HNF4A[0] == 1 && NFkB[0] == 0 && SNAI1[0] == 1 && p53[0] == 1 && p21[0] == 0 || E2F[0] == 1 && Bcatenin[0] == 0 && YAP1[0] == 1 && HNF4A[0] == 1 && NFkB[0] == 1 && p53[0] == 0 && p21[0] == 0 || E2F[0] == 1 && Bcatenin[0] == 0 && YAP1[0] == 1 && HNF4A[0] == 1 && NFkB[0] == 1 && p53[0] == 0 && p21[0] == 1 && RB[0] == 0 || E2F[0] == 1 && Bcatenin[0] == 0 && YAP1[0] == 1 && HNF4A[0] == 1 && NFkB[0] == 1 && p53[0] == 1 && p21[0] == 0 || E2F[0] == 1 && Bcatenin[0] == 1 && YAP1[0] == 0 && HNF4A[0] == 0 && p53[0] == 0 && RB[0] == 0 || E2F[0] == 1 && Bcatenin[0] == 1 && YAP1[0] == 0 && HNF4A[0] == 0 && p53[0] == 1 && p21[0] == 0 && RB[0] == 0 || E2F[0] == 1 && Bcatenin[0] == 1 && YAP1[0] == 0 && HNF4A[0] == 1 && NFkB[0] == 0 && SNAI1[0] == 0 && ZEB1[0] == 0 && TGFB[0] == 0 && p53[0] == 0 && RB[0] == 0 || E2F[0] == 1 && Bcatenin[0] == 1 && YAP1[0] == 0 && HNF4A[0] == 1 && NFkB[0] == 0 && SNAI1[0] == 0 && ZEB1[0] == 0 && TGFB[0] == 0 && p53[0] == 1 && p21[0] == 0 && RB[0] == 0 || E2F[0] == 1 && Bcatenin[0] == 1 && YAP1[0] == 0 && HNF4A[0] == 1 && NFkB[0] == 0 && SNAI1[0] == 0 && ZEB1[0] == 0 && TGFB[0] == 1 && p21[0] == 0 && RB[0] == 0 || E2F[0] == 1 && Bcatenin[0] == 1 && YAP1[0] == 0 && HNF4A[0] == 1 && NFkB[0] == 0 && SNAI1[0] == 0 && ZEB1[0] == 1 && p53[0] == 0 && RB[0] == 0 || E2F[0] == 1 && Bcatenin[0] == 1 && YAP1[0] == 0 && HNF4A[0] == 1 && NFkB[0] == 0 && SNAI1[0] == 0 && ZEB1[0] == 1 && p53[0] == 1 && p21[0] == 0 && RB[0] == 0 || E2F[0] == 1 && Bcatenin[0] == 1 && YAP1[0] == 0 && HNF4A[0] == 1 && NFkB[0] == 0 && SNAI1[0] == 1 && p53[0] == 0 && RB[0] == 0 || E2F[0] == 1 && Bcatenin[0] == 1 && YAP1[0] == 0 && HNF4A[0] == 1 && NFkB[0] == 1 && SNAI1[0] == 0 && ZEB1[0] == 0 && p53[0] == 0 && RB[0] == 0 || E2F[0] == 1 && Bcatenin[0] == 1 && YAP1[0] == 0 && HNF4A[0] == 1 && NFkB[0] == 1 && SNAI1[0] == 0 && ZEB1[0] == 0 && p53[0] == 1 && p21[0] == 0 && RB[0] == 0 || E2F[0] == 1 && Bcatenin[0] == 1 && YAP1[0] == 0 && HNF4A[0] == 1 && NFkB[0] == 1 && SNAI1[0] == 0 && ZEB1[0] == 1 && p53[0] == 0 && RB[0] == 0 || E2F[0] == 1 && Bcatenin[0] == 1 && YAP1[0] == 0 && HNF4A[0] == 1 && NFkB[0] == 1 && SNAI1[0] == 1 && p53[0] == 0 && RB[0] == 0 || E2F[0] == 1 && Bcatenin[0] == 1 && YAP1[0] == 1 && HNF4A[0] == 0 && p53[0] == 0 && p21[0] == 0 || E2F[0] == 1 && Bcatenin[0] == 1 && YAP1[0] == 1 && HNF4A[0] == 0 && p53[0] == 0 && p21[0] == 1 && RB[0] == 0 || E2F[0] == 1 && Bcatenin[0] == 1 && YAP1[0] == 1 && HNF4A[0] == 0 && p53[0] == 1 && p21[0] == 0 || E2F[0] == 1 && Bcatenin[0] == 1 && YAP1[0] == 1 && HNF4A[0] == 1 && NFkB[0] == 0 && SNAI1[0] == 0 && ZEB1[0] == 0 && TGFB[0] == 0 && p53[0] == 0 && p21[0] == 0 || E2F[0] == 1 && Bcatenin[0] == 1 && YAP1[0] == 1 && HNF4A[0] == 1 && NFkB[0] == 0 && SNAI1[0] == 0 && ZEB1[0] == 0 && TGFB[0] == 0 && p53[0] == 0 && p21[0] == 1 && RB[0] == 0 || E2F[0] == 1 && Bcatenin[0] == 1 && YAP1[0] == 1 && HNF4A[0] == 1 && NFkB[0] == 0 && SNAI1[0] == 0 && ZEB1[0] == 0 && TGFB[0] == 0 && p53[0] == 1 && p21[0] == 0 || E2F[0] == 1 && Bcatenin[0] == 1 && YAP1[0] == 1 && HNF4A[0] == 1 && NFkB[0] == 0 && SNAI1[0] == 0 && ZEB1[0] == 0 && TGFB[0] == 1 && p21[0] == 0 || E2F[0] == 1 && Bcatenin[0] == 1 && YAP1[0] == 1 && HNF4A[0] == 1 && NFkB[0] == 0 && SNAI1[0] == 0 && ZEB1[0] == 1 && p53[0] == 0 && p21[0] == 0 || E2F[0] == 1 && Bcatenin[0] == 1 && YAP1[0] == 1 && HNF4A[0] == 1 && NFkB[0] == 0 && SNAI1[0] == 0 && ZEB1[0] == 1 && p53[0] == 0 && p21[0] == 1 && RB[0] == 0 || E2F[0] == 1 && Bcatenin[0] == 1 && YAP1[0] == 1 && HNF4A[0] == 1 && NFkB[0] == 0 && SNAI1[0] == 0 && ZEB1[0] == 1 && p53[0] == 1 && p21[0] == 0 || E2F[0] == 1 && Bcatenin[0] == 1 && YAP1[0] == 1 && HNF4A[0] == 1 && NFkB[0] == 0 && SNAI1[0] == 1 && p53[0] == 0 && p21[0] == 0 || E2F[0] == 1 && Bcatenin[0] == 1 && YAP1[0] == 1 && HNF4A[0] == 1 && NFkB[0] == 0 && SNAI1[0] == 1 && p53[0] == 0 && p21[0] == 1 && RB[0] == 0 || E2F[0] == 1 && Bcatenin[0] == 1 && YAP1[0] == 1 && HNF4A[0] == 1 && NFkB[0] == 0 && SNAI1[0] == 1 && p53[0] == 1 && p21[0] == 0 || E2F[0] == 1 && Bcatenin[0] == 1 && YAP1[0] == 1 && HNF4A[0] == 1 && NFkB[0] == 1 && p53[0] == 0 && p21[0] == 0 || E2F[0] == 1 && Bcatenin[0] == 1 && YAP1[0] == 1 && HNF4A[0] == 1 && NFkB[0] == 1 && p53[0] == 0 && p21[0] == 1 && RB[0] == 0 || E2F[0] == 1 && Bcatenin[0] == 1 && YAP1[0] == 1 && HNF4A[0] == 1 && NFkB[0] == 1 && p53[0] == 1 && p21[0] == 0) { E2F[0] = 1; }

else { E2F[0] = 0; }

//Logic rule for CyclinD

if (E2F[0] == 0 && Bcatenin[0] == 0 && YAP1[0] == 1 && NFkB[0] == 1 && p53[0] == 0 && p16[0] == 0 || E2F[0] == 0 && Bcatenin[0] == 1 && YAP1[0] == 0 && HNF4A[0] == 0 && SOX2[0] == 1 && p53[0] == 0 && p16[0] == 0 || E2F[0] == 0 && Bcatenin[0] == 1 && YAP1[0] == 0 && HNF4A[0] == 1 && SNAI1[0] == 0 && ZEB1[0] == 0 && TGFB[0] == 0 && SOX2[0] == 1 && p53[0] == 0 && p16[0] == 0 || E2F[0] == 0 && Bcatenin[0] == 1 && YAP1[0] == 0 && HNF4A[0] == 1 && SNAI1[0] == 0 && ZEB1[0] == 1 && SOX2[0] == 1 && p53[0] == 0 && p16[0] == 0 || E2F[0] == 0 && Bcatenin[0] == 1 && YAP1[0] == 0 && HNF4A[0] == 1 && SNAI1[0] == 1 && SOX2[0] == 1 && p53[0] == 0 && p16[0] == 0 || E2F[0] == 0 && Bcatenin[0] == 1 && YAP1[0] == 1 && p16[0] == 0 || E2F[0] == 1 && Bcatenin[0] == 0 && YAP1[0] == 0 && HNF4A[0] == 0 && HNF6[0] == 0 && FOXA2[0] == 0 && NFkB[0] == 0 && SOX2[0] == 1 && p53[0] == 0 && p16[0] == 0 || E2F[0] == 1 && Bcatenin[0] == 0 && YAP1[0] == 0 && HNF4A[0] == 0 && HNF6[0] == 0 && FOXA2[0] == 0 && NFkB[0] == 1 && p16[0] == 0 || E2F[0] == 1 && Bcatenin[0] == 0 && YAP1[0] == 0 && HNF4A[0] == 0 && HNF6[0] == 0 && FOXA2[0] == 1 && p16[0] == 0 || E2F[0] == 1 && Bcatenin[0] == 0 && YAP1[0] == 0 && HNF4A[0] == 0 && HNF6[0] == 1 && p16[0] == 0 || E2F[0] == 1 && Bcatenin[0] == 0 && YAP1[0] == 0 && HNF4A[0] == 1 && HNF6[0] == 0 && FOXA2[0] == 0 && NFkB[0] == 0 && SNAI1[0] == 0 && ZEB1[0] == 0 && TGFB[0] == 0 && SOX2[0] == 1 && p53[0] == 0 && p16[0] == 0 || E2F[0] == 1 && Bcatenin[0] == 0 && YAP1[0] == 0 && HNF4A[0] == 1 && HNF6[0] == 0 && FOXA2[0] == 0 && NFkB[0] == 0 && SNAI1[0] == 0 && ZEB1[0] == 1 && SOX2[0] == 1 && p53[0] == 0 && p16[0] == 0 || E2F[0] == 1 && Bcatenin[0] == 0 && YAP1[0] == 0 && HNF4A[0] == 1 && HNF6[0] == 0 && FOXA2[0] == 0 && NFkB[0] == 0 && SNAI1[0] == 1 && SOX2[0] == 1 && p53[0] == 0 && p16[0] == 0 || E2F[0] == 1 && Bcatenin[0] == 0 && YAP1[0] == 0 && HNF4A[0] == 1 && HNF6[0] == 0 && FOXA2[0] == 0 && NFkB[0] == 1 && p16[0] == 0 || E2F[0] == 1 && Bcatenin[0] == 0 && YAP1[0] == 0 && HNF4A[0] == 1 && HNF6[0] == 0 && FOXA2[0] == 1 && p16[0] == 0 || E2F[0] == 1 && Bcatenin[0] == 0 && YAP1[0] == 0 && HNF4A[0] == 1 && HNF6[0] == 1 && p16[0] == 0 || E2F[0] == 1 && Bcatenin[0] == 0 && YAP1[0] == 1 && p16[0] == 0 || E2F[0] == 1 && Bcatenin[0] == 1 && p16[0] == 0) { CyclinD[0] = 1; }

else { CyclinD[0] = 0; }

//Logic rule for Bcatenin

if (EZH2[0] == 0 && HNF4A[0] == 0 && TGFB[0] == 1 && SOX2[0] == 0 && p53[0] == 0 || EZH2[0] == 0 && HNF4A[0] == 1 && ZEB1[0] == 0 && SNAI2[0] == 1 && TGFB[0] == 1 && SOX2[0] == 0 && p53[0] == 0 || EZH2[0] == 0 && HNF4A[0] == 1 && ZEB1[0] == 1 && TGFB[0] == 1 && SOX2[0] == 0 && p53[0] == 0 || EZH2[0] == 1 && BMI1[0] == 0 && HNF4A[0] == 0 && TGFB[0] == 1 && SOX2[0] == 0 && p53[0] == 0 || EZH2[0] == 1 && BMI1[0] == 0 && HNF4A[0] == 1 && ZEB1[0] == 0 && SNAI2[0] == 1 && TGFB[0] == 1 && SOX2[0] == 0 && p53[0] == 0 || EZH2[0] == 1 && BMI1[0] == 0 && HNF4A[0] == 1 && ZEB1[0] == 1 && TGFB[0] == 1 && SOX2[0] == 0 && p53[0] == 0 || EZH2[0] == 1 && BMI1[0] == 1 && ZEB1[0] == 0 && SNAI2[0] == 0 && SOX2[0] == 0 && p53[0] == 0 || EZH2[0] == 1 && BMI1[0] == 1 && ZEB1[0] == 0 && SNAI2[0] == 1 && p53[0] == 0 || EZH2[0] == 1 && BMI1[0] == 1 && ZEB1[0] == 1 && p53[0] == 0) { Bcatenin[0] = 1; }

else { Bcatenin[0] = 0; }

//Logic rule for RB

if (CyclinD[0] == 0 || CyclinD[0] == 1 && p16[0] == 0 && p21[0] == 1 || CyclinD[0] == 1 && p16[0] == 1) { RB[0] = 1; }

else { RB[0] = 0; }

//Logic rule for p21

if (E2F[0] == 0 && Bcatenin[0] == 0 && YAP1[0] == 0 && HNF4A[0] == 0 && FOXA2[0] == 0 && NFkB[0] == 0 && SNAI1[0] == 0 && TGFB[0] == 0 && SOX9[0] == 0 && NANOG[0] == 0 && p53[0] == 1 || E2F[0] == 0 && Bcatenin[0] == 0 && YAP1[0] == 0 && HNF4A[0] == 0 && FOXA2[0] == 0 && NFkB[0] == 0 && SNAI1[0] == 0 && TGFB[0] == 0 && SOX9[0] == 0 && NANOG[0] == 1 || E2F[0] == 0 && Bcatenin[0] == 0 && YAP1[0] == 0 && HNF4A[0] == 0 && FOXA2[0] == 0 && NFkB[0] == 0 && SNAI1[0] == 0 && TGFB[0] == 0 && SOX9[0] == 1 || E2F[0] == 0 && Bcatenin[0] == 0 && YAP1[0] == 0 && HNF4A[0] == 0 && FOXA2[0] == 0 && NFkB[0] == 0 && SNAI1[0] == 0 && TGFB[0] == 1 || E2F[0] == 0 && Bcatenin[0] == 0 && YAP1[0] == 0 && HNF4A[0] == 0 && FOXA2[0] == 0 && NFkB[0] == 0 && SNAI1[0] == 1 && TGFB[0] == 0 && SOX9[0] == 0 && p53[0] == 1 || E2F[0] == 0 && Bcatenin[0] == 0 && YAP1[0] == 0 && HNF4A[0] == 0 && FOXA2[0] == 0 && NFkB[0] == 0 && SNAI1[0] == 1 && TGFB[0] == 0 && SOX9[0] == 1 || E2F[0] == 0 && Bcatenin[0] == 0 && YAP1[0] == 0 && HNF4A[0] == 0 && FOXA2[0] == 0 && NFkB[0] == 0 && SNAI1[0] == 1 && TGFB[0] == 1 || E2F[0] == 0 && Bcatenin[0] == 0 && YAP1[0] == 0 && HNF4A[0] == 0 && FOXA2[0] == 0 && NFkB[0] == 1 || E2F[0] == 0 && Bcatenin[0] == 0 && YAP1[0] == 0 && HNF4A[0] == 0 && FOXA2[0] == 1 || E2F[0] == 0 && Bcatenin[0] == 0 && YAP1[0] == 0 && HNF4A[0] == 1 || E2F[0] == 0 && Bcatenin[0] == 0 && YAP1[0] == 1 && HNF4A[0] == 0 && FOXA2[0] == 0 && NFkB[0] == 0 && SNAI1[0] == 0 && TGFB[0] == 0 && SOX9[0] == 0 && NANOG[0] == 0 && p53[0] == 1 || E2F[0] == 0 && Bcatenin[0] == 0 && YAP1[0] == 1 && HNF4A[0] == 0 && FOXA2[0] == 0 && NFkB[0] == 0 && SNAI1[0] == 0 && TGFB[0] == 0 && SOX9[0] == 0 && NANOG[0] == 1 || E2F[0] == 0 && Bcatenin[0] == 0 && YAP1[0] == 1 && HNF4A[0] == 0 && FOXA2[0] == 0 && NFkB[0] == 0 && SNAI1[0] == 0 && TGFB[0] == 0 && SOX9[0] == 1 || E2F[0] == 0 && Bcatenin[0] == 0 && YAP1[0] == 1 && HNF4A[0] == 0 && FOXA2[0] == 0 && NFkB[0] == 0 && SNAI1[0] == 0 && TGFB[0] == 1 || E2F[0] == 0 && Bcatenin[0] == 0 && YAP1[0] == 1 && HNF4A[0] == 0 && FOXA2[0] == 0 && NFkB[0] == 0 && SNAI1[0] == 1 && TGFB[0] == 0 && SOX9[0] == 0 && p53[0] == 1 || E2F[0] == 0 && Bcatenin[0] == 0 && YAP1[0] == 1 && HNF4A[0] == 0 && FOXA2[0] == 0 && NFkB[0] == 0 && SNAI1[0] == 1 && TGFB[0] == 0 && SOX9[0] == 1 || E2F[0] == 0 && Bcatenin[0] == 0 && YAP1[0] == 1 && HNF4A[0] == 0 && FOXA2[0] == 0 && NFkB[0] == 0 && SNAI1[0] == 1 && TGFB[0] == 1 || E2F[0] == 0 && Bcatenin[0] == 0 && YAP1[0] == 1 && HNF4A[0] == 0 && FOXA2[0] == 0 && NFkB[0] == 1 && p53[0] == 1 || E2F[0] == 0 && Bcatenin[0] == 0 && YAP1[0] == 1 && HNF4A[0] == 0 && FOXA2[0] == 1 && NFkB[0] == 0 || E2F[0] == 0 && Bcatenin[0] == 0 && YAP1[0] == 1 && HNF4A[0] == 0 && FOXA2[0] == 1 && NFkB[0] == 1 && p53[0] == 1 || E2F[0] == 0 && Bcatenin[0] == 0 && YAP1[0] == 1 && HNF4A[0] == 1 && NFkB[0] == 0 || E2F[0] == 0 && Bcatenin[0] == 0 && YAP1[0] == 1 && HNF4A[0] == 1 && NFkB[0] == 1 && p53[0] == 1 || E2F[0] == 0 && Bcatenin[0] == 1 && p53[0] == 1 || E2F[0] == 1 && Bcatenin[0] == 0 && YAP1[0] == 0 && HNF4A[0] == 0 && FOXA2[0] == 0 && NFkB[0] == 0 && SNAI1[0] == 0 && TGFB[0] == 0 && SOX9[0] == 0 && NANOG[0] == 0 && p53[0] == 1 || E2F[0] == 1 && Bcatenin[0] == 0 && YAP1[0] == 0 && HNF4A[0] == 0 && FOXA2[0] == 0 && NFkB[0] == 0 && SNAI1[0] == 0 && TGFB[0] == 0 && SOX9[0] == 0 && NANOG[0] == 1 && SOX2[0] == 0 || E2F[0] == 1 && Bcatenin[0] == 0 && YAP1[0] == 0 && HNF4A[0] == 0 && FOXA2[0] == 0 && NFkB[0] == 0 && SNAI1[0] == 0 && TGFB[0] == 0 && SOX9[0] == 0 && NANOG[0] == 1 && SOX2[0] == 1 && p53[0] == 1 || E2F[0] == 1 && Bcatenin[0] == 0 && YAP1[0] == 0 && HNF4A[0] == 0 && FOXA2[0] == 0 && NFkB[0] == 0 && SNAI1[0] == 0 && TGFB[0] == 0 && SOX9[0] == 1 && SOX2[0] == 0 || E2F[0] == 1 && Bcatenin[0] == 0 && YAP1[0] == 0 && HNF4A[0] == 0 && FOXA2[0] == 0 && NFkB[0] == 0 && SNAI1[0] == 0 && TGFB[0] == 0 && SOX9[0] == 1 && SOX2[0] == 1 && p53[0] == 1 || E2F[0] == 1 && Bcatenin[0] == 0 && YAP1[0] == 0 && HNF4A[0] == 0 && FOXA2[0] == 0 && NFkB[0] == 0 && SNAI1[0] == 0 && TGFB[0] == 1 && SOX2[0] == 0 || E2F[0] == 1 && Bcatenin[0] == 0 && YAP1[0] == 0 && HNF4A[0] == 0 && FOXA2[0] == 0 && NFkB[0] == 0 && SNAI1[0] == 0 && TGFB[0] == 1 && SOX2[0] == 1 && p53[0] == 1 || E2F[0] == 1 && Bcatenin[0] == 0 && YAP1[0] == 0 && HNF4A[0] == 0 && FOXA2[0] == 0 && NFkB[0] == 0 && SNAI1[0] == 1 && TGFB[0] == 0 && SOX9[0] == 0 && p53[0] == 1 || E2F[0] == 1 && Bcatenin[0] == 0 && YAP1[0] == 0 && HNF4A[0] == 0 && FOXA2[0] == 0 && NFkB[0] == 0 && SNAI1[0] == 1 && TGFB[0] == 0 && SOX9[0] == 1 && SOX2[0] == 0 || E2F[0] == 1 && Bcatenin[0] == 0 && YAP1[0] == 0 && HNF4A[0] == 0 && FOXA2[0] == 0 && NFkB[0] == 0 && SNAI1[0] == 1 && TGFB[0] == 0 && SOX9[0] == 1 && SOX2[0] == 1 && p53[0] == 1 || E2F[0] == 1 && Bcatenin[0] == 0 && YAP1[0] == 0 && HNF4A[0] == 0 && FOXA2[0] == 0 && NFkB[0] == 0 && SNAI1[0] == 1 && TGFB[0] == 1 && SOX2[0] == 0 || E2F[0] == 1 && Bcatenin[0] == 0 && YAP1[0] == 0 && HNF4A[0] == 0 && FOXA2[0] == 0 && NFkB[0] == 0 && SNAI1[0] == 1 && TGFB[0] == 1 && SOX2[0] == 1 && p53[0] == 1 || E2F[0] == 1 && Bcatenin[0] == 0 && YAP1[0] == 0 && HNF4A[0] == 0 && FOXA2[0] == 0 && NFkB[0] == 1 && p53[0] == 1 || E2F[0] == 1 && Bcatenin[0] == 0 && YAP1[0] == 0 && HNF4A[0] == 0 && FOXA2[0] == 1 && NFkB[0] == 0 && SOX2[0] == 0 || E2F[0] == 1 && Bcatenin[0] == 0 && YAP1[0] == 0 && HNF4A[0] == 0 && FOXA2[0] == 1 && NFkB[0] == 0 && SOX2[0] == 1 && p53[0] == 1 || E2F[0] == 1 && Bcatenin[0] == 0 && YAP1[0] == 0 && HNF4A[0] == 0 && FOXA2[0] == 1 && NFkB[0] == 1 && p53[0] == 1 || E2F[0] == 1 && Bcatenin[0] == 0 && YAP1[0] == 0 && HNF4A[0] == 1 && NFkB[0] == 0 && SNAI1[0] == 0 && ZEB1[0] == 0 && TGFB[0] == 0 && SOX2[0] == 0 || E2F[0] == 1 && Bcatenin[0] == 0 && YAP1[0] == 0 && HNF4A[0] == 1 && NFkB[0] == 0 && SNAI1[0] == 0 && ZEB1[0] == 0 && TGFB[0] == 0 && SOX2[0] == 1 && p53[0] == 1 || E2F[0] == 1 && Bcatenin[0] == 0 && YAP1[0] == 0 && HNF4A[0] == 1 && NFkB[0] == 0 && SNAI1[0] == 0 && ZEB1[0] == 0 && TGFB[0] == 1 || E2F[0] == 1 && Bcatenin[0] == 0 && YAP1[0] == 0 && HNF4A[0] == 1 && NFkB[0] == 0 && SNAI1[0] == 0 && ZEB1[0] == 1 && SOX2[0] == 0 || E2F[0] == 1 && Bcatenin[0] == 0 && YAP1[0] == 0 && HNF4A[0] == 1 && NFkB[0] == 0 && SNAI1[0] == 0 && ZEB1[0] == 1 && SOX2[0] == 1 && p53[0] == 1 || E2F[0] == 1 && Bcatenin[0] == 0 && YAP1[0] == 0 && HNF4A[0] == 1 && NFkB[0] == 0 && SNAI1[0] == 1 && SOX2[0] == 0 || E2F[0] == 1 && Bcatenin[0] == 0 && YAP1[0] == 0 && HNF4A[0] == 1 && NFkB[0] == 0 && SNAI1[0] == 1 && SOX2[0] == 1 && p53[0] == 1 || E2F[0] == 1 && Bcatenin[0] == 0 && YAP1[0] == 0 && HNF4A[0] == 1 && NFkB[0] == 1 && p53[0] == 1 || E2F[0] == 1 && Bcatenin[0] == 0 && YAP1[0] == 1 && HNF4A[0] == 0 && p53[0] == 1 || E2F[0] == 1 && Bcatenin[0] == 0 && YAP1[0] == 1 && HNF4A[0] == 1 && NFkB[0] == 0 && SNAI1[0] == 0 && ZEB1[0] == 0 && TGFB[0] == 0 && p53[0] == 1 || E2F[0] == 1 && Bcatenin[0] == 0 && YAP1[0] == 1 && HNF4A[0] == 1 && NFkB[0] == 0 && SNAI1[0] == 0 && ZEB1[0] == 0 && TGFB[0] == 1 || E2F[0] == 1 && Bcatenin[0] == 0 && YAP1[0] == 1 && HNF4A[0] == 1 && NFkB[0] == 0 && SNAI1[0] == 0 && ZEB1[0] == 1 && p53[0] == 1 || E2F[0] == 1 && Bcatenin[0] == 0 && YAP1[0] == 1 && HNF4A[0] == 1 && NFkB[0] == 0 && SNAI1[0] == 1 && p53[0] == 1 || E2F[0] == 1 && Bcatenin[0] == 0 && YAP1[0] == 1 && HNF4A[0] == 1 && NFkB[0] == 1 && p53[0] == 1 || E2F[0] == 1 && Bcatenin[0] == 1 && p53[0] == 1) { p21[0] = 1; }

else { p21[0] = 0; }

//Logic rule for HNF4A

if (E2F[0] == 0 && Bcatenin[0] == 0 && YAP1[0] == 0 && HNF1A[0] == 0 && HNF4A[0] == 0 && HNF6[0] == 0 && FOXA2[0] == 0 && SNAI1[0] == 0 && ZEB1[0] == 0 && SNAI2[0] == 0 && SOX9[0] == 0 && NANOG[0] == 0 && SOX2[0] == 1 && p53[0] == 0 || E2F[0] == 0 && Bcatenin[0] == 0 && YAP1[0] == 0 && HNF1A[0] == 0 && HNF4A[0] == 0 && HNF6[0] == 0 && FOXA2[0] == 0 && SNAI1[0] == 0 && ZEB1[0] == 0 && SNAI2[0] == 0 && SOX9[0] == 1 && NANOG[0] == 0 && SOX2[0] == 1 || E2F[0] == 0 && Bcatenin[0] == 0 && YAP1[0] == 0 && HNF1A[0] == 0 && HNF4A[0] == 0 && HNF6[0] == 0 && FOXA2[0] == 0 && SNAI1[0] == 0 && ZEB1[0] == 1 && SNAI2[0] == 0 && NANOG[0] == 0 && SOX2[0] == 1 && p53[0] == 0 || E2F[0] == 0 && Bcatenin[0] == 0 && YAP1[0] == 0 && HNF1A[0] == 0 && HNF4A[0] == 0 && HNF6[0] == 0 && FOXA2[0] == 1 && SNAI1[0] == 0 && SNAI2[0] == 0 || E2F[0] == 0 && Bcatenin[0] == 0 && YAP1[0] == 0 && HNF1A[0] == 0 && HNF4A[0] == 0 && HNF6[0] == 1 && SNAI1[0] == 0 && SNAI2[0] == 0 || E2F[0] == 0 && Bcatenin[0] == 0 && YAP1[0] == 0 && HNF1A[0] == 0 && HNF4A[0] == 1 && HNF6[0] == 0 && FOXA2[0] == 0 && SNAI1[0] == 0 && SNAI2[0] == 0 || E2F[0] == 0 && Bcatenin[0] == 0 && YAP1[0] == 0 && HNF1A[0] == 0 && HNF4A[0] == 1 && HNF6[0] == 0 && FOXA2[0] == 1 && SNAI1[0] == 0 || E2F[0] == 0 && Bcatenin[0] == 0 && YAP1[0] == 0 && HNF1A[0] == 0 && HNF4A[0] == 1 && HNF6[0] == 0 && FOXA2[0] == 1 && SNAI1[0] == 1 && SNAI2[0] == 0 || E2F[0] == 0 && Bcatenin[0] == 0 && YAP1[0] == 0 && HNF1A[0] == 0 && HNF4A[0] == 1 && HNF6[0] == 1 && SNAI1[0] == 0 || E2F[0] == 0 && Bcatenin[0] == 0 && YAP1[0] == 0 && HNF1A[0] == 0 && HNF4A[0] == 1 && HNF6[0] == 1 && SNAI1[0] == 1 && SNAI2[0] == 0 || E2F[0] == 0 && Bcatenin[0] == 0 && YAP1[0] == 0 && HNF1A[0] == 1 && HNF4A[0] == 0 && HNF6[0] == 0 && FOXA2[0] == 0 && SNAI1[0] == 0 && SNAI2[0] == 0 || E2F[0] == 0 && Bcatenin[0] == 0 && YAP1[0] == 0 && HNF1A[0] == 1 && HNF4A[0] == 0 && HNF6[0] == 0 && FOXA2[0] == 1 && NFkB[0] == 0 && SNAI1[0] == 0 || E2F[0] == 0 && Bcatenin[0] == 0 && YAP1[0] == 0 && HNF1A[0] == 1 && HNF4A[0] == 0 && HNF6[0] == 0 && FOXA2[0] == 1 && NFkB[0] == 0 && SNAI1[0] == 1 && SNAI2[0] == 0 || E2F[0] == 0 && Bcatenin[0] == 0 && YAP1[0] == 0 && HNF1A[0] == 1 && HNF4A[0] == 0 && HNF6[0] == 0 && FOXA2[0] == 1 && NFkB[0] == 1 && SNAI1[0] == 0 && SNAI2[0] == 0 || E2F[0] == 0 && Bcatenin[0] == 0 && YAP1[0] == 0 && HNF1A[0] == 1 && HNF4A[0] == 0 && HNF6[0] == 0 && FOXA2[0] == 1 && NFkB[0] == 1 && SNAI1[0] == 0 && SNAI2[0] == 1 && p53[0] == 0 || E2F[0] == 0 && Bcatenin[0] == 0 && YAP1[0] == 0 && HNF1A[0] == 1 && HNF4A[0] == 0 && HNF6[0] == 0 && FOXA2[0] == 1 && NFkB[0] == 1 && SNAI1[0] == 1 && SNAI2[0] == 0 && p53[0] == 0 || E2F[0] == 0 && Bcatenin[0] == 0 && YAP1[0] == 0 && HNF1A[0] == 1 && HNF4A[0] == 0 && HNF6[0] == 1 && NFkB[0] == 0 && SNAI1[0] == 0 || E2F[0] == 0 && Bcatenin[0] == 0 && YAP1[0] == 0 && HNF1A[0] == 1 && HNF4A[0] == 0 && HNF6[0] == 1 && NFkB[0] == 0 && SNAI1[0] == 1 && SNAI2[0] == 0 || E2F[0] == 0 && Bcatenin[0] == 0 && YAP1[0] == 0 && HNF1A[0] == 1 && HNF4A[0] == 0 && HNF6[0] == 1 && NFkB[0] == 1 && SNAI1[0] == 0 && SNAI2[0] == 0 || E2F[0] == 0 && Bcatenin[0] == 0 && YAP1[0] == 0 && HNF1A[0] == 1 && HNF4A[0] == 0 && HNF6[0] == 1 && NFkB[0] == 1 && SNAI1[0] == 0 && SNAI2[0] == 1 && p53[0] == 0 || E2F[0] == 0 && Bcatenin[0] == 0 && YAP1[0] == 0 && HNF1A[0] == 1 && HNF4A[0] == 0 && HNF6[0] == 1 && NFkB[0] == 1 && SNAI1[0] == 1 && SNAI2[0] == 0 && p53[0] == 0 || E2F[0] == 0 && Bcatenin[0] == 0 && YAP1[0] == 0 && HNF1A[0] == 1 && HNF4A[0] == 1 && SNAI1[0] == 0 || E2F[0] == 0 && Bcatenin[0] == 0 && YAP1[0] == 0 && HNF1A[0] == 1 && HNF4A[0] == 1 && SNAI1[0] == 1 && SNAI2[0] == 0 || E2F[0] == 0 && Bcatenin[0] == 0 && YAP1[0] == 1 && HNF1A[0] == 0 && HNF4A[0] == 0 && HNF6[0] == 0 && FOXA2[0] == 0 && NFkB[0] == 0 && SNAI1[0] == 0 && ZEB1[0] == 0 && SNAI2[0] == 0 && SOX9[0] == 0 && NANOG[0] == 0 && SOX2[0] == 1 && p53[0] == 0 || E2F[0] == 0 && Bcatenin[0] == 0 && YAP1[0] == 1 && HNF1A[0] == 0 && HNF4A[0] == 0 && HNF6[0] == 0 && FOXA2[0] == 0 && NFkB[0] == 0 && SNAI1[0] == 0 && ZEB1[0] == 0 && SNAI2[0] == 0 && SOX9[0] == 1 && NANOG[0] == 0 && SOX2[0] == 1 || E2F[0] == 0 && Bcatenin[0] == 0 && YAP1[0] == 1 && HNF1A[0] == 0 && HNF4A[0] == 0 && HNF6[0] == 0 && FOXA2[0] == 0 && NFkB[0] == 0 && SNAI1[0] == 0 && ZEB1[0] == 1 && SNAI2[0] == 0 && NANOG[0] == 0 && SOX2[0] == 1 && p53[0] == 0 || E2F[0] == 0 && Bcatenin[0] == 0 && YAP1[0] == 1 && HNF1A[0] == 0 && HNF4A[0] == 0 && HNF6[0] == 0 && FOXA2[0] == 1 && NFkB[0] == 0 && SNAI1[0] == 0 && SNAI2[0] == 0 || E2F[0] == 0 && Bcatenin[0] == 0 && YAP1[0] == 1 && HNF1A[0] == 0 && HNF4A[0] == 0 && HNF6[0] == 0 && FOXA2[0] == 1 && NFkB[0] == 1 && SNAI1[0] == 0 && SNAI2[0] == 0 && p53[0] == 0 || E2F[0] == 0 && Bcatenin[0] == 0 && YAP1[0] == 1 && HNF1A[0] == 0 && HNF4A[0] == 0 && HNF6[0] == 1 && NFkB[0] == 0 && SNAI1[0] == 0 && SNAI2[0] == 0 || E2F[0] == 0 && Bcatenin[0] == 0 && YAP1[0] == 1 && HNF1A[0] == 0 && HNF4A[0] == 0 && HNF6[0] == 1 && NFkB[0] == 1 && SNAI1[0] == 0 && SNAI2[0] == 0 && p53[0] == 0 || E2F[0] == 0 && Bcatenin[0] == 0 && YAP1[0] == 1 && HNF1A[0] == 0 && HNF4A[0] == 1 && HNF6[0] == 0 && FOXA2[0] == 0 && NFkB[0] == 0 && SNAI1[0] == 0 && SNAI2[0] == 0 || E2F[0] == 0 && Bcatenin[0] == 0 && YAP1[0] == 1 && HNF1A[0] == 0 && HNF4A[0] == 1 && HNF6[0] == 0 && FOXA2[0] == 0 && NFkB[0] == 1 && SNAI1[0] == 0 && SNAI2[0] == 0 && p53[0] == 0 || E2F[0] == 0 && Bcatenin[0] == 0 && YAP1[0] == 1 && HNF1A[0] == 0 && HNF4A[0] == 1 && HNF6[0] == 0 && FOXA2[0] == 1 && SNAI1[0] == 0 || E2F[0] == 0 && Bcatenin[0] == 0 && YAP1[0] == 1 && HNF1A[0] == 0 && HNF4A[0] == 1 && HNF6[0] == 0 && FOXA2[0] == 1 && SNAI1[0] == 1 && SNAI2[0] == 0 || E2F[0] == 0 && Bcatenin[0] == 0 && YAP1[0] == 1 && HNF1A[0] == 0 && HNF4A[0] == 1 && HNF6[0] == 1 && SNAI1[0] == 0 || E2F[0] == 0 && Bcatenin[0] == 0 && YAP1[0] == 1 && HNF1A[0] == 0 && HNF4A[0] == 1 && HNF6[0] == 1 && SNAI1[0] == 1 && SNAI2[0] == 0 || E2F[0] == 0 && Bcatenin[0] == 0 && YAP1[0] == 1 && HNF1A[0] == 1 && HNF4A[0] == 0 && NFkB[0] == 0 && SNAI1[0] == 0 && SNAI2[0] == 0 || E2F[0] == 0 && Bcatenin[0] == 0 && YAP1[0] == 1 && HNF1A[0] == 1 && HNF4A[0] == 0 && NFkB[0] == 1 && SNAI1[0] == 0 && SNAI2[0] == 0 && p53[0] == 0 || E2F[0] == 0 && Bcatenin[0] == 0 && YAP1[0] == 1 && HNF1A[0] == 1 && HNF4A[0] == 1 && SNAI1[0] == 0 || E2F[0] == 0 && Bcatenin[0] == 0 && YAP1[0] == 1 && HNF1A[0] == 1 && HNF4A[0] == 1 && SNAI1[0] == 1 && SNAI2[0] == 0 || E2F[0] == 0 && Bcatenin[0] == 1 && YAP1[0] == 0 && HNF1A[0] == 0 && HNF4A[0] == 0 && HNF6[0] == 0 && FOXA2[0] == 0 && SNAI1[0] == 0 && ZEB1[0] == 0 && SNAI2[0] == 0 && SOX9[0] == 1 && NANOG[0] == 0 && SOX2[0] == 1 && p53[0] == 1 || E2F[0] == 0 && Bcatenin[0] == 1 && YAP1[0] == 0 && HNF1A[0] == 0 && HNF4A[0] == 0 && HNF6[0] == 0 && FOXA2[0] == 1 && SNAI1[0] == 0 && SNAI2[0] == 0 || E2F[0] == 0 && Bcatenin[0] == 1 && YAP1[0] == 0 && HNF1A[0] == 0 && HNF4A[0] == 0 && HNF6[0] == 1 && SNAI1[0] == 0 && SNAI2[0] == 0 || E2F[0] == 0 && Bcatenin[0] == 1 && YAP1[0] == 0 && HNF1A[0] == 0 && HNF4A[0] == 1 && HNF6[0] == 0 && FOXA2[0] == 0 && SNAI1[0] == 0 && SNAI2[0] == 0 || E2F[0] == 0 && Bcatenin[0] == 1 && YAP1[0] == 0 && HNF1A[0] == 0 && HNF4A[0] == 1 && HNF6[0] == 0 && FOXA2[0] == 1 && SNAI1[0] == 0 || E2F[0] == 0 && Bcatenin[0] == 1 && YAP1[0] == 0 && HNF1A[0] == 0 && HNF4A[0] == 1 && HNF6[0] == 0 && FOXA2[0] == 1 && SNAI1[0] == 1 && SNAI2[0] == 0 || E2F[0] == 0 && Bcatenin[0] == 1 && YAP1[0] == 0 && HNF1A[0] == 0 && HNF4A[0] == 1 && HNF6[0] == 1 && SNAI1[0] == 0 || E2F[0] == 0 && Bcatenin[0] == 1 && YAP1[0] == 0 && HNF1A[0] == 0 && HNF4A[0] == 1 && HNF6[0] == 1 && SNAI1[0] == 1 && SNAI2[0] == 0 || E2F[0] == 0 && Bcatenin[0] == 1 && YAP1[0] == 0 && HNF1A[0] == 1 && HNF4A[0] == 0 && HNF6[0] == 0 && FOXA2[0] == 0 && SNAI1[0] == 0 && SNAI2[0] == 0 || E2F[0] == 0 && Bcatenin[0] == 1 && YAP1[0] == 0 && HNF1A[0] == 1 && HNF4A[0] == 0 && HNF6[0] == 0 && FOXA2[0] == 1 && NFkB[0] == 0 && SNAI1[0] == 0 || E2F[0] == 0 && Bcatenin[0] == 1 && YAP1[0] == 0 && HNF1A[0] == 1 && HNF4A[0] == 0 && HNF6[0] == 0 && FOXA2[0] == 1 && NFkB[0] == 0 && SNAI1[0] == 1 && SNAI2[0] == 0 || E2F[0] == 0 && Bcatenin[0] == 1 && YAP1[0] == 0 && HNF1A[0] == 1 && HNF4A[0] == 0 && HNF6[0] == 0 && FOXA2[0] == 1 && NFkB[0] == 1 && SNAI1[0] == 0 && SNAI2[0] == 0 || E2F[0] == 0 && Bcatenin[0] == 1 && YAP1[0] == 0 && HNF1A[0] == 1 && HNF4A[0] == 0 && HNF6[0] == 0 && FOXA2[0] == 1 && NFkB[0] == 1 && SNAI1[0] == 0 && SNAI2[0] == 1 && p53[0] == 0 || E2F[0] == 0 && Bcatenin[0] == 1 && YAP1[0] == 0 && HNF1A[0] == 1 && HNF4A[0] == 0 && HNF6[0] == 0 && FOXA2[0] == 1 && NFkB[0] == 1 && SNAI1[0] == 1 && SNAI2[0] == 0 && p53[0] == 0 || E2F[0] == 0 && Bcatenin[0] == 1 && YAP1[0] == 0 && HNF1A[0] == 1 && HNF4A[0] == 0 && HNF6[0] == 1 && NFkB[0] == 0 && SNAI1[0] == 0 || E2F[0] == 0 && Bcatenin[0] == 1 && YAP1[0] == 0 && HNF1A[0] == 1 && HNF4A[0] == 0 && HNF6[0] == 1 && NFkB[0] == 0 && SNAI1[0] == 1 && SNAI2[0] == 0 || E2F[0] == 0 && Bcatenin[0] == 1 && YAP1[0] == 0 && HNF1A[0] == 1 && HNF4A[0] == 0 && HNF6[0] == 1 && NFkB[0] == 1 && SNAI1[0] == 0 && SNAI2[0] == 0 || E2F[0] == 0 && Bcatenin[0] == 1 && YAP1[0] == 0 && HNF1A[0] == 1 && HNF4A[0] == 0 && HNF6[0] == 1 && NFkB[0] == 1 && SNAI1[0] == 0 && SNAI2[0] == 1 && p53[0] == 0 || E2F[0] == 0 && Bcatenin[0] == 1 && YAP1[0] == 0 && HNF1A[0] == 1 && HNF4A[0] == 0 && HNF6[0] == 1 && NFkB[0] == 1 && SNAI1[0] == 1 && SNAI2[0] == 0 && p53[0] == 0 || E2F[0] == 0 && Bcatenin[0] == 1 && YAP1[0] == 0 && HNF1A[0] == 1 && HNF4A[0] == 1 && SNAI1[0] == 0 || E2F[0] == 0 && Bcatenin[0] == 1 && YAP1[0] == 0 && HNF1A[0] == 1 && HNF4A[0] == 1 && SNAI1[0] == 1 && SNAI2[0] == 0 || E2F[0] == 0 && Bcatenin[0] == 1 && YAP1[0] == 1 && HNF1A[0] == 0 && HNF4A[0] == 0 && HNF6[0] == 0 && FOXA2[0] == 0 && NFkB[0] == 0 && SNAI1[0] == 0 && ZEB1[0] == 0 && SNAI2[0] == 0 && SOX9[0] == 1 && NANOG[0] == 0 && SOX2[0] == 1 && p53[0] == 1 || E2F[0] == 0 && Bcatenin[0] == 1 && YAP1[0] == 1 && HNF1A[0] == 0 && HNF4A[0] == 0 && HNF6[0] == 0 && FOXA2[0] == 1 && NFkB[0] == 0 && SNAI1[0] == 0 && SNAI2[0] == 0 || E2F[0] == 0 && Bcatenin[0] == 1 && YAP1[0] == 1 && HNF1A[0] == 0 && HNF4A[0] == 0 && HNF6[0] == 0 && FOXA2[0] == 1 && NFkB[0] == 1 && SNAI1[0] == 0 && SNAI2[0] == 0 && p53[0] == 0 || E2F[0] == 0 && Bcatenin[0] == 1 && YAP1[0] == 1 && HNF1A[0] == 0 && HNF4A[0] == 0 && HNF6[0] == 1 && NFkB[0] == 0 && SNAI1[0] == 0 && SNAI2[0] == 0 || E2F[0] == 0 && Bcatenin[0] == 1 && YAP1[0] == 1 && HNF1A[0] == 0 && HNF4A[0] == 0 && HNF6[0] == 1 && NFkB[0] == 1 && SNAI1[0] == 0 && SNAI2[0] == 0 && p53[0] == 0 || E2F[0] == 0 && Bcatenin[0] == 1 && YAP1[0] == 1 && HNF1A[0] == 0 && HNF4A[0] == 1 && HNF6[0] == 0 && FOXA2[0] == 0 && NFkB[0] == 0 && SNAI1[0] == 0 && SNAI2[0] == 0 || E2F[0] == 0 && Bcatenin[0] == 1 && YAP1[0] == 1 && HNF1A[0] == 0 && HNF4A[0] == 1 && HNF6[0] == 0 && FOXA2[0] == 0 && NFkB[0] == 1 && SNAI1[0] == 0 && SNAI2[0] == 0 && p53[0] == 0 || E2F[0] == 0 && Bcatenin[0] == 1 && YAP1[0] == 1 && HNF1A[0] == 0 && HNF4A[0] == 1 && HNF6[0] == 0 && FOXA2[0] == 1 && SNAI1[0] == 0 || E2F[0] == 0 && Bcatenin[0] == 1 && YAP1[0] == 1 && HNF1A[0] == 0 && HNF4A[0] == 1 && HNF6[0] == 0 && FOXA2[0] == 1 && SNAI1[0] == 1 && SNAI2[0] == 0 || E2F[0] == 0 && Bcatenin[0] == 1 && YAP1[0] == 1 && HNF1A[0] == 0 && HNF4A[0] == 1 && HNF6[0] == 1 && SNAI1[0] == 0 || E2F[0] == 0 && Bcatenin[0] == 1 && YAP1[0] == 1 && HNF1A[0] == 0 && HNF4A[0] == 1 && HNF6[0] == 1 && SNAI1[0] == 1 && SNAI2[0] == 0 || E2F[0] == 0 && Bcatenin[0] == 1 && YAP1[0] == 1 && HNF1A[0] == 1 && HNF4A[0] == 0 && NFkB[0] == 0 && SNAI1[0] == 0 && SNAI2[0] == 0 || E2F[0] == 0 && Bcatenin[0] == 1 && YAP1[0] == 1 && HNF1A[0] == 1 && HNF4A[0] == 0 && NFkB[0] == 1 && SNAI1[0] == 0 && SNAI2[0] == 0 && p53[0] == 0 || E2F[0] == 0 && Bcatenin[0] == 1 && YAP1[0] == 1 && HNF1A[0] == 1 && HNF4A[0] == 1 && SNAI1[0] == 0 || E2F[0] == 0 && Bcatenin[0] == 1 && YAP1[0] == 1 && HNF1A[0] == 1 && HNF4A[0] == 1 && SNAI1[0] == 1 && SNAI2[0] == 0 || E2F[0] == 1 && YAP1[0] == 0 && HNF1A[0] == 0 && HNF4A[0] == 0 && HNF6[0] == 0 && FOXA2[0] == 0 && SNAI1[0] == 0 && ZEB1[0] == 0 && SNAI2[0] == 0 && SOX9[0] == 1 && NANOG[0] == 0 && SOX2[0] == 1 && p53[0] == 1 || E2F[0] == 1 && YAP1[0] == 0 && HNF1A[0] == 0 && HNF4A[0] == 0 && HNF6[0] == 0 && FOXA2[0] == 1 && SNAI1[0] == 0 && SNAI2[0] == 0 || E2F[0] == 1 && YAP1[0] == 0 && HNF1A[0] == 0 && HNF4A[0] == 0 && HNF6[0] == 1 && SNAI1[0] == 0 && SNAI2[0] == 0 || E2F[0] == 1 && YAP1[0] == 0 && HNF1A[0] == 0 && HNF4A[0] == 1 && HNF6[0] == 0 && FOXA2[0] == 0 && SNAI1[0] == 0 && SNAI2[0] == 0 || E2F[0] == 1 && YAP1[0] == 0 && HNF1A[0] == 0 && HNF4A[0] == 1 && HNF6[0] == 0 && FOXA2[0] == 1 && SNAI1[0] == 0 || E2F[0] == 1 && YAP1[0] == 0 && HNF1A[0] == 0 && HNF4A[0] == 1 && HNF6[0] == 0 && FOXA2[0] == 1 && SNAI1[0] == 1 && SNAI2[0] == 0 || E2F[0] == 1 && YAP1[0] == 0 && HNF1A[0] == 0 && HNF4A[0] == 1 && HNF6[0] == 1 && SNAI1[0] == 0 || E2F[0] == 1 && YAP1[0] == 0 && HNF1A[0] == 0 && HNF4A[0] == 1 && HNF6[0] == 1 && SNAI1[0] == 1 && SNAI2[0] == 0 || E2F[0] == 1 && YAP1[0] == 0 && HNF1A[0] == 1 && HNF4A[0] == 0 && HNF6[0] == 0 && FOXA2[0] == 0 && SNAI1[0] == 0 && SNAI2[0] == 0 || E2F[0] == 1 && YAP1[0] == 0 && HNF1A[0] == 1 && HNF4A[0] == 0 && HNF6[0] == 0 && FOXA2[0] == 1 && NFkB[0] == 0 && SNAI1[0] == 0 || E2F[0] == 1 && YAP1[0] == 0 && HNF1A[0] == 1 && HNF4A[0] == 0 && HNF6[0] == 0 && FOXA2[0] == 1 && NFkB[0] == 0 && SNAI1[0] == 1 && SNAI2[0] == 0 || E2F[0] == 1 && YAP1[0] == 0 && HNF1A[0] == 1 && HNF4A[0] == 0 && HNF6[0] == 0 && FOXA2[0] == 1 && NFkB[0] == 1 && SNAI1[0] == 0 && SNAI2[0] == 0 || E2F[0] == 1 && YAP1[0] == 0 && HNF1A[0] == 1 && HNF4A[0] == 0 && HNF6[0] == 0 && FOXA2[0] == 1 && NFkB[0] == 1 && SNAI1[0] == 0 && SNAI2[0] == 1 && p53[0] == 0 || E2F[0] == 1 && YAP1[0] == 0 && HNF1A[0] == 1 && HNF4A[0] == 0 && HNF6[0] == 0 && FOXA2[0] == 1 && NFkB[0] == 1 && SNAI1[0] == 1 && SNAI2[0] == 0 && p53[0] == 0 || E2F[0] == 1 && YAP1[0] == 0 && HNF1A[0] == 1 && HNF4A[0] == 0 && HNF6[0] == 1 && NFkB[0] == 0 && SNAI1[0] == 0 || E2F[0] == 1 && YAP1[0] == 0 && HNF1A[0] == 1 && HNF4A[0] == 0 && HNF6[0] == 1 && NFkB[0] == 0 && SNAI1[0] == 1 && SNAI2[0] == 0 || E2F[0] == 1 && YAP1[0] == 0 && HNF1A[0] == 1 && HNF4A[0] == 0 && HNF6[0] == 1 && NFkB[0] == 1 && SNAI1[0] == 0 && SNAI2[0] == 0 || E2F[0] == 1 && YAP1[0] == 0 && HNF1A[0] == 1 && HNF4A[0] == 0 && HNF6[0] == 1 && NFkB[0] == 1 && SNAI1[0] == 0 && SNAI2[0] == 1 && p53[0] == 0 || E2F[0] == 1 && YAP1[0] == 0 && HNF1A[0] == 1 && HNF4A[0] == 0 && HNF6[0] == 1 && NFkB[0] == 1 && SNAI1[0] == 1 && SNAI2[0] == 0 && p53[0] == 0 || E2F[0] == 1 && YAP1[0] == 0 && HNF1A[0] == 1 && HNF4A[0] == 1 && SNAI1[0] == 0 || E2F[0] == 1 && YAP1[0] == 0 && HNF1A[0] == 1 && HNF4A[0] == 1 && SNAI1[0] == 1 && SNAI2[0] == 0 || E2F[0] == 1 && YAP1[0] == 1 && HNF1A[0] == 0 && HNF4A[0] == 0 && HNF6[0] == 0 && FOXA2[0] == 0 && NFkB[0] == 0 && SNAI1[0] == 0 && ZEB1[0] == 0 && SNAI2[0] == 0 && SOX9[0] == 1 && NANOG[0] == 0 && SOX2[0] == 1 && p53[0] == 1 || E2F[0] == 1 && YAP1[0] == 1 && HNF1A[0] == 0 && HNF4A[0] == 0 && HNF6[0] == 0 && FOXA2[0] == 1 && NFkB[0] == 0 && SNAI1[0] == 0 && SNAI2[0] == 0 || E2F[0] == 1 && YAP1[0] == 1 && HNF1A[0] == 0 && HNF4A[0] == 0 && HNF6[0] == 0 && FOXA2[0] == 1 && NFkB[0] == 1 && SNAI1[0] == 0 && SNAI2[0] == 0 && p53[0] == 0 || E2F[0] == 1 && YAP1[0] == 1 && HNF1A[0] == 0 && HNF4A[0] == 0 && HNF6[0] == 1 && NFkB[0] == 0 && SNAI1[0] == 0 && SNAI2[0] == 0 || E2F[0] == 1 && YAP1[0] == 1 && HNF1A[0] == 0 && HNF4A[0] == 0 && HNF6[0] == 1 && NFkB[0] == 1 && SNAI1[0] == 0 && SNAI2[0] == 0 && p53[0] == 0 || E2F[0] == 1 && YAP1[0] == 1 && HNF1A[0] == 0 && HNF4A[0] == 1 && HNF6[0] == 0 && FOXA2[0] == 0 && NFkB[0] == 0 && SNAI1[0] == 0 && SNAI2[0] == 0 || E2F[0] == 1 && YAP1[0] == 1 && HNF1A[0] == 0 && HNF4A[0] == 1 && HNF6[0] == 0 && FOXA2[0] == 0 && NFkB[0] == 1 && SNAI1[0] == 0 && SNAI2[0] == 0 && p53[0] == 0 || E2F[0] == 1 && YAP1[0] == 1 && HNF1A[0] == 0 && HNF4A[0] == 1 && HNF6[0] == 0 && FOXA2[0] == 1 && SNAI1[0] == 0 || E2F[0] == 1 && YAP1[0] == 1 && HNF1A[0] == 0 && HNF4A[0] == 1 && HNF6[0] == 0 && FOXA2[0] == 1 && SNAI1[0] == 1 && SNAI2[0] == 0 || E2F[0] == 1 && YAP1[0] == 1 && HNF1A[0] == 0 && HNF4A[0] == 1 && HNF6[0] == 1 && SNAI1[0] == 0 || E2F[0] == 1 && YAP1[0] == 1 && HNF1A[0] == 0 && HNF4A[0] == 1 && HNF6[0] == 1 && SNAI1[0] == 1 && SNAI2[0] == 0 || E2F[0] == 1 && YAP1[0] == 1 && HNF1A[0] == 1 && HNF4A[0] == 0 && NFkB[0] == 0 && SNAI1[0] == 0 && SNAI2[0] == 0 || E2F[0] == 1 && YAP1[0] == 1 && HNF1A[0] == 1 && HNF4A[0] == 0 && NFkB[0] == 1 && SNAI1[0] == 0 && SNAI2[0] == 0 && p53[0] == 0 || E2F[0] == 1 && YAP1[0] == 1 && HNF1A[0] == 1 && HNF4A[0] == 1 && SNAI1[0] == 0 || E2F[0] == 1 && YAP1[0] == 1 && HNF1A[0] == 1 && HNF4A[0] == 1 && SNAI1[0] == 1 && SNAI2[0] == 0) { HNF4A[0] = 1; }

else { HNF4A[0] = 0; }

//Logic rule for HNF6

if (HNF1A[0] == 0 && HNF4A[0] == 1 && HNF6[0] == 0 && SNAI1[0] == 0 && SOX9[0] == 0 && OCT4[0] == 0 && NANOG[0] == 0 && SOX2[0] == 0 || HNF1A[0] == 0 && HNF4A[0] == 1 && HNF6[0] == 0 && SNAI1[0] == 0 && SOX9[0] == 0 && OCT4[0] == 0 && NANOG[0] == 0 && SOX2[0] == 1 && p53[0] == 1 || HNF1A[0] == 0 && HNF4A[0] == 1 && HNF6[0] == 0 && SNAI1[0] == 0 && SOX9[0] == 0 && OCT4[0] == 0 && NANOG[0] == 1 || HNF1A[0] == 0 && HNF4A[0] == 1 && HNF6[0] == 0 && SNAI1[0] == 0 && SOX9[0] == 0 && OCT4[0] == 1 && NANOG[0] == 0 && p53[0] == 1 || HNF1A[0] == 0 && HNF4A[0] == 1 && HNF6[0] == 0 && SNAI1[0] == 0 && SOX9[0] == 0 && OCT4[0] == 1 && NANOG[0] == 1 && SOX2[0] == 0 || HNF1A[0] == 0 && HNF4A[0] == 1 && HNF6[0] == 0 && SNAI1[0] == 0 && SOX9[0] == 1 && OCT4[0] == 0 || HNF1A[0] == 0 && HNF4A[0] == 1 && HNF6[0] == 0 && SNAI1[0] == 0 && SOX9[0] == 1 && OCT4[0] == 1 && NANOG[0] == 0 || HNF1A[0] == 0 && HNF4A[0] == 1 && HNF6[0] == 0 && SNAI1[0] == 0 && SOX9[0] == 1 && OCT4[0] == 1 && NANOG[0] == 1 && SOX2[0] == 0 || HNF1A[0] == 0 && HNF4A[0] == 1 && HNF6[0] == 0 && SNAI1[0] == 1 && SOX9[0] == 0 && OCT4[0] == 0 && NANOG[0] == 0 && SOX2[0] == 0 || HNF1A[0] == 0 && HNF4A[0] == 1 && HNF6[0] == 0 && SNAI1[0] == 1 && SOX9[0] == 1 && OCT4[0] == 0 || HNF1A[0] == 0 && HNF4A[0] == 1 && HNF6[0] == 0 && SNAI1[0] == 1 && SOX9[0] == 1 && OCT4[0] == 1 && NANOG[0] == 0 || HNF1A[0] == 0 && HNF4A[0] == 1 && HNF6[0] == 0 && SNAI1[0] == 1 && SOX9[0] == 1 && OCT4[0] == 1 && NANOG[0] == 1 && SOX2[0] == 0 || HNF1A[0] == 0 && HNF4A[0] == 1 && HNF6[0] == 1 && OCT4[0] == 0 || HNF1A[0] == 0 && HNF4A[0] == 1 && HNF6[0] == 1 && OCT4[0] == 1 && NANOG[0] == 0 || HNF1A[0] == 0 && HNF4A[0] == 1 && HNF6[0] == 1 && OCT4[0] == 1 && NANOG[0] == 1 && SOX2[0] == 0 || HNF1A[0] == 1 && HNF6[0] == 0 && SNAI1[0] == 0 && SOX9[0] == 0 && OCT4[0] == 0 && NANOG[0] == 0 && SOX2[0] == 0 || HNF1A[0] == 1 && HNF6[0] == 0 && SNAI1[0] == 0 && SOX9[0] == 0 && OCT4[0] == 0 && NANOG[0] == 0 && SOX2[0] == 1 && p53[0] == 1 || HNF1A[0] == 1 && HNF6[0] == 0 && SNAI1[0] == 0 && SOX9[0] == 0 && OCT4[0] == 0 && NANOG[0] == 1 || HNF1A[0] == 1 && HNF6[0] == 0 && SNAI1[0] == 0 && SOX9[0] == 0 && OCT4[0] == 1 && NANOG[0] == 0 && p53[0] == 1 || HNF1A[0] == 1 && HNF6[0] == 0 && SNAI1[0] == 0 && SOX9[0] == 0 && OCT4[0] == 1 && NANOG[0] == 1 && SOX2[0] == 0 || HNF1A[0] == 1 && HNF6[0] == 0 && SNAI1[0] == 0 && SOX9[0] == 1 && OCT4[0] == 0 || HNF1A[0] == 1 && HNF6[0] == 0 && SNAI1[0] == 0 && SOX9[0] == 1 && OCT4[0] == 1 && NANOG[0] == 0 || HNF1A[0] == 1 && HNF6[0] == 0 && SNAI1[0] == 0 && SOX9[0] == 1 && OCT4[0] == 1 && NANOG[0] == 1 && SOX2[0] == 0 || HNF1A[0] == 1 && HNF6[0] == 0 && SNAI1[0] == 1 && SOX9[0] == 0 && OCT4[0] == 0 && NANOG[0] == 0 && SOX2[0] == 0 || HNF1A[0] == 1 && HNF6[0] == 0 && SNAI1[0] == 1 && SOX9[0] == 1 && OCT4[0] == 0 || HNF1A[0] == 1 && HNF6[0] == 0 && SNAI1[0] == 1 && SOX9[0] == 1 && OCT4[0] == 1 && NANOG[0] == 0 || HNF1A[0] == 1 && HNF6[0] == 0 && SNAI1[0] == 1 && SOX9[0] == 1 && OCT4[0] == 1 && NANOG[0] == 1 && SOX2[0] == 0 || HNF1A[0] == 1 && HNF6[0] == 1 && OCT4[0] == 0 || HNF1A[0] == 1 && HNF6[0] == 1 && OCT4[0] == 1 && NANOG[0] == 0 || HNF1A[0] == 1 && HNF6[0] == 1 && OCT4[0] == 1 && NANOG[0] == 1 && SOX2[0] == 0) { HNF6[0] = 1; }

else { HNF6[0] = 0; }

//Logic rule for HNF1A

if (HNF1A[0] == 0 && HNF4A[0] == 0 && FOXA2[0] == 1 && NFkB[0] == 0 && SNAI1[0] == 0 || HNF1A[0] == 0 && HNF4A[0] == 1 && FOXA2[0] == 0 && SNAI1[0] == 0 || HNF1A[0] == 0 && HNF4A[0] == 1 && FOXA2[0] == 1 || HNF1A[0] == 1 && HNF4A[0] == 0 && NFkB[0] == 0 && SNAI1[0] == 0 || HNF1A[0] == 1 && HNF4A[0] == 1) { HNF1A[0] = 1; }

else { HNF1A[0] = 0; }

//Logic rule for FOXA2

if (Bcatenin[0] == 0 && HNF4A[0] == 0 && HNF6[0] == 1 && FOXA2[0] == 0 && SOX9[0] == 1 && SOX2[0] == 0 || Bcatenin[0] == 0 && HNF4A[0] == 0 && HNF6[0] == 1 && FOXA2[0] == 1 && SOX2[0] == 0 || Bcatenin[0] == 0 && HNF4A[0] == 1 && HNF6[0] == 0 && FOXA2[0] == 0 && ZEB1[0] == 0 && SNAI2[0] == 0 && TGFB[0] == 0 && SOX9[0] == 1 && SOX2[0] == 0 || Bcatenin[0] == 0 && HNF4A[0] == 1 && HNF6[0] == 0 && FOXA2[0] == 0 && ZEB1[0] == 0 && SNAI2[0] == 1 && SOX9[0] == 1 && SOX2[0] == 0 || Bcatenin[0] == 0 && HNF4A[0] == 1 && HNF6[0] == 0 && FOXA2[0] == 0 && ZEB1[0] == 1 && SOX9[0] == 1 && SOX2[0] == 0 || Bcatenin[0] == 0 && HNF4A[0] == 1 && HNF6[0] == 0 && FOXA2[0] == 1 && ZEB1[0] == 0 && SNAI2[0] == 0 && TGFB[0] == 0 && SOX2[0] == 0 || Bcatenin[0] == 0 && HNF4A[0] == 1 && HNF6[0] == 0 && FOXA2[0] == 1 && ZEB1[0] == 0 && SNAI2[0] == 1 && SOX2[0] == 0 || Bcatenin[0] == 0 && HNF4A[0] == 1 && HNF6[0] == 0 && FOXA2[0] == 1 && ZEB1[0] == 1 && SOX2[0] == 0 || Bcatenin[0] == 0 && HNF4A[0] == 1 && HNF6[0] == 1 && FOXA2[0] == 0 && ZEB1[0] == 0 && SNAI2[0] == 0 && TGFB[0] == 0 && SOX2[0] == 0 || Bcatenin[0] == 0 && HNF4A[0] == 1 && HNF6[0] == 1 && FOXA2[0] == 0 && ZEB1[0] == 0 && SNAI2[0] == 1 && TGFB[0] == 0 && SOX2[0] == 0 || Bcatenin[0] == 0 && HNF4A[0] == 1 && HNF6[0] == 1 && FOXA2[0] == 0 && ZEB1[0] == 0 && SNAI2[0] == 1 && TGFB[0] == 1 && SOX9[0] == 1 && SOX2[0] == 0 || Bcatenin[0] == 0 && HNF4A[0] == 1 && HNF6[0] == 1 && FOXA2[0] == 0 && ZEB1[0] == 1 && TGFB[0] == 0 && SOX2[0] == 0 || Bcatenin[0] == 0 && HNF4A[0] == 1 && HNF6[0] == 1 && FOXA2[0] == 0 && ZEB1[0] == 1 && TGFB[0] == 1 && SOX9[0] == 1 && SOX2[0] == 0 || Bcatenin[0] == 0 && HNF4A[0] == 1 && HNF6[0] == 1 && FOXA2[0] == 1 && ZEB1[0] == 0 && SNAI2[0] == 0 && TGFB[0] == 0 && SOX2[0] == 0 || Bcatenin[0] == 0 && HNF4A[0] == 1 && HNF6[0] == 1 && FOXA2[0] == 1 && ZEB1[0] == 0 && SNAI2[0] == 1 && SOX2[0] == 0 || Bcatenin[0] == 0 && HNF4A[0] == 1 && HNF6[0] == 1 && FOXA2[0] == 1 && ZEB1[0] == 1 && SOX2[0] == 0 || Bcatenin[0] == 1 && HNF4A[0] == 0 && HNF6[0] == 1 && SOX2[0] == 0 || Bcatenin[0] == 1 && HNF4A[0] == 1 && ZEB1[0] == 0 && SNAI2[0] == 0 && TGFB[0] == 0 && SOX2[0] == 0 || Bcatenin[0] == 1 && HNF4A[0] == 1 && ZEB1[0] == 0 && SNAI2[0] == 1 && SOX2[0] == 0 || Bcatenin[0] == 1 && HNF4A[0] == 1 && ZEB1[0] == 1 && SOX2[0] == 0) { FOXA2[0] = 1; }

else { FOXA2[0] = 0; }

//Logic rule for NFkB

if (YAP1[0] == 0 && HNF1A[0] == 0 || YAP1[0] == 0 && HNF1A[0] == 1 && HNF4A[0] == 0 || YAP1[0] == 0 && HNF1A[0] == 1 && HNF4A[0] == 1 && FOXA2[0] == 0 && NFkB[0] == 0 && SNAI1[0] == 0 && ZEB1[0] == 0 && TGFB[0] == 0 && SOX9[0] == 0 || YAP1[0] == 0 && HNF1A[0] == 1 && HNF4A[0] == 1 && FOXA2[0] == 0 && NFkB[0] == 0 && SNAI1[0] == 0 && ZEB1[0] == 0 && TGFB[0] == 0 && SOX9[0] == 1 && p53[0] == 0 || YAP1[0] == 0 && HNF1A[0] == 1 && HNF4A[0] == 1 && FOXA2[0] == 0 && NFkB[0] == 0 && SNAI1[0] == 0 && ZEB1[0] == 0 && TGFB[0] == 1 || YAP1[0] == 0 && HNF1A[0] == 1 && HNF4A[0] == 1 && FOXA2[0] == 0 && NFkB[0] == 0 && SNAI1[0] == 0 && ZEB1[0] == 1 || YAP1[0] == 0 && HNF1A[0] == 1 && HNF4A[0] == 1 && FOXA2[0] == 0 && NFkB[0] == 0 && SNAI1[0] == 1 || YAP1[0] == 0 && HNF1A[0] == 1 && HNF4A[0] == 1 && FOXA2[0] == 0 && NFkB[0] == 1 || YAP1[0] == 0 && HNF1A[0] == 1 && HNF4A[0] == 1 && FOXA2[0] == 1 && NFkB[0] == 0 && SNAI1[0] == 0 && ZEB1[0] == 0 && TGFB[0] == 0 && SOX9[0] == 0 || YAP1[0] == 0 && HNF1A[0] == 1 && HNF4A[0] == 1 && FOXA2[0] == 1 && NFkB[0] == 0 && SNAI1[0] == 0 && ZEB1[0] == 0 && TGFB[0] == 0 && SOX9[0] == 1 && p53[0] == 0 || YAP1[0] == 0 && HNF1A[0] == 1 && HNF4A[0] == 1 && FOXA2[0] == 1 && NFkB[0] == 0 && SNAI1[0] == 0 && ZEB1[0] == 0 && TGFB[0] == 1 || YAP1[0] == 0 && HNF1A[0] == 1 && HNF4A[0] == 1 && FOXA2[0] == 1 && NFkB[0] == 0 && SNAI1[0] == 0 && ZEB1[0] == 1 || YAP1[0] == 0 && HNF1A[0] == 1 && HNF4A[0] == 1 && FOXA2[0] == 1 && NFkB[0] == 0 && SNAI1[0] == 1 || YAP1[0] == 0 && HNF1A[0] == 1 && HNF4A[0] == 1 && FOXA2[0] == 1 && NFkB[0] == 1 && SNAI1[0] == 0 && ZEB1[0] == 0 && SOX9[0] == 0 || YAP1[0] == 0 && HNF1A[0] == 1 && HNF4A[0] == 1 && FOXA2[0] == 1 && NFkB[0] == 1 && SNAI1[0] == 0 && ZEB1[0] == 0 && SOX9[0] == 1 && p53[0] == 0 || YAP1[0] == 0 && HNF1A[0] == 1 && HNF4A[0] == 1 && FOXA2[0] == 1 && NFkB[0] == 1 && SNAI1[0] == 0 && ZEB1[0] == 1 || YAP1[0] == 0 && HNF1A[0] == 1 && HNF4A[0] == 1 && FOXA2[0] == 1 && NFkB[0] == 1 && SNAI1[0] == 1 || YAP1[0] == 1) { NFkB[0] = 1; }

else { NFkB[0] = 0; }

//Logic rule for SNAI1

if (E2F[0] == 0 && Bcatenin[0] == 0 && YAP1[0] == 0 && HNF1A[0] == 0 && HNF4A[0] == 0 && NFkB[0] == 0 && SNAI1[0] == 0 && ZEB1[0] == 0 && TGFB[0] == 1 && p53[0] == 0 || E2F[0] == 0 && Bcatenin[0] == 0 && YAP1[0] == 0 && HNF1A[0] == 0 && HNF4A[0] == 0 && NFkB[0] == 0 && SNAI1[0] == 0 && ZEB1[0] == 1 && TGFB[0] == 1 || E2F[0] == 0 && Bcatenin[0] == 0 && YAP1[0] == 0 && HNF1A[0] == 0 && HNF4A[0] == 0 && NFkB[0] == 0 && SNAI1[0] == 1 && SNAI2[0] == 0 && TGFB[0] == 1 || E2F[0] == 0 && Bcatenin[0] == 0 && YAP1[0] == 0 && HNF1A[0] == 0 && HNF4A[0] == 0 && NFkB[0] == 0 && SNAI1[0] == 1 && SNAI2[0] == 1 && TGFB[0] == 1 && NANOG[0] == 0 && SOX2[0] == 0 || E2F[0] == 0 && Bcatenin[0] == 0 && YAP1[0] == 0 && HNF1A[0] == 0 && HNF4A[0] == 0 && NFkB[0] == 0 && SNAI1[0] == 1 && SNAI2[0] == 1 && TGFB[0] == 1 && NANOG[0] == 1 || E2F[0] == 0 && Bcatenin[0] == 0 && YAP1[0] == 0 && HNF1A[0] == 0 && HNF4A[0] == 0 && NFkB[0] == 1 || E2F[0] == 0 && Bcatenin[0] == 0 && YAP1[0] == 0 && HNF1A[0] == 0 && HNF4A[0] == 1 && NFkB[0] == 0 && SNAI1[0] == 0 && ZEB1[0] == 1 && TGFB[0] == 1 || E2F[0] == 0 && Bcatenin[0] == 0 && YAP1[0] == 0 && HNF1A[0] == 0 && HNF4A[0] == 1 && NFkB[0] == 0 && SNAI1[0] == 1 && SNAI2[0] == 0 && TGFB[0] == 1 || E2F[0] == 0 && Bcatenin[0] == 0 && YAP1[0] == 0 && HNF1A[0] == 0 && HNF4A[0] == 1 && NFkB[0] == 0 && SNAI1[0] == 1 && SNAI2[0] == 1 && TGFB[0] == 1 && NANOG[0] == 0 && SOX2[0] == 0 || E2F[0] == 0 && Bcatenin[0] == 0 && YAP1[0] == 0 && HNF1A[0] == 0 && HNF4A[0] == 1 && NFkB[0] == 0 && SNAI1[0] == 1 && SNAI2[0] == 1 && TGFB[0] == 1 && NANOG[0] == 1 || E2F[0] == 0 && Bcatenin[0] == 0 && YAP1[0] == 0 && HNF1A[0] == 0 && HNF4A[0] == 1 && NFkB[0] == 1 && SNAI1[0] == 0 && ZEB1[0] == 1 || E2F[0] == 0 && Bcatenin[0] == 0 && YAP1[0] == 0 && HNF1A[0] == 0 && HNF4A[0] == 1 && NFkB[0] == 1 && SNAI1[0] == 1 || E2F[0] == 0 && Bcatenin[0] == 0 && YAP1[0] == 0 && HNF1A[0] == 1 && HNF4A[0] == 0 && NFkB[0] == 0 && SNAI1[0] == 0 && ZEB1[0] == 0 && TGFB[0] == 1 && p53[0] == 0 || E2F[0] == 0 && Bcatenin[0] == 0 && YAP1[0] == 0 && HNF1A[0] == 1 && HNF4A[0] == 0 && NFkB[0] == 0 && SNAI1[0] == 0 && ZEB1[0] == 1 && TGFB[0] == 1 || E2F[0] == 0 && Bcatenin[0] == 0 && YAP1[0] == 0 && HNF1A[0] == 1 && HNF4A[0] == 0 && NFkB[0] == 0 && SNAI1[0] == 1 && SNAI2[0] == 0 && TGFB[0] == 1 || E2F[0] == 0 && Bcatenin[0] == 0 && YAP1[0] == 0 && HNF1A[0] == 1 && HNF4A[0] == 0 && NFkB[0] == 0 && SNAI1[0] == 1 && SNAI2[0] == 1 && TGFB[0] == 1 && NANOG[0] == 0 && SOX2[0] == 0 || E2F[0] == 0 && Bcatenin[0] == 0 && YAP1[0] == 0 && HNF1A[0] == 1 && HNF4A[0] == 0 && NFkB[0] == 0 && SNAI1[0] == 1 && SNAI2[0] == 1 && TGFB[0] == 1 && NANOG[0] == 1 || E2F[0] == 0 && Bcatenin[0] == 0 && YAP1[0] == 0 && HNF1A[0] == 1 && HNF4A[0] == 0 && NFkB[0] == 1 && SNAI1[0] == 0 && ZEB1[0] == 0 && p53[0] == 0 || E2F[0] == 0 && Bcatenin[0] == 0 && YAP1[0] == 0 && HNF1A[0] == 1 && HNF4A[0] == 0 && NFkB[0] == 1 && SNAI1[0] == 0 && ZEB1[0] == 1 || E2F[0] == 0 && Bcatenin[0] == 0 && YAP1[0] == 0 && HNF1A[0] == 1 && HNF4A[0] == 0 && NFkB[0] == 1 && SNAI1[0] == 1 && SNAI2[0] == 0 || E2F[0] == 0 && Bcatenin[0] == 0 && YAP1[0] == 0 && HNF1A[0] == 1 && HNF4A[0] == 0 && NFkB[0] == 1 && SNAI1[0] == 1 && SNAI2[0] == 1 && NANOG[0] == 0 && SOX2[0] == 0 || E2F[0] == 0 && Bcatenin[0] == 0 && YAP1[0] == 0 && HNF1A[0] == 1 && HNF4A[0] == 0 && NFkB[0] == 1 && SNAI1[0] == 1 && SNAI2[0] == 1 && NANOG[0] == 1 || E2F[0] == 0 && Bcatenin[0] == 0 && YAP1[0] == 1 && HNF1A[0] == 0 && HNF4A[0] == 0 && NFkB[0] == 0 && SNAI1[0] == 0 && ZEB1[0] == 0 && OCT4[0] == 0 && NANOG[0] == 0 && p53[0] == 0 || E2F[0] == 0 && Bcatenin[0] == 0 && YAP1[0] == 1 && HNF1A[0] == 0 && HNF4A[0] == 0 && NFkB[0] == 0 && SNAI1[0] == 0 && ZEB1[0] == 0 && OCT4[0] == 0 && NANOG[0] == 1 || E2F[0] == 0 && Bcatenin[0] == 0 && YAP1[0] == 1 && HNF1A[0] == 0 && HNF4A[0] == 0 && NFkB[0] == 0 && SNAI1[0] == 0 && ZEB1[0] == 0 && OCT4[0] == 1 || E2F[0] == 0 && Bcatenin[0] == 0 && YAP1[0] == 1 && HNF1A[0] == 0 && HNF4A[0] == 0 && NFkB[0] == 0 && SNAI1[0] == 0 && ZEB1[0] == 1 || E2F[0] == 0 && Bcatenin[0] == 0 && YAP1[0] == 1 && HNF1A[0] == 0 && HNF4A[0] == 0 && NFkB[0] == 0 && SNAI1[0] == 1 && SNAI2[0] == 0 || E2F[0] == 0 && Bcatenin[0] == 0 && YAP1[0] == 1 && HNF1A[0] == 0 && HNF4A[0] == 0 && NFkB[0] == 0 && SNAI1[0] == 1 && SNAI2[0] == 1 && OCT4[0] == 0 && NANOG[0] == 0 && SOX2[0] == 0 || E2F[0] == 0 && Bcatenin[0] == 0 && YAP1[0] == 1 && HNF1A[0] == 0 && HNF4A[0] == 0 && NFkB[0] == 0 && SNAI1[0] == 1 && SNAI2[0] == 1 && OCT4[0] == 0 && NANOG[0] == 1 || E2F[0] == 0 && Bcatenin[0] == 0 && YAP1[0] == 1 && HNF1A[0] == 0 && HNF4A[0] == 0 && NFkB[0] == 0 && SNAI1[0] == 1 && SNAI2[0] == 1 && OCT4[0] == 1 || E2F[0] == 0 && Bcatenin[0] == 0 && YAP1[0] == 1 && HNF1A[0] == 0 && HNF4A[0] == 0 && NFkB[0] == 1 || E2F[0] == 0 && Bcatenin[0] == 0 && YAP1[0] == 1 && HNF1A[0] == 0 && HNF4A[0] == 1 && NFkB[0] == 0 && SNAI1[0] == 0 && ZEB1[0] == 0 && OCT4[0] == 0 && NANOG[0] == 1 || E2F[0] == 0 && Bcatenin[0] == 0 && YAP1[0] == 1 && HNF1A[0] == 0 && HNF4A[0] == 1 && NFkB[0] == 0 && SNAI1[0] == 0 && ZEB1[0] == 0 && OCT4[0] == 1 || E2F[0] == 0 && Bcatenin[0] == 0 && YAP1[0] == 1 && HNF1A[0] == 0 && HNF4A[0] == 1 && NFkB[0] == 0 && SNAI1[0] == 0 && ZEB1[0] == 1 || E2F[0] == 0 && Bcatenin[0] == 0 && YAP1[0] == 1 && HNF1A[0] == 0 && HNF4A[0] == 1 && NFkB[0] == 0 && SNAI1[0] == 1 && SNAI2[0] == 0 || E2F[0] == 0 && Bcatenin[0] == 0 && YAP1[0] == 1 && HNF1A[0] == 0 && HNF4A[0] == 1 && NFkB[0] == 0 && SNAI1[0] == 1 && SNAI2[0] == 1 && OCT4[0] == 0 && NANOG[0] == 0 && SOX2[0] == 0 || E2F[0] == 0 && Bcatenin[0] == 0 && YAP1[0] == 1 && HNF1A[0] == 0 && HNF4A[0] == 1 && NFkB[0] == 0 && SNAI1[0] == 1 && SNAI2[0] == 1 && OCT4[0] == 0 && NANOG[0] == 1 || E2F[0] == 0 && Bcatenin[0] == 0 && YAP1[0] == 1 && HNF1A[0] == 0 && HNF4A[0] == 1 && NFkB[0] == 0 && SNAI1[0] == 1 && SNAI2[0] == 1 && OCT4[0] == 1 || E2F[0] == 0 && Bcatenin[0] == 0 && YAP1[0] == 1 && HNF1A[0] == 0 && HNF4A[0] == 1 && NFkB[0] == 1 && SNAI1[0] == 0 && ZEB1[0] == 0 && TGFB[0] == 0 && OCT4[0] == 0 && NANOG[0] == 1 || E2F[0] == 0 && Bcatenin[0] == 0 && YAP1[0] == 1 && HNF1A[0] == 0 && HNF4A[0] == 1 && NFkB[0] == 1 && SNAI1[0] == 0 && ZEB1[0] == 0 && TGFB[0] == 0 && OCT4[0] == 1 || E2F[0] == 0 && Bcatenin[0] == 0 && YAP1[0] == 1 && HNF1A[0] == 0 && HNF4A[0] == 1 && NFkB[0] == 1 && SNAI1[0] == 0 && ZEB1[0] == 0 && TGFB[0] == 1 || E2F[0] == 0 && Bcatenin[0] == 0 && YAP1[0] == 1 && HNF1A[0] == 0 && HNF4A[0] == 1 && NFkB[0] == 1 && SNAI1[0] == 0 && ZEB1[0] == 1 || E2F[0] == 0 && Bcatenin[0] == 0 && YAP1[0] == 1 && HNF1A[0] == 0 && HNF4A[0] == 1 && NFkB[0] == 1 && SNAI1[0] == 1 || E2F[0] == 0 && Bcatenin[0] == 0 && YAP1[0] == 1 && HNF1A[0] == 1 && HNF4A[0] == 0 && NFkB[0] == 0 && SNAI1[0] == 0 && ZEB1[0] == 0 && OCT4[0] == 0 && NANOG[0] == 0 && p53[0] == 0 || E2F[0] == 0 && Bcatenin[0] == 0 && YAP1[0] == 1 && HNF1A[0] == 1 && HNF4A[0] == 0 && NFkB[0] == 0 && SNAI1[0] == 0 && ZEB1[0] == 0 && OCT4[0] == 0 && NANOG[0] == 1 || E2F[0] == 0 && Bcatenin[0] == 0 && YAP1[0] == 1 && HNF1A[0] == 1 && HNF4A[0] == 0 && NFkB[0] == 0 && SNAI1[0] == 0 && ZEB1[0] == 0 && OCT4[0] == 1 || E2F[0] == 0 && Bcatenin[0] == 0 && YAP1[0] == 1 && HNF1A[0] == 1 && HNF4A[0] == 0 && NFkB[0] == 0 && SNAI1[0] == 0 && ZEB1[0] == 1 || E2F[0] == 0 && Bcatenin[0] == 0 && YAP1[0] == 1 && HNF1A[0] == 1 && HNF4A[0] == 0 && NFkB[0] == 0 && SNAI1[0] == 1 && SNAI2[0] == 0 || E2F[0] == 0 && Bcatenin[0] == 0 && YAP1[0] == 1 && HNF1A[0] == 1 && HNF4A[0] == 0 && NFkB[0] == 0 && SNAI1[0] == 1 && SNAI2[0] == 1 && OCT4[0] == 0 && NANOG[0] == 0 && SOX2[0] == 0 || E2F[0] == 0 && Bcatenin[0] == 0 && YAP1[0] == 1 && HNF1A[0] == 1 && HNF4A[0] == 0 && NFkB[0] == 0 && SNAI1[0] == 1 && SNAI2[0] == 1 && OCT4[0] == 0 && NANOG[0] == 1 || E2F[0] == 0 && Bcatenin[0] == 0 && YAP1[0] == 1 && HNF1A[0] == 1 && HNF4A[0] == 0 && NFkB[0] == 0 && SNAI1[0] == 1 && SNAI2[0] == 1 && OCT4[0] == 1 || E2F[0] == 0 && Bcatenin[0] == 0 && YAP1[0] == 1 && HNF1A[0] == 1 && HNF4A[0] == 0 && NFkB[0] == 1 && SNAI1[0] == 0 && ZEB1[0] == 0 && TGFB[0] == 0 && OCT4[0] == 0 && NANOG[0] == 0 && p53[0] == 0 || E2F[0] == 0 && Bcatenin[0] == 0 && YAP1[0] == 1 && HNF1A[0] == 1 && HNF4A[0] == 0 && NFkB[0] == 1 && SNAI1[0] == 0 && ZEB1[0] == 0 && TGFB[0] == 0 && OCT4[0] == 0 && NANOG[0] == 1 || E2F[0] == 0 && Bcatenin[0] == 0 && YAP1[0] == 1 && HNF1A[0] == 1 && HNF4A[0] == 0 && NFkB[0] == 1 && SNAI1[0] == 0 && ZEB1[0] == 0 && TGFB[0] == 0 && OCT4[0] == 1 || E2F[0] == 0 && Bcatenin[0] == 0 && YAP1[0] == 1 && HNF1A[0] == 1 && HNF4A[0] == 0 && NFkB[0] == 1 && SNAI1[0] == 0 && ZEB1[0] == 0 && TGFB[0] == 1 || E2F[0] == 0 && Bcatenin[0] == 0 && YAP1[0] == 1 && HNF1A[0] == 1 && HNF4A[0] == 0 && NFkB[0] == 1 && SNAI1[0] == 0 && ZEB1[0] == 1 || E2F[0] == 0 && Bcatenin[0] == 0 && YAP1[0] == 1 && HNF1A[0] == 1 && HNF4A[0] == 0 && NFkB[0] == 1 && SNAI1[0] == 1 && SNAI2[0] == 0 || E2F[0] == 0 && Bcatenin[0] == 0 && YAP1[0] == 1 && HNF1A[0] == 1 && HNF4A[0] == 0 && NFkB[0] == 1 && SNAI1[0] == 1 && SNAI2[0] == 1 && TGFB[0] == 0 && OCT4[0] == 0 && NANOG[0] == 0 && SOX2[0] == 0 || E2F[0] == 0 && Bcatenin[0] == 0 && YAP1[0] == 1 && HNF1A[0] == 1 && HNF4A[0] == 0 && NFkB[0] == 1 && SNAI1[0] == 1 && SNAI2[0] == 1 && TGFB[0] == 0 && OCT4[0] == 0 && NANOG[0] == 0 && SOX2[0] == 1 && p53[0] == 0 || E2F[0] == 0 && Bcatenin[0] == 0 && YAP1[0] == 1 && HNF1A[0] == 1 && HNF4A[0] == 0 && NFkB[0] == 1 && SNAI1[0] == 1 && SNAI2[0] == 1 && TGFB[0] == 0 && OCT4[0] == 0 && NANOG[0] == 1 || E2F[0] == 0 && Bcatenin[0] == 0 && YAP1[0] == 1 && HNF1A[0] == 1 && HNF4A[0] == 0 && NFkB[0] == 1 && SNAI1[0] == 1 && SNAI2[0] == 1 && TGFB[0] == 0 && OCT4[0] == 1 || E2F[0] == 0 && Bcatenin[0] == 0 && YAP1[0] == 1 && HNF1A[0] == 1 && HNF4A[0] == 0 && NFkB[0] == 1 && SNAI1[0] == 1 && SNAI2[0] == 1 && TGFB[0] == 1 || E2F[0] == 0 && Bcatenin[0] == 0 && YAP1[0] == 1 && HNF1A[0] == 1 && HNF4A[0] == 1 && NFkB[0] == 1 && TGFB[0] == 1 || E2F[0] == 0 && Bcatenin[0] == 1 && YAP1[0] == 0 && HNF1A[0] == 0 && HNF4A[0] == 0 && NFkB[0] == 0 && SNAI1[0] == 0 && ZEB1[0] == 0 && TGFB[0] == 0 && SOX2[0] == 1 && p53[0] == 0 || E2F[0] == 0 && Bcatenin[0] == 1 && YAP1[0] == 0 && HNF1A[0] == 0 && HNF4A[0] == 0 && NFkB[0] == 0 && SNAI1[0] == 0 && ZEB1[0] == 0 && TGFB[0] == 1 && p53[0] == 0 || E2F[0] == 0 && Bcatenin[0] == 1 && YAP1[0] == 0 && HNF1A[0] == 0 && HNF4A[0] == 0 && NFkB[0] == 0 && SNAI1[0] == 0 && ZEB1[0] == 1 && TGFB[0] == 0 && SOX2[0] == 1 && p53[0] == 0 || E2F[0] == 0 && Bcatenin[0] == 1 && YAP1[0] == 0 && HNF1A[0] == 0 && HNF4A[0] == 0 && NFkB[0] == 0 && SNAI1[0] == 0 && ZEB1[0] == 1 && TGFB[0] == 1 || E2F[0] == 0 && Bcatenin[0] == 1 && YAP1[0] == 0 && HNF1A[0] == 0 && HNF4A[0] == 0 && NFkB[0] == 0 && SNAI1[0] == 1 && SNAI2[0] == 0 && TGFB[0] == 0 && SOX2[0] == 1 && p53[0] == 0 || E2F[0] == 0 && Bcatenin[0] == 1 && YAP1[0] == 0 && HNF1A[0] == 0 && HNF4A[0] == 0 && NFkB[0] == 0 && SNAI1[0] == 1 && SNAI2[0] == 0 && TGFB[0] == 1 || E2F[0] == 0 && Bcatenin[0] == 1 && YAP1[0] == 0 && HNF1A[0] == 0 && HNF4A[0] == 0 && NFkB[0] == 0 && SNAI1[0] == 1 && SNAI2[0] == 1 && TGFB[0] == 0 && SOX2[0] == 1 && p53[0] == 0 || E2F[0] == 0 && Bcatenin[0] == 1 && YAP1[0] == 0 && HNF1A[0] == 0 && HNF4A[0] == 0 && NFkB[0] == 0 && SNAI1[0] == 1 && SNAI2[0] == 1 && TGFB[0] == 1 && NANOG[0] == 0 && SOX2[0] == 0 || E2F[0] == 0 && Bcatenin[0] == 1 && YAP1[0] == 0 && HNF1A[0] == 0 && HNF4A[0] == 0 && NFkB[0] == 0 && SNAI1[0] == 1 && SNAI2[0] == 1 && TGFB[0] == 1 && NANOG[0] == 0 && SOX2[0] == 1 && p53[0] == 0 || E2F[0] == 0 && Bcatenin[0] == 1 && YAP1[0] == 0 && HNF1A[0] == 0 && HNF4A[0] == 0 && NFkB[0] == 0 && SNAI1[0] == 1 && SNAI2[0] == 1 && TGFB[0] == 1 && NANOG[0] == 1 || E2F[0] == 0 && Bcatenin[0] == 1 && YAP1[0] == 0 && HNF1A[0] == 0 && HNF4A[0] == 0 && NFkB[0] == 1 || E2F[0] == 0 && Bcatenin[0] == 1 && YAP1[0] == 0 && HNF1A[0] == 0 && HNF4A[0] == 1 && NFkB[0] == 0 && SNAI1[0] == 0 && ZEB1[0] == 0 && TGFB[0] == 0 && OCT4[0] == 0 && NANOG[0] == 1 && SOX2[0] == 1 && p53[0] == 0 || E2F[0] == 0 && Bcatenin[0] == 1 && YAP1[0] == 0 && HNF1A[0] == 0 && HNF4A[0] == 1 && NFkB[0] == 0 && SNAI1[0] == 0 && ZEB1[0] == 0 && TGFB[0] == 0 && OCT4[0] == 1 && SOX2[0] == 1 && p53[0] == 0 || E2F[0] == 0 && Bcatenin[0] == 1 && YAP1[0] == 0 && HNF1A[0] == 0 && HNF4A[0] == 1 && NFkB[0] == 0 && SNAI1[0] == 0 && ZEB1[0] == 1 && TGFB[0] == 0 && SOX2[0] == 1 && p53[0] == 0 || E2F[0] == 0 && Bcatenin[0] == 1 && YAP1[0] == 0 && HNF1A[0] == 0 && HNF4A[0] == 1 && NFkB[0] == 0 && SNAI1[0] == 0 && ZEB1[0] == 1 && TGFB[0] == 1 || E2F[0] == 0 && Bcatenin[0] == 1 && YAP1[0] == 0 && HNF1A[0] == 0 && HNF4A[0] == 1 && NFkB[0] == 0 && SNAI1[0] == 1 && SNAI2[0] == 0 && TGFB[0] == 0 && SOX2[0] == 1 && p53[0] == 0 || E2F[0] == 0 && Bcatenin[0] == 1 && YAP1[0] == 0 && HNF1A[0] == 0 && HNF4A[0] == 1 && NFkB[0] == 0 && SNAI1[0] == 1 && SNAI2[0] == 0 && TGFB[0] == 1 || E2F[0] == 0 && Bcatenin[0] == 1 && YAP1[0] == 0 && HNF1A[0] == 0 && HNF4A[0] == 1 && NFkB[0] == 0 && SNAI1[0] == 1 && SNAI2[0] == 1 && TGFB[0] == 0 && SOX2[0] == 1 && p53[0] == 0 || E2F[0] == 0 && Bcatenin[0] == 1 && YAP1[0] == 0 && HNF1A[0] == 0 && HNF4A[0] == 1 && NFkB[0] == 0 && SNAI1[0] == 1 && SNAI2[0] == 1 && TGFB[0] == 1 && NANOG[0] == 0 && SOX2[0] == 0 || E2F[0] == 0 && Bcatenin[0] == 1 && YAP1[0] == 0 && HNF1A[0] == 0 && HNF4A[0] == 1 && NFkB[0] == 0 && SNAI1[0] == 1 && SNAI2[0] == 1 && TGFB[0] == 1 && NANOG[0] == 0 && SOX2[0] == 1 && p53[0] == 0 || E2F[0] == 0 && Bcatenin[0] == 1 && YAP1[0] == 0 && HNF1A[0] == 0 && HNF4A[0] == 1 && NFkB[0] == 0 && SNAI1[0] == 1 && SNAI2[0] == 1 && TGFB[0] == 1 && NANOG[0] == 1 || E2F[0] == 0 && Bcatenin[0] == 1 && YAP1[0] == 0 && HNF1A[0] == 0 && HNF4A[0] == 1 && NFkB[0] == 1 && SNAI1[0] == 0 && ZEB1[0] == 0 && TGFB[0] == 0 && OCT4[0] == 0 && NANOG[0] == 1 && SOX2[0] == 1 && p53[0] == 0 || E2F[0] == 0 && Bcatenin[0] == 1 && YAP1[0] == 0 && HNF1A[0] == 0 && HNF4A[0] == 1 && NFkB[0] == 1 && SNAI1[0] == 0 && ZEB1[0] == 0 && TGFB[0] == 0 && OCT4[0] == 1 && SOX2[0] == 1 && p53[0] == 0 || E2F[0] == 0 && Bcatenin[0] == 1 && YAP1[0] == 0 && HNF1A[0] == 0 && HNF4A[0] == 1 && NFkB[0] == 1 && SNAI1[0] == 0 && ZEB1[0] == 1 || E2F[0] == 0 && Bcatenin[0] == 1 && YAP1[0] == 0 && HNF1A[0] == 0 && HNF4A[0] == 1 && NFkB[0] == 1 && SNAI1[0] == 1 || E2F[0] == 0 && Bcatenin[0] == 1 && YAP1[0] == 0 && HNF1A[0] == 1 && HNF4A[0] == 0 && NFkB[0] == 0 && SNAI1[0] == 0 && ZEB1[0] == 0 && TGFB[0] == 0 && SOX2[0] == 1 && p53[0] == 0 || E2F[0] == 0 && Bcatenin[0] == 1 && YAP1[0] == 0 && HNF1A[0] == 1 && HNF4A[0] == 0 && NFkB[0] == 0 && SNAI1[0] == 0 && ZEB1[0] == 0 && TGFB[0] == 1 && p53[0] == 0 || E2F[0] == 0 && Bcatenin[0] == 1 && YAP1[0] == 0 && HNF1A[0] == 1 && HNF4A[0] == 0 && NFkB[0] == 0 && SNAI1[0] == 0 && ZEB1[0] == 1 && TGFB[0] == 0 && SOX2[0] == 1 && p53[0] == 0 || E2F[0] == 0 && Bcatenin[0] == 1 && YAP1[0] == 0 && HNF1A[0] == 1 && HNF4A[0] == 0 && NFkB[0] == 0 && SNAI1[0] == 0 && ZEB1[0] == 1 && TGFB[0] == 1 || E2F[0] == 0 && Bcatenin[0] == 1 && YAP1[0] == 0 && HNF1A[0] == 1 && HNF4A[0] == 0 && NFkB[0] == 0 && SNAI1[0] == 1 && SNAI2[0] == 0 && TGFB[0] == 0 && SOX2[0] == 1 && p53[0] == 0 || E2F[0] == 0 && Bcatenin[0] == 1 && YAP1[0] == 0 && HNF1A[0] == 1 && HNF4A[0] == 0 && NFkB[0] == 0 && SNAI1[0] == 1 && SNAI2[0] == 0 && TGFB[0] == 1 || E2F[0] == 0 && Bcatenin[0] == 1 && YAP1[0] == 0 && HNF1A[0] == 1 && HNF4A[0] == 0 && NFkB[0] == 0 && SNAI1[0] == 1 && SNAI2[0] == 1 && TGFB[0] == 0 && SOX2[0] == 1 && p53[0] == 0 || E2F[0] == 0 && Bcatenin[0] == 1 && YAP1[0] == 0 && HNF1A[0] == 1 && HNF4A[0] == 0 && NFkB[0] == 0 && SNAI1[0] == 1 && SNAI2[0] == 1 && TGFB[0] == 1 && NANOG[0] == 0 && SOX2[0] == 0 || E2F[0] == 0 && Bcatenin[0] == 1 && YAP1[0] == 0 && HNF1A[0] == 1 && HNF4A[0] == 0 && NFkB[0] == 0 && SNAI1[0] == 1 && SNAI2[0] == 1 && TGFB[0] == 1 && NANOG[0] == 0 && SOX2[0] == 1 && p53[0] == 0 || E2F[0] == 0 && Bcatenin[0] == 1 && YAP1[0] == 0 && HNF1A[0] == 1 && HNF4A[0] == 0 && NFkB[0] == 0 && SNAI1[0] == 1 && SNAI2[0] == 1 && TGFB[0] == 1 && NANOG[0] == 1 || E2F[0] == 0 && Bcatenin[0] == 1 && YAP1[0] == 0 && HNF1A[0] == 1 && HNF4A[0] == 0 && NFkB[0] == 1 && SNAI1[0] == 0 && ZEB1[0] == 0 && p53[0] == 0 || E2F[0] == 0 && Bcatenin[0] == 1 && YAP1[0] == 0 && HNF1A[0] == 1 && HNF4A[0] == 0 && NFkB[0] == 1 && SNAI1[0] == 0 && ZEB1[0] == 1 || E2F[0] == 0 && Bcatenin[0] == 1 && YAP1[0] == 0 && HNF1A[0] == 1 && HNF4A[0] == 0 && NFkB[0] == 1 && SNAI1[0] == 1 && SNAI2[0] == 0 || E2F[0] == 0 && Bcatenin[0] == 1 && YAP1[0] == 0 && HNF1A[0] == 1 && HNF4A[0] == 0 && NFkB[0] == 1 && SNAI1[0] == 1 && SNAI2[0] == 1 && NANOG[0] == 0 && SOX2[0] == 0 || E2F[0] == 0 && Bcatenin[0] == 1 && YAP1[0] == 0 && HNF1A[0] == 1 && HNF4A[0] == 0 && NFkB[0] == 1 && SNAI1[0] == 1 && SNAI2[0] == 1 && NANOG[0] == 0 && SOX2[0] == 1 && p53[0] == 0 || E2F[0] == 0 && Bcatenin[0] == 1 && YAP1[0] == 0 && HNF1A[0] == 1 && HNF4A[0] == 0 && NFkB[0] == 1 && SNAI1[0] == 1 && SNAI2[0] == 1 && NANOG[0] == 1 || E2F[0] == 0 && Bcatenin[0] == 1 && YAP1[0] == 0 && HNF1A[0] == 1 && HNF4A[0] == 1 && NFkB[0] == 1 && SNAI1[0] == 0 && ZEB1[0] == 1 && TGFB[0] == 1 && SOX2[0] == 1 && p53[0] == 0 || E2F[0] == 0 && Bcatenin[0] == 1 && YAP1[0] == 0 && HNF1A[0] == 1 && HNF4A[0] == 1 && NFkB[0] == 1 && SNAI1[0] == 1 && TGFB[0] == 1 && SOX2[0] == 1 && p53[0] == 0 || E2F[0] == 0 && Bcatenin[0] == 1 && YAP1[0] == 1 && HNF1A[0] == 0 && HNF4A[0] == 0 && NFkB[0] == 0 && SNAI1[0] == 0 && ZEB1[0] == 0 && OCT4[0] == 0 && NANOG[0] == 0 && p53[0] == 0 || E2F[0] == 0 && Bcatenin[0] == 1 && YAP1[0] == 1 && HNF1A[0] == 0 && HNF4A[0] == 0 && NFkB[0] == 0 && SNAI1[0] == 0 && ZEB1[0] == 0 && OCT4[0] == 0 && NANOG[0] == 1 || E2F[0] == 0 && Bcatenin[0] == 1 && YAP1[0] == 1 && HNF1A[0] == 0 && HNF4A[0] == 0 && NFkB[0] == 0 && SNAI1[0] == 0 && ZEB1[0] == 0 && OCT4[0] == 1 || E2F[0] == 0 && Bcatenin[0] == 1 && YAP1[0] == 1 && HNF1A[0] == 0 && HNF4A[0] == 0 && NFkB[0] == 0 && SNAI1[0] == 0 && ZEB1[0] == 1 || E2F[0] == 0 && Bcatenin[0] == 1 && YAP1[0] == 1 && HNF1A[0] == 0 && HNF4A[0] == 0 && NFkB[0] == 0 && SNAI1[0] == 1 && SNAI2[0] == 0 || E2F[0] == 0 && Bcatenin[0] == 1 && YAP1[0] == 1 && HNF1A[0] == 0 && HNF4A[0] == 0 && NFkB[0] == 0 && SNAI1[0] == 1 && SNAI2[0] == 1 && OCT4[0] == 0 && NANOG[0] == 0 && SOX2[0] == 0 || E2F[0] == 0 && Bcatenin[0] == 1 && YAP1[0] == 1 && HNF1A[0] == 0 && HNF4A[0] == 0 && NFkB[0] == 0 && SNAI1[0] == 1 && SNAI2[0] == 1 && OCT4[0] == 0 && NANOG[0] == 0 && SOX2[0] == 1 && p53[0] == 0 || E2F[0] == 0 && Bcatenin[0] == 1 && YAP1[0] == 1 && HNF1A[0] == 0 && HNF4A[0] == 0 && NFkB[0] == 0 && SNAI1[0] == 1 && SNAI2[0] == 1 && OCT4[0] == 0 && NANOG[0] == 1 || E2F[0] == 0 && Bcatenin[0] == 1 && YAP1[0] == 1 && HNF1A[0] == 0 && HNF4A[0] == 0 && NFkB[0] == 0 && SNAI1[0] == 1 && SNAI2[0] == 1 && OCT4[0] == 1 || E2F[0] == 0 && Bcatenin[0] == 1 && YAP1[0] == 1 && HNF1A[0] == 0 && HNF4A[0] == 0 && NFkB[0] == 1 || E2F[0] == 0 && Bcatenin[0] == 1 && YAP1[0] == 1 && HNF1A[0] == 0 && HNF4A[0] == 1 && NFkB[0] == 0 && SNAI1[0] == 0 && ZEB1[0] == 0 && OCT4[0] == 0 && NANOG[0] == 1 || E2F[0] == 0 && Bcatenin[0] == 1 && YAP1[0] == 1 && HNF1A[0] == 0 && HNF4A[0] == 1 && NFkB[0] == 0 && SNAI1[0] == 0 && ZEB1[0] == 0 && OCT4[0] == 1 || E2F[0] == 0 && Bcatenin[0] == 1 && YAP1[0] == 1 && HNF1A[0] == 0 && HNF4A[0] == 1 && NFkB[0] == 0 && SNAI1[0] == 0 && ZEB1[0] == 1 || E2F[0] == 0 && Bcatenin[0] == 1 && YAP1[0] == 1 && HNF1A[0] == 0 && HNF4A[0] == 1 && NFkB[0] == 0 && SNAI1[0] == 1 && SNAI2[0] == 0 || E2F[0] == 0 && Bcatenin[0] == 1 && YAP1[0] == 1 && HNF1A[0] == 0 && HNF4A[0] == 1 && NFkB[0] == 0 && SNAI1[0] == 1 && SNAI2[0] == 1 && OCT4[0] == 0 && NANOG[0] == 0 && SOX2[0] == 0 || E2F[0] == 0 && Bcatenin[0] == 1 && YAP1[0] == 1 && HNF1A[0] == 0 && HNF4A[0] == 1 && NFkB[0] == 0 && SNAI1[0] == 1 && SNAI2[0] == 1 && OCT4[0] == 0 && NANOG[0] == 0 && SOX2[0] == 1 && p53[0] == 0 || E2F[0] == 0 && Bcatenin[0] == 1 && YAP1[0] == 1 && HNF1A[0] == 0 && HNF4A[0] == 1 && NFkB[0] == 0 && SNAI1[0] == 1 && SNAI2[0] == 1 && OCT4[0] == 0 && NANOG[0] == 1 || E2F[0] == 0 && Bcatenin[0] == 1 && YAP1[0] == 1 && HNF1A[0] == 0 && HNF4A[0] == 1 && NFkB[0] == 0 && SNAI1[0] == 1 && SNAI2[0] == 1 && OCT4[0] == 1 || E2F[0] == 0 && Bcatenin[0] == 1 && YAP1[0] == 1 && HNF1A[0] == 0 && HNF4A[0] == 1 && NFkB[0] == 1 && SNAI1[0] == 0 && ZEB1[0] == 0 && TGFB[0] == 0 && OCT4[0] == 0 && NANOG[0] == 1 || E2F[0] == 0 && Bcatenin[0] == 1 && YAP1[0] == 1 && HNF1A[0] == 0 && HNF4A[0] == 1 && NFkB[0] == 1 && SNAI1[0] == 0 && ZEB1[0] == 0 && TGFB[0] == 0 && OCT4[0] == 1 || E2F[0] == 0 && Bcatenin[0] == 1 && YAP1[0] == 1 && HNF1A[0] == 0 && HNF4A[0] == 1 && NFkB[0] == 1 && SNAI1[0] == 0 && ZEB1[0] == 0 && TGFB[0] == 1 || E2F[0] == 0 && Bcatenin[0] == 1 && YAP1[0] == 1 && HNF1A[0] == 0 && HNF4A[0] == 1 && NFkB[0] == 1 && SNAI1[0] == 0 && ZEB1[0] == 1 || E2F[0] == 0 && Bcatenin[0] == 1 && YAP1[0] == 1 && HNF1A[0] == 0 && HNF4A[0] == 1 && NFkB[0] == 1 && SNAI1[0] == 1 || E2F[0] == 0 && Bcatenin[0] == 1 && YAP1[0] == 1 && HNF1A[0] == 1 && HNF4A[0] == 0 && NFkB[0] == 0 && SNAI1[0] == 0 && ZEB1[0] == 0 && OCT4[0] == 0 && NANOG[0] == 0 && p53[0] == 0 || E2F[0] == 0 && Bcatenin[0] == 1 && YAP1[0] == 1 && HNF1A[0] == 1 && HNF4A[0] == 0 && NFkB[0] == 0 && SNAI1[0] == 0 && ZEB1[0] == 0 && OCT4[0] == 0 && NANOG[0] == 1 || E2F[0] == 0 && Bcatenin[0] == 1 && YAP1[0] == 1 && HNF1A[0] == 1 && HNF4A[0] == 0 && NFkB[0] == 0 && SNAI1[0] == 0 && ZEB1[0] == 0 && OCT4[0] == 1 || E2F[0] == 0 && Bcatenin[0] == 1 && YAP1[0] == 1 && HNF1A[0] == 1 && HNF4A[0] == 0 && NFkB[0] == 0 && SNAI1[0] == 0 && ZEB1[0] == 1 || E2F[0] == 0 && Bcatenin[0] == 1 && YAP1[0] == 1 && HNF1A[0] == 1 && HNF4A[0] == 0 && NFkB[0] == 0 && SNAI1[0] == 1 && SNAI2[0] == 0 || E2F[0] == 0 && Bcatenin[0] == 1 && YAP1[0] == 1 && HNF1A[0] == 1 && HNF4A[0] == 0 && NFkB[0] == 0 && SNAI1[0] == 1 && SNAI2[0] == 1 && OCT4[0] == 0 && NANOG[0] == 0 && SOX2[0] == 0 || E2F[0] == 0 && Bcatenin[0] == 1 && YAP1[0] == 1 && HNF1A[0] == 1 && HNF4A[0] == 0 && NFkB[0] == 0 && SNAI1[0] == 1 && SNAI2[0] == 1 && OCT4[0] == 0 && NANOG[0] == 0 && SOX2[0] == 1 && p53[0] == 0 || E2F[0] == 0 && Bcatenin[0] == 1 && YAP1[0] == 1 && HNF1A[0] == 1 && HNF4A[0] == 0 && NFkB[0] == 0 && SNAI1[0] == 1 && SNAI2[0] == 1 && OCT4[0] == 0 && NANOG[0] == 1 || E2F[0] == 0 && Bcatenin[0] == 1 && YAP1[0] == 1 && HNF1A[0] == 1 && HNF4A[0] == 0 && NFkB[0] == 0 && SNAI1[0] == 1 && SNAI2[0] == 1 && OCT4[0] == 1 || E2F[0] == 0 && Bcatenin[0] == 1 && YAP1[0] == 1 && HNF1A[0] == 1 && HNF4A[0] == 0 && NFkB[0] == 1 && SNAI1[0] == 0 && ZEB1[0] == 0 && TGFB[0] == 0 && OCT4[0] == 0 && NANOG[0] == 0 && p53[0] == 0 || E2F[0] == 0 && Bcatenin[0] == 1 && YAP1[0] == 1 && HNF1A[0] == 1 && HNF4A[0] == 0 && NFkB[0] == 1 && SNAI1[0] == 0 && ZEB1[0] == 0 && TGFB[0] == 0 && OCT4[0] == 0 && NANOG[0] == 1 || E2F[0] == 0 && Bcatenin[0] == 1 && YAP1[0] == 1 && HNF1A[0] == 1 && HNF4A[0] == 0 && NFkB[0] == 1 && SNAI1[0] == 0 && ZEB1[0] == 0 && TGFB[0] == 0 && OCT4[0] == 1 || E2F[0] == 0 && Bcatenin[0] == 1 && YAP1[0] == 1 && HNF1A[0] == 1 && HNF4A[0] == 0 && NFkB[0] == 1 && SNAI1[0] == 0 && ZEB1[0] == 0 && TGFB[0] == 1 || E2F[0] == 0 && Bcatenin[0] == 1 && YAP1[0] == 1 && HNF1A[0] == 1 && HNF4A[0] == 0 && NFkB[0] == 1 && SNAI1[0] == 0 && ZEB1[0] == 1 || E2F[0] == 0 && Bcatenin[0] == 1 && YAP1[0] == 1 && HNF1A[0] == 1 && HNF4A[0] == 0 && NFkB[0] == 1 && SNAI1[0] == 1 && SNAI2[0] == 0 || E2F[0] == 0 && Bcatenin[0] == 1 && YAP1[0] == 1 && HNF1A[0] == 1 && HNF4A[0] == 0 && NFkB[0] == 1 && SNAI1[0] == 1 && SNAI2[0] == 1 && TGFB[0] == 0 && OCT4[0] == 0 && NANOG[0] == 0 && SOX2[0] == 0 || E2F[0] == 0 && Bcatenin[0] == 1 && YAP1[0] == 1 && HNF1A[0] == 1 && HNF4A[0] == 0 && NFkB[0] == 1 && SNAI1[0] == 1 && SNAI2[0] == 1 && TGFB[0] == 0 && OCT4[0] == 0 && NANOG[0] == 0 && SOX2[0] == 1 && p53[0] == 0 || E2F[0] == 0 && Bcatenin[0] == 1 && YAP1[0] == 1 && HNF1A[0] == 1 && HNF4A[0] == 0 && NFkB[0] == 1 && SNAI1[0] == 1 && SNAI2[0] == 1 && TGFB[0] == 0 && OCT4[0] == 0 && NANOG[0] == 1 || E2F[0] == 0 && Bcatenin[0] == 1 && YAP1[0] == 1 && HNF1A[0] == 1 && HNF4A[0] == 0 && NFkB[0] == 1 && SNAI1[0] == 1 && SNAI2[0] == 1 && TGFB[0] == 0 && OCT4[0] == 1 || E2F[0] == 0 && Bcatenin[0] == 1 && YAP1[0] == 1 && HNF1A[0] == 1 && HNF4A[0] == 0 && NFkB[0] == 1 && SNAI1[0] == 1 && SNAI2[0] == 1 && TGFB[0] == 1 || E2F[0] == 0 && Bcatenin[0] == 1 && YAP1[0] == 1 && HNF1A[0] == 1 && HNF4A[0] == 1 && NFkB[0] == 1 && TGFB[0] == 1 || E2F[0] == 1 && Bcatenin[0] == 0 && YAP1[0] == 0 && HNF1A[0] == 0 && HNF4A[0] == 0 && NFkB[0] == 0 && SNAI1[0] == 0 && ZEB1[0] == 0 && TGFB[0] == 0 && SOX2[0] == 1 && p53[0] == 0 || E2F[0] == 1 && Bcatenin[0] == 0 && YAP1[0] == 0 && HNF1A[0] == 0 && HNF4A[0] == 0 && NFkB[0] == 0 && SNAI1[0] == 0 && ZEB1[0] == 0 && TGFB[0] == 1 && p53[0] == 0 || E2F[0] == 1 && Bcatenin[0] == 0 && YAP1[0] == 0 && HNF1A[0] == 0 && HNF4A[0] == 0 && NFkB[0] == 0 && SNAI1[0] == 0 && ZEB1[0] == 1 && TGFB[0] == 0 && SOX2[0] == 1 && p53[0] == 0 || E2F[0] == 1 && Bcatenin[0] == 0 && YAP1[0] == 0 && HNF1A[0] == 0 && HNF4A[0] == 0 && NFkB[0] == 0 && SNAI1[0] == 0 && ZEB1[0] == 1 && TGFB[0] == 1 || E2F[0] == 1 && Bcatenin[0] == 0 && YAP1[0] == 0 && HNF1A[0] == 0 && HNF4A[0] == 0 && NFkB[0] == 0 && SNAI1[0] == 1 && SNAI2[0] == 0 && TGFB[0] == 0 && SOX2[0] == 1 && p53[0] == 0 || E2F[0] == 1 && Bcatenin[0] == 0 && YAP1[0] == 0 && HNF1A[0] == 0 && HNF4A[0] == 0 && NFkB[0] == 0 && SNAI1[0] == 1 && SNAI2[0] == 0 && TGFB[0] == 1 || E2F[0] == 1 && Bcatenin[0] == 0 && YAP1[0] == 0 && HNF1A[0] == 0 && HNF4A[0] == 0 && NFkB[0] == 0 && SNAI1[0] == 1 && SNAI2[0] == 1 && TGFB[0] == 0 && SOX2[0] == 1 && p53[0] == 0 || E2F[0] == 1 && Bcatenin[0] == 0 && YAP1[0] == 0 && HNF1A[0] == 0 && HNF4A[0] == 0 && NFkB[0] == 0 && SNAI1[0] == 1 && SNAI2[0] == 1 && TGFB[0] == 1 && NANOG[0] == 0 && SOX2[0] == 0 || E2F[0] == 1 && Bcatenin[0] == 0 && YAP1[0] == 0 && HNF1A[0] == 0 && HNF4A[0] == 0 && NFkB[0] == 0 && SNAI1[0] == 1 && SNAI2[0] == 1 && TGFB[0] == 1 && NANOG[0] == 0 && SOX2[0] == 1 && p53[0] == 0 || E2F[0] == 1 && Bcatenin[0] == 0 && YAP1[0] == 0 && HNF1A[0] == 0 && HNF4A[0] == 0 && NFkB[0] == 0 && SNAI1[0] == 1 && SNAI2[0] == 1 && TGFB[0] == 1 && NANOG[0] == 1 || E2F[0] == 1 && Bcatenin[0] == 0 && YAP1[0] == 0 && HNF1A[0] == 0 && HNF4A[0] == 0 && NFkB[0] == 1 || E2F[0] == 1 && Bcatenin[0] == 0 && YAP1[0] == 0 && HNF1A[0] == 0 && HNF4A[0] == 1 && NFkB[0] == 0 && SNAI1[0] == 0 && ZEB1[0] == 0 && TGFB[0] == 0 && OCT4[0] == 0 && NANOG[0] == 1 && SOX2[0] == 1 && p53[0] == 0 || E2F[0] == 1 && Bcatenin[0] == 0 && YAP1[0] == 0 && HNF1A[0] == 0 && HNF4A[0] == 1 && NFkB[0] == 0 && SNAI1[0] == 0 && ZEB1[0] == 0 && TGFB[0] == 0 && OCT4[0] == 1 && SOX2[0] == 1 && p53[0] == 0 || E2F[0] == 1 && Bcatenin[0] == 0 && YAP1[0] == 0 && HNF1A[0] == 0 && HNF4A[0] == 1 && NFkB[0] == 0 && SNAI1[0] == 0 && ZEB1[0] == 1 && TGFB[0] == 0 && SOX2[0] == 1 && p53[0] == 0 || E2F[0] == 1 && Bcatenin[0] == 0 && YAP1[0] == 0 && HNF1A[0] == 0 && HNF4A[0] == 1 && NFkB[0] == 0 && SNAI1[0] == 0 && ZEB1[0] == 1 && TGFB[0] == 1 || E2F[0] == 1 && Bcatenin[0] == 0 && YAP1[0] == 0 && HNF1A[0] == 0 && HNF4A[0] == 1 && NFkB[0] == 0 && SNAI1[0] == 1 && SNAI2[0] == 0 && TGFB[0] == 0 && SOX2[0] == 1 && p53[0] == 0 || E2F[0] == 1 && Bcatenin[0] == 0 && YAP1[0] == 0 && HNF1A[0] == 0 && HNF4A[0] == 1 && NFkB[0] == 0 && SNAI1[0] == 1 && SNAI2[0] == 0 && TGFB[0] == 1 || E2F[0] == 1 && Bcatenin[0] == 0 && YAP1[0] == 0 && HNF1A[0] == 0 && HNF4A[0] == 1 && NFkB[0] == 0 && SNAI1[0] == 1 && SNAI2[0] == 1 && TGFB[0] == 0 && SOX2[0] == 1 && p53[0] == 0 || E2F[0] == 1 && Bcatenin[0] == 0 && YAP1[0] == 0 && HNF1A[0] == 0 && HNF4A[0] == 1 && NFkB[0] == 0 && SNAI1[0] == 1 && SNAI2[0] == 1 && TGFB[0] == 1 && NANOG[0] == 0 && SOX2[0] == 0 || E2F[0] == 1 && Bcatenin[0] == 0 && YAP1[0] == 0 && HNF1A[0] == 0 && HNF4A[0] == 1 && NFkB[0] == 0 && SNAI1[0] == 1 && SNAI2[0] == 1 && TGFB[0] == 1 && NANOG[0] == 0 && SOX2[0] == 1 && p53[0] == 0 || E2F[0] == 1 && Bcatenin[0] == 0 && YAP1[0] == 0 && HNF1A[0] == 0 && HNF4A[0] == 1 && NFkB[0] == 0 && SNAI1[0] == 1 && SNAI2[0] == 1 && TGFB[0] == 1 && NANOG[0] == 1 || E2F[0] == 1 && Bcatenin[0] == 0 && YAP1[0] == 0 && HNF1A[0] == 0 && HNF4A[0] == 1 && NFkB[0] == 1 && SNAI1[0] == 0 && ZEB1[0] == 0 && TGFB[0] == 0 && OCT4[0] == 0 && NANOG[0] == 1 && p53[0] == 0 || E2F[0] == 1 && Bcatenin[0] == 0 && YAP1[0] == 0 && HNF1A[0] == 0 && HNF4A[0] == 1 && NFkB[0] == 1 && SNAI1[0] == 0 && ZEB1[0] == 0 && TGFB[0] == 0 && OCT4[0] == 1 && p53[0] == 0 || E2F[0] == 1 && Bcatenin[0] == 0 && YAP1[0] == 0 && HNF1A[0] == 0 && HNF4A[0] == 1 && NFkB[0] == 1 && SNAI1[0] == 0 && ZEB1[0] == 0 && TGFB[0] == 1 && p53[0] == 0 || E2F[0] == 1 && Bcatenin[0] == 0 && YAP1[0] == 0 && HNF1A[0] == 0 && HNF4A[0] == 1 && NFkB[0] == 1 && SNAI1[0] == 0 && ZEB1[0] == 1 || E2F[0] == 1 && Bcatenin[0] == 0 && YAP1[0] == 0 && HNF1A[0] == 0 && HNF4A[0] == 1 && NFkB[0] == 1 && SNAI1[0] == 1 || E2F[0] == 1 && Bcatenin[0] == 0 && YAP1[0] == 0 && HNF1A[0] == 1 && HNF4A[0] == 0 && NFkB[0] == 0 && SNAI1[0] == 0 && ZEB1[0] == 0 && TGFB[0] == 0 && SOX2[0] == 1 && p53[0] == 0 || E2F[0] == 1 && Bcatenin[0] == 0 && YAP1[0] == 0 && HNF1A[0] == 1 && HNF4A[0] == 0 && NFkB[0] == 0 && SNAI1[0] == 0 && ZEB1[0] == 0 && TGFB[0] == 1 && p53[0] == 0 || E2F[0] == 1 && Bcatenin[0] == 0 && YAP1[0] == 0 && HNF1A[0] == 1 && HNF4A[0] == 0 && NFkB[0] == 0 && SNAI1[0] == 0 && ZEB1[0] == 1 && TGFB[0] == 0 && SOX2[0] == 1 && p53[0] == 0 || E2F[0] == 1 && Bcatenin[0] == 0 && YAP1[0] == 0 && HNF1A[0] == 1 && HNF4A[0] == 0 && NFkB[0] == 0 && SNAI1[0] == 0 && ZEB1[0] == 1 && TGFB[0] == 1 || E2F[0] == 1 && Bcatenin[0] == 0 && YAP1[0] == 0 && HNF1A[0] == 1 && HNF4A[0] == 0 && NFkB[0] == 0 && SNAI1[0] == 1 && SNAI2[0] == 0 && TGFB[0] == 0 && SOX2[0] == 1 && p53[0] == 0 || E2F[0] == 1 && Bcatenin[0] == 0 && YAP1[0] == 0 && HNF1A[0] == 1 && HNF4A[0] == 0 && NFkB[0] == 0 && SNAI1[0] == 1 && SNAI2[0] == 0 && TGFB[0] == 1 || E2F[0] == 1 && Bcatenin[0] == 0 && YAP1[0] == 0 && HNF1A[0] == 1 && HNF4A[0] == 0 && NFkB[0] == 0 && SNAI1[0] == 1 && SNAI2[0] == 1 && TGFB[0] == 0 && SOX2[0] == 1 && p53[0] == 0 || E2F[0] == 1 && Bcatenin[0] == 0 && YAP1[0] == 0 && HNF1A[0] == 1 && HNF4A[0] == 0 && NFkB[0] == 0 && SNAI1[0] == 1 && SNAI2[0] == 1 && TGFB[0] == 1 && NANOG[0] == 0 && SOX2[0] == 0 || E2F[0] == 1 && Bcatenin[0] == 0 && YAP1[0] == 0 && HNF1A[0] == 1 && HNF4A[0] == 0 && NFkB[0] == 0 && SNAI1[0] == 1 && SNAI2[0] == 1 && TGFB[0] == 1 && NANOG[0] == 0 && SOX2[0] == 1 && p53[0] == 0 || E2F[0] == 1 && Bcatenin[0] == 0 && YAP1[0] == 0 && HNF1A[0] == 1 && HNF4A[0] == 0 && NFkB[0] == 0 && SNAI1[0] == 1 && SNAI2[0] == 1 && TGFB[0] == 1 && NANOG[0] == 1 || E2F[0] == 1 && Bcatenin[0] == 0 && YAP1[0] == 0 && HNF1A[0] == 1 && HNF4A[0] == 0 && NFkB[0] == 1 && SNAI1[0] == 0 && ZEB1[0] == 0 && p53[0] == 0 || E2F[0] == 1 && Bcatenin[0] == 0 && YAP1[0] == 0 && HNF1A[0] == 1 && HNF4A[0] == 0 && NFkB[0] == 1 && SNAI1[0] == 0 && ZEB1[0] == 1 || E2F[0] == 1 && Bcatenin[0] == 0 && YAP1[0] == 0 && HNF1A[0] == 1 && HNF4A[0] == 0 && NFkB[0] == 1 && SNAI1[0] == 1 && SNAI2[0] == 0 || E2F[0] == 1 && Bcatenin[0] == 0 && YAP1[0] == 0 && HNF1A[0] == 1 && HNF4A[0] == 0 && NFkB[0] == 1 && SNAI1[0] == 1 && SNAI2[0] == 1 && NANOG[0] == 0 && SOX2[0] == 0 || E2F[0] == 1 && Bcatenin[0] == 0 && YAP1[0] == 0 && HNF1A[0] == 1 && HNF4A[0] == 0 && NFkB[0] == 1 && SNAI1[0] == 1 && SNAI2[0] == 1 && NANOG[0] == 0 && SOX2[0] == 1 && p53[0] == 0 || E2F[0] == 1 && Bcatenin[0] == 0 && YAP1[0] == 0 && HNF1A[0] == 1 && HNF4A[0] == 0 && NFkB[0] == 1 && SNAI1[0] == 1 && SNAI2[0] == 1 && NANOG[0] == 1 || E2F[0] == 1 && Bcatenin[0] == 0 && YAP1[0] == 0 && HNF1A[0] == 1 && HNF4A[0] == 1 && NFkB[0] == 1 && TGFB[0] == 1 && p53[0] == 0 || E2F[0] == 1 && Bcatenin[0] == 0 && YAP1[0] == 1 && HNF1A[0] == 0 && HNF4A[0] == 0 && NFkB[0] == 0 && SNAI1[0] == 0 && ZEB1[0] == 0 && OCT4[0] == 0 && NANOG[0] == 0 && p53[0] == 0 || E2F[0] == 1 && Bcatenin[0] == 0 && YAP1[0] == 1 && HNF1A[0] == 0 && HNF4A[0] == 0 && NFkB[0] == 0 && SNAI1[0] == 0 && ZEB1[0] == 0 && OCT4[0] == 0 && NANOG[0] == 1 || E2F[0] == 1 && Bcatenin[0] == 0 && YAP1[0] == 1 && HNF1A[0] == 0 && HNF4A[0] == 0 && NFkB[0] == 0 && SNAI1[0] == 0 && ZEB1[0] == 0 && OCT4[0] == 1 || E2F[0] == 1 && Bcatenin[0] == 0 && YAP1[0] == 1 && HNF1A[0] == 0 && HNF4A[0] == 0 && NFkB[0] == 0 && SNAI1[0] == 0 && ZEB1[0] == 1 || E2F[0] == 1 && Bcatenin[0] == 0 && YAP1[0] == 1 && HNF1A[0] == 0 && HNF4A[0] == 0 && NFkB[0] == 0 && SNAI1[0] == 1 && SNAI2[0] == 0 || E2F[0] == 1 && Bcatenin[0] == 0 && YAP1[0] == 1 && HNF1A[0] == 0 && HNF4A[0] == 0 && NFkB[0] == 0 && SNAI1[0] == 1 && SNAI2[0] == 1 && OCT4[0] == 0 && NANOG[0] == 0 && SOX2[0] == 0 || E2F[0] == 1 && Bcatenin[0] == 0 && YAP1[0] == 1 && HNF1A[0] == 0 && HNF4A[0] == 0 && NFkB[0] == 0 && SNAI1[0] == 1 && SNAI2[0] == 1 && OCT4[0] == 0 && NANOG[0] == 0 && SOX2[0] == 1 && p53[0] == 0 || E2F[0] == 1 && Bcatenin[0] == 0 && YAP1[0] == 1 && HNF1A[0] == 0 && HNF4A[0] == 0 && NFkB[0] == 0 && SNAI1[0] == 1 && SNAI2[0] == 1 && OCT4[0] == 0 && NANOG[0] == 1 || E2F[0] == 1 && Bcatenin[0] == 0 && YAP1[0] == 1 && HNF1A[0] == 0 && HNF4A[0] == 0 && NFkB[0] == 0 && SNAI1[0] == 1 && SNAI2[0] == 1 && OCT4[0] == 1 || E2F[0] == 1 && Bcatenin[0] == 0 && YAP1[0] == 1 && HNF1A[0] == 0 && HNF4A[0] == 0 && NFkB[0] == 1 || E2F[0] == 1 && Bcatenin[0] == 0 && YAP1[0] == 1 && HNF1A[0] == 0 && HNF4A[0] == 1 && NFkB[0] == 0 && SNAI1[0] == 0 && ZEB1[0] == 0 && OCT4[0] == 0 && NANOG[0] == 1 || E2F[0] == 1 && Bcatenin[0] == 0 && YAP1[0] == 1 && HNF1A[0] == 0 && HNF4A[0] == 1 && NFkB[0] == 0 && SNAI1[0] == 0 && ZEB1[0] == 0 && OCT4[0] == 1 || E2F[0] == 1 && Bcatenin[0] == 0 && YAP1[0] == 1 && HNF1A[0] == 0 && HNF4A[0] == 1 && NFkB[0] == 0 && SNAI1[0] == 0 && ZEB1[0] == 1 || E2F[0] == 1 && Bcatenin[0] == 0 && YAP1[0] == 1 && HNF1A[0] == 0 && HNF4A[0] == 1 && NFkB[0] == 0 && SNAI1[0] == 1 && SNAI2[0] == 0 || E2F[0] == 1 && Bcatenin[0] == 0 && YAP1[0] == 1 && HNF1A[0] == 0 && HNF4A[0] == 1 && NFkB[0] == 0 && SNAI1[0] == 1 && SNAI2[0] == 1 && OCT4[0] == 0 && NANOG[0] == 0 && SOX2[0] == 0 || E2F[0] == 1 && Bcatenin[0] == 0 && YAP1[0] == 1 && HNF1A[0] == 0 && HNF4A[0] == 1 && NFkB[0] == 0 && SNAI1[0] == 1 && SNAI2[0] == 1 && OCT4[0] == 0 && NANOG[0] == 0 && SOX2[0] == 1 && p53[0] == 0 || E2F[0] == 1 && Bcatenin[0] == 0 && YAP1[0] == 1 && HNF1A[0] == 0 && HNF4A[0] == 1 && NFkB[0] == 0 && SNAI1[0] == 1 && SNAI2[0] == 1 && OCT4[0] == 0 && NANOG[0] == 1 || E2F[0] == 1 && Bcatenin[0] == 0 && YAP1[0] == 1 && HNF1A[0] == 0 && HNF4A[0] == 1 && NFkB[0] == 0 && SNAI1[0] == 1 && SNAI2[0] == 1 && OCT4[0] == 1 || E2F[0] == 1 && Bcatenin[0] == 0 && YAP1[0] == 1 && HNF1A[0] == 0 && HNF4A[0] == 1 && NFkB[0] == 1 && SNAI1[0] == 0 && ZEB1[0] == 0 && TGFB[0] == 0 && OCT4[0] == 0 && NANOG[0] == 1 || E2F[0] == 1 && Bcatenin[0] == 0 && YAP1[0] == 1 && HNF1A[0] == 0 && HNF4A[0] == 1 && NFkB[0] == 1 && SNAI1[0] == 0 && ZEB1[0] == 0 && TGFB[0] == 0 && OCT4[0] == 1 || E2F[0] == 1 && Bcatenin[0] == 0 && YAP1[0] == 1 && HNF1A[0] == 0 && HNF4A[0] == 1 && NFkB[0] == 1 && SNAI1[0] == 0 && ZEB1[0] == 0 && TGFB[0] == 1 || E2F[0] == 1 && Bcatenin[0] == 0 && YAP1[0] == 1 && HNF1A[0] == 0 && HNF4A[0] == 1 && NFkB[0] == 1 && SNAI1[0] == 0 && ZEB1[0] == 1 || E2F[0] == 1 && Bcatenin[0] == 0 && YAP1[0] == 1 && HNF1A[0] == 0 && HNF4A[0] == 1 && NFkB[0] == 1 && SNAI1[0] == 1 || E2F[0] == 1 && Bcatenin[0] == 0 && YAP1[0] == 1 && HNF1A[0] == 1 && HNF4A[0] == 0 && NFkB[0] == 0 && SNAI1[0] == 0 && ZEB1[0] == 0 && OCT4[0] == 0 && NANOG[0] == 0 && p53[0] == 0 || E2F[0] == 1 && Bcatenin[0] == 0 && YAP1[0] == 1 && HNF1A[0] == 1 && HNF4A[0] == 0 && NFkB[0] == 0 && SNAI1[0] == 0 && ZEB1[0] == 0 && OCT4[0] == 0 && NANOG[0] == 1 || E2F[0] == 1 && Bcatenin[0] == 0 && YAP1[0] == 1 && HNF1A[0] == 1 && HNF4A[0] == 0 && NFkB[0] == 0 && SNAI1[0] == 0 && ZEB1[0] == 0 && OCT4[0] == 1 || E2F[0] == 1 && Bcatenin[0] == 0 && YAP1[0] == 1 && HNF1A[0] == 1 && HNF4A[0] == 0 && NFkB[0] == 0 && SNAI1[0] == 0 && ZEB1[0] == 1 || E2F[0] == 1 && Bcatenin[0] == 0 && YAP1[0] == 1 && HNF1A[0] == 1 && HNF4A[0] == 0 && NFkB[0] == 0 && SNAI1[0] == 1 && SNAI2[0] == 0 || E2F[0] == 1 && Bcatenin[0] == 0 && YAP1[0] == 1 && HNF1A[0] == 1 && HNF4A[0] == 0 && NFkB[0] == 0 && SNAI1[0] == 1 && SNAI2[0] == 1 && OCT4[0] == 0 && NANOG[0] == 0 && SOX2[0] == 0 || E2F[0] == 1 && Bcatenin[0] == 0 && YAP1[0] == 1 && HNF1A[0] == 1 && HNF4A[0] == 0 && NFkB[0] == 0 && SNAI1[0] == 1 && SNAI2[0] == 1 && OCT4[0] == 0 && NANOG[0] == 0 && SOX2[0] == 1 && p53[0] == 0 || E2F[0] == 1 && Bcatenin[0] == 0 && YAP1[0] == 1 && HNF1A[0] == 1 && HNF4A[0] == 0 && NFkB[0] == 0 && SNAI1[0] == 1 && SNAI2[0] == 1 && OCT4[0] == 0 && NANOG[0] == 1 || E2F[0] == 1 && Bcatenin[0] == 0 && YAP1[0] == 1 && HNF1A[0] == 1 && HNF4A[0] == 0 && NFkB[0] == 0 && SNAI1[0] == 1 && SNAI2[0] == 1 && OCT4[0] == 1 || E2F[0] == 1 && Bcatenin[0] == 0 && YAP1[0] == 1 && HNF1A[0] == 1 && HNF4A[0] == 0 && NFkB[0] == 1 && SNAI1[0] == 0 && ZEB1[0] == 0 && TGFB[0] == 0 && OCT4[0] == 0 && NANOG[0] == 0 && p53[0] == 0 || E2F[0] == 1 && Bcatenin[0] == 0 && YAP1[0] == 1 && HNF1A[0] == 1 && HNF4A[0] == 0 && NFkB[0] == 1 && SNAI1[0] == 0 && ZEB1[0] == 0 && TGFB[0] == 0 && OCT4[0] == 0 && NANOG[0] == 1 || E2F[0] == 1 && Bcatenin[0] == 0 && YAP1[0] == 1 && HNF1A[0] == 1 && HNF4A[0] == 0 && NFkB[0] == 1 && SNAI1[0] == 0 && ZEB1[0] == 0 && TGFB[0] == 0 && OCT4[0] == 1 || E2F[0] == 1 && Bcatenin[0] == 0 && YAP1[0] == 1 && HNF1A[0] == 1 && HNF4A[0] == 0 && NFkB[0] == 1 && SNAI1[0] == 0 && ZEB1[0] == 0 && TGFB[0] == 1 || E2F[0] == 1 && Bcatenin[0] == 0 && YAP1[0] == 1 && HNF1A[0] == 1 && HNF4A[0] == 0 && NFkB[0] == 1 && SNAI1[0] == 0 && ZEB1[0] == 1 || E2F[0] == 1 && Bcatenin[0] == 0 && YAP1[0] == 1 && HNF1A[0] == 1 && HNF4A[0] == 0 && NFkB[0] == 1 && SNAI1[0] == 1 && SNAI2[0] == 0 || E2F[0] == 1 && Bcatenin[0] == 0 && YAP1[0] == 1 && HNF1A[0] == 1 && HNF4A[0] == 0 && NFkB[0] == 1 && SNAI1[0] == 1 && SNAI2[0] == 1 && TGFB[0] == 0 && OCT4[0] == 0 && NANOG[0] == 0 && SOX2[0] == 0 || E2F[0] == 1 && Bcatenin[0] == 0 && YAP1[0] == 1 && HNF1A[0] == 1 && HNF4A[0] == 0 && NFkB[0] == 1 && SNAI1[0] == 1 && SNAI2[0] == 1 && TGFB[0] == 0 && OCT4[0] == 0 && NANOG[0] == 0 && SOX2[0] == 1 && p53[0] == 0 || E2F[0] == 1 && Bcatenin[0] == 0 && YAP1[0] == 1 && HNF1A[0] == 1 && HNF4A[0] == 0 && NFkB[0] == 1 && SNAI1[0] == 1 && SNAI2[0] == 1 && TGFB[0] == 0 && OCT4[0] == 0 && NANOG[0] == 1 || E2F[0] == 1 && Bcatenin[0] == 0 && YAP1[0] == 1 && HNF1A[0] == 1 && HNF4A[0] == 0 && NFkB[0] == 1 && SNAI1[0] == 1 && SNAI2[0] == 1 && TGFB[0] == 0 && OCT4[0] == 1 || E2F[0] == 1 && Bcatenin[0] == 0 && YAP1[0] == 1 && HNF1A[0] == 1 && HNF4A[0] == 0 && NFkB[0] == 1 && SNAI1[0] == 1 && SNAI2[0] == 1 && TGFB[0] == 1 || E2F[0] == 1 && Bcatenin[0] == 0 && YAP1[0] == 1 && HNF1A[0] == 1 && HNF4A[0] == 1 && NFkB[0] == 1 && TGFB[0] == 1 || E2F[0] == 1 && Bcatenin[0] == 1 && YAP1[0] == 0 && HNF1A[0] == 0 && HNF4A[0] == 0 && NFkB[0] == 0 && SNAI1[0] == 0 && ZEB1[0] == 0 && p53[0] == 0 || E2F[0] == 1 && Bcatenin[0] == 1 && YAP1[0] == 0 && HNF1A[0] == 0 && HNF4A[0] == 0 && NFkB[0] == 0 && SNAI1[0] == 0 && ZEB1[0] == 1 && TGFB[0] == 0 && p53[0] == 0 || E2F[0] == 1 && Bcatenin[0] == 1 && YAP1[0] == 0 && HNF1A[0] == 0 && HNF4A[0] == 0 && NFkB[0] == 0 && SNAI1[0] == 0 && ZEB1[0] == 1 && TGFB[0] == 1 || E2F[0] == 1 && Bcatenin[0] == 1 && YAP1[0] == 0 && HNF1A[0] == 0 && HNF4A[0] == 0 && NFkB[0] == 0 && SNAI1[0] == 1 && SNAI2[0] == 0 && TGFB[0] == 0 && p53[0] == 0 || E2F[0] == 1 && Bcatenin[0] == 1 && YAP1[0] == 0 && HNF1A[0] == 0 && HNF4A[0] == 0 && NFkB[0] == 0 && SNAI1[0] == 1 && SNAI2[0] == 0 && TGFB[0] == 1 || E2F[0] == 1 && Bcatenin[0] == 1 && YAP1[0] == 0 && HNF1A[0] == 0 && HNF4A[0] == 0 && NFkB[0] == 0 && SNAI1[0] == 1 && SNAI2[0] == 1 && TGFB[0] == 0 && p53[0] == 0 || E2F[0] == 1 && Bcatenin[0] == 1 && YAP1[0] == 0 && HNF1A[0] == 0 && HNF4A[0] == 0 && NFkB[0] == 0 && SNAI1[0] == 1 && SNAI2[0] == 1 && TGFB[0] == 1 && NANOG[0] == 0 && SOX2[0] == 0 || E2F[0] == 1 && Bcatenin[0] == 1 && YAP1[0] == 0 && HNF1A[0] == 0 && HNF4A[0] == 0 && NFkB[0] == 0 && SNAI1[0] == 1 && SNAI2[0] == 1 && TGFB[0] == 1 && NANOG[0] == 0 && SOX2[0] == 1 && p53[0] == 0 || E2F[0] == 1 && Bcatenin[0] == 1 && YAP1[0] == 0 && HNF1A[0] == 0 && HNF4A[0] == 0 && NFkB[0] == 0 && SNAI1[0] == 1 && SNAI2[0] == 1 && TGFB[0] == 1 && NANOG[0] == 1 || E2F[0] == 1 && Bcatenin[0] == 1 && YAP1[0] == 0 && HNF1A[0] == 0 && HNF4A[0] == 0 && NFkB[0] == 1 || E2F[0] == 1 && Bcatenin[0] == 1 && YAP1[0] == 0 && HNF1A[0] == 0 && HNF4A[0] == 1 && NFkB[0] == 0 && SNAI1[0] == 0 && ZEB1[0] == 0 && TGFB[0] == 0 && OCT4[0] == 0 && NANOG[0] == 1 && p53[0] == 0 || E2F[0] == 1 && Bcatenin[0] == 1 && YAP1[0] == 0 && HNF1A[0] == 0 && HNF4A[0] == 1 && NFkB[0] == 0 && SNAI1[0] == 0 && ZEB1[0] == 0 && TGFB[0] == 0 && OCT4[0] == 1 && p53[0] == 0 || E2F[0] == 1 && Bcatenin[0] == 1 && YAP1[0] == 0 && HNF1A[0] == 0 && HNF4A[0] == 1 && NFkB[0] == 0 && SNAI1[0] == 0 && ZEB1[0] == 1 && TGFB[0] == 0 && p53[0] == 0 || E2F[0] == 1 && Bcatenin[0] == 1 && YAP1[0] == 0 && HNF1A[0] == 0 && HNF4A[0] == 1 && NFkB[0] == 0 && SNAI1[0] == 0 && ZEB1[0] == 1 && TGFB[0] == 1 || E2F[0] == 1 && Bcatenin[0] == 1 && YAP1[0] == 0 && HNF1A[0] == 0 && HNF4A[0] == 1 && NFkB[0] == 0 && SNAI1[0] == 1 && SNAI2[0] == 0 && TGFB[0] == 0 && p53[0] == 0 || E2F[0] == 1 && Bcatenin[0] == 1 && YAP1[0] == 0 && HNF1A[0] == 0 && HNF4A[0] == 1 && NFkB[0] == 0 && SNAI1[0] == 1 && SNAI2[0] == 0 && TGFB[0] == 1 || E2F[0] == 1 && Bcatenin[0] == 1 && YAP1[0] == 0 && HNF1A[0] == 0 && HNF4A[0] == 1 && NFkB[0] == 0 && SNAI1[0] == 1 && SNAI2[0] == 1 && TGFB[0] == 0 && p53[0] == 0 || E2F[0] == 1 && Bcatenin[0] == 1 && YAP1[0] == 0 && HNF1A[0] == 0 && HNF4A[0] == 1 && NFkB[0] == 0 && SNAI1[0] == 1 && SNAI2[0] == 1 && TGFB[0] == 1 && NANOG[0] == 0 && SOX2[0] == 0 || E2F[0] == 1 && Bcatenin[0] == 1 && YAP1[0] == 0 && HNF1A[0] == 0 && HNF4A[0] == 1 && NFkB[0] == 0 && SNAI1[0] == 1 && SNAI2[0] == 1 && TGFB[0] == 1 && NANOG[0] == 0 && SOX2[0] == 1 && p53[0] == 0 || E2F[0] == 1 && Bcatenin[0] == 1 && YAP1[0] == 0 && HNF1A[0] == 0 && HNF4A[0] == 1 && NFkB[0] == 0 && SNAI1[0] == 1 && SNAI2[0] == 1 && TGFB[0] == 1 && NANOG[0] == 1 || E2F[0] == 1 && Bcatenin[0] == 1 && YAP1[0] == 0 && HNF1A[0] == 0 && HNF4A[0] == 1 && NFkB[0] == 1 && SNAI1[0] == 0 && ZEB1[0] == 0 && TGFB[0] == 0 && OCT4[0] == 0 && NANOG[0] == 1 && p53[0] == 0 || E2F[0] == 1 && Bcatenin[0] == 1 && YAP1[0] == 0 && HNF1A[0] == 0 && HNF4A[0] == 1 && NFkB[0] == 1 && SNAI1[0] == 0 && ZEB1[0] == 0 && TGFB[0] == 0 && OCT4[0] == 1 && p53[0] == 0 || E2F[0] == 1 && Bcatenin[0] == 1 && YAP1[0] == 0 && HNF1A[0] == 0 && HNF4A[0] == 1 && NFkB[0] == 1 && SNAI1[0] == 0 && ZEB1[0] == 0 && TGFB[0] == 1 && p53[0] == 0 || E2F[0] == 1 && Bcatenin[0] == 1 && YAP1[0] == 0 && HNF1A[0] == 0 && HNF4A[0] == 1 && NFkB[0] == 1 && SNAI1[0] == 0 && ZEB1[0] == 1 || E2F[0] == 1 && Bcatenin[0] == 1 && YAP1[0] == 0 && HNF1A[0] == 0 && HNF4A[0] == 1 && NFkB[0] == 1 && SNAI1[0] == 1 || E2F[0] == 1 && Bcatenin[0] == 1 && YAP1[0] == 0 && HNF1A[0] == 1 && HNF4A[0] == 0 && NFkB[0] == 0 && SNAI1[0] == 0 && ZEB1[0] == 0 && p53[0] == 0 || E2F[0] == 1 && Bcatenin[0] == 1 && YAP1[0] == 0 && HNF1A[0] == 1 && HNF4A[0] == 0 && NFkB[0] == 0 && SNAI1[0] == 0 && ZEB1[0] == 1 && TGFB[0] == 0 && p53[0] == 0 || E2F[0] == 1 && Bcatenin[0] == 1 && YAP1[0] == 0 && HNF1A[0] == 1 && HNF4A[0] == 0 && NFkB[0] == 0 && SNAI1[0] == 0 && ZEB1[0] == 1 && TGFB[0] == 1 || E2F[0] == 1 && Bcatenin[0] == 1 && YAP1[0] == 0 && HNF1A[0] == 1 && HNF4A[0] == 0 && NFkB[0] == 0 && SNAI1[0] == 1 && SNAI2[0] == 0 && TGFB[0] == 0 && p53[0] == 0 || E2F[0] == 1 && Bcatenin[0] == 1 && YAP1[0] == 0 && HNF1A[0] == 1 && HNF4A[0] == 0 && NFkB[0] == 0 && SNAI1[0] == 1 && SNAI2[0] == 0 && TGFB[0] == 1 || E2F[0] == 1 && Bcatenin[0] == 1 && YAP1[0] == 0 && HNF1A[0] == 1 && HNF4A[0] == 0 && NFkB[0] == 0 && SNAI1[0] == 1 && SNAI2[0] == 1 && TGFB[0] == 0 && p53[0] == 0 || E2F[0] == 1 && Bcatenin[0] == 1 && YAP1[0] == 0 && HNF1A[0] == 1 && HNF4A[0] == 0 && NFkB[0] == 0 && SNAI1[0] == 1 && SNAI2[0] == 1 && TGFB[0] == 1 && NANOG[0] == 0 && SOX2[0] == 0 || E2F[0] == 1 && Bcatenin[0] == 1 && YAP1[0] == 0 && HNF1A[0] == 1 && HNF4A[0] == 0 && NFkB[0] == 0 && SNAI1[0] == 1 && SNAI2[0] == 1 && TGFB[0] == 1 && NANOG[0] == 0 && SOX2[0] == 1 && p53[0] == 0 || E2F[0] == 1 && Bcatenin[0] == 1 && YAP1[0] == 0 && HNF1A[0] == 1 && HNF4A[0] == 0 && NFkB[0] == 0 && SNAI1[0] == 1 && SNAI2[0] == 1 && TGFB[0] == 1 && NANOG[0] == 1 || E2F[0] == 1 && Bcatenin[0] == 1 && YAP1[0] == 0 && HNF1A[0] == 1 && HNF4A[0] == 0 && NFkB[0] == 1 && SNAI1[0] == 0 && ZEB1[0] == 0 && p53[0] == 0 || E2F[0] == 1 && Bcatenin[0] == 1 && YAP1[0] == 0 && HNF1A[0] == 1 && HNF4A[0] == 0 && NFkB[0] == 1 && SNAI1[0] == 0 && ZEB1[0] == 1 || E2F[0] == 1 && Bcatenin[0] == 1 && YAP1[0] == 0 && HNF1A[0] == 1 && HNF4A[0] == 0 && NFkB[0] == 1 && SNAI1[0] == 1 && SNAI2[0] == 0 || E2F[0] == 1 && Bcatenin[0] == 1 && YAP1[0] == 0 && HNF1A[0] == 1 && HNF4A[0] == 0 && NFkB[0] == 1 && SNAI1[0] == 1 && SNAI2[0] == 1 && NANOG[0] == 0 && SOX2[0] == 0 || E2F[0] == 1 && Bcatenin[0] == 1 && YAP1[0] == 0 && HNF1A[0] == 1 && HNF4A[0] == 0 && NFkB[0] == 1 && SNAI1[0] == 1 && SNAI2[0] == 1 && NANOG[0] == 0 && SOX2[0] == 1 && p53[0] == 0 || E2F[0] == 1 && Bcatenin[0] == 1 && YAP1[0] == 0 && HNF1A[0] == 1 && HNF4A[0] == 0 && NFkB[0] == 1 && SNAI1[0] == 1 && SNAI2[0] == 1 && NANOG[0] == 1 || E2F[0] == 1 && Bcatenin[0] == 1 && YAP1[0] == 0 && HNF1A[0] == 1 && HNF4A[0] == 1 && NFkB[0] == 1 && TGFB[0] == 1 && p53[0] == 0 || E2F[0] == 1 && Bcatenin[0] == 1 && YAP1[0] == 1 && HNF1A[0] == 0 && HNF4A[0] == 0 && NFkB[0] == 0 && SNAI1[0] == 0 && ZEB1[0] == 0 && OCT4[0] == 0 && NANOG[0] == 0 && p53[0] == 0 || E2F[0] == 1 && Bcatenin[0] == 1 && YAP1[0] == 1 && HNF1A[0] == 0 && HNF4A[0] == 0 && NFkB[0] == 0 && SNAI1[0] == 0 && ZEB1[0] == 0 && OCT4[0] == 0 && NANOG[0] == 1 || E2F[0] == 1 && Bcatenin[0] == 1 && YAP1[0] == 1 && HNF1A[0] == 0 && HNF4A[0] == 0 && NFkB[0] == 0 && SNAI1[0] == 0 && ZEB1[0] == 0 && OCT4[0] == 1 || E2F[0] == 1 && Bcatenin[0] == 1 && YAP1[0] == 1 && HNF1A[0] == 0 && HNF4A[0] == 0 && NFkB[0] == 0 && SNAI1[0] == 0 && ZEB1[0] == 1 || E2F[0] == 1 && Bcatenin[0] == 1 && YAP1[0] == 1 && HNF1A[0] == 0 && HNF4A[0] == 0 && NFkB[0] == 0 && SNAI1[0] == 1 && SNAI2[0] == 0 || E2F[0] == 1 && Bcatenin[0] == 1 && YAP1[0] == 1 && HNF1A[0] == 0 && HNF4A[0] == 0 && NFkB[0] == 0 && SNAI1[0] == 1 && SNAI2[0] == 1 && OCT4[0] == 0 && NANOG[0] == 0 && SOX2[0] == 0 || E2F[0] == 1 && Bcatenin[0] == 1 && YAP1[0] == 1 && HNF1A[0] == 0 && HNF4A[0] == 0 && NFkB[0] == 0 && SNAI1[0] == 1 && SNAI2[0] == 1 && OCT4[0] == 0 && NANOG[0] == 0 && SOX2[0] == 1 && p53[0] == 0 || E2F[0] == 1 && Bcatenin[0] == 1 && YAP1[0] == 1 && HNF1A[0] == 0 && HNF4A[0] == 0 && NFkB[0] == 0 && SNAI1[0] == 1 && SNAI2[0] == 1 && OCT4[0] == 0 && NANOG[0] == 1 || E2F[0] == 1 && Bcatenin[0] == 1 && YAP1[0] == 1 && HNF1A[0] == 0 && HNF4A[0] == 0 && NFkB[0] == 0 && SNAI1[0] == 1 && SNAI2[0] == 1 && OCT4[0] == 1 || E2F[0] == 1 && Bcatenin[0] == 1 && YAP1[0] == 1 && HNF1A[0] == 0 && HNF4A[0] == 0 && NFkB[0] == 1 || E2F[0] == 1 && Bcatenin[0] == 1 && YAP1[0] == 1 && HNF1A[0] == 0 && HNF4A[0] == 1 && NFkB[0] == 0 && SNAI1[0] == 0 && ZEB1[0] == 0 && OCT4[0] == 0 && NANOG[0] == 1 || E2F[0] == 1 && Bcatenin[0] == 1 && YAP1[0] == 1 && HNF1A[0] == 0 && HNF4A[0] == 1 && NFkB[0] == 0 && SNAI1[0] == 0 && ZEB1[0] == 0 && OCT4[0] == 1 || E2F[0] == 1 && Bcatenin[0] == 1 && YAP1[0] == 1 && HNF1A[0] == 0 && HNF4A[0] == 1 && NFkB[0] == 0 && SNAI1[0] == 0 && ZEB1[0] == 1 || E2F[0] == 1 && Bcatenin[0] == 1 && YAP1[0] == 1 && HNF1A[0] == 0 && HNF4A[0] == 1 && NFkB[0] == 0 && SNAI1[0] == 1 && SNAI2[0] == 0 || E2F[0] == 1 && Bcatenin[0] == 1 && YAP1[0] == 1 && HNF1A[0] == 0 && HNF4A[0] == 1 && NFkB[0] == 0 && SNAI1[0] == 1 && SNAI2[0] == 1 && OCT4[0] == 0 && NANOG[0] == 0 && SOX2[0] == 0 || E2F[0] == 1 && Bcatenin[0] == 1 && YAP1[0] == 1 && HNF1A[0] == 0 && HNF4A[0] == 1 && NFkB[0] == 0 && SNAI1[0] == 1 && SNAI2[0] == 1 && OCT4[0] == 0 && NANOG[0] == 0 && SOX2[0] == 1 && p53[0] == 0 || E2F[0] == 1 && Bcatenin[0] == 1 && YAP1[0] == 1 && HNF1A[0] == 0 && HNF4A[0] == 1 && NFkB[0] == 0 && SNAI1[0] == 1 && SNAI2[0] == 1 && OCT4[0] == 0 && NANOG[0] == 1 || E2F[0] == 1 && Bcatenin[0] == 1 && YAP1[0] == 1 && HNF1A[0] == 0 && HNF4A[0] == 1 && NFkB[0] == 0 && SNAI1[0] == 1 && SNAI2[0] == 1 && OCT4[0] == 1 || E2F[0] == 1 && Bcatenin[0] == 1 && YAP1[0] == 1 && HNF1A[0] == 0 && HNF4A[0] == 1 && NFkB[0] == 1 && SNAI1[0] == 0 && ZEB1[0] == 0 && TGFB[0] == 0 && OCT4[0] == 0 && NANOG[0] == 1 || E2F[0] == 1 && Bcatenin[0] == 1 && YAP1[0] == 1 && HNF1A[0] == 0 && HNF4A[0] == 1 && NFkB[0] == 1 && SNAI1[0] == 0 && ZEB1[0] == 0 && TGFB[0] == 0 && OCT4[0] == 1 || E2F[0] == 1 && Bcatenin[0] == 1 && YAP1[0] == 1 && HNF1A[0] == 0 && HNF4A[0] == 1 && NFkB[0] == 1 && SNAI1[0] == 0 && ZEB1[0] == 0 && TGFB[0] == 1 || E2F[0] == 1 && Bcatenin[0] == 1 && YAP1[0] == 1 && HNF1A[0] == 0 && HNF4A[0] == 1 && NFkB[0] == 1 && SNAI1[0] == 0 && ZEB1[0] == 1 || E2F[0] == 1 && Bcatenin[0] == 1 && YAP1[0] == 1 && HNF1A[0] == 0 && HNF4A[0] == 1 && NFkB[0] == 1 && SNAI1[0] == 1 || E2F[0] == 1 && Bcatenin[0] == 1 && YAP1[0] == 1 && HNF1A[0] == 1 && HNF4A[0] == 0 && NFkB[0] == 0 && SNAI1[0] == 0 && ZEB1[0] == 0 && OCT4[0] == 0 && NANOG[0] == 0 && p53[0] == 0 || E2F[0] == 1 && Bcatenin[0] == 1 && YAP1[0] == 1 && HNF1A[0] == 1 && HNF4A[0] == 0 && NFkB[0] == 0 && SNAI1[0] == 0 && ZEB1[0] == 0 && OCT4[0] == 0 && NANOG[0] == 1 || E2F[0] == 1 && Bcatenin[0] == 1 && YAP1[0] == 1 && HNF1A[0] == 1 && HNF4A[0] == 0 && NFkB[0] == 0 && SNAI1[0] == 0 && ZEB1[0] == 0 && OCT4[0] == 1 || E2F[0] == 1 && Bcatenin[0] == 1 && YAP1[0] == 1 && HNF1A[0] == 1 && HNF4A[0] == 0 && NFkB[0] == 0 && SNAI1[0] == 0 && ZEB1[0] == 1 || E2F[0] == 1 && Bcatenin[0] == 1 && YAP1[0] == 1 && HNF1A[0] == 1 && HNF4A[0] == 0 && NFkB[0] == 0 && SNAI1[0] == 1 && SNAI2[0] == 0 || E2F[0] == 1 && Bcatenin[0] == 1 && YAP1[0] == 1 && HNF1A[0] == 1 && HNF4A[0] == 0 && NFkB[0] == 0 && SNAI1[0] == 1 && SNAI2[0] == 1 && OCT4[0] == 0 && NANOG[0] == 0 && SOX2[0] == 0 || E2F[0] == 1 && Bcatenin[0] == 1 && YAP1[0] == 1 && HNF1A[0] == 1 && HNF4A[0] == 0 && NFkB[0] == 0 && SNAI1[0] == 1 && SNAI2[0] == 1 && OCT4[0] == 0 && NANOG[0] == 0 && SOX2[0] == 1 && p53[0] == 0 || E2F[0] == 1 && Bcatenin[0] == 1 && YAP1[0] == 1 && HNF1A[0] == 1 && HNF4A[0] == 0 && NFkB[0] == 0 && SNAI1[0] == 1 && SNAI2[0] == 1 && OCT4[0] == 0 && NANOG[0] == 1 || E2F[0] == 1 && Bcatenin[0] == 1 && YAP1[0] == 1 && HNF1A[0] == 1 && HNF4A[0] == 0 && NFkB[0] == 0 && SNAI1[0] == 1 && SNAI2[0] == 1 && OCT4[0] == 1 || E2F[0] == 1 && Bcatenin[0] == 1 && YAP1[0] == 1 && HNF1A[0] == 1 && HNF4A[0] == 0 && NFkB[0] == 1 && SNAI1[0] == 0 && ZEB1[0] == 0 && TGFB[0] == 0 && OCT4[0] == 0 && NANOG[0] == 0 && p53[0] == 0 || E2F[0] == 1 && Bcatenin[0] == 1 && YAP1[0] == 1 && HNF1A[0] == 1 && HNF4A[0] == 0 && NFkB[0] == 1 && SNAI1[0] == 0 && ZEB1[0] == 0 && TGFB[0] == 0 && OCT4[0] == 0 && NANOG[0] == 1 || E2F[0] == 1 && Bcatenin[0] == 1 && YAP1[0] == 1 && HNF1A[0] == 1 && HNF4A[0] == 0 && NFkB[0] == 1 && SNAI1[0] == 0 && ZEB1[0] == 0 && TGFB[0] == 0 && OCT4[0] == 1 || E2F[0] == 1 && Bcatenin[0] == 1 && YAP1[0] == 1 && HNF1A[0] == 1 && HNF4A[0] == 0 && NFkB[0] == 1 && SNAI1[0] == 0 && ZEB1[0] == 0 && TGFB[0] == 1 || E2F[0] == 1 && Bcatenin[0] == 1 && YAP1[0] == 1 && HNF1A[0] == 1 && HNF4A[0] == 0 && NFkB[0] == 1 && SNAI1[0] == 0 && ZEB1[0] == 1 || E2F[0] == 1 && Bcatenin[0] == 1 && YAP1[0] == 1 && HNF1A[0] == 1 && HNF4A[0] == 0 && NFkB[0] == 1 && SNAI1[0] == 1 && SNAI2[0] == 0 || E2F[0] == 1 && Bcatenin[0] == 1 && YAP1[0] == 1 && HNF1A[0] == 1 && HNF4A[0] == 0 && NFkB[0] == 1 && SNAI1[0] == 1 && SNAI2[0] == 1 && TGFB[0] == 0 && OCT4[0] == 0 && NANOG[0] == 0 && SOX2[0] == 0 || E2F[0] == 1 && Bcatenin[0] == 1 && YAP1[0] == 1 && HNF1A[0] == 1 && HNF4A[0] == 0 && NFkB[0] == 1 && SNAI1[0] == 1 && SNAI2[0] == 1 && TGFB[0] == 0 && OCT4[0] == 0 && NANOG[0] == 0 && SOX2[0] == 1 && p53[0] == 0 || E2F[0] == 1 && Bcatenin[0] == 1 && YAP1[0] == 1 && HNF1A[0] == 1 && HNF4A[0] == 0 && NFkB[0] == 1 && SNAI1[0] == 1 && SNAI2[0] == 1 && TGFB[0] == 0 && OCT4[0] == 0 && NANOG[0] == 1 || E2F[0] == 1 && Bcatenin[0] == 1 && YAP1[0] == 1 && HNF1A[0] == 1 && HNF4A[0] == 0 && NFkB[0] == 1 && SNAI1[0] == 1 && SNAI2[0] == 1 && TGFB[0] == 0 && OCT4[0] == 1 || E2F[0] == 1 && Bcatenin[0] == 1 && YAP1[0] == 1 && HNF1A[0] == 1 && HNF4A[0] == 0 && NFkB[0] == 1 && SNAI1[0] == 1 && SNAI2[0] == 1 && TGFB[0] == 1 || E2F[0] == 1 && Bcatenin[0] == 1 && YAP1[0] == 1 && HNF1A[0] == 1 && HNF4A[0] == 1 && NFkB[0] == 1 && TGFB[0] == 1) { SNAI1[0] = 1; }

else { SNAI1[0] = 0; }

//Logic rule for SNAI2

if (BMI1[0] == 0 && Bcatenin[0] == 0 && YAP1[0] == 0 && HNF1A[0] == 0 && HNF4A[0] == 0 && NFkB[0] == 0 && SNAI1[0] == 0 && ZEB1[0] == 0 && SNAI2[0] == 0 && SOX9[0] == 1 && OCT4[0] == 0 && SOX2[0] == 0 || BMI1[0] == 0 && Bcatenin[0] == 0 && YAP1[0] == 0 && HNF1A[0] == 0 && HNF4A[0] == 0 && NFkB[0] == 0 && SNAI1[0] == 0 && ZEB1[0] == 0 && SNAI2[0] == 1 && OCT4[0] == 0 || BMI1[0] == 0 && Bcatenin[0] == 0 && YAP1[0] == 0 && HNF1A[0] == 0 && HNF4A[0] == 0 && NFkB[0] == 0 && SNAI1[0] == 0 && ZEB1[0] == 1 && SNAI2[0] == 0 && SOX9[0] == 1 && OCT4[0] == 0 || BMI1[0] == 0 && Bcatenin[0] == 0 && YAP1[0] == 0 && HNF1A[0] == 0 && HNF4A[0] == 0 && NFkB[0] == 0 && SNAI1[0] == 0 && ZEB1[0] == 1 && SNAI2[0] == 0 && SOX9[0] == 1 && OCT4[0] == 1 && p53[0] == 0 || BMI1[0] == 0 && Bcatenin[0] == 0 && YAP1[0] == 0 && HNF1A[0] == 0 && HNF4A[0] == 0 && NFkB[0] == 0 && SNAI1[0] == 0 && ZEB1[0] == 1 && SNAI2[0] == 1 && OCT4[0] == 0 || BMI1[0] == 0 && Bcatenin[0] == 0 && YAP1[0] == 0 && HNF1A[0] == 0 && HNF4A[0] == 0 && NFkB[0] == 0 && SNAI1[0] == 0 && ZEB1[0] == 1 && SNAI2[0] == 1 && OCT4[0] == 1 && p53[0] == 0 || BMI1[0] == 0 && Bcatenin[0] == 0 && YAP1[0] == 0 && HNF1A[0] == 0 && HNF4A[0] == 0 && NFkB[0] == 0 && SNAI1[0] == 1 && ZEB1[0] == 0 && SNAI2[0] == 0 && OCT4[0] == 0 && SOX2[0] == 0 || BMI1[0] == 0 && Bcatenin[0] == 0 && YAP1[0] == 0 && HNF1A[0] == 0 && HNF4A[0] == 0 && NFkB[0] == 0 && SNAI1[0] == 1 && ZEB1[0] == 0 && SNAI2[0] == 0 && OCT4[0] == 1 && SOX2[0] == 0 && p53[0] == 0 || BMI1[0] == 0 && Bcatenin[0] == 0 && YAP1[0] == 0 && HNF1A[0] == 0 && HNF4A[0] == 0 && NFkB[0] == 0 && SNAI1[0] == 1 && ZEB1[0] == 0 && SNAI2[0] == 1 && OCT4[0] == 0 || BMI1[0] == 0 && Bcatenin[0] == 0 && YAP1[0] == 0 && HNF1A[0] == 0 && HNF4A[0] == 0 && NFkB[0] == 0 && SNAI1[0] == 1 && ZEB1[0] == 0 && SNAI2[0] == 1 && OCT4[0] == 1 && p53[0] == 0 || BMI1[0] == 0 && Bcatenin[0] == 0 && YAP1[0] == 0 && HNF1A[0] == 0 && HNF4A[0] == 0 && NFkB[0] == 0 && SNAI1[0] == 1 && ZEB1[0] == 1 || BMI1[0] == 0 && Bcatenin[0] == 0 && YAP1[0] == 0 && HNF1A[0] == 0 && HNF4A[0] == 0 && NFkB[0] == 1 && SNAI1[0] == 0 && ZEB1[0] == 0 && SNAI2[0] == 0 && OCT4[0] == 0 && SOX2[0] == 0 || BMI1[0] == 0 && Bcatenin[0] == 0 && YAP1[0] == 0 && HNF1A[0] == 0 && HNF4A[0] == 0 && NFkB[0] == 1 && SNAI1[0] == 0 && ZEB1[0] == 0 && SNAI2[0] == 1 && OCT4[0] == 0 || BMI1[0] == 0 && Bcatenin[0] == 0 && YAP1[0] == 0 && HNF1A[0] == 0 && HNF4A[0] == 0 && NFkB[0] == 1 && SNAI1[0] == 0 && ZEB1[0] == 1 && OCT4[0] == 0 || BMI1[0] == 0 && Bcatenin[0] == 0 && YAP1[0] == 0 && HNF1A[0] == 0 && HNF4A[0] == 0 && NFkB[0] == 1 && SNAI1[0] == 0 && ZEB1[0] == 1 && OCT4[0] == 1 && p53[0] == 0 || BMI1[0] == 0 && Bcatenin[0] == 0 && YAP1[0] == 0 && HNF1A[0] == 0 && HNF4A[0] == 0 && NFkB[0] == 1 && SNAI1[0] == 1 && ZEB1[0] == 0 && SNAI2[0] == 0 && OCT4[0] == 0 && SOX2[0] == 0 || BMI1[0] == 0 && Bcatenin[0] == 0 && YAP1[0] == 0 && HNF1A[0] == 0 && HNF4A[0] == 0 && NFkB[0] == 1 && SNAI1[0] == 1 && ZEB1[0] == 0 && SNAI2[0] == 0 && OCT4[0] == 1 && SOX2[0] == 0 && p53[0] == 0 || BMI1[0] == 0 && Bcatenin[0] == 0 && YAP1[0] == 0 && HNF1A[0] == 0 && HNF4A[0] == 0 && NFkB[0] == 1 && SNAI1[0] == 1 && ZEB1[0] == 0 && SNAI2[0] == 1 && OCT4[0] == 0 || BMI1[0] == 0 && Bcatenin[0] == 0 && YAP1[0] == 0 && HNF1A[0] == 0 && HNF4A[0] == 0 && NFkB[0] == 1 && SNAI1[0] == 1 && ZEB1[0] == 0 && SNAI2[0] == 1 && OCT4[0] == 1 && p53[0] == 0 || BMI1[0] == 0 && Bcatenin[0] == 0 && YAP1[0] == 0 && HNF1A[0] == 0 && HNF4A[0] == 0 && NFkB[0] == 1 && SNAI1[0] == 1 && ZEB1[0] == 1 || BMI1[0] == 0 && Bcatenin[0] == 0 && YAP1[0] == 0 && HNF1A[0] == 1 && HNF4A[0] == 0 && FOXA2[0] == 0 && NFkB[0] == 0 && SNAI1[0] == 0 && ZEB1[0] == 0 && SNAI2[0] == 0 && SOX9[0] == 1 && OCT4[0] == 0 && SOX2[0] == 0 || BMI1[0] == 0 && Bcatenin[0] == 0 && YAP1[0] == 0 && HNF1A[0] == 1 && HNF4A[0] == 0 && FOXA2[0] == 0 && NFkB[0] == 0 && SNAI1[0] == 0 && ZEB1[0] == 0 && SNAI2[0] == 1 && OCT4[0] == 0 || BMI1[0] == 0 && Bcatenin[0] == 0 && YAP1[0] == 0 && HNF1A[0] == 1 && HNF4A[0] == 0 && FOXA2[0] == 0 && NFkB[0] == 0 && SNAI1[0] == 0 && ZEB1[0] == 1 && SNAI2[0] == 0 && SOX9[0] == 1 && OCT4[0] == 0 || BMI1[0] == 0 && Bcatenin[0] == 0 && YAP1[0] == 0 && HNF1A[0] == 1 && HNF4A[0] == 0 && FOXA2[0] == 0 && NFkB[0] == 0 && SNAI1[0] == 0 && ZEB1[0] == 1 && SNAI2[0] == 0 && SOX9[0] == 1 && OCT4[0] == 1 && p53[0] == 0 || BMI1[0] == 0 && Bcatenin[0] == 0 && YAP1[0] == 0 && HNF1A[0] == 1 && HNF4A[0] == 0 && FOXA2[0] == 0 && NFkB[0] == 0 && SNAI1[0] == 0 && ZEB1[0] == 1 && SNAI2[0] == 1 && OCT4[0] == 0 || BMI1[0] == 0 && Bcatenin[0] == 0 && YAP1[0] == 0 && HNF1A[0] == 1 && HNF4A[0] == 0 && FOXA2[0] == 0 && NFkB[0] == 0 && SNAI1[0] == 0 && ZEB1[0] == 1 && SNAI2[0] == 1 && OCT4[0] == 1 && p53[0] == 0 || BMI1[0] == 0 && Bcatenin[0] == 0 && YAP1[0] == 0 && HNF1A[0] == 1 && HNF4A[0] == 0 && FOXA2[0] == 0 && NFkB[0] == 0 && SNAI1[0] == 1 && ZEB1[0] == 0 && SNAI2[0] == 0 && OCT4[0] == 0 && SOX2[0] == 0 || BMI1[0] == 0 && Bcatenin[0] == 0 && YAP1[0] == 0 && HNF1A[0] == 1 && HNF4A[0] == 0 && FOXA2[0] == 0 && NFkB[0] == 0 && SNAI1[0] == 1 && ZEB1[0] == 0 && SNAI2[0] == 0 && OCT4[0] == 1 && SOX2[0] == 0 && p53[0] == 0 || BMI1[0] == 0 && Bcatenin[0] == 0 && YAP1[0] == 0 && HNF1A[0] == 1 && HNF4A[0] == 0 && FOXA2[0] == 0 && NFkB[0] == 0 && SNAI1[0] == 1 && ZEB1[0] == 0 && SNAI2[0] == 1 && OCT4[0] == 0 || BMI1[0] == 0 && Bcatenin[0] == 0 && YAP1[0] == 0 && HNF1A[0] == 1 && HNF4A[0] == 0 && FOXA2[0] == 0 && NFkB[0] == 0 && SNAI1[0] == 1 && ZEB1[0] == 0 && SNAI2[0] == 1 && OCT4[0] == 1 && p53[0] == 0 || BMI1[0] == 0 && Bcatenin[0] == 0 && YAP1[0] == 0 && HNF1A[0] == 1 && HNF4A[0] == 0 && FOXA2[0] == 0 && NFkB[0] == 0 && SNAI1[0] == 1 && ZEB1[0] == 1 || BMI1[0] == 0 && Bcatenin[0] == 0 && YAP1[0] == 0 && HNF1A[0] == 1 && HNF4A[0] == 0 && FOXA2[0] == 0 && NFkB[0] == 1 && SNAI1[0] == 0 && ZEB1[0] == 0 && SNAI2[0] == 0 && OCT4[0] == 0 && SOX2[0] == 0 || BMI1[0] == 0 && Bcatenin[0] == 0 && YAP1[0] == 0 && HNF1A[0] == 1 && HNF4A[0] == 0 && FOXA2[0] == 0 && NFkB[0] == 1 && SNAI1[0] == 0 && ZEB1[0] == 0 && SNAI2[0] == 1 && OCT4[0] == 0 || BMI1[0] == 0 && Bcatenin[0] == 0 && YAP1[0] == 0 && HNF1A[0] == 1 && HNF4A[0] == 0 && FOXA2[0] == 0 && NFkB[0] == 1 && SNAI1[0] == 0 && ZEB1[0] == 1 && OCT4[0] == 0 || BMI1[0] == 0 && Bcatenin[0] == 0 && YAP1[0] == 0 && HNF1A[0] == 1 && HNF4A[0] == 0 && FOXA2[0] == 0 && NFkB[0] == 1 && SNAI1[0] == 0 && ZEB1[0] == 1 && OCT4[0] == 1 && p53[0] == 0 || BMI1[0] == 0 && Bcatenin[0] == 0 && YAP1[0] == 0 && HNF1A[0] == 1 && HNF4A[0] == 0 && FOXA2[0] == 0 && NFkB[0] == 1 && SNAI1[0] == 1 && ZEB1[0] == 0 && SNAI2[0] == 0 && OCT4[0] == 0 && SOX2[0] == 0 || BMI1[0] == 0 && Bcatenin[0] == 0 && YAP1[0] == 0 && HNF1A[0] == 1 && HNF4A[0] == 0 && FOXA2[0] == 0 && NFkB[0] == 1 && SNAI1[0] == 1 && ZEB1[0] == 0 && SNAI2[0] == 0 && OCT4[0] == 1 && SOX2[0] == 0 && p53[0] == 0 || BMI1[0] == 0 && Bcatenin[0] == 0 && YAP1[0] == 0 && HNF1A[0] == 1 && HNF4A[0] == 0 && FOXA2[0] == 0 && NFkB[0] == 1 && SNAI1[0] == 1 && ZEB1[0] == 0 && SNAI2[0] == 1 && OCT4[0] == 0 || BMI1[0] == 0 && Bcatenin[0] == 0 && YAP1[0] == 0 && HNF1A[0] == 1 && HNF4A[0] == 0 && FOXA2[0] == 0 && NFkB[0] == 1 && SNAI1[0] == 1 && ZEB1[0] == 0 && SNAI2[0] == 1 && OCT4[0] == 1 && p53[0] == 0 || BMI1[0] == 0 && Bcatenin[0] == 0 && YAP1[0] == 0 && HNF1A[0] == 1 && HNF4A[0] == 0 && FOXA2[0] == 0 && NFkB[0] == 1 && SNAI1[0] == 1 && ZEB1[0] == 1 || BMI1[0] == 0 && Bcatenin[0] == 0 && YAP1[0] == 1 && HNF1A[0] == 0 && HNF4A[0] == 0 && NFkB[0] == 0 && SNAI1[0] == 0 && ZEB1[0] == 0 && SNAI2[0] == 0 && SOX9[0] == 1 && OCT4[0] == 0 && SOX2[0] == 0 || BMI1[0] == 0 && Bcatenin[0] == 0 && YAP1[0] == 1 && HNF1A[0] == 0 && HNF4A[0] == 0 && NFkB[0] == 0 && SNAI1[0] == 0 && ZEB1[0] == 0 && SNAI2[0] == 1 && OCT4[0] == 0 || BMI1[0] == 0 && Bcatenin[0] == 0 && YAP1[0] == 1 && HNF1A[0] == 0 && HNF4A[0] == 0 && NFkB[0] == 0 && SNAI1[0] == 0 && ZEB1[0] == 1 && SNAI2[0] == 0 && SOX9[0] == 1 && OCT4[0] == 0 || BMI1[0] == 0 && Bcatenin[0] == 0 && YAP1[0] == 1 && HNF1A[0] == 0 && HNF4A[0] == 0 && NFkB[0] == 0 && SNAI1[0] == 0 && ZEB1[0] == 1 && SNAI2[0] == 0 && SOX9[0] == 1 && OCT4[0] == 1 && p53[0] == 0 || BMI1[0] == 0 && Bcatenin[0] == 0 && YAP1[0] == 1 && HNF1A[0] == 0 && HNF4A[0] == 0 && NFkB[0] == 0 && SNAI1[0] == 0 && ZEB1[0] == 1 && SNAI2[0] == 1 && OCT4[0] == 0 || BMI1[0] == 0 && Bcatenin[0] == 0 && YAP1[0] == 1 && HNF1A[0] == 0 && HNF4A[0] == 0 && NFkB[0] == 0 && SNAI1[0] == 0 && ZEB1[0] == 1 && SNAI2[0] == 1 && OCT4[0] == 1 && p53[0] == 0 || BMI1[0] == 0 && Bcatenin[0] == 0 && YAP1[0] == 1 && HNF1A[0] == 0 && HNF4A[0] == 0 && NFkB[0] == 0 && SNAI1[0] == 1 && ZEB1[0] == 0 && SNAI2[0] == 0 && OCT4[0] == 0 && SOX2[0] == 0 || BMI1[0] == 0 && Bcatenin[0] == 0 && YAP1[0] == 1 && HNF1A[0] == 0 && HNF4A[0] == 0 && NFkB[0] == 0 && SNAI1[0] == 1 && ZEB1[0] == 0 && SNAI2[0] == 0 && OCT4[0] == 1 && SOX2[0] == 0 && p53[0] == 0 || BMI1[0] == 0 && Bcatenin[0] == 0 && YAP1[0] == 1 && HNF1A[0] == 0 && HNF4A[0] == 0 && NFkB[0] == 0 && SNAI1[0] == 1 && ZEB1[0] == 0 && SNAI2[0] == 1 && OCT4[0] == 0 || BMI1[0] == 0 && Bcatenin[0] == 0 && YAP1[0] == 1 && HNF1A[0] == 0 && HNF4A[0] == 0 && NFkB[0] == 0 && SNAI1[0] == 1 && ZEB1[0] == 0 && SNAI2[0] == 1 && OCT4[0] == 1 && p53[0] == 0 || BMI1[0] == 0 && Bcatenin[0] == 0 && YAP1[0] == 1 && HNF1A[0] == 0 && HNF4A[0] == 0 && NFkB[0] == 0 && SNAI1[0] == 1 && ZEB1[0] == 1 || BMI1[0] == 0 && Bcatenin[0] == 0 && YAP1[0] == 1 && HNF1A[0] == 0 && HNF4A[0] == 0 && NFkB[0] == 1 && SNAI1[0] == 0 && ZEB1[0] == 0 && SNAI2[0] == 0 && OCT4[0] == 0 && SOX2[0] == 0 || BMI1[0] == 0 && Bcatenin[0] == 0 && YAP1[0] == 1 && HNF1A[0] == 0 && HNF4A[0] == 0 && NFkB[0] == 1 && SNAI1[0] == 0 && ZEB1[0] == 0 && SNAI2[0] == 1 && OCT4[0] == 0 || BMI1[0] == 0 && Bcatenin[0] == 0 && YAP1[0] == 1 && HNF1A[0] == 0 && HNF4A[0] == 0 && NFkB[0] == 1 && SNAI1[0] == 0 && ZEB1[0] == 1 && OCT4[0] == 0 || BMI1[0] == 0 && Bcatenin[0] == 0 && YAP1[0] == 1 && HNF1A[0] == 0 && HNF4A[0] == 0 && NFkB[0] == 1 && SNAI1[0] == 0 && ZEB1[0] == 1 && OCT4[0] == 1 && p53[0] == 0 || BMI1[0] == 0 && Bcatenin[0] == 0 && YAP1[0] == 1 && HNF1A[0] == 0 && HNF4A[0] == 0 && NFkB[0] == 1 && SNAI1[0] == 1 && ZEB1[0] == 0 && SNAI2[0] == 0 && OCT4[0] == 0 && SOX2[0] == 0 || BMI1[0] == 0 && Bcatenin[0] == 0 && YAP1[0] == 1 && HNF1A[0] == 0 && HNF4A[0] == 0 && NFkB[0] == 1 && SNAI1[0] == 1 && ZEB1[0] == 0 && SNAI2[0] == 0 && OCT4[0] == 1 && SOX2[0] == 0 && p53[0] == 0 || BMI1[0] == 0 && Bcatenin[0] == 0 && YAP1[0] == 1 && HNF1A[0] == 0 && HNF4A[0] == 0 && NFkB[0] == 1 && SNAI1[0] == 1 && ZEB1[0] == 0 && SNAI2[0] == 1 && OCT4[0] == 0 || BMI1[0] == 0 && Bcatenin[0] == 0 && YAP1[0] == 1 && HNF1A[0] == 0 && HNF4A[0] == 0 && NFkB[0] == 1 && SNAI1[0] == 1 && ZEB1[0] == 0 && SNAI2[0] == 1 && OCT4[0] == 1 && p53[0] == 0 || BMI1[0] == 0 && Bcatenin[0] == 0 && YAP1[0] == 1 && HNF1A[0] == 0 && HNF4A[0] == 0 && NFkB[0] == 1 && SNAI1[0] == 1 && ZEB1[0] == 1 || BMI1[0] == 0 && Bcatenin[0] == 0 && YAP1[0] == 1 && HNF1A[0] == 0 && HNF4A[0] == 1 && FOXA2[0] == 0 && NFkB[0] == 0 && SNAI1[0] == 0 && ZEB1[0] == 1 && SNAI2[0] == 1 && p53[0] == 0 || BMI1[0] == 0 && Bcatenin[0] == 0 && YAP1[0] == 1 && HNF1A[0] == 0 && HNF4A[0] == 1 && FOXA2[0] == 0 && NFkB[0] == 0 && SNAI1[0] == 1 && ZEB1[0] == 0 && SNAI2[0] == 1 && p53[0] == 0 || BMI1[0] == 0 && Bcatenin[0] == 0 && YAP1[0] == 1 && HNF1A[0] == 0 && HNF4A[0] == 1 && FOXA2[0] == 0 && NFkB[0] == 0 && SNAI1[0] == 1 && ZEB1[0] == 1 && SNAI2[0] == 0 && NANOG[0] == 1 || BMI1[0] == 0 && Bcatenin[0] == 0 && YAP1[0] == 1 && HNF1A[0] == 0 && HNF4A[0] == 1 && FOXA2[0] == 0 && NFkB[0] == 0 && SNAI1[0] == 1 && ZEB1[0] == 1 && SNAI2[0] == 1 || BMI1[0] == 0 && Bcatenin[0] == 0 && YAP1[0] == 1 && HNF1A[0] == 0 && HNF4A[0] == 1 && FOXA2[0] == 0 && NFkB[0] == 1 && SNAI1[0] == 0 && ZEB1[0] == 1 && p53[0] == 0 || BMI1[0] == 0 && Bcatenin[0] == 0 && YAP1[0] == 1 && HNF1A[0] == 0 && HNF4A[0] == 1 && FOXA2[0] == 0 && NFkB[0] == 1 && SNAI1[0] == 1 && ZEB1[0] == 0 && SNAI2[0] == 1 && p53[0] == 0 || BMI1[0] == 0 && Bcatenin[0] == 0 && YAP1[0] == 1 && HNF1A[0] == 0 && HNF4A[0] == 1 && FOXA2[0] == 0 && NFkB[0] == 1 && SNAI1[0] == 1 && ZEB1[0] == 1 || BMI1[0] == 0 && Bcatenin[0] == 0 && YAP1[0] == 1 && HNF1A[0] == 0 && HNF4A[0] == 1 && FOXA2[0] == 1 && NFkB[0] == 0 && SNAI1[0] == 0 && ZEB1[0] == 1 && SNAI2[0] == 1 && NANOG[0] == 1 && p53[0] == 0 || BMI1[0] == 0 && Bcatenin[0] == 0 && YAP1[0] == 1 && HNF1A[0] == 0 && HNF4A[0] == 1 && FOXA2[0] == 1 && NFkB[0] == 0 && SNAI1[0] == 1 && ZEB1[0] == 0 && SNAI2[0] == 1 && NANOG[0] == 1 && p53[0] == 0 || BMI1[0] == 0 && Bcatenin[0] == 0 && YAP1[0] == 1 && HNF1A[0] == 0 && HNF4A[0] == 1 && FOXA2[0] == 1 && NFkB[0] == 0 && SNAI1[0] == 1 && ZEB1[0] == 1 && NANOG[0] == 1 || BMI1[0] == 0 && Bcatenin[0] == 0 && YAP1[0] == 1 && HNF1A[0] == 0 && HNF4A[0] == 1 && FOXA2[0] == 1 && NFkB[0] == 1 && SNAI1[0] == 0 && ZEB1[0] == 1 && NANOG[0] == 1 && p53[0] == 0 || BMI1[0] == 0 && Bcatenin[0] == 0 && YAP1[0] == 1 && HNF1A[0] == 0 && HNF4A[0] == 1 && FOXA2[0] == 1 && NFkB[0] == 1 && SNAI1[0] == 1 && ZEB1[0] == 0 && SNAI2[0] == 1 && NANOG[0] == 1 && p53[0] == 0 || BMI1[0] == 0 && Bcatenin[0] == 0 && YAP1[0] == 1 && HNF1A[0] == 0 && HNF4A[0] == 1 && FOXA2[0] == 1 && NFkB[0] == 1 && SNAI1[0] == 1 && ZEB1[0] == 1 && NANOG[0] == 1 || BMI1[0] == 0 && Bcatenin[0] == 0 && YAP1[0] == 1 && HNF1A[0] == 1 && HNF4A[0] == 0 && FOXA2[0] == 0 && NFkB[0] == 0 && SNAI1[0] == 0 && ZEB1[0] == 0 && SNAI2[0] == 0 && SOX9[0] == 1 && OCT4[0] == 0 && SOX2[0] == 0 || BMI1[0] == 0 && Bcatenin[0] == 0 && YAP1[0] == 1 && HNF1A[0] == 1 && HNF4A[0] == 0 && FOXA2[0] == 0 && NFkB[0] == 0 && SNAI1[0] == 0 && ZEB1[0] == 0 && SNAI2[0] == 1 && OCT4[0] == 0 || BMI1[0] == 0 && Bcatenin[0] == 0 && YAP1[0] == 1 && HNF1A[0] == 1 && HNF4A[0] == 0 && FOXA2[0] == 0 && NFkB[0] == 0 && SNAI1[0] == 0 && ZEB1[0] == 1 && SNAI2[0] == 0 && SOX9[0] == 1 && OCT4[0] == 0 || BMI1[0] == 0 && Bcatenin[0] == 0 && YAP1[0] == 1 && HNF1A[0] == 1 && HNF4A[0] == 0 && FOXA2[0] == 0 && NFkB[0] == 0 && SNAI1[0] == 0 && ZEB1[0] == 1 && SNAI2[0] == 0 && SOX9[0] == 1 && OCT4[0] == 1 && p53[0] == 0 || BMI1[0] == 0 && Bcatenin[0] == 0 && YAP1[0] == 1 && HNF1A[0] == 1 && HNF4A[0] == 0 && FOXA2[0] == 0 && NFkB[0] == 0 && SNAI1[0] == 0 && ZEB1[0] == 1 && SNAI2[0] == 1 && OCT4[0] == 0 || BMI1[0] == 0 && Bcatenin[0] == 0 && YAP1[0] == 1 && HNF1A[0] == 1 && HNF4A[0] == 0 && FOXA2[0] == 0 && NFkB[0] == 0 && SNAI1[0] == 0 && ZEB1[0] == 1 && SNAI2[0] == 1 && OCT4[0] == 1 && p53[0] == 0 || BMI1[0] == 0 && Bcatenin[0] == 0 && YAP1[0] == 1 && HNF1A[0] == 1 && HNF4A[0] == 0 && FOXA2[0] == 0 && NFkB[0] == 0 && SNAI1[0] == 1 && ZEB1[0] == 0 && SNAI2[0] == 0 && OCT4[0] == 0 && SOX2[0] == 0 || BMI1[0] == 0 && Bcatenin[0] == 0 && YAP1[0] == 1 && HNF1A[0] == 1 && HNF4A[0] == 0 && FOXA2[0] == 0 && NFkB[0] == 0 && SNAI1[0] == 1 && ZEB1[0] == 0 && SNAI2[0] == 0 && OCT4[0] == 1 && SOX2[0] == 0 && p53[0] == 0 || BMI1[0] == 0 && Bcatenin[0] == 0 && YAP1[0] == 1 && HNF1A[0] == 1 && HNF4A[0] == 0 && FOXA2[0] == 0 && NFkB[0] == 0 && SNAI1[0] == 1 && ZEB1[0] == 0 && SNAI2[0] == 1 && OCT4[0] == 0 || BMI1[0] == 0 && Bcatenin[0] == 0 && YAP1[0] == 1 && HNF1A[0] == 1 && HNF4A[0] == 0 && FOXA2[0] == 0 && NFkB[0] == 0 && SNAI1[0] == 1 && ZEB1[0] == 0 && SNAI2[0] == 1 && OCT4[0] == 1 && p53[0] == 0 || BMI1[0] == 0 && Bcatenin[0] == 0 && YAP1[0] == 1 && HNF1A[0] == 1 && HNF4A[0] == 0 && FOXA2[0] == 0 && NFkB[0] == 0 && SNAI1[0] == 1 && ZEB1[0] == 1 || BMI1[0] == 0 && Bcatenin[0] == 0 && YAP1[0] == 1 && HNF1A[0] == 1 && HNF4A[0] == 0 && FOXA2[0] == 0 && NFkB[0] == 1 && SNAI1[0] == 0 && ZEB1[0] == 0 && SNAI2[0] == 0 && OCT4[0] == 0 && SOX2[0] == 0 || BMI1[0] == 0 && Bcatenin[0] == 0 && YAP1[0] == 1 && HNF1A[0] == 1 && HNF4A[0] == 0 && FOXA2[0] == 0 && NFkB[0] == 1 && SNAI1[0] == 0 && ZEB1[0] == 0 && SNAI2[0] == 1 && OCT4[0] == 0 || BMI1[0] == 0 && Bcatenin[0] == 0 && YAP1[0] == 1 && HNF1A[0] == 1 && HNF4A[0] == 0 && FOXA2[0] == 0 && NFkB[0] == 1 && SNAI1[0] == 0 && ZEB1[0] == 1 && OCT4[0] == 0 || BMI1[0] == 0 && Bcatenin[0] == 0 && YAP1[0] == 1 && HNF1A[0] == 1 && HNF4A[0] == 0 && FOXA2[0] == 0 && NFkB[0] == 1 && SNAI1[0] == 0 && ZEB1[0] == 1 && OCT4[0] == 1 && p53[0] == 0 || BMI1[0] == 0 && Bcatenin[0] == 0 && YAP1[0] == 1 && HNF1A[0] == 1 && HNF4A[0] == 0 && FOXA2[0] == 0 && NFkB[0] == 1 && SNAI1[0] == 1 && ZEB1[0] == 0 && SNAI2[0] == 0 && OCT4[0] == 0 && SOX2[0] == 0 || BMI1[0] == 0 && Bcatenin[0] == 0 && YAP1[0] == 1 && HNF1A[0] == 1 && HNF4A[0] == 0 && FOXA2[0] == 0 && NFkB[0] == 1 && SNAI1[0] == 1 && ZEB1[0] == 0 && SNAI2[0] == 0 && OCT4[0] == 1 && SOX2[0] == 0 && p53[0] == 0 || BMI1[0] == 0 && Bcatenin[0] == 0 && YAP1[0] == 1 && HNF1A[0] == 1 && HNF4A[0] == 0 && FOXA2[0] == 0 && NFkB[0] == 1 && SNAI1[0] == 1 && ZEB1[0] == 0 && SNAI2[0] == 1 && OCT4[0] == 0 || BMI1[0] == 0 && Bcatenin[0] == 0 && YAP1[0] == 1 && HNF1A[0] == 1 && HNF4A[0] == 0 && FOXA2[0] == 0 && NFkB[0] == 1 && SNAI1[0] == 1 && ZEB1[0] == 0 && SNAI2[0] == 1 && OCT4[0] == 1 && p53[0] == 0 || BMI1[0] == 0 && Bcatenin[0] == 0 && YAP1[0] == 1 && HNF1A[0] == 1 && HNF4A[0] == 0 && FOXA2[0] == 0 && NFkB[0] == 1 && SNAI1[0] == 1 && ZEB1[0] == 1 || BMI1[0] == 0 && Bcatenin[0] == 0 && YAP1[0] == 1 && HNF1A[0] == 1 && HNF4A[0] == 0 && FOXA2[0] == 1 && NFkB[0] == 0 && SNAI1[0] == 0 && ZEB1[0] == 0 && SNAI2[0] == 1 && OCT4[0] == 0 && NANOG[0] == 1 || BMI1[0] == 0 && Bcatenin[0] == 0 && YAP1[0] == 1 && HNF1A[0] == 1 && HNF4A[0] == 0 && FOXA2[0] == 1 && NFkB[0] == 0 && SNAI1[0] == 0 && ZEB1[0] == 1 && SNAI2[0] == 1 && OCT4[0] == 0 && NANOG[0] == 1 || BMI1[0] == 0 && Bcatenin[0] == 0 && YAP1[0] == 1 && HNF1A[0] == 1 && HNF4A[0] == 0 && FOXA2[0] == 1 && NFkB[0] == 0 && SNAI1[0] == 0 && ZEB1[0] == 1 && SNAI2[0] == 1 && OCT4[0] == 1 && NANOG[0] == 1 && p53[0] == 0 || BMI1[0] == 0 && Bcatenin[0] == 0 && YAP1[0] == 1 && HNF1A[0] == 1 && HNF4A[0] == 0 && FOXA2[0] == 1 && NFkB[0] == 0 && SNAI1[0] == 1 && ZEB1[0] == 0 && SNAI2[0] == 0 && OCT4[0] == 0 && NANOG[0] == 1 && SOX2[0] == 0 || BMI1[0] == 0 && Bcatenin[0] == 0 && YAP1[0] == 1 && HNF1A[0] == 1 && HNF4A[0] == 0 && FOXA2[0] == 1 && NFkB[0] == 0 && SNAI1[0] == 1 && ZEB1[0] == 0 && SNAI2[0] == 0 && OCT4[0] == 1 && NANOG[0] == 1 && SOX2[0] == 0 && p53[0] == 0 || BMI1[0] == 0 && Bcatenin[0] == 0 && YAP1[0] == 1 && HNF1A[0] == 1 && HNF4A[0] == 0 && FOXA2[0] == 1 && NFkB[0] == 0 && SNAI1[0] == 1 && ZEB1[0] == 0 && SNAI2[0] == 1 && OCT4[0] == 0 && NANOG[0] == 1 || BMI1[0] == 0 && Bcatenin[0] == 0 && YAP1[0] == 1 && HNF1A[0] == 1 && HNF4A[0] == 0 && FOXA2[0] == 1 && NFkB[0] == 0 && SNAI1[0] == 1 && ZEB1[0] == 0 && SNAI2[0] == 1 && OCT4[0] == 1 && NANOG[0] == 1 && p53[0] == 0 || BMI1[0] == 0 && Bcatenin[0] == 0 && YAP1[0] == 1 && HNF1A[0] == 1 && HNF4A[0] == 0 && FOXA2[0] == 1 && NFkB[0] == 0 && SNAI1[0] == 1 && ZEB1[0] == 1 && NANOG[0] == 1 || BMI1[0] == 0 && Bcatenin[0] == 0 && YAP1[0] == 1 && HNF1A[0] == 1 && HNF4A[0] == 0 && FOXA2[0] == 1 && NFkB[0] == 1 && SNAI1[0] == 0 && ZEB1[0] == 0 && SNAI2[0] == 0 && OCT4[0] == 0 && NANOG[0] == 1 && SOX2[0] == 0 && p53[0] == 0 || BMI1[0] == 0 && Bcatenin[0] == 0 && YAP1[0] == 1 && HNF1A[0] == 1 && HNF4A[0] == 0 && FOXA2[0] == 1 && NFkB[0] == 1 && SNAI1[0] == 0 && ZEB1[0] == 0 && SNAI2[0] == 1 && OCT4[0] == 0 && NANOG[0] == 1 || BMI1[0] == 0 && Bcatenin[0] == 0 && YAP1[0] == 1 && HNF1A[0] == 1 && HNF4A[0] == 0 && FOXA2[0] == 1 && NFkB[0] == 1 && SNAI1[0] == 0 && ZEB1[0] == 1 && OCT4[0] == 0 && NANOG[0] == 1 || BMI1[0] == 0 && Bcatenin[0] == 0 && YAP1[0] == 1 && HNF1A[0] == 1 && HNF4A[0] == 0 && FOXA2[0] == 1 && NFkB[0] == 1 && SNAI1[0] == 0 && ZEB1[0] == 1 && OCT4[0] == 1 && NANOG[0] == 1 && p53[0] == 0 || BMI1[0] == 0 && Bcatenin[0] == 0 && YAP1[0] == 1 && HNF1A[0] == 1 && HNF4A[0] == 0 && FOXA2[0] == 1 && NFkB[0] == 1 && SNAI1[0] == 1 && ZEB1[0] == 0 && SNAI2[0] == 0 && OCT4[0] == 0 && NANOG[0] == 1 && SOX2[0] == 0 || BMI1[0] == 0 && Bcatenin[0] == 0 && YAP1[0] == 1 && HNF1A[0] == 1 && HNF4A[0] == 0 && FOXA2[0] == 1 && NFkB[0] == 1 && SNAI1[0] == 1 && ZEB1[0] == 0 && SNAI2[0] == 0 && OCT4[0] == 1 && NANOG[0] == 1 && SOX2[0] == 0 && p53[0] == 0 || BMI1[0] == 0 && Bcatenin[0] == 0 && YAP1[0] == 1 && HNF1A[0] == 1 && HNF4A[0] == 0 && FOXA2[0] == 1 && NFkB[0] == 1 && SNAI1[0] == 1 && ZEB1[0] == 0 && SNAI2[0] == 1 && OCT4[0] == 0 && NANOG[0] == 1 || BMI1[0] == 0 && Bcatenin[0] == 0 && YAP1[0] == 1 && HNF1A[0] == 1 && HNF4A[0] == 0 && FOXA2[0] == 1 && NFkB[0] == 1 && SNAI1[0] == 1 && ZEB1[0] == 0 && SNAI2[0] == 1 && OCT4[0] == 1 && NANOG[0] == 1 && p53[0] == 0 || BMI1[0] == 0 && Bcatenin[0] == 0 && YAP1[0] == 1 && HNF1A[0] == 1 && HNF4A[0] == 0 && FOXA2[0] == 1 && NFkB[0] == 1 && SNAI1[0] == 1 && ZEB1[0] == 1 && NANOG[0] == 1 || BMI1[0] == 0 && Bcatenin[0] == 1 && HNF1A[0] == 0 && HNF4A[0] == 0 && NFkB[0] == 0 && SNAI1[0] == 0 && ZEB1[0] == 0 && SNAI2[0] == 0 && SOX9[0] == 1 && OCT4[0] == 0 && SOX2[0] == 0 || BMI1[0] == 0 && Bcatenin[0] == 1 && HNF1A[0] == 0 && HNF4A[0] == 0 && NFkB[0] == 0 && SNAI1[0] == 0 && ZEB1[0] == 0 && SNAI2[0] == 1 && OCT4[0] == 0 || BMI1[0] == 0 && Bcatenin[0] == 1 && HNF1A[0] == 0 && HNF4A[0] == 0 && NFkB[0] == 0 && SNAI1[0] == 0 && ZEB1[0] == 1 && SNAI2[0] == 0 && SOX9[0] == 1 && OCT4[0] == 0 || BMI1[0] == 0 && Bcatenin[0] == 1 && HNF1A[0] == 0 && HNF4A[0] == 0 && NFkB[0] == 0 && SNAI1[0] == 0 && ZEB1[0] == 1 && SNAI2[0] == 0 && SOX9[0] == 1 && OCT4[0] == 1 && p53[0] == 0 || BMI1[0] == 0 && Bcatenin[0] == 1 && HNF1A[0] == 0 && HNF4A[0] == 0 && NFkB[0] == 0 && SNAI1[0] == 0 && ZEB1[0] == 1 && SNAI2[0] == 1 && OCT4[0] == 0 || BMI1[0] == 0 && Bcatenin[0] == 1 && HNF1A[0] == 0 && HNF4A[0] == 0 && NFkB[0] == 0 && SNAI1[0] == 0 && ZEB1[0] == 1 && SNAI2[0] == 1 && OCT4[0] == 1 && p53[0] == 0 || BMI1[0] == 0 && Bcatenin[0] == 1 && HNF1A[0] == 0 && HNF4A[0] == 0 && NFkB[0] == 0 && SNAI1[0] == 1 && ZEB1[0] == 0 && SNAI2[0] == 0 && OCT4[0] == 0 && SOX2[0] == 0 || BMI1[0] == 0 && Bcatenin[0] == 1 && HNF1A[0] == 0 && HNF4A[0] == 0 && NFkB[0] == 0 && SNAI1[0] == 1 && ZEB1[0] == 0 && SNAI2[0] == 0 && OCT4[0] == 1 && SOX2[0] == 0 && p53[0] == 0 || BMI1[0] == 0 && Bcatenin[0] == 1 && HNF1A[0] == 0 && HNF4A[0] == 0 && NFkB[0] == 0 && SNAI1[0] == 1 && ZEB1[0] == 0 && SNAI2[0] == 1 && OCT4[0] == 0 || BMI1[0] == 0 && Bcatenin[0] == 1 && HNF1A[0] == 0 && HNF4A[0] == 0 && NFkB[0] == 0 && SNAI1[0] == 1 && ZEB1[0] == 0 && SNAI2[0] == 1 && OCT4[0] == 1 && p53[0] == 0 || BMI1[0] == 0 && Bcatenin[0] == 1 && HNF1A[0] == 0 && HNF4A[0] == 0 && NFkB[0] == 0 && SNAI1[0] == 1 && ZEB1[0] == 1 || BMI1[0] == 0 && Bcatenin[0] == 1 && HNF1A[0] == 0 && HNF4A[0] == 0 && NFkB[0] == 1 && SNAI1[0] == 0 && ZEB1[0] == 0 && SNAI2[0] == 0 && OCT4[0] == 0 && SOX2[0] == 0 || BMI1[0] == 0 && Bcatenin[0] == 1 && HNF1A[0] == 0 && HNF4A[0] == 0 && NFkB[0] == 1 && SNAI1[0] == 0 && ZEB1[0] == 0 && SNAI2[0] == 1 && OCT4[0] == 0 || BMI1[0] == 0 && Bcatenin[0] == 1 && HNF1A[0] == 0 && HNF4A[0] == 0 && NFkB[0] == 1 && SNAI1[0] == 0 && ZEB1[0] == 1 && OCT4[0] == 0 || BMI1[0] == 0 && Bcatenin[0] == 1 && HNF1A[0] == 0 && HNF4A[0] == 0 && NFkB[0] == 1 && SNAI1[0] == 0 && ZEB1[0] == 1 && OCT4[0] == 1 && p53[0] == 0 || BMI1[0] == 0 && Bcatenin[0] == 1 && HNF1A[0] == 0 && HNF4A[0] == 0 && NFkB[0] == 1 && SNAI1[0] == 1 && ZEB1[0] == 0 && SNAI2[0] == 0 && OCT4[0] == 0 && SOX2[0] == 0 || BMI1[0] == 0 && Bcatenin[0] == 1 && HNF1A[0] == 0 && HNF4A[0] == 0 && NFkB[0] == 1 && SNAI1[0] == 1 && ZEB1[0] == 0 && SNAI2[0] == 0 && OCT4[0] == 1 && SOX2[0] == 0 && p53[0] == 0 || BMI1[0] == 0 && Bcatenin[0] == 1 && HNF1A[0] == 0 && HNF4A[0] == 0 && NFkB[0] == 1 && SNAI1[0] == 1 && ZEB1[0] == 0 && SNAI2[0] == 1 && OCT4[0] == 0 || BMI1[0] == 0 && Bcatenin[0] == 1 && HNF1A[0] == 0 && HNF4A[0] == 0 && NFkB[0] == 1 && SNAI1[0] == 1 && ZEB1[0] == 0 && SNAI2[0] == 1 && OCT4[0] == 1 && p53[0] == 0 || BMI1[0] == 0 && Bcatenin[0] == 1 && HNF1A[0] == 0 && HNF4A[0] == 0 && NFkB[0] == 1 && SNAI1[0] == 1 && ZEB1[0] == 1 || BMI1[0] == 0 && Bcatenin[0] == 1 && HNF1A[0] == 0 && HNF4A[0] == 1 && FOXA2[0] == 0 && NFkB[0] == 0 && SNAI1[0] == 0 && ZEB1[0] == 1 && SNAI2[0] == 1 && p53[0] == 0 || BMI1[0] == 0 && Bcatenin[0] == 1 && HNF1A[0] == 0 && HNF4A[0] == 1 && FOXA2[0] == 0 && NFkB[0] == 0 && SNAI1[0] == 1 && ZEB1[0] == 0 && SNAI2[0] == 1 && p53[0] == 0 || BMI1[0] == 0 && Bcatenin[0] == 1 && HNF1A[0] == 0 && HNF4A[0] == 1 && FOXA2[0] == 0 && NFkB[0] == 0 && SNAI1[0] == 1 && ZEB1[0] == 1 && SNAI2[0] == 0 && NANOG[0] == 1 || BMI1[0] == 0 && Bcatenin[0] == 1 && HNF1A[0] == 0 && HNF4A[0] == 1 && FOXA2[0] == 0 && NFkB[0] == 0 && SNAI1[0] == 1 && ZEB1[0] == 1 && SNAI2[0] == 1 || BMI1[0] == 0 && Bcatenin[0] == 1 && HNF1A[0] == 0 && HNF4A[0] == 1 && FOXA2[0] == 0 && NFkB[0] == 1 && SNAI1[0] == 0 && ZEB1[0] == 1 && p53[0] == 0 || BMI1[0] == 0 && Bcatenin[0] == 1 && HNF1A[0] == 0 && HNF4A[0] == 1 && FOXA2[0] == 0 && NFkB[0] == 1 && SNAI1[0] == 1 && ZEB1[0] == 0 && SNAI2[0] == 1 && p53[0] == 0 || BMI1[0] == 0 && Bcatenin[0] == 1 && HNF1A[0] == 0 && HNF4A[0] == 1 && FOXA2[0] == 0 && NFkB[0] == 1 && SNAI1[0] == 1 && ZEB1[0] == 1 || BMI1[0] == 0 && Bcatenin[0] == 1 && HNF1A[0] == 0 && HNF4A[0] == 1 && FOXA2[0] == 1 && NFkB[0] == 0 && SNAI1[0] == 0 && ZEB1[0] == 1 && SNAI2[0] == 1 && NANOG[0] == 1 && p53[0] == 0 || BMI1[0] == 0 && Bcatenin[0] == 1 && HNF1A[0] == 0 && HNF4A[0] == 1 && FOXA2[0] == 1 && NFkB[0] == 0 && SNAI1[0] == 1 && ZEB1[0] == 0 && SNAI2[0] == 1 && NANOG[0] == 1 && p53[0] == 0 || BMI1[0] == 0 && Bcatenin[0] == 1 && HNF1A[0] == 0 && HNF4A[0] == 1 && FOXA2[0] == 1 && NFkB[0] == 0 && SNAI1[0] == 1 && ZEB1[0] == 1 && NANOG[0] == 1 || BMI1[0] == 0 && Bcatenin[0] == 1 && HNF1A[0] == 0 && HNF4A[0] == 1 && FOXA2[0] == 1 && NFkB[0] == 1 && SNAI1[0] == 0 && ZEB1[0] == 1 && NANOG[0] == 1 && p53[0] == 0 || BMI1[0] == 0 && Bcatenin[0] == 1 && HNF1A[0] == 0 && HNF4A[0] == 1 && FOXA2[0] == 1 && NFkB[0] == 1 && SNAI1[0] == 1 && ZEB1[0] == 0 && SNAI2[0] == 1 && NANOG[0] == 1 && p53[0] == 0 || BMI1[0] == 0 && Bcatenin[0] == 1 && HNF1A[0] == 0 && HNF4A[0] == 1 && FOXA2[0] == 1 && NFkB[0] == 1 && SNAI1[0] == 1 && ZEB1[0] == 1 && NANOG[0] == 1 || BMI1[0] == 0 && Bcatenin[0] == 1 && HNF1A[0] == 1 && HNF4A[0] == 0 && FOXA2[0] == 0 && NFkB[0] == 0 && SNAI1[0] == 0 && ZEB1[0] == 0 && SNAI2[0] == 0 && SOX9[0] == 1 && OCT4[0] == 0 && SOX2[0] == 0 || BMI1[0] == 0 && Bcatenin[0] == 1 && HNF1A[0] == 1 && HNF4A[0] == 0 && FOXA2[0] == 0 && NFkB[0] == 0 && SNAI1[0] == 0 && ZEB1[0] == 0 && SNAI2[0] == 1 && OCT4[0] == 0 || BMI1[0] == 0 && Bcatenin[0] == 1 && HNF1A[0] == 1 && HNF4A[0] == 0 && FOXA2[0] == 0 && NFkB[0] == 0 && SNAI1[0] == 0 && ZEB1[0] == 1 && SNAI2[0] == 0 && SOX9[0] == 1 && OCT4[0] == 0 || BMI1[0] == 0 && Bcatenin[0] == 1 && HNF1A[0] == 1 && HNF4A[0] == 0 && FOXA2[0] == 0 && NFkB[0] == 0 && SNAI1[0] == 0 && ZEB1[0] == 1 && SNAI2[0] == 0 && SOX9[0] == 1 && OCT4[0] == 1 && p53[0] == 0 || BMI1[0] == 0 && Bcatenin[0] == 1 && HNF1A[0] == 1 && HNF4A[0] == 0 && FOXA2[0] == 0 && NFkB[0] == 0 && SNAI1[0] == 0 && ZEB1[0] == 1 && SNAI2[0] == 1 && OCT4[0] == 0 || BMI1[0] == 0 && Bcatenin[0] == 1 && HNF1A[0] == 1 && HNF4A[0] == 0 && FOXA2[0] == 0 && NFkB[0] == 0 && SNAI1[0] == 0 && ZEB1[0] == 1 && SNAI2[0] == 1 && OCT4[0] == 1 && p53[0] == 0 || BMI1[0] == 0 && Bcatenin[0] == 1 && HNF1A[0] == 1 && HNF4A[0] == 0 && FOXA2[0] == 0 && NFkB[0] == 0 && SNAI1[0] == 1 && ZEB1[0] == 0 && SNAI2[0] == 0 && OCT4[0] == 0 && SOX2[0] == 0 || BMI1[0] == 0 && Bcatenin[0] == 1 && HNF1A[0] == 1 && HNF4A[0] == 0 && FOXA2[0] == 0 && NFkB[0] == 0 && SNAI1[0] == 1 && ZEB1[0] == 0 && SNAI2[0] == 0 && OCT4[0] == 1 && SOX2[0] == 0 && p53[0] == 0 || BMI1[0] == 0 && Bcatenin[0] == 1 && HNF1A[0] == 1 && HNF4A[0] == 0 && FOXA2[0] == 0 && NFkB[0] == 0 && SNAI1[0] == 1 && ZEB1[0] == 0 && SNAI2[0] == 1 && OCT4[0] == 0 || BMI1[0] == 0 && Bcatenin[0] == 1 && HNF1A[0] == 1 && HNF4A[0] == 0 && FOXA2[0] == 0 && NFkB[0] == 0 && SNAI1[0] == 1 && ZEB1[0] == 0 && SNAI2[0] == 1 && OCT4[0] == 1 && p53[0] == 0 || BMI1[0] == 0 && Bcatenin[0] == 1 && HNF1A[0] == 1 && HNF4A[0] == 0 && FOXA2[0] == 0 && NFkB[0] == 0 && SNAI1[0] == 1 && ZEB1[0] == 1 || BMI1[0] == 0 && Bcatenin[0] == 1 && HNF1A[0] == 1 && HNF4A[0] == 0 && FOXA2[0] == 0 && NFkB[0] == 1 && SNAI1[0] == 0 && ZEB1[0] == 0 && SNAI2[0] == 0 && OCT4[0] == 0 && SOX2[0] == 0 || BMI1[0] == 0 && Bcatenin[0] == 1 && HNF1A[0] == 1 && HNF4A[0] == 0 && FOXA2[0] == 0 && NFkB[0] == 1 && SNAI1[0] == 0 && ZEB1[0] == 0 && SNAI2[0] == 1 && OCT4[0] == 0 || BMI1[0] == 0 && Bcatenin[0] == 1 && HNF1A[0] == 1 && HNF4A[0] == 0 && FOXA2[0] == 0 && NFkB[0] == 1 && SNAI1[0] == 0 && ZEB1[0] == 1 && OCT4[0] == 0 || BMI1[0] == 0 && Bcatenin[0] == 1 && HNF1A[0] == 1 && HNF4A[0] == 0 && FOXA2[0] == 0 && NFkB[0] == 1 && SNAI1[0] == 0 && ZEB1[0] == 1 && OCT4[0] == 1 && p53[0] == 0 || BMI1[0] == 0 && Bcatenin[0] == 1 && HNF1A[0] == 1 && HNF4A[0] == 0 && FOXA2[0] == 0 && NFkB[0] == 1 && SNAI1[0] == 1 && ZEB1[0] == 0 && SNAI2[0] == 0 && OCT4[0] == 0 && SOX2[0] == 0 || BMI1[0] == 0 && Bcatenin[0] == 1 && HNF1A[0] == 1 && HNF4A[0] == 0 && FOXA2[0] == 0 && NFkB[0] == 1 && SNAI1[0] == 1 && ZEB1[0] == 0 && SNAI2[0] == 0 && OCT4[0] == 1 && SOX2[0] == 0 && p53[0] == 0 || BMI1[0] == 0 && Bcatenin[0] == 1 && HNF1A[0] == 1 && HNF4A[0] == 0 && FOXA2[0] == 0 && NFkB[0] == 1 && SNAI1[0] == 1 && ZEB1[0] == 0 && SNAI2[0] == 1 && OCT4[0] == 0 || BMI1[0] == 0 && Bcatenin[0] == 1 && HNF1A[0] == 1 && HNF4A[0] == 0 && FOXA2[0] == 0 && NFkB[0] == 1 && SNAI1[0] == 1 && ZEB1[0] == 0 && SNAI2[0] == 1 && OCT4[0] == 1 && p53[0] == 0 || BMI1[0] == 0 && Bcatenin[0] == 1 && HNF1A[0] == 1 && HNF4A[0] == 0 && FOXA2[0] == 0 && NFkB[0] == 1 && SNAI1[0] == 1 && ZEB1[0] == 1 || BMI1[0] == 0 && Bcatenin[0] == 1 && HNF1A[0] == 1 && HNF4A[0] == 0 && FOXA2[0] == 1 && NFkB[0] == 0 && SNAI1[0] == 0 && ZEB1[0] == 0 && SNAI2[0] == 1 && OCT4[0] == 0 && NANOG[0] == 1 || BMI1[0] == 0 && Bcatenin[0] == 1 && HNF1A[0] == 1 && HNF4A[0] == 0 && FOXA2[0] == 1 && NFkB[0] == 0 && SNAI1[0] == 0 && ZEB1[0] == 1 && SNAI2[0] == 1 && OCT4[0] == 0 && NANOG[0] == 1 || BMI1[0] == 0 && Bcatenin[0] == 1 && HNF1A[0] == 1 && HNF4A[0] == 0 && FOXA2[0] == 1 && NFkB[0] == 0 && SNAI1[0] == 0 && ZEB1[0] == 1 && SNAI2[0] == 1 && OCT4[0] == 1 && NANOG[0] == 1 && p53[0] == 0 || BMI1[0] == 0 && Bcatenin[0] == 1 && HNF1A[0] == 1 && HNF4A[0] == 0 && FOXA2[0] == 1 && NFkB[0] == 0 && SNAI1[0] == 1 && ZEB1[0] == 0 && SNAI2[0] == 0 && OCT4[0] == 0 && NANOG[0] == 1 && SOX2[0] == 0 || BMI1[0] == 0 && Bcatenin[0] == 1 && HNF1A[0] == 1 && HNF4A[0] == 0 && FOXA2[0] == 1 && NFkB[0] == 0 && SNAI1[0] == 1 && ZEB1[0] == 0 && SNAI2[0] == 0 && OCT4[0] == 1 && NANOG[0] == 1 && SOX2[0] == 0 && p53[0] == 0 || BMI1[0] == 0 && Bcatenin[0] == 1 && HNF1A[0] == 1 && HNF4A[0] == 0 && FOXA2[0] == 1 && NFkB[0] == 0 && SNAI1[0] == 1 && ZEB1[0] == 0 && SNAI2[0] == 1 && OCT4[0] == 0 && NANOG[0] == 1 || BMI1[0] == 0 && Bcatenin[0] == 1 && HNF1A[0] == 1 && HNF4A[0] == 0 && FOXA2[0] == 1 && NFkB[0] == 0 && SNAI1[0] == 1 && ZEB1[0] == 0 && SNAI2[0] == 1 && OCT4[0] == 1 && NANOG[0] == 1 && p53[0] == 0 || BMI1[0] == 0 && Bcatenin[0] == 1 && HNF1A[0] == 1 && HNF4A[0] == 0 && FOXA2[0] == 1 && NFkB[0] == 0 && SNAI1[0] == 1 && ZEB1[0] == 1 && NANOG[0] == 1 || BMI1[0] == 0 && Bcatenin[0] == 1 && HNF1A[0] == 1 && HNF4A[0] == 0 && FOXA2[0] == 1 && NFkB[0] == 1 && SNAI1[0] == 0 && ZEB1[0] == 0 && SNAI2[0] == 0 && OCT4[0] == 0 && NANOG[0] == 1 && SOX2[0] == 0 && p53[0] == 0 || BMI1[0] == 0 && Bcatenin[0] == 1 && HNF1A[0] == 1 && HNF4A[0] == 0 && FOXA2[0] == 1 && NFkB[0] == 1 && SNAI1[0] == 0 && ZEB1[0] == 0 && SNAI2[0] == 1 && OCT4[0] == 0 && NANOG[0] == 1 || BMI1[0] == 0 && Bcatenin[0] == 1 && HNF1A[0] == 1 && HNF4A[0] == 0 && FOXA2[0] == 1 && NFkB[0] == 1 && SNAI1[0] == 0 && ZEB1[0] == 1 && OCT4[0] == 0 && NANOG[0] == 1 || BMI1[0] == 0 && Bcatenin[0] == 1 && HNF1A[0] == 1 && HNF4A[0] == 0 && FOXA2[0] == 1 && NFkB[0] == 1 && SNAI1[0] == 0 && ZEB1[0] == 1 && OCT4[0] == 1 && NANOG[0] == 1 && p53[0] == 0 || BMI1[0] == 0 && Bcatenin[0] == 1 && HNF1A[0] == 1 && HNF4A[0] == 0 && FOXA2[0] == 1 && NFkB[0] == 1 && SNAI1[0] == 1 && ZEB1[0] == 0 && SNAI2[0] == 0 && OCT4[0] == 0 && NANOG[0] == 1 && SOX2[0] == 0 || BMI1[0] == 0 && Bcatenin[0] == 1 && HNF1A[0] == 1 && HNF4A[0] == 0 && FOXA2[0] == 1 && NFkB[0] == 1 && SNAI1[0] == 1 && ZEB1[0] == 0 && SNAI2[0] == 0 && OCT4[0] == 1 && NANOG[0] == 1 && SOX2[0] == 0 && p53[0] == 0 || BMI1[0] == 0 && Bcatenin[0] == 1 && HNF1A[0] == 1 && HNF4A[0] == 0 && FOXA2[0] == 1 && NFkB[0] == 1 && SNAI1[0] == 1 && ZEB1[0] == 0 && SNAI2[0] == 1 && OCT4[0] == 0 && NANOG[0] == 1 || BMI1[0] == 0 && Bcatenin[0] == 1 && HNF1A[0] == 1 && HNF4A[0] == 0 && FOXA2[0] == 1 && NFkB[0] == 1 && SNAI1[0] == 1 && ZEB1[0] == 0 && SNAI2[0] == 1 && OCT4[0] == 1 && NANOG[0] == 1 && p53[0] == 0 || BMI1[0] == 0 && Bcatenin[0] == 1 && HNF1A[0] == 1 && HNF4A[0] == 0 && FOXA2[0] == 1 && NFkB[0] == 1 && SNAI1[0] == 1 && ZEB1[0] == 1 && NANOG[0] == 1 || BMI1[0] == 1 && Bcatenin[0] == 0 && YAP1[0] == 0 && HNF1A[0] == 0 && HNF4A[0] == 0 && NFkB[0] == 0 && SNAI1[0] == 0 && ZEB1[0] == 0 && SNAI2[0] == 0 && SOX9[0] == 1 && SOX2[0] == 0 || BMI1[0] == 1 && Bcatenin[0] == 0 && YAP1[0] == 0 && HNF1A[0] == 0 && HNF4A[0] == 0 && NFkB[0] == 0 && SNAI1[0] == 0 && ZEB1[0] == 0 && SNAI2[0] == 1 || BMI1[0] == 1 && Bcatenin[0] == 0 && YAP1[0] == 0 && HNF1A[0] == 0 && HNF4A[0] == 0 && NFkB[0] == 0 && SNAI1[0] == 0 && ZEB1[0] == 1 && SNAI2[0] == 0 && SOX9[0] == 1 || BMI1[0] == 1 && Bcatenin[0] == 0 && YAP1[0] == 0 && HNF1A[0] == 0 && HNF4A[0] == 0 && NFkB[0] == 0 && SNAI1[0] == 0 && ZEB1[0] == 1 && SNAI2[0] == 1 || BMI1[0] == 1 && Bcatenin[0] == 0 && YAP1[0] == 0 && HNF1A[0] == 0 && HNF4A[0] == 0 && NFkB[0] == 0 && SNAI1[0] == 1 && ZEB1[0] == 0 && SNAI2[0] == 0 && SOX2[0] == 0 || BMI1[0] == 1 && Bcatenin[0] == 0 && YAP1[0] == 0 && HNF1A[0] == 0 && HNF4A[0] == 0 && NFkB[0] == 0 && SNAI1[0] == 1 && ZEB1[0] == 0 && SNAI2[0] == 1 || BMI1[0] == 1 && Bcatenin[0] == 0 && YAP1[0] == 0 && HNF1A[0] == 0 && HNF4A[0] == 0 && NFkB[0] == 0 && SNAI1[0] == 1 && ZEB1[0] == 1 || BMI1[0] == 1 && Bcatenin[0] == 0 && YAP1[0] == 0 && HNF1A[0] == 0 && HNF4A[0] == 0 && NFkB[0] == 1 && ZEB1[0] == 0 && SNAI2[0] == 0 && SOX2[0] == 0 || BMI1[0] == 1 && Bcatenin[0] == 0 && YAP1[0] == 0 && HNF1A[0] == 0 && HNF4A[0] == 0 && NFkB[0] == 1 && ZEB1[0] == 0 && SNAI2[0] == 1 || BMI1[0] == 1 && Bcatenin[0] == 0 && YAP1[0] == 0 && HNF1A[0] == 0 && HNF4A[0] == 0 && NFkB[0] == 1 && ZEB1[0] == 1 || BMI1[0] == 1 && Bcatenin[0] == 0 && YAP1[0] == 0 && HNF1A[0] == 1 && HNF4A[0] == 0 && FOXA2[0] == 0 && NFkB[0] == 0 && SNAI1[0] == 0 && ZEB1[0] == 0 && SNAI2[0] == 0 && SOX9[0] == 1 && SOX2[0] == 0 || BMI1[0] == 1 && Bcatenin[0] == 0 && YAP1[0] == 0 && HNF1A[0] == 1 && HNF4A[0] == 0 && FOXA2[0] == 0 && NFkB[0] == 0 && SNAI1[0] == 0 && ZEB1[0] == 0 && SNAI2[0] == 1 || BMI1[0] == 1 && Bcatenin[0] == 0 && YAP1[0] == 0 && HNF1A[0] == 1 && HNF4A[0] == 0 && FOXA2[0] == 0 && NFkB[0] == 0 && SNAI1[0] == 0 && ZEB1[0] == 1 && SNAI2[0] == 0 && SOX9[0] == 1 || BMI1[0] == 1 && Bcatenin[0] == 0 && YAP1[0] == 0 && HNF1A[0] == 1 && HNF4A[0] == 0 && FOXA2[0] == 0 && NFkB[0] == 0 && SNAI1[0] == 0 && ZEB1[0] == 1 && SNAI2[0] == 1 || BMI1[0] == 1 && Bcatenin[0] == 0 && YAP1[0] == 0 && HNF1A[0] == 1 && HNF4A[0] == 0 && FOXA2[0] == 0 && NFkB[0] == 0 && SNAI1[0] == 1 && ZEB1[0] == 0 && SNAI2[0] == 0 && SOX2[0] == 0 || BMI1[0] == 1 && Bcatenin[0] == 0 && YAP1[0] == 0 && HNF1A[0] == 1 && HNF4A[0] == 0 && FOXA2[0] == 0 && NFkB[0] == 0 && SNAI1[0] == 1 && ZEB1[0] == 0 && SNAI2[0] == 1 || BMI1[0] == 1 && Bcatenin[0] == 0 && YAP1[0] == 0 && HNF1A[0] == 1 && HNF4A[0] == 0 && FOXA2[0] == 0 && NFkB[0] == 0 && SNAI1[0] == 1 && ZEB1[0] == 1 || BMI1[0] == 1 && Bcatenin[0] == 0 && YAP1[0] == 0 && HNF1A[0] == 1 && HNF4A[0] == 0 && FOXA2[0] == 0 && NFkB[0] == 1 && ZEB1[0] == 0 && SNAI2[0] == 0 && SOX2[0] == 0 || BMI1[0] == 1 && Bcatenin[0] == 0 && YAP1[0] == 0 && HNF1A[0] == 1 && HNF4A[0] == 0 && FOXA2[0] == 0 && NFkB[0] == 1 && ZEB1[0] == 0 && SNAI2[0] == 1 || BMI1[0] == 1 && Bcatenin[0] == 0 && YAP1[0] == 0 && HNF1A[0] == 1 && HNF4A[0] == 0 && FOXA2[0] == 0 && NFkB[0] == 1 && ZEB1[0] == 1 || BMI1[0] == 1 && Bcatenin[0] == 0 && YAP1[0] == 1 && HNF1A[0] == 0 && HNF4A[0] == 0 && NFkB[0] == 0 && SNAI1[0] == 0 && ZEB1[0] == 0 && SNAI2[0] == 0 && SOX9[0] == 1 && SOX2[0] == 0 || BMI1[0] == 1 && Bcatenin[0] == 0 && YAP1[0] == 1 && HNF1A[0] == 0 && HNF4A[0] == 0 && NFkB[0] == 0 && SNAI1[0] == 0 && ZEB1[0] == 0 && SNAI2[0] == 1 || BMI1[0] == 1 && Bcatenin[0] == 0 && YAP1[0] == 1 && HNF1A[0] == 0 && HNF4A[0] == 0 && NFkB[0] == 0 && SNAI1[0] == 0 && ZEB1[0] == 1 && SNAI2[0] == 0 && SOX9[0] == 1 || BMI1[0] == 1 && Bcatenin[0] == 0 && YAP1[0] == 1 && HNF1A[0] == 0 && HNF4A[0] == 0 && NFkB[0] == 0 && SNAI1[0] == 0 && ZEB1[0] == 1 && SNAI2[0] == 1 || BMI1[0] == 1 && Bcatenin[0] == 0 && YAP1[0] == 1 && HNF1A[0] == 0 && HNF4A[0] == 0 && NFkB[0] == 0 && SNAI1[0] == 1 && ZEB1[0] == 0 && SNAI2[0] == 0 && SOX2[0] == 0 || BMI1[0] == 1 && Bcatenin[0] == 0 && YAP1[0] == 1 && HNF1A[0] == 0 && HNF4A[0] == 0 && NFkB[0] == 0 && SNAI1[0] == 1 && ZEB1[0] == 0 && SNAI2[0] == 1 || BMI1[0] == 1 && Bcatenin[0] == 0 && YAP1[0] == 1 && HNF1A[0] == 0 && HNF4A[0] == 0 && NFkB[0] == 0 && SNAI1[0] == 1 && ZEB1[0] == 1 || BMI1[0] == 1 && Bcatenin[0] == 0 && YAP1[0] == 1 && HNF1A[0] == 0 && HNF4A[0] == 0 && NFkB[0] == 1 && ZEB1[0] == 0 && SNAI2[0] == 0 && SOX2[0] == 0 || BMI1[0] == 1 && Bcatenin[0] == 0 && YAP1[0] == 1 && HNF1A[0] == 0 && HNF4A[0] == 0 && NFkB[0] == 1 && ZEB1[0] == 0 && SNAI2[0] == 1 || BMI1[0] == 1 && Bcatenin[0] == 0 && YAP1[0] == 1 && HNF1A[0] == 0 && HNF4A[0] == 0 && NFkB[0] == 1 && ZEB1[0] == 1 || BMI1[0] == 1 && Bcatenin[0] == 0 && YAP1[0] == 1 && HNF1A[0] == 0 && HNF4A[0] == 1 && FOXA2[0] == 0 && NFkB[0] == 0 && SNAI1[0] == 0 && SNAI2[0] == 1 || BMI1[0] == 1 && Bcatenin[0] == 0 && YAP1[0] == 1 && HNF1A[0] == 0 && HNF4A[0] == 1 && FOXA2[0] == 0 && NFkB[0] == 0 && SNAI1[0] == 1 && ZEB1[0] == 0 && SNAI2[0] == 1 || BMI1[0] == 1 && Bcatenin[0] == 0 && YAP1[0] == 1 && HNF1A[0] == 0 && HNF4A[0] == 1 && FOXA2[0] == 0 && NFkB[0] == 0 && SNAI1[0] == 1 && ZEB1[0] == 1 && SNAI2[0] == 0 && NANOG[0] == 1 || BMI1[0] == 1 && Bcatenin[0] == 0 && YAP1[0] == 1 && HNF1A[0] == 0 && HNF4A[0] == 1 && FOXA2[0] == 0 && NFkB[0] == 0 && SNAI1[0] == 1 && ZEB1[0] == 1 && SNAI2[0] == 1 || BMI1[0] == 1 && Bcatenin[0] == 0 && YAP1[0] == 1 && HNF1A[0] == 0 && HNF4A[0] == 1 && FOXA2[0] == 0 && NFkB[0] == 1 && ZEB1[0] == 0 && SNAI2[0] == 1 || BMI1[0] == 1 && Bcatenin[0] == 0 && YAP1[0] == 1 && HNF1A[0] == 0 && HNF4A[0] == 1 && FOXA2[0] == 0 && NFkB[0] == 1 && ZEB1[0] == 1 || BMI1[0] == 1 && Bcatenin[0] == 0 && YAP1[0] == 1 && HNF1A[0] == 0 && HNF4A[0] == 1 && FOXA2[0] == 1 && NFkB[0] == 0 && SNAI1[0] == 0 && SNAI2[0] == 1 && NANOG[0] == 1 || BMI1[0] == 1 && Bcatenin[0] == 0 && YAP1[0] == 1 && HNF1A[0] == 0 && HNF4A[0] == 1 && FOXA2[0] == 1 && NFkB[0] == 0 && SNAI1[0] == 1 && ZEB1[0] == 0 && SNAI2[0] == 1 && NANOG[0] == 1 || BMI1[0] == 1 && Bcatenin[0] == 0 && YAP1[0] == 1 && HNF1A[0] == 0 && HNF4A[0] == 1 && FOXA2[0] == 1 && NFkB[0] == 0 && SNAI1[0] == 1 && ZEB1[0] == 1 && NANOG[0] == 1 || BMI1[0] == 1 && Bcatenin[0] == 0 && YAP1[0] == 1 && HNF1A[0] == 0 && HNF4A[0] == 1 && FOXA2[0] == 1 && NFkB[0] == 1 && ZEB1[0] == 0 && SNAI2[0] == 1 && NANOG[0] == 1 || BMI1[0] == 1 && Bcatenin[0] == 0 && YAP1[0] == 1 && HNF1A[0] == 0 && HNF4A[0] == 1 && FOXA2[0] == 1 && NFkB[0] == 1 && ZEB1[0] == 1 && NANOG[0] == 1 || BMI1[0] == 1 && Bcatenin[0] == 0 && YAP1[0] == 1 && HNF1A[0] == 1 && HNF4A[0] == 0 && FOXA2[0] == 0 && NFkB[0] == 0 && SNAI1[0] == 0 && ZEB1[0] == 0 && SNAI2[0] == 0 && SOX9[0] == 1 && SOX2[0] == 0 || BMI1[0] == 1 && Bcatenin[0] == 0 && YAP1[0] == 1 && HNF1A[0] == 1 && HNF4A[0] == 0 && FOXA2[0] == 0 && NFkB[0] == 0 && SNAI1[0] == 0 && ZEB1[0] == 0 && SNAI2[0] == 1 || BMI1[0] == 1 && Bcatenin[0] == 0 && YAP1[0] == 1 && HNF1A[0] == 1 && HNF4A[0] == 0 && FOXA2[0] == 0 && NFkB[0] == 0 && SNAI1[0] == 0 && ZEB1[0] == 1 && SNAI2[0] == 0 && SOX9[0] == 1 || BMI1[0] == 1 && Bcatenin[0] == 0 && YAP1[0] == 1 && HNF1A[0] == 1 && HNF4A[0] == 0 && FOXA2[0] == 0 && NFkB[0] == 0 && SNAI1[0] == 0 && ZEB1[0] == 1 && SNAI2[0] == 1 || BMI1[0] == 1 && Bcatenin[0] == 0 && YAP1[0] == 1 && HNF1A[0] == 1 && HNF4A[0] == 0 && FOXA2[0] == 0 && NFkB[0] == 0 && SNAI1[0] == 1 && ZEB1[0] == 0 && SNAI2[0] == 0 && SOX2[0] == 0 || BMI1[0] == 1 && Bcatenin[0] == 0 && YAP1[0] == 1 && HNF1A[0] == 1 && HNF4A[0] == 0 && FOXA2[0] == 0 && NFkB[0] == 0 && SNAI1[0] == 1 && ZEB1[0] == 0 && SNAI2[0] == 1 || BMI1[0] == 1 && Bcatenin[0] == 0 && YAP1[0] == 1 && HNF1A[0] == 1 && HNF4A[0] == 0 && FOXA2[0] == 0 && NFkB[0] == 0 && SNAI1[0] == 1 && ZEB1[0] == 1 || BMI1[0] == 1 && Bcatenin[0] == 0 && YAP1[0] == 1 && HNF1A[0] == 1 && HNF4A[0] == 0 && FOXA2[0] == 0 && NFkB[0] == 1 && ZEB1[0] == 0 && SNAI2[0] == 0 && SOX2[0] == 0 || BMI1[0] == 1 && Bcatenin[0] == 0 && YAP1[0] == 1 && HNF1A[0] == 1 && HNF4A[0] == 0 && FOXA2[0] == 0 && NFkB[0] == 1 && ZEB1[0] == 0 && SNAI2[0] == 1 || BMI1[0] == 1 && Bcatenin[0] == 0 && YAP1[0] == 1 && HNF1A[0] == 1 && HNF4A[0] == 0 && FOXA2[0] == 0 && NFkB[0] == 1 && ZEB1[0] == 1 || BMI1[0] == 1 && Bcatenin[0] == 0 && YAP1[0] == 1 && HNF1A[0] == 1 && HNF4A[0] == 0 && FOXA2[0] == 1 && NFkB[0] == 0 && SNAI1[0] == 0 && SNAI2[0] == 1 && NANOG[0] == 1 || BMI1[0] == 1 && Bcatenin[0] == 0 && YAP1[0] == 1 && HNF1A[0] == 1 && HNF4A[0] == 0 && FOXA2[0] == 1 && NFkB[0] == 0 && SNAI1[0] == 1 && ZEB1[0] == 0 && SNAI2[0] == 0 && NANOG[0] == 1 && SOX2[0] == 0 || BMI1[0] == 1 && Bcatenin[0] == 0 && YAP1[0] == 1 && HNF1A[0] == 1 && HNF4A[0] == 0 && FOXA2[0] == 1 && NFkB[0] == 0 && SNAI1[0] == 1 && ZEB1[0] == 0 && SNAI2[0] == 1 && NANOG[0] == 1 || BMI1[0] == 1 && Bcatenin[0] == 0 && YAP1[0] == 1 && HNF1A[0] == 1 && HNF4A[0] == 0 && FOXA2[0] == 1 && NFkB[0] == 0 && SNAI1[0] == 1 && ZEB1[0] == 1 && NANOG[0] == 1 || BMI1[0] == 1 && Bcatenin[0] == 0 && YAP1[0] == 1 && HNF1A[0] == 1 && HNF4A[0] == 0 && FOXA2[0] == 1 && NFkB[0] == 1 && SNAI1[0] == 0 && ZEB1[0] == 0 && SNAI2[0] == 0 && NANOG[0] == 1 && SOX2[0] == 0 && p53[0] == 0 || BMI1[0] == 1 && Bcatenin[0] == 0 && YAP1[0] == 1 && HNF1A[0] == 1 && HNF4A[0] == 0 && FOXA2[0] == 1 && NFkB[0] == 1 && SNAI1[0] == 0 && ZEB1[0] == 0 && SNAI2[0] == 1 && NANOG[0] == 1 || BMI1[0] == 1 && Bcatenin[0] == 0 && YAP1[0] == 1 && HNF1A[0] == 1 && HNF4A[0] == 0 && FOXA2[0] == 1 && NFkB[0] == 1 && SNAI1[0] == 0 && ZEB1[0] == 1 && NANOG[0] == 1 || BMI1[0] == 1 && Bcatenin[0] == 0 && YAP1[0] == 1 && HNF1A[0] == 1 && HNF4A[0] == 0 && FOXA2[0] == 1 && NFkB[0] == 1 && SNAI1[0] == 1 && ZEB1[0] == 0 && SNAI2[0] == 0 && NANOG[0] == 1 && SOX2[0] == 0 || BMI1[0] == 1 && Bcatenin[0] == 0 && YAP1[0] == 1 && HNF1A[0] == 1 && HNF4A[0] == 0 && FOXA2[0] == 1 && NFkB[0] == 1 && SNAI1[0] == 1 && ZEB1[0] == 0 && SNAI2[0] == 1 && NANOG[0] == 1 || BMI1[0] == 1 && Bcatenin[0] == 0 && YAP1[0] == 1 && HNF1A[0] == 1 && HNF4A[0] == 0 && FOXA2[0] == 1 && NFkB[0] == 1 && SNAI1[0] == 1 && ZEB1[0] == 1 && NANOG[0] == 1 || BMI1[0] == 1 && Bcatenin[0] == 1 && HNF1A[0] == 0 && HNF4A[0] == 0 && NFkB[0] == 0 && SNAI1[0] == 0 && ZEB1[0] == 0 && SNAI2[0] == 0 && SOX9[0] == 1 && SOX2[0] == 0 || BMI1[0] == 1 && Bcatenin[0] == 1 && HNF1A[0] == 0 && HNF4A[0] == 0 && NFkB[0] == 0 && SNAI1[0] == 0 && ZEB1[0] == 0 && SNAI2[0] == 1 || BMI1[0] == 1 && Bcatenin[0] == 1 && HNF1A[0] == 0 && HNF4A[0] == 0 && NFkB[0] == 0 && SNAI1[0] == 0 && ZEB1[0] == 1 && SNAI2[0] == 0 && SOX9[0] == 1 || BMI1[0] == 1 && Bcatenin[0] == 1 && HNF1A[0] == 0 && HNF4A[0] == 0 && NFkB[0] == 0 && SNAI1[0] == 0 && ZEB1[0] == 1 && SNAI2[0] == 1 || BMI1[0] == 1 && Bcatenin[0] == 1 && HNF1A[0] == 0 && HNF4A[0] == 0 && NFkB[0] == 0 && SNAI1[0] == 1 && ZEB1[0] == 0 && SNAI2[0] == 0 && SOX2[0] == 0 || BMI1[0] == 1 && Bcatenin[0] == 1 && HNF1A[0] == 0 && HNF4A[0] == 0 && NFkB[0] == 0 && SNAI1[0] == 1 && ZEB1[0] == 0 && SNAI2[0] == 1 || BMI1[0] == 1 && Bcatenin[0] == 1 && HNF1A[0] == 0 && HNF4A[0] == 0 && NFkB[0] == 0 && SNAI1[0] == 1 && ZEB1[0] == 1 || BMI1[0] == 1 && Bcatenin[0] == 1 && HNF1A[0] == 0 && HNF4A[0] == 0 && NFkB[0] == 1 && ZEB1[0] == 0 && SNAI2[0] == 0 && SOX2[0] == 0 || BMI1[0] == 1 && Bcatenin[0] == 1 && HNF1A[0] == 0 && HNF4A[0] == 0 && NFkB[0] == 1 && ZEB1[0] == 0 && SNAI2[0] == 1 || BMI1[0] == 1 && Bcatenin[0] == 1 && HNF1A[0] == 0 && HNF4A[0] == 0 && NFkB[0] == 1 && ZEB1[0] == 1 || BMI1[0] == 1 && Bcatenin[0] == 1 && HNF1A[0] == 0 && HNF4A[0] == 1 && FOXA2[0] == 0 && NFkB[0] == 0 && SNAI1[0] == 0 && SNAI2[0] == 1 || BMI1[0] == 1 && Bcatenin[0] == 1 && HNF1A[0] == 0 && HNF4A[0] == 1 && FOXA2[0] == 0 && NFkB[0] == 0 && SNAI1[0] == 1 && ZEB1[0] == 0 && SNAI2[0] == 1 || BMI1[0] == 1 && Bcatenin[0] == 1 && HNF1A[0] == 0 && HNF4A[0] == 1 && FOXA2[0] == 0 && NFkB[0] == 0 && SNAI1[0] == 1 && ZEB1[0] == 1 && SNAI2[0] == 0 && NANOG[0] == 1 || BMI1[0] == 1 && Bcatenin[0] == 1 && HNF1A[0] == 0 && HNF4A[0] == 1 && FOXA2[0] == 0 && NFkB[0] == 0 && SNAI1[0] == 1 && ZEB1[0] == 1 && SNAI2[0] == 1 || BMI1[0] == 1 && Bcatenin[0] == 1 && HNF1A[0] == 0 && HNF4A[0] == 1 && FOXA2[0] == 0 && NFkB[0] == 1 && ZEB1[0] == 0 && SNAI2[0] == 1 || BMI1[0] == 1 && Bcatenin[0] == 1 && HNF1A[0] == 0 && HNF4A[0] == 1 && FOXA2[0] == 0 && NFkB[0] == 1 && ZEB1[0] == 1 || BMI1[0] == 1 && Bcatenin[0] == 1 && HNF1A[0] == 0 && HNF4A[0] == 1 && FOXA2[0] == 1 && NFkB[0] == 0 && SNAI1[0] == 0 && SNAI2[0] == 1 && NANOG[0] == 1 || BMI1[0] == 1 && Bcatenin[0] == 1 && HNF1A[0] == 0 && HNF4A[0] == 1 && FOXA2[0] == 1 && NFkB[0] == 0 && SNAI1[0] == 1 && ZEB1[0] == 0 && SNAI2[0] == 1 && NANOG[0] == 1 || BMI1[0] == 1 && Bcatenin[0] == 1 && HNF1A[0] == 0 && HNF4A[0] == 1 && FOXA2[0] == 1 && NFkB[0] == 0 && SNAI1[0] == 1 && ZEB1[0] == 1 && NANOG[0] == 1 || BMI1[0] == 1 && Bcatenin[0] == 1 && HNF1A[0] == 0 && HNF4A[0] == 1 && FOXA2[0] == 1 && NFkB[0] == 1 && ZEB1[0] == 0 && SNAI2[0] == 1 && NANOG[0] == 1 || BMI1[0] == 1 && Bcatenin[0] == 1 && HNF1A[0] == 0 && HNF4A[0] == 1 && FOXA2[0] == 1 && NFkB[0] == 1 && ZEB1[0] == 1 && NANOG[0] == 1 || BMI1[0] == 1 && Bcatenin[0] == 1 && HNF1A[0] == 1 && HNF4A[0] == 0 && FOXA2[0] == 0 && NFkB[0] == 0 && SNAI1[0] == 0 && ZEB1[0] == 0 && SNAI2[0] == 0 && SOX9[0] == 1 && SOX2[0] == 0 || BMI1[0] == 1 && Bcatenin[0] == 1 && HNF1A[0] == 1 && HNF4A[0] == 0 && FOXA2[0] == 0 && NFkB[0] == 0 && SNAI1[0] == 0 && ZEB1[0] == 0 && SNAI2[0] == 1 || BMI1[0] == 1 && Bcatenin[0] == 1 && HNF1A[0] == 1 && HNF4A[0] == 0 && FOXA2[0] == 0 && NFkB[0] == 0 && SNAI1[0] == 0 && ZEB1[0] == 1 && SNAI2[0] == 0 && SOX9[0] == 1 || BMI1[0] == 1 && Bcatenin[0] == 1 && HNF1A[0] == 1 && HNF4A[0] == 0 && FOXA2[0] == 0 && NFkB[0] == 0 && SNAI1[0] == 0 && ZEB1[0] == 1 && SNAI2[0] == 1 || BMI1[0] == 1 && Bcatenin[0] == 1 && HNF1A[0] == 1 && HNF4A[0] == 0 && FOXA2[0] == 0 && NFkB[0] == 0 && SNAI1[0] == 1 && ZEB1[0] == 0 && SNAI2[0] == 0 && SOX2[0] == 0 || BMI1[0] == 1 && Bcatenin[0] == 1 && HNF1A[0] == 1 && HNF4A[0] == 0 && FOXA2[0] == 0 && NFkB[0] == 0 && SNAI1[0] == 1 && ZEB1[0] == 0 && SNAI2[0] == 1 || BMI1[0] == 1 && Bcatenin[0] == 1 && HNF1A[0] == 1 && HNF4A[0] == 0 && FOXA2[0] == 0 && NFkB[0] == 0 && SNAI1[0] == 1 && ZEB1[0] == 1 || BMI1[0] == 1 && Bcatenin[0] == 1 && HNF1A[0] == 1 && HNF4A[0] == 0 && FOXA2[0] == 0 && NFkB[0] == 1 && ZEB1[0] == 0 && SNAI2[0] == 0 && SOX2[0] == 0 || BMI1[0] == 1 && Bcatenin[0] == 1 && HNF1A[0] == 1 && HNF4A[0] == 0 && FOXA2[0] == 0 && NFkB[0] == 1 && ZEB1[0] == 0 && SNAI2[0] == 1 || BMI1[0] == 1 && Bcatenin[0] == 1 && HNF1A[0] == 1 && HNF4A[0] == 0 && FOXA2[0] == 0 && NFkB[0] == 1 && ZEB1[0] == 1 || BMI1[0] == 1 && Bcatenin[0] == 1 && HNF1A[0] == 1 && HNF4A[0] == 0 && FOXA2[0] == 1 && NFkB[0] == 0 && SNAI1[0] == 0 && SNAI2[0] == 1 && NANOG[0] == 1 || BMI1[0] == 1 && Bcatenin[0] == 1 && HNF1A[0] == 1 && HNF4A[0] == 0 && FOXA2[0] == 1 && NFkB[0] == 0 && SNAI1[0] == 1 && ZEB1[0] == 0 && SNAI2[0] == 0 && NANOG[0] == 1 && SOX2[0] == 0 || BMI1[0] == 1 && Bcatenin[0] == 1 && HNF1A[0] == 1 && HNF4A[0] == 0 && FOXA2[0] == 1 && NFkB[0] == 0 && SNAI1[0] == 1 && ZEB1[0] == 0 && SNAI2[0] == 1 && NANOG[0] == 1 || BMI1[0] == 1 && Bcatenin[0] == 1 && HNF1A[0] == 1 && HNF4A[0] == 0 && FOXA2[0] == 1 && NFkB[0] == 0 && SNAI1[0] == 1 && ZEB1[0] == 1 && NANOG[0] == 1 || BMI1[0] == 1 && Bcatenin[0] == 1 && HNF1A[0] == 1 && HNF4A[0] == 0 && FOXA2[0] == 1 && NFkB[0] == 1 && SNAI1[0] == 0 && ZEB1[0] == 0 && SNAI2[0] == 0 && NANOG[0] == 1 && SOX2[0] == 0 && p53[0] == 0 || BMI1[0] == 1 && Bcatenin[0] == 1 && HNF1A[0] == 1 && HNF4A[0] == 0 && FOXA2[0] == 1 && NFkB[0] == 1 && SNAI1[0] == 0 && ZEB1[0] == 0 && SNAI2[0] == 1 && NANOG[0] == 1 || BMI1[0] == 1 && Bcatenin[0] == 1 && HNF1A[0] == 1 && HNF4A[0] == 0 && FOXA2[0] == 1 && NFkB[0] == 1 && SNAI1[0] == 0 && ZEB1[0] == 1 && NANOG[0] == 1 || BMI1[0] == 1 && Bcatenin[0] == 1 && HNF1A[0] == 1 && HNF4A[0] == 0 && FOXA2[0] == 1 && NFkB[0] == 1 && SNAI1[0] == 1 && ZEB1[0] == 0 && SNAI2[0] == 0 && NANOG[0] == 1 && SOX2[0] == 0 || BMI1[0] == 1 && Bcatenin[0] == 1 && HNF1A[0] == 1 && HNF4A[0] == 0 && FOXA2[0] == 1 && NFkB[0] == 1 && SNAI1[0] == 1 && ZEB1[0] == 0 && SNAI2[0] == 1 && NANOG[0] == 1 || BMI1[0] == 1 && Bcatenin[0] == 1 && HNF1A[0] == 1 && HNF4A[0] == 0 && FOXA2[0] == 1 && NFkB[0] == 1 && SNAI1[0] == 1 && ZEB1[0] == 1 && NANOG[0] == 1) { SNAI2[0] = 1; }

else { SNAI2[0] = 0; }

//Logic rule for ZEB1

if (BMI1[0] == 0 && E2F[0] == 0 && Bcatenin[0] == 0 && YAP1[0] == 0 && HNF1A[0] == 0 && HNF4A[0] == 0 && NFkB[0] == 0 && SNAI1[0] == 0 && SNAI2[0] == 1 || BMI1[0] == 0 && E2F[0] == 0 && Bcatenin[0] == 0 && YAP1[0] == 0 && HNF1A[0] == 0 && HNF4A[0] == 0 && NFkB[0] == 0 && SNAI1[0] == 1 || BMI1[0] == 0 && E2F[0] == 0 && Bcatenin[0] == 0 && YAP1[0] == 0 && HNF1A[0] == 0 && HNF4A[0] == 0 && NFkB[0] == 1 && SNAI1[0] == 0 && ZEB1[0] == 0 && SNAI2[0] == 0 && SOX9[0] == 0 || BMI1[0] == 0 && E2F[0] == 0 && Bcatenin[0] == 0 && YAP1[0] == 0 && HNF1A[0] == 0 && HNF4A[0] == 0 && NFkB[0] == 1 && SNAI1[0] == 0 && ZEB1[0] == 0 && SNAI2[0] == 0 && SOX9[0] == 1 && OCT4[0] == 0 || BMI1[0] == 0 && E2F[0] == 0 && Bcatenin[0] == 0 && YAP1[0] == 0 && HNF1A[0] == 0 && HNF4A[0] == 0 && NFkB[0] == 1 && SNAI1[0] == 0 && ZEB1[0] == 0 && SNAI2[0] == 0 && SOX9[0] == 1 && OCT4[0] == 1 && NANOG[0] == 0 && SOX2[0] == 0 || BMI1[0] == 0 && E2F[0] == 0 && Bcatenin[0] == 0 && YAP1[0] == 0 && HNF1A[0] == 0 && HNF4A[0] == 0 && NFkB[0] == 1 && SNAI1[0] == 0 && ZEB1[0] == 0 && SNAI2[0] == 0 && SOX9[0] == 1 && OCT4[0] == 1 && NANOG[0] == 0 && SOX2[0] == 1 && p53[0] == 0 || BMI1[0] == 0 && E2F[0] == 0 && Bcatenin[0] == 0 && YAP1[0] == 0 && HNF1A[0] == 0 && HNF4A[0] == 0 && NFkB[0] == 1 && SNAI1[0] == 0 && ZEB1[0] == 0 && SNAI2[0] == 0 && SOX9[0] == 1 && OCT4[0] == 1 && NANOG[0] == 1 || BMI1[0] == 0 && E2F[0] == 0 && Bcatenin[0] == 0 && YAP1[0] == 0 && HNF1A[0] == 0 && HNF4A[0] == 0 && NFkB[0] == 1 && SNAI1[0] == 0 && ZEB1[0] == 0 && SNAI2[0] == 1 || BMI1[0] == 0 && E2F[0] == 0 && Bcatenin[0] == 0 && YAP1[0] == 0 && HNF1A[0] == 0 && HNF4A[0] == 0 && NFkB[0] == 1 && SNAI1[0] == 0 && ZEB1[0] == 1 || BMI1[0] == 0 && E2F[0] == 0 && Bcatenin[0] == 0 && YAP1[0] == 0 && HNF1A[0] == 0 && HNF4A[0] == 0 && NFkB[0] == 1 && SNAI1[0] == 1 || BMI1[0] == 0 && E2F[0] == 0 && Bcatenin[0] == 0 && YAP1[0] == 0 && HNF1A[0] == 0 && HNF4A[0] == 1 && NFkB[0] == 0 && SNAI1[0] == 0 && SNAI2[0] == 1 || BMI1[0] == 0 && E2F[0] == 0 && Bcatenin[0] == 0 && YAP1[0] == 0 && HNF1A[0] == 0 && HNF4A[0] == 1 && NFkB[0] == 0 && SNAI1[0] == 1 || BMI1[0] == 0 && E2F[0] == 0 && Bcatenin[0] == 0 && YAP1[0] == 0 && HNF1A[0] == 0 && HNF4A[0] == 1 && NFkB[0] == 1 && SNAI1[0] == 0 && ZEB1[0] == 0 && SNAI2[0] == 0 && SOX9[0] == 0 && NANOG[0] == 0 && SOX2[0] == 0 || BMI1[0] == 0 && E2F[0] == 0 && Bcatenin[0] == 0 && YAP1[0] == 0 && HNF1A[0] == 0 && HNF4A[0] == 1 && NFkB[0] == 1 && SNAI1[0] == 0 && ZEB1[0] == 0 && SNAI2[0] == 0 && SOX9[0] == 0 && NANOG[0] == 0 && SOX2[0] == 1 && p53[0] == 1 || BMI1[0] == 0 && E2F[0] == 0 && Bcatenin[0] == 0 && YAP1[0] == 0 && HNF1A[0] == 0 && HNF4A[0] == 1 && NFkB[0] == 1 && SNAI1[0] == 0 && ZEB1[0] == 0 && SNAI2[0] == 0 && SOX9[0] == 0 && NANOG[0] == 1 || BMI1[0] == 0 && E2F[0] == 0 && Bcatenin[0] == 0 && YAP1[0] == 0 && HNF1A[0] == 0 && HNF4A[0] == 1 && NFkB[0] == 1 && SNAI1[0] == 0 && ZEB1[0] == 0 && SNAI2[0] == 0 && SOX9[0] == 1 && NANOG[0] == 0 && SOX2[0] == 0 || BMI1[0] == 0 && E2F[0] == 0 && Bcatenin[0] == 0 && YAP1[0] == 0 && HNF1A[0] == 0 && HNF4A[0] == 1 && NFkB[0] == 1 && SNAI1[0] == 0 && ZEB1[0] == 0 && SNAI2[0] == 0 && SOX9[0] == 1 && NANOG[0] == 1 || BMI1[0] == 0 && E2F[0] == 0 && Bcatenin[0] == 0 && YAP1[0] == 0 && HNF1A[0] == 0 && HNF4A[0] == 1 && NFkB[0] == 1 && SNAI1[0] == 0 && ZEB1[0] == 0 && SNAI2[0] == 1 || BMI1[0] == 0 && E2F[0] == 0 && Bcatenin[0] == 0 && YAP1[0] == 0 && HNF1A[0] == 0 && HNF4A[0] == 1 && NFkB[0] == 1 && SNAI1[0] == 0 && ZEB1[0] == 1 || BMI1[0] == 0 && E2F[0] == 0 && Bcatenin[0] == 0 && YAP1[0] == 0 && HNF1A[0] == 0 && HNF4A[0] == 1 && NFkB[0] == 1 && SNAI1[0] == 1 || BMI1[0] == 0 && E2F[0] == 0 && Bcatenin[0] == 0 && YAP1[0] == 1 && HNF1A[0] == 0 && HNF4A[0] == 0 && NFkB[0] == 0 && SNAI1[0] == 0 && SNAI2[0] == 1 || BMI1[0] == 0 && E2F[0] == 0 && Bcatenin[0] == 0 && YAP1[0] == 1 && HNF1A[0] == 0 && HNF4A[0] == 0 && NFkB[0] == 0 && SNAI1[0] == 1 || BMI1[0] == 0 && E2F[0] == 0 && Bcatenin[0] == 0 && YAP1[0] == 1 && HNF1A[0] == 0 && HNF4A[0] == 0 && NFkB[0] == 1 && SNAI1[0] == 0 && ZEB1[0] == 0 && SNAI2[0] == 0 && SOX9[0] == 0 || BMI1[0] == 0 && E2F[0] == 0 && Bcatenin[0] == 0 && YAP1[0] == 1 && HNF1A[0] == 0 && HNF4A[0] == 0 && NFkB[0] == 1 && SNAI1[0] == 0 && ZEB1[0] == 0 && SNAI2[0] == 0 && SOX9[0] == 1 && OCT4[0] == 0 || BMI1[0] == 0 && E2F[0] == 0 && Bcatenin[0] == 0 && YAP1[0] == 1 && HNF1A[0] == 0 && HNF4A[0] == 0 && NFkB[0] == 1 && SNAI1[0] == 0 && ZEB1[0] == 0 && SNAI2[0] == 0 && SOX9[0] == 1 && OCT4[0] == 1 && NANOG[0] == 0 && SOX2[0] == 0 || BMI1[0] == 0 && E2F[0] == 0 && Bcatenin[0] == 0 && YAP1[0] == 1 && HNF1A[0] == 0 && HNF4A[0] == 0 && NFkB[0] == 1 && SNAI1[0] == 0 && ZEB1[0] == 0 && SNAI2[0] == 0 && SOX9[0] == 1 && OCT4[0] == 1 && NANOG[0] == 0 && SOX2[0] == 1 && p53[0] == 0 || BMI1[0] == 0 && E2F[0] == 0 && Bcatenin[0] == 0 && YAP1[0] == 1 && HNF1A[0] == 0 && HNF4A[0] == 0 && NFkB[0] == 1 && SNAI1[0] == 0 && ZEB1[0] == 0 && SNAI2[0] == 0 && SOX9[0] == 1 && OCT4[0] == 1 && NANOG[0] == 1 || BMI1[0] == 0 && E2F[0] == 0 && Bcatenin[0] == 0 && YAP1[0] == 1 && HNF1A[0] == 0 && HNF4A[0] == 0 && NFkB[0] == 1 && SNAI1[0] == 0 && ZEB1[0] == 0 && SNAI2[0] == 1 || BMI1[0] == 0 && E2F[0] == 0 && Bcatenin[0] == 0 && YAP1[0] == 1 && HNF1A[0] == 0 && HNF4A[0] == 0 && NFkB[0] == 1 && SNAI1[0] == 0 && ZEB1[0] == 1 || BMI1[0] == 0 && E2F[0] == 0 && Bcatenin[0] == 0 && YAP1[0] == 1 && HNF1A[0] == 0 && HNF4A[0] == 0 && NFkB[0] == 1 && SNAI1[0] == 1 || BMI1[0] == 0 && E2F[0] == 0 && Bcatenin[0] == 0 && YAP1[0] == 1 && HNF1A[0] == 0 && HNF4A[0] == 1 && NFkB[0] == 0 && SNAI1[0] == 0 && SNAI2[0] == 1 || BMI1[0] == 0 && E2F[0] == 0 && Bcatenin[0] == 0 && YAP1[0] == 1 && HNF1A[0] == 0 && HNF4A[0] == 1 && NFkB[0] == 0 && SNAI1[0] == 1 || BMI1[0] == 0 && E2F[0] == 0 && Bcatenin[0] == 0 && YAP1[0] == 1 && HNF1A[0] == 0 && HNF4A[0] == 1 && NFkB[0] == 1 && SNAI1[0] == 0 && ZEB1[0] == 0 && SNAI2[0] == 0 && SOX9[0] == 0 || BMI1[0] == 0 && E2F[0] == 0 && Bcatenin[0] == 0 && YAP1[0] == 1 && HNF1A[0] == 0 && HNF4A[0] == 1 && NFkB[0] == 1 && SNAI1[0] == 0 && ZEB1[0] == 0 && SNAI2[0] == 0 && SOX9[0] == 1 && NANOG[0] == 0 && SOX2[0] == 0 || BMI1[0] == 0 && E2F[0] == 0 && Bcatenin[0] == 0 && YAP1[0] == 1 && HNF1A[0] == 0 && HNF4A[0] == 1 && NFkB[0] == 1 && SNAI1[0] == 0 && ZEB1[0] == 0 && SNAI2[0] == 0 && SOX9[0] == 1 && NANOG[0] == 0 && SOX2[0] == 1 && p53[0] == 0 || BMI1[0] == 0 && E2F[0] == 0 && Bcatenin[0] == 0 && YAP1[0] == 1 && HNF1A[0] == 0 && HNF4A[0] == 1 && NFkB[0] == 1 && SNAI1[0] == 0 && ZEB1[0] == 0 && SNAI2[0] == 0 && SOX9[0] == 1 && NANOG[0] == 1 || BMI1[0] == 0 && E2F[0] == 0 && Bcatenin[0] == 0 && YAP1[0] == 1 && HNF1A[0] == 0 && HNF4A[0] == 1 && NFkB[0] == 1 && SNAI1[0] == 0 && ZEB1[0] == 0 && SNAI2[0] == 1 || BMI1[0] == 0 && E2F[0] == 0 && Bcatenin[0] == 0 && YAP1[0] == 1 && HNF1A[0] == 0 && HNF4A[0] == 1 && NFkB[0] == 1 && SNAI1[0] == 0 && ZEB1[0] == 1 || BMI1[0] == 0 && E2F[0] == 0 && Bcatenin[0] == 0 && YAP1[0] == 1 && HNF1A[0] == 0 && HNF4A[0] == 1 && NFkB[0] == 1 && SNAI1[0] == 1 || BMI1[0] == 0 && E2F[0] == 0 && Bcatenin[0] == 1 && YAP1[0] == 0 && HNF1A[0] == 0 && HNF4A[0] == 0 && SNAI1[0] == 0 && ZEB1[0] == 0 && SNAI2[0] == 0 && SOX9[0] == 0 || BMI1[0] == 0 && E2F[0] == 0 && Bcatenin[0] == 1 && YAP1[0] == 0 && HNF1A[0] == 0 && HNF4A[0] == 0 && SNAI1[0] == 0 && ZEB1[0] == 0 && SNAI2[0] == 0 && SOX9[0] == 1 && OCT4[0] == 0 || BMI1[0] == 0 && E2F[0] == 0 && Bcatenin[0] == 1 && YAP1[0] == 0 && HNF1A[0] == 0 && HNF4A[0] == 0 && SNAI1[0] == 0 && ZEB1[0] == 0 && SNAI2[0] == 0 && SOX9[0] == 1 && OCT4[0] == 1 && NANOG[0] == 0 && SOX2[0] == 0 || BMI1[0] == 0 && E2F[0] == 0 && Bcatenin[0] == 1 && YAP1[0] == 0 && HNF1A[0] == 0 && HNF4A[0] == 0 && SNAI1[0] == 0 && ZEB1[0] == 0 && SNAI2[0] == 0 && SOX9[0] == 1 && OCT4[0] == 1 && NANOG[0] == 0 && SOX2[0] == 1 && p53[0] == 0 || BMI1[0] == 0 && E2F[0] == 0 && Bcatenin[0] == 1 && YAP1[0] == 0 && HNF1A[0] == 0 && HNF4A[0] == 0 && SNAI1[0] == 0 && ZEB1[0] == 0 && SNAI2[0] == 0 && SOX9[0] == 1 && OCT4[0] == 1 && NANOG[0] == 1 || BMI1[0] == 0 && E2F[0] == 0 && Bcatenin[0] == 1 && YAP1[0] == 0 && HNF1A[0] == 0 && HNF4A[0] == 0 && SNAI1[0] == 0 && ZEB1[0] == 0 && SNAI2[0] == 1 || BMI1[0] == 0 && E2F[0] == 0 && Bcatenin[0] == 1 && YAP1[0] == 0 && HNF1A[0] == 0 && HNF4A[0] == 0 && SNAI1[0] == 0 && ZEB1[0] == 1 || BMI1[0] == 0 && E2F[0] == 0 && Bcatenin[0] == 1 && YAP1[0] == 0 && HNF1A[0] == 0 && HNF4A[0] == 0 && SNAI1[0] == 1 || BMI1[0] == 0 && E2F[0] == 0 && Bcatenin[0] == 1 && YAP1[0] == 0 && HNF1A[0] == 0 && HNF4A[0] == 1 && SNAI1[0] == 0 && ZEB1[0] == 0 && SNAI2[0] == 0 && TGFB[0] == 0 && SOX9[0] == 0 || BMI1[0] == 0 && E2F[0] == 0 && Bcatenin[0] == 1 && YAP1[0] == 0 && HNF1A[0] == 0 && HNF4A[0] == 1 && SNAI1[0] == 0 && ZEB1[0] == 0 && SNAI2[0] == 0 && TGFB[0] == 0 && SOX9[0] == 1 && NANOG[0] == 0 && SOX2[0] == 0 || BMI1[0] == 0 && E2F[0] == 0 && Bcatenin[0] == 1 && YAP1[0] == 0 && HNF1A[0] == 0 && HNF4A[0] == 1 && SNAI1[0] == 0 && ZEB1[0] == 0 && SNAI2[0] == 0 && TGFB[0] == 0 && SOX9[0] == 1 && NANOG[0] == 0 && SOX2[0] == 1 && p53[0] == 0 || BMI1[0] == 0 && E2F[0] == 0 && Bcatenin[0] == 1 && YAP1[0] == 0 && HNF1A[0] == 0 && HNF4A[0] == 1 && SNAI1[0] == 0 && ZEB1[0] == 0 && SNAI2[0] == 0 && TGFB[0] == 0 && SOX9[0] == 1 && NANOG[0] == 1 || BMI1[0] == 0 && E2F[0] == 0 && Bcatenin[0] == 1 && YAP1[0] == 0 && HNF1A[0] == 0 && HNF4A[0] == 1 && SNAI1[0] == 0 && ZEB1[0] == 0 && SNAI2[0] == 0 && TGFB[0] == 1 && SOX9[0] == 0 && NANOG[0] == 0 && SOX2[0] == 0 || BMI1[0] == 0 && E2F[0] == 0 && Bcatenin[0] == 1 && YAP1[0] == 0 && HNF1A[0] == 0 && HNF4A[0] == 1 && SNAI1[0] == 0 && ZEB1[0] == 0 && SNAI2[0] == 0 && TGFB[0] == 1 && SOX9[0] == 0 && NANOG[0] == 0 && SOX2[0] == 1 && p53[0] == 1 || BMI1[0] == 0 && E2F[0] == 0 && Bcatenin[0] == 1 && YAP1[0] == 0 && HNF1A[0] == 0 && HNF4A[0] == 1 && SNAI1[0] == 0 && ZEB1[0] == 0 && SNAI2[0] == 0 && TGFB[0] == 1 && SOX9[0] == 0 && NANOG[0] == 1 || BMI1[0] == 0 && E2F[0] == 0 && Bcatenin[0] == 1 && YAP1[0] == 0 && HNF1A[0] == 0 && HNF4A[0] == 1 && SNAI1[0] == 0 && ZEB1[0] == 0 && SNAI2[0] == 0 && TGFB[0] == 1 && SOX9[0] == 1 && NANOG[0] == 0 && SOX2[0] == 0 || BMI1[0] == 0 && E2F[0] == 0 && Bcatenin[0] == 1 && YAP1[0] == 0 && HNF1A[0] == 0 && HNF4A[0] == 1 && SNAI1[0] == 0 && ZEB1[0] == 0 && SNAI2[0] == 0 && TGFB[0] == 1 && SOX9[0] == 1 && NANOG[0] == 1 || BMI1[0] == 0 && E2F[0] == 0 && Bcatenin[0] == 1 && YAP1[0] == 0 && HNF1A[0] == 0 && HNF4A[0] == 1 && SNAI1[0] == 0 && ZEB1[0] == 0 && SNAI2[0] == 1 || BMI1[0] == 0 && E2F[0] == 0 && Bcatenin[0] == 1 && YAP1[0] == 0 && HNF1A[0] == 0 && HNF4A[0] == 1 && SNAI1[0] == 0 && ZEB1[0] == 1 || BMI1[0] == 0 && E2F[0] == 0 && Bcatenin[0] == 1 && YAP1[0] == 0 && HNF1A[0] == 0 && HNF4A[0] == 1 && SNAI1[0] == 1 || BMI1[0] == 0 && E2F[0] == 0 && Bcatenin[0] == 1 && YAP1[0] == 1 && HNF1A[0] == 0 && HNF4A[0] == 0 && SNAI1[0] == 0 && ZEB1[0] == 0 && SNAI2[0] == 0 && SOX9[0] == 0 || BMI1[0] == 0 && E2F[0] == 0 && Bcatenin[0] == 1 && YAP1[0] == 1 && HNF1A[0] == 0 && HNF4A[0] == 0 && SNAI1[0] == 0 && ZEB1[0] == 0 && SNAI2[0] == 0 && SOX9[0] == 1 && OCT4[0] == 0 || BMI1[0] == 0 && E2F[0] == 0 && Bcatenin[0] == 1 && YAP1[0] == 1 && HNF1A[0] == 0 && HNF4A[0] == 0 && SNAI1[0] == 0 && ZEB1[0] == 0 && SNAI2[0] == 0 && SOX9[0] == 1 && OCT4[0] == 1 && NANOG[0] == 0 && SOX2[0] == 0 || BMI1[0] == 0 && E2F[0] == 0 && Bcatenin[0] == 1 && YAP1[0] == 1 && HNF1A[0] == 0 && HNF4A[0] == 0 && SNAI1[0] == 0 && ZEB1[0] == 0 && SNAI2[0] == 0 && SOX9[0] == 1 && OCT4[0] == 1 && NANOG[0] == 0 && SOX2[0] == 1 && p53[0] == 0 || BMI1[0] == 0 && E2F[0] == 0 && Bcatenin[0] == 1 && YAP1[0] == 1 && HNF1A[0] == 0 && HNF4A[0] == 0 && SNAI1[0] == 0 && ZEB1[0] == 0 && SNAI2[0] == 0 && SOX9[0] == 1 && OCT4[0] == 1 && NANOG[0] == 1 || BMI1[0] == 0 && E2F[0] == 0 && Bcatenin[0] == 1 && YAP1[0] == 1 && HNF1A[0] == 0 && HNF4A[0] == 0 && SNAI1[0] == 0 && ZEB1[0] == 0 && SNAI2[0] == 1 || BMI1[0] == 0 && E2F[0] == 0 && Bcatenin[0] == 1 && YAP1[0] == 1 && HNF1A[0] == 0 && HNF4A[0] == 0 && SNAI1[0] == 0 && ZEB1[0] == 1 || BMI1[0] == 0 && E2F[0] == 0 && Bcatenin[0] == 1 && YAP1[0] == 1 && HNF1A[0] == 0 && HNF4A[0] == 0 && SNAI1[0] == 1 || BMI1[0] == 0 && E2F[0] == 0 && Bcatenin[0] == 1 && YAP1[0] == 1 && HNF1A[0] == 0 && HNF4A[0] == 1 && NFkB[0] == 0 && SNAI1[0] == 0 && ZEB1[0] == 0 && SNAI2[0] == 0 && TGFB[0] == 0 && SOX9[0] == 0 || BMI1[0] == 0 && E2F[0] == 0 && Bcatenin[0] == 1 && YAP1[0] == 1 && HNF1A[0] == 0 && HNF4A[0] == 1 && NFkB[0] == 0 && SNAI1[0] == 0 && ZEB1[0] == 0 && SNAI2[0] == 0 && TGFB[0] == 0 && SOX9[0] == 1 && NANOG[0] == 0 && SOX2[0] == 0 || BMI1[0] == 0 && E2F[0] == 0 && Bcatenin[0] == 1 && YAP1[0] == 1 && HNF1A[0] == 0 && HNF4A[0] == 1 && NFkB[0] == 0 && SNAI1[0] == 0 && ZEB1[0] == 0 && SNAI2[0] == 0 && TGFB[0] == 0 && SOX9[0] == 1 && NANOG[0] == 0 && SOX2[0] == 1 && p53[0] == 0 || BMI1[0] == 0 && E2F[0] == 0 && Bcatenin[0] == 1 && YAP1[0] == 1 && HNF1A[0] == 0 && HNF4A[0] == 1 && NFkB[0] == 0 && SNAI1[0] == 0 && ZEB1[0] == 0 && SNAI2[0] == 0 && TGFB[0] == 0 && SOX9[0] == 1 && NANOG[0] == 1 || BMI1[0] == 0 && E2F[0] == 0 && Bcatenin[0] == 1 && YAP1[0] == 1 && HNF1A[0] == 0 && HNF4A[0] == 1 && NFkB[0] == 0 && SNAI1[0] == 0 && ZEB1[0] == 0 && SNAI2[0] == 0 && TGFB[0] == 1 && SOX9[0] == 0 && NANOG[0] == 0 && SOX2[0] == 0 || BMI1[0] == 0 && E2F[0] == 0 && Bcatenin[0] == 1 && YAP1[0] == 1 && HNF1A[0] == 0 && HNF4A[0] == 1 && NFkB[0] == 0 && SNAI1[0] == 0 && ZEB1[0] == 0 && SNAI2[0] == 0 && TGFB[0] == 1 && SOX9[0] == 0 && NANOG[0] == 0 && SOX2[0] == 1 && p53[0] == 1 || BMI1[0] == 0 && E2F[0] == 0 && Bcatenin[0] == 1 && YAP1[0] == 1 && HNF1A[0] == 0 && HNF4A[0] == 1 && NFkB[0] == 0 && SNAI1[0] == 0 && ZEB1[0] == 0 && SNAI2[0] == 0 && TGFB[0] == 1 && SOX9[0] == 0 && NANOG[0] == 1 || BMI1[0] == 0 && E2F[0] == 0 && Bcatenin[0] == 1 && YAP1[0] == 1 && HNF1A[0] == 0 && HNF4A[0] == 1 && NFkB[0] == 0 && SNAI1[0] == 0 && ZEB1[0] == 0 && SNAI2[0] == 0 && TGFB[0] == 1 && SOX9[0] == 1 && NANOG[0] == 0 && SOX2[0] == 0 || BMI1[0] == 0 && E2F[0] == 0 && Bcatenin[0] == 1 && YAP1[0] == 1 && HNF1A[0] == 0 && HNF4A[0] == 1 && NFkB[0] == 0 && SNAI1[0] == 0 && ZEB1[0] == 0 && SNAI2[0] == 0 && TGFB[0] == 1 && SOX9[0] == 1 && NANOG[0] == 1 || BMI1[0] == 0 && E2F[0] == 0 && Bcatenin[0] == 1 && YAP1[0] == 1 && HNF1A[0] == 0 && HNF4A[0] == 1 && NFkB[0] == 0 && SNAI1[0] == 0 && ZEB1[0] == 0 && SNAI2[0] == 1 || BMI1[0] == 0 && E2F[0] == 0 && Bcatenin[0] == 1 && YAP1[0] == 1 && HNF1A[0] == 0 && HNF4A[0] == 1 && NFkB[0] == 0 && SNAI1[0] == 0 && ZEB1[0] == 1 || BMI1[0] == 0 && E2F[0] == 0 && Bcatenin[0] == 1 && YAP1[0] == 1 && HNF1A[0] == 0 && HNF4A[0] == 1 && NFkB[0] == 0 && SNAI1[0] == 1 || BMI1[0] == 0 && E2F[0] == 0 && Bcatenin[0] == 1 && YAP1[0] == 1 && HNF1A[0] == 0 && HNF4A[0] == 1 && NFkB[0] == 1 && SNAI1[0] == 0 && ZEB1[0] == 0 && SNAI2[0] == 0 && SOX9[0] == 0 || BMI1[0] == 0 && E2F[0] == 0 && Bcatenin[0] == 1 && YAP1[0] == 1 && HNF1A[0] == 0 && HNF4A[0] == 1 && NFkB[0] == 1 && SNAI1[0] == 0 && ZEB1[0] == 0 && SNAI2[0] == 0 && SOX9[0] == 1 && NANOG[0] == 0 && SOX2[0] == 0 || BMI1[0] == 0 && E2F[0] == 0 && Bcatenin[0] == 1 && YAP1[0] == 1 && HNF1A[0] == 0 && HNF4A[0] == 1 && NFkB[0] == 1 && SNAI1[0] == 0 && ZEB1[0] == 0 && SNAI2[0] == 0 && SOX9[0] == 1 && NANOG[0] == 0 && SOX2[0] == 1 && p53[0] == 0 || BMI1[0] == 0 && E2F[0] == 0 && Bcatenin[0] == 1 && YAP1[0] == 1 && HNF1A[0] == 0 && HNF4A[0] == 1 && NFkB[0] == 1 && SNAI1[0] == 0 && ZEB1[0] == 0 && SNAI2[0] == 0 && SOX9[0] == 1 && NANOG[0] == 1 || BMI1[0] == 0 && E2F[0] == 0 && Bcatenin[0] == 1 && YAP1[0] == 1 && HNF1A[0] == 0 && HNF4A[0] == 1 && NFkB[0] == 1 && SNAI1[0] == 0 && ZEB1[0] == 0 && SNAI2[0] == 1 || BMI1[0] == 0 && E2F[0] == 0 && Bcatenin[0] == 1 && YAP1[0] == 1 && HNF1A[0] == 0 && HNF4A[0] == 1 && NFkB[0] == 1 && SNAI1[0] == 0 && ZEB1[0] == 1 || BMI1[0] == 0 && E2F[0] == 0 && Bcatenin[0] == 1 && YAP1[0] == 1 && HNF1A[0] == 0 && HNF4A[0] == 1 && NFkB[0] == 1 && SNAI1[0] == 1 || BMI1[0] == 0 && E2F[0] == 1 && Bcatenin[0] == 0 && HNF1A[0] == 0 && HNF4A[0] == 0 && NFkB[0] == 0 && SNAI1[0] == 0 && SNAI2[0] == 1 || BMI1[0] == 0 && E2F[0] == 1 && Bcatenin[0] == 0 && HNF1A[0] == 0 && HNF4A[0] == 0 && NFkB[0] == 0 && SNAI1[0] == 1 || BMI1[0] == 0 && E2F[0] == 1 && Bcatenin[0] == 0 && HNF1A[0] == 0 && HNF4A[0] == 0 && NFkB[0] == 1 && SNAI1[0] == 0 && ZEB1[0] == 0 && SNAI2[0] == 0 && SOX9[0] == 0 || BMI1[0] == 0 && E2F[0] == 1 && Bcatenin[0] == 0 && HNF1A[0] == 0 && HNF4A[0] == 0 && NFkB[0] == 1 && SNAI1[0] == 0 && ZEB1[0] == 0 && SNAI2[0] == 0 && SOX9[0] == 1 && OCT4[0] == 0 || BMI1[0] == 0 && E2F[0] == 1 && Bcatenin[0] == 0 && HNF1A[0] == 0 && HNF4A[0] == 0 && NFkB[0] == 1 && SNAI1[0] == 0 && ZEB1[0] == 0 && SNAI2[0] == 0 && SOX9[0] == 1 && OCT4[0] == 1 && NANOG[0] == 0 && SOX2[0] == 0 || BMI1[0] == 0 && E2F[0] == 1 && Bcatenin[0] == 0 && HNF1A[0] == 0 && HNF4A[0] == 0 && NFkB[0] == 1 && SNAI1[0] == 0 && ZEB1[0] == 0 && SNAI2[0] == 0 && SOX9[0] == 1 && OCT4[0] == 1 && NANOG[0] == 0 && SOX2[0] == 1 && p53[0] == 0 || BMI1[0] == 0 && E2F[0] == 1 && Bcatenin[0] == 0 && HNF1A[0] == 0 && HNF4A[0] == 0 && NFkB[0] == 1 && SNAI1[0] == 0 && ZEB1[0] == 0 && SNAI2[0] == 0 && SOX9[0] == 1 && OCT4[0] == 1 && NANOG[0] == 1 || BMI1[0] == 0 && E2F[0] == 1 && Bcatenin[0] == 0 && HNF1A[0] == 0 && HNF4A[0] == 0 && NFkB[0] == 1 && SNAI1[0] == 0 && ZEB1[0] == 0 && SNAI2[0] == 1 || BMI1[0] == 0 && E2F[0] == 1 && Bcatenin[0] == 0 && HNF1A[0] == 0 && HNF4A[0] == 0 && NFkB[0] == 1 && SNAI1[0] == 0 && ZEB1[0] == 1 || BMI1[0] == 0 && E2F[0] == 1 && Bcatenin[0] == 0 && HNF1A[0] == 0 && HNF4A[0] == 0 && NFkB[0] == 1 && SNAI1[0] == 1 || BMI1[0] == 0 && E2F[0] == 1 && Bcatenin[0] == 0 && HNF1A[0] == 0 && HNF4A[0] == 1 && NFkB[0] == 0 && SNAI1[0] == 0 && SNAI2[0] == 1 || BMI1[0] == 0 && E2F[0] == 1 && Bcatenin[0] == 0 && HNF1A[0] == 0 && HNF4A[0] == 1 && NFkB[0] == 0 && SNAI1[0] == 1 || BMI1[0] == 0 && E2F[0] == 1 && Bcatenin[0] == 0 && HNF1A[0] == 0 && HNF4A[0] == 1 && NFkB[0] == 1 && SNAI1[0] == 0 && ZEB1[0] == 0 && SNAI2[0] == 0 && SOX9[0] == 0 || BMI1[0] == 0 && E2F[0] == 1 && Bcatenin[0] == 0 && HNF1A[0] == 0 && HNF4A[0] == 1 && NFkB[0] == 1 && SNAI1[0] == 0 && ZEB1[0] == 0 && SNAI2[0] == 0 && SOX9[0] == 1 && NANOG[0] == 0 && SOX2[0] == 0 || BMI1[0] == 0 && E2F[0] == 1 && Bcatenin[0] == 0 && HNF1A[0] == 0 && HNF4A[0] == 1 && NFkB[0] == 1 && SNAI1[0] == 0 && ZEB1[0] == 0 && SNAI2[0] == 0 && SOX9[0] == 1 && NANOG[0] == 0 && SOX2[0] == 1 && p53[0] == 0 || BMI1[0] == 0 && E2F[0] == 1 && Bcatenin[0] == 0 && HNF1A[0] == 0 && HNF4A[0] == 1 && NFkB[0] == 1 && SNAI1[0] == 0 && ZEB1[0] == 0 && SNAI2[0] == 0 && SOX9[0] == 1 && NANOG[0] == 1 || BMI1[0] == 0 && E2F[0] == 1 && Bcatenin[0] == 0 && HNF1A[0] == 0 && HNF4A[0] == 1 && NFkB[0] == 1 && SNAI1[0] == 0 && ZEB1[0] == 0 && SNAI2[0] == 1 || BMI1[0] == 0 && E2F[0] == 1 && Bcatenin[0] == 0 && HNF1A[0] == 0 && HNF4A[0] == 1 && NFkB[0] == 1 && SNAI1[0] == 0 && ZEB1[0] == 1 || BMI1[0] == 0 && E2F[0] == 1 && Bcatenin[0] == 0 && HNF1A[0] == 0 && HNF4A[0] == 1 && NFkB[0] == 1 && SNAI1[0] == 1 || BMI1[0] == 0 && E2F[0] == 1 && Bcatenin[0] == 1 && HNF1A[0] == 0 && HNF4A[0] == 0 && SNAI1[0] == 0 && ZEB1[0] == 0 && SNAI2[0] == 0 && SOX9[0] == 0 || BMI1[0] == 0 && E2F[0] == 1 && Bcatenin[0] == 1 && HNF1A[0] == 0 && HNF4A[0] == 0 && SNAI1[0] == 0 && ZEB1[0] == 0 && SNAI2[0] == 0 && SOX9[0] == 1 && OCT4[0] == 0 || BMI1[0] == 0 && E2F[0] == 1 && Bcatenin[0] == 1 && HNF1A[0] == 0 && HNF4A[0] == 0 && SNAI1[0] == 0 && ZEB1[0] == 0 && SNAI2[0] == 0 && SOX9[0] == 1 && OCT4[0] == 1 && NANOG[0] == 0 && SOX2[0] == 0 || BMI1[0] == 0 && E2F[0] == 1 && Bcatenin[0] == 1 && HNF1A[0] == 0 && HNF4A[0] == 0 && SNAI1[0] == 0 && ZEB1[0] == 0 && SNAI2[0] == 0 && SOX9[0] == 1 && OCT4[0] == 1 && NANOG[0] == 0 && SOX2[0] == 1 && p53[0] == 0 || BMI1[0] == 0 && E2F[0] == 1 && Bcatenin[0] == 1 && HNF1A[0] == 0 && HNF4A[0] == 0 && SNAI1[0] == 0 && ZEB1[0] == 0 && SNAI2[0] == 0 && SOX9[0] == 1 && OCT4[0] == 1 && NANOG[0] == 1 || BMI1[0] == 0 && E2F[0] == 1 && Bcatenin[0] == 1 && HNF1A[0] == 0 && HNF4A[0] == 0 && SNAI1[0] == 0 && ZEB1[0] == 0 && SNAI2[0] == 1 || BMI1[0] == 0 && E2F[0] == 1 && Bcatenin[0] == 1 && HNF1A[0] == 0 && HNF4A[0] == 0 && SNAI1[0] == 0 && ZEB1[0] == 1 || BMI1[0] == 0 && E2F[0] == 1 && Bcatenin[0] == 1 && HNF1A[0] == 0 && HNF4A[0] == 0 && SNAI1[0] == 1 || BMI1[0] == 0 && E2F[0] == 1 && Bcatenin[0] == 1 && HNF1A[0] == 0 && HNF4A[0] == 1 && NFkB[0] == 0 && SNAI1[0] == 0 && ZEB1[0] == 0 && SNAI2[0] == 0 && TGFB[0] == 0 && SOX9[0] == 0 || BMI1[0] == 0 && E2F[0] == 1 && Bcatenin[0] == 1 && HNF1A[0] == 0 && HNF4A[0] == 1 && NFkB[0] == 0 && SNAI1[0] == 0 && ZEB1[0] == 0 && SNAI2[0] == 0 && TGFB[0] == 0 && SOX9[0] == 1 && NANOG[0] == 0 && SOX2[0] == 0 || BMI1[0] == 0 && E2F[0] == 1 && Bcatenin[0] == 1 && HNF1A[0] == 0 && HNF4A[0] == 1 && NFkB[0] == 0 && SNAI1[0] == 0 && ZEB1[0] == 0 && SNAI2[0] == 0 && TGFB[0] == 0 && SOX9[0] == 1 && NANOG[0] == 0 && SOX2[0] == 1 && p53[0] == 0 || BMI1[0] == 0 && E2F[0] == 1 && Bcatenin[0] == 1 && HNF1A[0] == 0 && HNF4A[0] == 1 && NFkB[0] == 0 && SNAI1[0] == 0 && ZEB1[0] == 0 && SNAI2[0] == 0 && TGFB[0] == 0 && SOX9[0] == 1 && NANOG[0] == 1 || BMI1[0] == 0 && E2F[0] == 1 && Bcatenin[0] == 1 && HNF1A[0] == 0 && HNF4A[0] == 1 && NFkB[0] == 0 && SNAI1[0] == 0 && ZEB1[0] == 0 && SNAI2[0] == 0 && TGFB[0] == 1 && SOX9[0] == 0 && NANOG[0] == 0 && SOX2[0] == 0 || BMI1[0] == 0 && E2F[0] == 1 && Bcatenin[0] == 1 && HNF1A[0] == 0 && HNF4A[0] == 1 && NFkB[0] == 0 && SNAI1[0] == 0 && ZEB1[0] == 0 && SNAI2[0] == 0 && TGFB[0] == 1 && SOX9[0] == 0 && NANOG[0] == 0 && SOX2[0] == 1 && p53[0] == 1 || BMI1[0] == 0 && E2F[0] == 1 && Bcatenin[0] == 1 && HNF1A[0] == 0 && HNF4A[0] == 1 && NFkB[0] == 0 && SNAI1[0] == 0 && ZEB1[0] == 0 && SNAI2[0] == 0 && TGFB[0] == 1 && SOX9[0] == 0 && NANOG[0] == 1 || BMI1[0] == 0 && E2F[0] == 1 && Bcatenin[0] == 1 && HNF1A[0] == 0 && HNF4A[0] == 1 && NFkB[0] == 0 && SNAI1[0] == 0 && ZEB1[0] == 0 && SNAI2[0] == 0 && TGFB[0] == 1 && SOX9[0] == 1 && NANOG[0] == 0 && SOX2[0] == 0 || BMI1[0] == 0 && E2F[0] == 1 && Bcatenin[0] == 1 && HNF1A[0] == 0 && HNF4A[0] == 1 && NFkB[0] == 0 && SNAI1[0] == 0 && ZEB1[0] == 0 && SNAI2[0] == 0 && TGFB[0] == 1 && SOX9[0] == 1 && NANOG[0] == 1 || BMI1[0] == 0 && E2F[0] == 1 && Bcatenin[0] == 1 && HNF1A[0] == 0 && HNF4A[0] == 1 && NFkB[0] == 0 && SNAI1[0] == 0 && ZEB1[0] == 0 && SNAI2[0] == 1 || BMI1[0] == 0 && E2F[0] == 1 && Bcatenin[0] == 1 && HNF1A[0] == 0 && HNF4A[0] == 1 && NFkB[0] == 0 && SNAI1[0] == 0 && ZEB1[0] == 1 || BMI1[0] == 0 && E2F[0] == 1 && Bcatenin[0] == 1 && HNF1A[0] == 0 && HNF4A[0] == 1 && NFkB[0] == 0 && SNAI1[0] == 1 || BMI1[0] == 0 && E2F[0] == 1 && Bcatenin[0] == 1 && HNF1A[0] == 0 && HNF4A[0] == 1 && NFkB[0] == 1 && SNAI1[0] == 0 && ZEB1[0] == 0 && SNAI2[0] == 0 && SOX9[0] == 0 || BMI1[0] == 0 && E2F[0] == 1 && Bcatenin[0] == 1 && HNF1A[0] == 0 && HNF4A[0] == 1 && NFkB[0] == 1 && SNAI1[0] == 0 && ZEB1[0] == 0 && SNAI2[0] == 0 && SOX9[0] == 1 && NANOG[0] == 0 && SOX2[0] == 0 || BMI1[0] == 0 && E2F[0] == 1 && Bcatenin[0] == 1 && HNF1A[0] == 0 && HNF4A[0] == 1 && NFkB[0] == 1 && SNAI1[0] == 0 && ZEB1[0] == 0 && SNAI2[0] == 0 && SOX9[0] == 1 && NANOG[0] == 0 && SOX2[0] == 1 && p53[0] == 0 || BMI1[0] == 0 && E2F[0] == 1 && Bcatenin[0] == 1 && HNF1A[0] == 0 && HNF4A[0] == 1 && NFkB[0] == 1 && SNAI1[0] == 0 && ZEB1[0] == 0 && SNAI2[0] == 0 && SOX9[0] == 1 && NANOG[0] == 1 || BMI1[0] == 0 && E2F[0] == 1 && Bcatenin[0] == 1 && HNF1A[0] == 0 && HNF4A[0] == 1 && NFkB[0] == 1 && SNAI1[0] == 0 && ZEB1[0] == 0 && SNAI2[0] == 1 || BMI1[0] == 0 && E2F[0] == 1 && Bcatenin[0] == 1 && HNF1A[0] == 0 && HNF4A[0] == 1 && NFkB[0] == 1 && SNAI1[0] == 0 && ZEB1[0] == 1 || BMI1[0] == 0 && E2F[0] == 1 && Bcatenin[0] == 1 && HNF1A[0] == 0 && HNF4A[0] == 1 && NFkB[0] == 1 && SNAI1[0] == 1 || BMI1[0] == 1 && Bcatenin[0] == 0 && HNF1A[0] == 0 && NFkB[0] == 0 && SNAI1[0] == 0 && SNAI2[0] == 1 || BMI1[0] == 1 && Bcatenin[0] == 0 && HNF1A[0] == 0 && NFkB[0] == 0 && SNAI1[0] == 1 || BMI1[0] == 1 && Bcatenin[0] == 0 && HNF1A[0] == 0 && NFkB[0] == 1 || BMI1[0] == 1 && Bcatenin[0] == 1 && HNF1A[0] == 0) { ZEB1[0] = 1; }

else { ZEB1[0] = 0; }

//Logic rule for YAP1

if (Bcatenin[0] == 1 && HNF4A[0] == 0 && SOX2[0] == 1 && p53[0] == 0) { YAP1[0] = 1; }

else { YAP1[0] = 0; }

//Logic rule for TGFB

if (E2F[0] == 0 && Bcatenin[0] == 0 && YAP1[0] == 1 && HNF4A[0] == 0 && NFkB[0] == 0 && SNAI1[0] == 0 && ZEB1[0] == 0 && SOX9[0] == 0 && NANOG[0] == 0 && SOX2[0] == 0 && p53[0] == 0 || E2F[0] == 0 && Bcatenin[0] == 0 && YAP1[0] == 1 && HNF4A[0] == 0 && NFkB[0] == 0 && SNAI1[0] == 0 && ZEB1[0] == 0 && SOX9[0] == 1 && NANOG[0] == 0 && SOX2[0] == 0 || E2F[0] == 0 && Bcatenin[0] == 0 && YAP1[0] == 1 && HNF4A[0] == 0 && NFkB[0] == 0 && SNAI1[0] == 0 && ZEB1[0] == 0 && SOX9[0] == 1 && NANOG[0] == 1 && p53[0] == 1 || E2F[0] == 0 && Bcatenin[0] == 0 && YAP1[0] == 1 && HNF4A[0] == 0 && NFkB[0] == 0 && SNAI1[0] == 0 && ZEB1[0] == 1 && NANOG[0] == 0 && SOX2[0] == 0 && p53[0] == 0 || E2F[0] == 0 && Bcatenin[0] == 0 && YAP1[0] == 1 && HNF4A[0] == 0 && NFkB[0] == 0 && SNAI1[0] == 1 && NANOG[0] == 0 && SOX2[0] == 0 || E2F[0] == 0 && Bcatenin[0] == 0 && YAP1[0] == 1 && HNF4A[0] == 0 && NFkB[0] == 0 && SNAI1[0] == 1 && NANOG[0] == 1 || E2F[0] == 0 && Bcatenin[0] == 0 && YAP1[0] == 1 && HNF4A[0] == 0 && NFkB[0] == 1 && SNAI1[0] == 0 && ZEB1[0] == 0 && SOX9[0] == 0 && NANOG[0] == 0 && p53[0] == 0 || E2F[0] == 0 && Bcatenin[0] == 0 && YAP1[0] == 1 && HNF4A[0] == 0 && NFkB[0] == 1 && SNAI1[0] == 0 && ZEB1[0] == 0 && SOX9[0] == 1 && NANOG[0] == 0 && SOX2[0] == 0 || E2F[0] == 0 && Bcatenin[0] == 0 && YAP1[0] == 1 && HNF4A[0] == 0 && NFkB[0] == 1 && SNAI1[0] == 0 && ZEB1[0] == 0 && SOX9[0] == 1 && NANOG[0] == 0 && SOX2[0] == 1 && p53[0] == 0 || E2F[0] == 0 && Bcatenin[0] == 0 && YAP1[0] == 1 && HNF4A[0] == 0 && NFkB[0] == 1 && SNAI1[0] == 0 && ZEB1[0] == 0 && SOX9[0] == 1 && NANOG[0] == 1 && p53[0] == 1 || E2F[0] == 0 && Bcatenin[0] == 0 && YAP1[0] == 1 && HNF4A[0] == 0 && NFkB[0] == 1 && SNAI1[0] == 0 && ZEB1[0] == 1 && NANOG[0] == 0 && p53[0] == 0 || E2F[0] == 0 && Bcatenin[0] == 0 && YAP1[0] == 1 && HNF4A[0] == 0 && NFkB[0] == 1 && SNAI1[0] == 1 && NANOG[0] == 0 && SOX2[0] == 0 || E2F[0] == 0 && Bcatenin[0] == 0 && YAP1[0] == 1 && HNF4A[0] == 0 && NFkB[0] == 1 && SNAI1[0] == 1 && NANOG[0] == 0 && SOX2[0] == 1 && p53[0] == 0 || E2F[0] == 0 && Bcatenin[0] == 0 && YAP1[0] == 1 && HNF4A[0] == 0 && NFkB[0] == 1 && SNAI1[0] == 1 && NANOG[0] == 1 || E2F[0] == 0 && Bcatenin[0] == 0 && YAP1[0] == 1 && HNF4A[0] == 1 && NFkB[0] == 0 && SNAI1[0] == 0 && ZEB1[0] == 0 && SOX9[0] == 0 && NANOG[0] == 0 && SOX2[0] == 0 && p53[0] == 0 || E2F[0] == 0 && Bcatenin[0] == 0 && YAP1[0] == 1 && HNF4A[0] == 1 && NFkB[0] == 0 && SNAI1[0] == 0 && ZEB1[0] == 0 && SOX9[0] == 1 && NANOG[0] == 0 && SOX2[0] == 0 || E2F[0] == 0 && Bcatenin[0] == 0 && YAP1[0] == 1 && HNF4A[0] == 1 && NFkB[0] == 0 && SNAI1[0] == 0 && ZEB1[0] == 0 && SOX9[0] == 1 && NANOG[0] == 1 || E2F[0] == 0 && Bcatenin[0] == 0 && YAP1[0] == 1 && HNF4A[0] == 1 && NFkB[0] == 0 && SNAI1[0] == 0 && ZEB1[0] == 1 && NANOG[0] == 0 && SOX2[0] == 0 && p53[0] == 0 || E2F[0] == 0 && Bcatenin[0] == 0 && YAP1[0] == 1 && HNF4A[0] == 1 && NFkB[0] == 0 && SNAI1[0] == 1 && NANOG[0] == 0 && SOX2[0] == 0 || E2F[0] == 0 && Bcatenin[0] == 0 && YAP1[0] == 1 && HNF4A[0] == 1 && NFkB[0] == 0 && SNAI1[0] == 1 && NANOG[0] == 1 || E2F[0] == 0 && Bcatenin[0] == 0 && YAP1[0] == 1 && HNF4A[0] == 1 && NFkB[0] == 1 && SNAI1[0] == 0 && ZEB1[0] == 0 && SOX9[0] == 0 && NANOG[0] == 0 && p53[0] == 0 || E2F[0] == 0 && Bcatenin[0] == 0 && YAP1[0] == 1 && HNF4A[0] == 1 && NFkB[0] == 1 && SNAI1[0] == 0 && ZEB1[0] == 0 && SOX9[0] == 1 && NANOG[0] == 0 && SOX2[0] == 0 || E2F[0] == 0 && Bcatenin[0] == 0 && YAP1[0] == 1 && HNF4A[0] == 1 && NFkB[0] == 1 && SNAI1[0] == 0 && ZEB1[0] == 0 && SOX9[0] == 1 && NANOG[0] == 0 && SOX2[0] == 1 && p53[0] == 0 || E2F[0] == 0 && Bcatenin[0] == 0 && YAP1[0] == 1 && HNF4A[0] == 1 && NFkB[0] == 1 && SNAI1[0] == 0 && ZEB1[0] == 0 && SOX9[0] == 1 && NANOG[0] == 1 || E2F[0] == 0 && Bcatenin[0] == 0 && YAP1[0] == 1 && HNF4A[0] == 1 && NFkB[0] == 1 && SNAI1[0] == 0 && ZEB1[0] == 1 && NANOG[0] == 0 && p53[0] == 0 || E2F[0] == 0 && Bcatenin[0] == 0 && YAP1[0] == 1 && HNF4A[0] == 1 && NFkB[0] == 1 && SNAI1[0] == 1 && NANOG[0] == 0 && SOX2[0] == 0 || E2F[0] == 0 && Bcatenin[0] == 0 && YAP1[0] == 1 && HNF4A[0] == 1 && NFkB[0] == 1 && SNAI1[0] == 1 && NANOG[0] == 0 && SOX2[0] == 1 && p53[0] == 0 || E2F[0] == 0 && Bcatenin[0] == 0 && YAP1[0] == 1 && HNF4A[0] == 1 && NFkB[0] == 1 && SNAI1[0] == 1 && NANOG[0] == 1 || E2F[0] == 0 && Bcatenin[0] == 1 && YAP1[0] == 1 && HNF4A[0] == 0 && SNAI1[0] == 0 && ZEB1[0] == 0 && SOX9[0] == 0 && NANOG[0] == 0 && p53[0] == 0 || E2F[0] == 0 && Bcatenin[0] == 1 && YAP1[0] == 1 && HNF4A[0] == 0 && SNAI1[0] == 0 && ZEB1[0] == 0 && SOX9[0] == 1 && NANOG[0] == 0 && SOX2[0] == 0 || E2F[0] == 0 && Bcatenin[0] == 1 && YAP1[0] == 1 && HNF4A[0] == 0 && SNAI1[0] == 0 && ZEB1[0] == 0 && SOX9[0] == 1 && NANOG[0] == 0 && SOX2[0] == 1 && p53[0] == 0 || E2F[0] == 0 && Bcatenin[0] == 1 && YAP1[0] == 1 && HNF4A[0] == 0 && SNAI1[0] == 0 && ZEB1[0] == 0 && SOX9[0] == 1 && NANOG[0] == 1 && p53[0] == 1 || E2F[0] == 0 && Bcatenin[0] == 1 && YAP1[0] == 1 && HNF4A[0] == 0 && SNAI1[0] == 0 && ZEB1[0] == 1 && NANOG[0] == 0 && p53[0] == 0 || E2F[0] == 0 && Bcatenin[0] == 1 && YAP1[0] == 1 && HNF4A[0] == 0 && SNAI1[0] == 1 && NANOG[0] == 0 && SOX2[0] == 0 || E2F[0] == 0 && Bcatenin[0] == 1 && YAP1[0] == 1 && HNF4A[0] == 0 && SNAI1[0] == 1 && NANOG[0] == 0 && SOX2[0] == 1 && p53[0] == 0 || E2F[0] == 0 && Bcatenin[0] == 1 && YAP1[0] == 1 && HNF4A[0] == 0 && SNAI1[0] == 1 && NANOG[0] == 1 || E2F[0] == 0 && Bcatenin[0] == 1 && YAP1[0] == 1 && HNF4A[0] == 1 && NFkB[0] == 0 && SNAI1[0] == 0 && ZEB1[0] == 0 && TGFB[0] == 0 && SOX9[0] == 0 && NANOG[0] == 0 && p53[0] == 0 || E2F[0] == 0 && Bcatenin[0] == 1 && YAP1[0] == 1 && HNF4A[0] == 1 && NFkB[0] == 0 && SNAI1[0] == 0 && ZEB1[0] == 0 && TGFB[0] == 0 && SOX9[0] == 1 && NANOG[0] == 0 && SOX2[0] == 0 || E2F[0] == 0 && Bcatenin[0] == 1 && YAP1[0] == 1 && HNF4A[0] == 1 && NFkB[0] == 0 && SNAI1[0] == 0 && ZEB1[0] == 0 && TGFB[0] == 0 && SOX9[0] == 1 && NANOG[0] == 0 && SOX2[0] == 1 && p53[0] == 0 || E2F[0] == 0 && Bcatenin[0] == 1 && YAP1[0] == 1 && HNF4A[0] == 1 && NFkB[0] == 0 && SNAI1[0] == 0 && ZEB1[0] == 0 && TGFB[0] == 0 && SOX9[0] == 1 && NANOG[0] == 1 || E2F[0] == 0 && Bcatenin[0] == 1 && YAP1[0] == 1 && HNF4A[0] == 1 && NFkB[0] == 0 && SNAI1[0] == 0 && ZEB1[0] == 0 && TGFB[0] == 1 && SOX9[0] == 0 && NANOG[0] == 0 && SOX2[0] == 0 && p53[0] == 0 || E2F[0] == 0 && Bcatenin[0] == 1 && YAP1[0] == 1 && HNF4A[0] == 1 && NFkB[0] == 0 && SNAI1[0] == 0 && ZEB1[0] == 0 && TGFB[0] == 1 && SOX9[0] == 1 && NANOG[0] == 0 && SOX2[0] == 0 || E2F[0] == 0 && Bcatenin[0] == 1 && YAP1[0] == 1 && HNF4A[0] == 1 && NFkB[0] == 0 && SNAI1[0] == 0 && ZEB1[0] == 0 && TGFB[0] == 1 && SOX9[0] == 1 && NANOG[0] == 1 || E2F[0] == 0 && Bcatenin[0] == 1 && YAP1[0] == 1 && HNF4A[0] == 1 && NFkB[0] == 0 && SNAI1[0] == 0 && ZEB1[0] == 1 && NANOG[0] == 0 && p53[0] == 0 || E2F[0] == 0 && Bcatenin[0] == 1 && YAP1[0] == 1 && HNF4A[0] == 1 && NFkB[0] == 0 && SNAI1[0] == 1 && NANOG[0] == 0 && SOX2[0] == 0 || E2F[0] == 0 && Bcatenin[0] == 1 && YAP1[0] == 1 && HNF4A[0] == 1 && NFkB[0] == 0 && SNAI1[0] == 1 && NANOG[0] == 0 && SOX2[0] == 1 && p53[0] == 0 || E2F[0] == 0 && Bcatenin[0] == 1 && YAP1[0] == 1 && HNF4A[0] == 1 && NFkB[0] == 0 && SNAI1[0] == 1 && NANOG[0] == 1 || E2F[0] == 0 && Bcatenin[0] == 1 && YAP1[0] == 1 && HNF4A[0] == 1 && NFkB[0] == 1 && SNAI1[0] == 0 && ZEB1[0] == 0 && SOX9[0] == 0 && NANOG[0] == 0 && p53[0] == 0 || E2F[0] == 0 && Bcatenin[0] == 1 && YAP1[0] == 1 && HNF4A[0] == 1 && NFkB[0] == 1 && SNAI1[0] == 0 && ZEB1[0] == 0 && SOX9[0] == 1 && NANOG[0] == 0 && SOX2[0] == 0 || E2F[0] == 0 && Bcatenin[0] == 1 && YAP1[0] == 1 && HNF4A[0] == 1 && NFkB[0] == 1 && SNAI1[0] == 0 && ZEB1[0] == 0 && SOX9[0] == 1 && NANOG[0] == 0 && SOX2[0] == 1 && p53[0] == 0 || E2F[0] == 0 && Bcatenin[0] == 1 && YAP1[0] == 1 && HNF4A[0] == 1 && NFkB[0] == 1 && SNAI1[0] == 0 && ZEB1[0] == 0 && SOX9[0] == 1 && NANOG[0] == 1 || E2F[0] == 0 && Bcatenin[0] == 1 && YAP1[0] == 1 && HNF4A[0] == 1 && NFkB[0] == 1 && SNAI1[0] == 0 && ZEB1[0] == 1 && NANOG[0] == 0 && p53[0] == 0 || E2F[0] == 0 && Bcatenin[0] == 1 && YAP1[0] == 1 && HNF4A[0] == 1 && NFkB[0] == 1 && SNAI1[0] == 1 && NANOG[0] == 0 && SOX2[0] == 0 || E2F[0] == 0 && Bcatenin[0] == 1 && YAP1[0] == 1 && HNF4A[0] == 1 && NFkB[0] == 1 && SNAI1[0] == 1 && NANOG[0] == 0 && SOX2[0] == 1 && p53[0] == 0 || E2F[0] == 0 && Bcatenin[0] == 1 && YAP1[0] == 1 && HNF4A[0] == 1 && NFkB[0] == 1 && SNAI1[0] == 1 && NANOG[0] == 1 || E2F[0] == 1 && YAP1[0] == 1 && HNF4A[0] == 0 && SNAI1[0] == 0 && ZEB1[0] == 0 && SOX9[0] == 0 && NANOG[0] == 0 && p53[0] == 0 || E2F[0] == 1 && YAP1[0] == 1 && HNF4A[0] == 0 && SNAI1[0] == 0 && ZEB1[0] == 0 && SOX9[0] == 1 && NANOG[0] == 0 && SOX2[0] == 0 || E2F[0] == 1 && YAP1[0] == 1 && HNF4A[0] == 0 && SNAI1[0] == 0 && ZEB1[0] == 0 && SOX9[0] == 1 && NANOG[0] == 0 && SOX2[0] == 1 && p53[0] == 0 || E2F[0] == 1 && YAP1[0] == 1 && HNF4A[0] == 0 && SNAI1[0] == 0 && ZEB1[0] == 0 && SOX9[0] == 1 && NANOG[0] == 1 && p53[0] == 1 || E2F[0] == 1 && YAP1[0] == 1 && HNF4A[0] == 0 && SNAI1[0] == 0 && ZEB1[0] == 1 && NANOG[0] == 0 && p53[0] == 0 || E2F[0] == 1 && YAP1[0] == 1 && HNF4A[0] == 0 && SNAI1[0] == 1 && NANOG[0] == 0 && SOX2[0] == 0 || E2F[0] == 1 && YAP1[0] == 1 && HNF4A[0] == 0 && SNAI1[0] == 1 && NANOG[0] == 0 && SOX2[0] == 1 && p53[0] == 0 || E2F[0] == 1 && YAP1[0] == 1 && HNF4A[0] == 0 && SNAI1[0] == 1 && NANOG[0] == 1 || E2F[0] == 1 && YAP1[0] == 1 && HNF4A[0] == 1 && NFkB[0] == 0 && SNAI1[0] == 0 && ZEB1[0] == 0 && TGFB[0] == 0 && SOX9[0] == 0 && NANOG[0] == 0 && p53[0] == 0 || E2F[0] == 1 && YAP1[0] == 1 && HNF4A[0] == 1 && NFkB[0] == 0 && SNAI1[0] == 0 && ZEB1[0] == 0 && TGFB[0] == 0 && SOX9[0] == 1 && NANOG[0] == 0 && SOX2[0] == 0 || E2F[0] == 1 && YAP1[0] == 1 && HNF4A[0] == 1 && NFkB[0] == 0 && SNAI1[0] == 0 && ZEB1[0] == 0 && TGFB[0] == 0 && SOX9[0] == 1 && NANOG[0] == 0 && SOX2[0] == 1 && p53[0] == 0 || E2F[0] == 1 && YAP1[0] == 1 && HNF4A[0] == 1 && NFkB[0] == 0 && SNAI1[0] == 0 && ZEB1[0] == 0 && TGFB[0] == 0 && SOX9[0] == 1 && NANOG[0] == 1 || E2F[0] == 1 && YAP1[0] == 1 && HNF4A[0] == 1 && NFkB[0] == 0 && SNAI1[0] == 0 && ZEB1[0] == 0 && TGFB[0] == 1 && SOX9[0] == 0 && NANOG[0] == 0 && SOX2[0] == 0 && p53[0] == 0 || E2F[0] == 1 && YAP1[0] == 1 && HNF4A[0] == 1 && NFkB[0] == 0 && SNAI1[0] == 0 && ZEB1[0] == 0 && TGFB[0] == 1 && SOX9[0] == 1 && NANOG[0] == 0 && SOX2[0] == 0 || E2F[0] == 1 && YAP1[0] == 1 && HNF4A[0] == 1 && NFkB[0] == 0 && SNAI1[0] == 0 && ZEB1[0] == 0 && TGFB[0] == 1 && SOX9[0] == 1 && NANOG[0] == 1 || E2F[0] == 1 && YAP1[0] == 1 && HNF4A[0] == 1 && NFkB[0] == 0 && SNAI1[0] == 0 && ZEB1[0] == 1 && NANOG[0] == 0 && p53[0] == 0 || E2F[0] == 1 && YAP1[0] == 1 && HNF4A[0] == 1 && NFkB[0] == 0 && SNAI1[0] == 1 && NANOG[0] == 0 && SOX2[0] == 0 || E2F[0] == 1 && YAP1[0] == 1 && HNF4A[0] == 1 && NFkB[0] == 0 && SNAI1[0] == 1 && NANOG[0] == 0 && SOX2[0] == 1 && p53[0] == 0 || E2F[0] == 1 && YAP1[0] == 1 && HNF4A[0] == 1 && NFkB[0] == 0 && SNAI1[0] == 1 && NANOG[0] == 1 || E2F[0] == 1 && YAP1[0] == 1 && HNF4A[0] == 1 && NFkB[0] == 1 && SNAI1[0] == 0 && ZEB1[0] == 0 && SOX9[0] == 0 && NANOG[0] == 0 && p53[0] == 0 || E2F[0] == 1 && YAP1[0] == 1 && HNF4A[0] == 1 && NFkB[0] == 1 && SNAI1[0] == 0 && ZEB1[0] == 0 && SOX9[0] == 1 && NANOG[0] == 0 && SOX2[0] == 0 || E2F[0] == 1 && YAP1[0] == 1 && HNF4A[0] == 1 && NFkB[0] == 1 && SNAI1[0] == 0 && ZEB1[0] == 0 && SOX9[0] == 1 && NANOG[0] == 0 && SOX2[0] == 1 && p53[0] == 0 || E2F[0] == 1 && YAP1[0] == 1 && HNF4A[0] == 1 && NFkB[0] == 1 && SNAI1[0] == 0 && ZEB1[0] == 0 && SOX9[0] == 1 && NANOG[0] == 1 || E2F[0] == 1 && YAP1[0] == 1 && HNF4A[0] == 1 && NFkB[0] == 1 && SNAI1[0] == 0 && ZEB1[0] == 1 && NANOG[0] == 0 && p53[0] == 0 || E2F[0] == 1 && YAP1[0] == 1 && HNF4A[0] == 1 && NFkB[0] == 1 && SNAI1[0] == 1 && NANOG[0] == 0 && SOX2[0] == 0 || E2F[0] == 1 && YAP1[0] == 1 && HNF4A[0] == 1 && NFkB[0] == 1 && SNAI1[0] == 1 && NANOG[0] == 0 && SOX2[0] == 1 && p53[0] == 0 || E2F[0] == 1 && YAP1[0] == 1 && HNF4A[0] == 1 && NFkB[0] == 1 && SNAI1[0] == 1 && NANOG[0] == 1) { TGFB[0] = 1; }

else { TGFB[0] = 0; }

//Logic rule for OCT4

if (YAP1[0] == 0 && NANOG[0] == 1 && SOX2[0] == 1 && p53[0] == 0 && RB[0] == 0 || YAP1[0] == 1 && HNF4A[0] == 0 && SNAI1[0] == 0 && OCT4[0] == 0 && NANOG[0] == 1 && p53[0] == 0 || YAP1[0] == 1 && HNF4A[0] == 0 && SNAI1[0] == 0 && OCT4[0] == 1 && NANOG[0] == 0 && SOX2[0] == 1 && p53[0] == 0 || YAP1[0] == 1 && HNF4A[0] == 0 && SNAI1[0] == 0 && OCT4[0] == 1 && NANOG[0] == 1 && p53[0] == 0 || YAP1[0] == 1 && HNF4A[0] == 0 && SNAI1[0] == 1 && OCT4[0] == 0 && NANOG[0] == 1 && SOX2[0] == 1 && p53[0] == 0 && RB[0] == 0 || YAP1[0] == 1 && HNF4A[0] == 0 && SNAI1[0] == 1 && OCT4[0] == 1 && NANOG[0] == 0 && SOX2[0] == 1 && p53[0] == 0 || YAP1[0] == 1 && HNF4A[0] == 0 && SNAI1[0] == 1 && OCT4[0] == 1 && NANOG[0] == 1 && p53[0] == 0 || YAP1[0] == 1 && HNF4A[0] == 1 && SNAI1[0] == 0 && ZEB1[0] == 0 && SOX9[0] == 0 && OCT4[0] == 0 && NANOG[0] == 1 && p53[0] == 0 || YAP1[0] == 1 && HNF4A[0] == 1 && SNAI1[0] == 0 && ZEB1[0] == 0 && SOX9[0] == 0 && OCT4[0] == 1 && NANOG[0] == 0 && SOX2[0] == 1 && p53[0] == 0 || YAP1[0] == 1 && HNF4A[0] == 1 && SNAI1[0] == 0 && ZEB1[0] == 0 && SOX9[0] == 0 && OCT4[0] == 1 && NANOG[0] == 1 && p53[0] == 0 || YAP1[0] == 1 && HNF4A[0] == 1 && SNAI1[0] == 0 && ZEB1[0] == 0 && SOX9[0] == 1 && OCT4[0] == 0 && NANOG[0] == 1 && SOX2[0] == 1 && p53[0] == 0 && RB[0] == 0 || YAP1[0] == 1 && HNF4A[0] == 1 && SNAI1[0] == 0 && ZEB1[0] == 0 && SOX9[0] == 1 && OCT4[0] == 1 && NANOG[0] == 0 && SOX2[0] == 1 && p53[0] == 0 || YAP1[0] == 1 && HNF4A[0] == 1 && SNAI1[0] == 0 && ZEB1[0] == 0 && SOX9[0] == 1 && OCT4[0] == 1 && NANOG[0] == 1 && p53[0] == 0 || YAP1[0] == 1 && HNF4A[0] == 1 && SNAI1[0] == 0 && ZEB1[0] == 1 && OCT4[0] == 0 && NANOG[0] == 1 && p53[0] == 0 || YAP1[0] == 1 && HNF4A[0] == 1 && SNAI1[0] == 0 && ZEB1[0] == 1 && OCT4[0] == 1 && NANOG[0] == 0 && SOX2[0] == 1 && p53[0] == 0 || YAP1[0] == 1 && HNF4A[0] == 1 && SNAI1[0] == 0 && ZEB1[0] == 1 && OCT4[0] == 1 && NANOG[0] == 1 && p53[0] == 0 || YAP1[0] == 1 && HNF4A[0] == 1 && SNAI1[0] == 1 && OCT4[0] == 0 && NANOG[0] == 1 && SOX2[0] == 1 && p53[0] == 0 && RB[0] == 0 || YAP1[0] == 1 && HNF4A[0] == 1 && SNAI1[0] == 1 && OCT4[0] == 1 && NANOG[0] == 0 && SOX2[0] == 1 && p53[0] == 0 || YAP1[0] == 1 && HNF4A[0] == 1 && SNAI1[0] == 1 && OCT4[0] == 1 && NANOG[0] == 1 && p53[0] == 0) { OCT4[0] = 1; }

else { OCT4[0] = 0; }

//Logic rule for SOX2

if (BMI1[0] == 0 && E2F[0] == 0 && Bcatenin[0] == 0 && YAP1[0] == 0 && OCT4[0] == 1 && NANOG[0] == 1 && SOX2[0] == 1 && p53[0] == 0 && p21[0] == 0 && RB[0] == 0 || BMI1[0] == 0 && E2F[0] == 0 && Bcatenin[0] == 0 && YAP1[0] == 1 && HNF4A[0] == 0 && OCT4[0] == 0 && NANOG[0] == 1 && p53[0] == 0 && p21[0] == 0 && RB[0] == 0 || BMI1[0] == 0 && E2F[0] == 0 && Bcatenin[0] == 0 && YAP1[0] == 1 && HNF4A[0] == 0 && OCT4[0] == 1 && p53[0] == 0 && p21[0] == 0 && RB[0] == 0 || BMI1[0] == 0 && E2F[0] == 0 && Bcatenin[0] == 0 && YAP1[0] == 1 && HNF4A[0] == 1 && NFkB[0] == 0 && SNAI1[0] == 0 && ZEB1[0] == 0 && OCT4[0] == 1 && NANOG[0] == 1 && SOX2[0] == 1 && p53[0] == 0 && p21[0] == 0 && RB[0] == 0 || BMI1[0] == 0 && E2F[0] == 0 && Bcatenin[0] == 0 && YAP1[0] == 1 && HNF4A[0] == 1 && NFkB[0] == 0 && SNAI1[0] == 0 && ZEB1[0] == 1 && OCT4[0] == 0 && NANOG[0] == 1 && p53[0] == 0 && p21[0] == 0 && RB[0] == 0 || BMI1[0] == 0 && E2F[0] == 0 && Bcatenin[0] == 0 && YAP1[0] == 1 && HNF4A[0] == 1 && NFkB[0] == 0 && SNAI1[0] == 0 && ZEB1[0] == 1 && OCT4[0] == 1 && p53[0] == 0 && p21[0] == 0 && RB[0] == 0 || BMI1[0] == 0 && E2F[0] == 0 && Bcatenin[0] == 0 && YAP1[0] == 1 && HNF4A[0] == 1 && NFkB[0] == 0 && SNAI1[0] == 1 && OCT4[0] == 0 && NANOG[0] == 1 && p53[0] == 0 && p21[0] == 0 && RB[0] == 0 || BMI1[0] == 0 && E2F[0] == 0 && Bcatenin[0] == 0 && YAP1[0] == 1 && HNF4A[0] == 1 && NFkB[0] == 0 && SNAI1[0] == 1 && OCT4[0] == 1 && p53[0] == 0 && p21[0] == 0 && RB[0] == 0 || BMI1[0] == 0 && E2F[0] == 0 && Bcatenin[0] == 0 && YAP1[0] == 1 && HNF4A[0] == 1 && NFkB[0] == 1 && SNAI1[0] == 0 && ZEB1[0] == 0 && OCT4[0] == 0 && NANOG[0] == 1 && SOX2[0] == 1 && p53[0] == 0 && p21[0] == 0 && RB[0] == 0 || BMI1[0] == 0 && E2F[0] == 0 && Bcatenin[0] == 0 && YAP1[0] == 1 && HNF4A[0] == 1 && NFkB[0] == 1 && SNAI1[0] == 0 && ZEB1[0] == 0 && OCT4[0] == 1 && NANOG[0] == 1 && p53[0] == 0 && p21[0] == 0 && RB[0] == 0 || BMI1[0] == 0 && E2F[0] == 0 && Bcatenin[0] == 0 && YAP1[0] == 1 && HNF4A[0] == 1 && NFkB[0] == 1 && SNAI1[0] == 0 && ZEB1[0] == 1 && OCT4[0] == 0 && NANOG[0] == 1 && p53[0] == 0 && p21[0] == 0 && RB[0] == 0 || BMI1[0] == 0 && E2F[0] == 0 && Bcatenin[0] == 0 && YAP1[0] == 1 && HNF4A[0] == 1 && NFkB[0] == 1 && SNAI1[0] == 0 && ZEB1[0] == 1 && OCT4[0] == 1 && p53[0] == 0 && p21[0] == 0 && RB[0] == 0 || BMI1[0] == 0 && E2F[0] == 0 && Bcatenin[0] == 0 && YAP1[0] == 1 && HNF4A[0] == 1 && NFkB[0] == 1 && SNAI1[0] == 1 && OCT4[0] == 0 && NANOG[0] == 1 && p53[0] == 0 && p21[0] == 0 && RB[0] == 0 || BMI1[0] == 0 && E2F[0] == 0 && Bcatenin[0] == 0 && YAP1[0] == 1 && HNF4A[0] == 1 && NFkB[0] == 1 && SNAI1[0] == 1 && OCT4[0] == 1 && p53[0] == 0 && p21[0] == 0 && RB[0] == 0 || BMI1[0] == 0 && E2F[0] == 0 && Bcatenin[0] == 1 && YAP1[0] == 0 && HNF4A[0] == 0 && OCT4[0] == 0 && NANOG[0] == 1 && p53[0] == 0 && p21[0] == 0 && RB[0] == 0 || BMI1[0] == 0 && E2F[0] == 0 && Bcatenin[0] == 1 && YAP1[0] == 0 && HNF4A[0] == 0 && OCT4[0] == 1 && NANOG[0] == 0 && p53[0] == 0 && p21[0] == 0 && RB[0] == 0 || BMI1[0] == 0 && E2F[0] == 0 && Bcatenin[0] == 1 && YAP1[0] == 0 && HNF4A[0] == 0 && OCT4[0] == 1 && NANOG[0] == 1 && p53[0] == 0 && p21[0] == 0 || BMI1[0] == 0 && E2F[0] == 0 && Bcatenin[0] == 1 && YAP1[0] == 0 && HNF4A[0] == 1 && SNAI1[0] == 0 && ZEB1[0] == 0 && TGFB[0] == 0 && OCT4[0] == 0 && NANOG[0] == 1 && SOX2[0] == 1 && p53[0] == 0 && p21[0] == 0 && RB[0] == 0 || BMI1[0] == 0 && E2F[0] == 0 && Bcatenin[0] == 1 && YAP1[0] == 0 && HNF4A[0] == 1 && SNAI1[0] == 0 && ZEB1[0] == 0 && TGFB[0] == 0 && OCT4[0] == 1 && NANOG[0] == 1 && p53[0] == 0 && p21[0] == 0 || BMI1[0] == 0 && E2F[0] == 0 && Bcatenin[0] == 1 && YAP1[0] == 0 && HNF4A[0] == 1 && SNAI1[0] == 0 && ZEB1[0] == 0 && TGFB[0] == 1 && OCT4[0] == 1 && NANOG[0] == 1 && p53[0] == 0 && p21[0] == 0 || BMI1[0] == 0 && E2F[0] == 0 && Bcatenin[0] == 1 && YAP1[0] == 0 && HNF4A[0] == 1 && SNAI1[0] == 0 && ZEB1[0] == 1 && OCT4[0] == 0 && NANOG[0] == 1 && p53[0] == 0 && p21[0] == 0 && RB[0] == 0 || BMI1[0] == 0 && E2F[0] == 0 && Bcatenin[0] == 1 && YAP1[0] == 0 && HNF4A[0] == 1 && SNAI1[0] == 0 && ZEB1[0] == 1 && OCT4[0] == 1 && NANOG[0] == 0 && p53[0] == 0 && p21[0] == 0 && RB[0] == 0 || BMI1[0] == 0 && E2F[0] == 0 && Bcatenin[0] == 1 && YAP1[0] == 0 && HNF4A[0] == 1 && SNAI1[0] == 0 && ZEB1[0] == 1 && OCT4[0] == 1 && NANOG[0] == 1 && p53[0] == 0 && p21[0] == 0 || BMI1[0] == 0 && E2F[0] == 0 && Bcatenin[0] == 1 && YAP1[0] == 0 && HNF4A[0] == 1 && SNAI1[0] == 1 && OCT4[0] == 0 && NANOG[0] == 1 && p53[0] == 0 && p21[0] == 0 && RB[0] == 0 || BMI1[0] == 0 && E2F[0] == 0 && Bcatenin[0] == 1 && YAP1[0] == 0 && HNF4A[0] == 1 && SNAI1[0] == 1 && OCT4[0] == 1 && NANOG[0] == 0 && p53[0] == 0 && p21[0] == 0 && RB[0] == 0 || BMI1[0] == 0 && E2F[0] == 0 && Bcatenin[0] == 1 && YAP1[0] == 0 && HNF4A[0] == 1 && SNAI1[0] == 1 && OCT4[0] == 1 && NANOG[0] == 1 && p53[0] == 0 && p21[0] == 0 || BMI1[0] == 0 && E2F[0] == 0 && Bcatenin[0] == 1 && YAP1[0] == 1 && HNF4A[0] == 0 && OCT4[0] == 0 && NANOG[0] == 1 && p53[0] == 0 && p21[0] == 0 && RB[0] == 0 || BMI1[0] == 0 && E2F[0] == 0 && Bcatenin[0] == 1 && YAP1[0] == 1 && HNF4A[0] == 0 && OCT4[0] == 1 && NANOG[0] == 0 && p53[0] == 0 && p21[0] == 0 && RB[0] == 0 || BMI1[0] == 0 && E2F[0] == 0 && Bcatenin[0] == 1 && YAP1[0] == 1 && HNF4A[0] == 0 && OCT4[0] == 1 && NANOG[0] == 1 && p53[0] == 0 && p21[0] == 0 || BMI1[0] == 0 && E2F[0] == 0 && Bcatenin[0] == 1 && YAP1[0] == 1 && HNF4A[0] == 1 && NFkB[0] == 0 && SNAI1[0] == 0 && ZEB1[0] == 0 && TGFB[0] == 0 && OCT4[0] == 0 && NANOG[0] == 1 && SOX2[0] == 1 && p53[0] == 0 && p21[0] == 0 && RB[0] == 0 || BMI1[0] == 0 && E2F[0] == 0 && Bcatenin[0] == 1 && YAP1[0] == 1 && HNF4A[0] == 1 && NFkB[0] == 0 && SNAI1[0] == 0 && ZEB1[0] == 0 && TGFB[0] == 0 && OCT4[0] == 1 && NANOG[0] == 1 && p53[0] == 0 && p21[0] == 0 || BMI1[0] == 0 && E2F[0] == 0 && Bcatenin[0] == 1 && YAP1[0] == 1 && HNF4A[0] == 1 && NFkB[0] == 0 && SNAI1[0] == 0 && ZEB1[0] == 0 && TGFB[0] == 1 && OCT4[0] == 1 && NANOG[0] == 1 && p53[0] == 0 && p21[0] == 0 || BMI1[0] == 0 && E2F[0] == 0 && Bcatenin[0] == 1 && YAP1[0] == 1 && HNF4A[0] == 1 && NFkB[0] == 0 && SNAI1[0] == 0 && ZEB1[0] == 1 && OCT4[0] == 0 && NANOG[0] == 1 && p53[0] == 0 && p21[0] == 0 && RB[0] == 0 || BMI1[0] == 0 && E2F[0] == 0 && Bcatenin[0] == 1 && YAP1[0] == 1 && HNF4A[0] == 1 && NFkB[0] == 0 && SNAI1[0] == 0 && ZEB1[0] == 1 && OCT4[0] == 1 && NANOG[0] == 0 && p53[0] == 0 && p21[0] == 0 && RB[0] == 0 || BMI1[0] == 0 && E2F[0] == 0 && Bcatenin[0] == 1 && YAP1[0] == 1 && HNF4A[0] == 1 && NFkB[0] == 0 && SNAI1[0] == 0 && ZEB1[0] == 1 && OCT4[0] == 1 && NANOG[0] == 1 && p53[0] == 0 && p21[0] == 0 || BMI1[0] == 0 && E2F[0] == 0 && Bcatenin[0] == 1 && YAP1[0] == 1 && HNF4A[0] == 1 && NFkB[0] == 0 && SNAI1[0] == 1 && OCT4[0] == 0 && NANOG[0] == 1 && p53[0] == 0 && p21[0] == 0 && RB[0] == 0 || BMI1[0] == 0 && E2F[0] == 0 && Bcatenin[0] == 1 && YAP1[0] == 1 && HNF4A[0] == 1 && NFkB[0] == 0 && SNAI1[0] == 1 && OCT4[0] == 1 && NANOG[0] == 0 && p53[0] == 0 && p21[0] == 0 && RB[0] == 0 || BMI1[0] == 0 && E2F[0] == 0 && Bcatenin[0] == 1 && YAP1[0] == 1 && HNF4A[0] == 1 && NFkB[0] == 0 && SNAI1[0] == 1 && OCT4[0] == 1 && NANOG[0] == 1 && p53[0] == 0 && p21[0] == 0 || BMI1[0] == 0 && E2F[0] == 0 && Bcatenin[0] == 1 && YAP1[0] == 1 && HNF4A[0] == 1 && NFkB[0] == 1 && SNAI1[0] == 0 && ZEB1[0] == 0 && OCT4[0] == 0 && NANOG[0] == 1 && SOX2[0] == 1 && p53[0] == 0 && p21[0] == 0 && RB[0] == 0 || BMI1[0] == 0 && E2F[0] == 0 && Bcatenin[0] == 1 && YAP1[0] == 1 && HNF4A[0] == 1 && NFkB[0] == 1 && SNAI1[0] == 0 && ZEB1[0] == 0 && OCT4[0] == 1 && NANOG[0] == 1 && p53[0] == 0 && p21[0] == 0 || BMI1[0] == 0 && E2F[0] == 0 && Bcatenin[0] == 1 && YAP1[0] == 1 && HNF4A[0] == 1 && NFkB[0] == 1 && SNAI1[0] == 0 && ZEB1[0] == 1 && OCT4[0] == 0 && NANOG[0] == 1 && p53[0] == 0 && p21[0] == 0 && RB[0] == 0 || BMI1[0] == 0 && E2F[0] == 0 && Bcatenin[0] == 1 && YAP1[0] == 1 && HNF4A[0] == 1 && NFkB[0] == 1 && SNAI1[0] == 0 && ZEB1[0] == 1 && OCT4[0] == 1 && NANOG[0] == 0 && p53[0] == 0 && p21[0] == 0 && RB[0] == 0 || BMI1[0] == 0 && E2F[0] == 0 && Bcatenin[0] == 1 && YAP1[0] == 1 && HNF4A[0] == 1 && NFkB[0] == 1 && SNAI1[0] == 0 && ZEB1[0] == 1 && OCT4[0] == 1 && NANOG[0] == 1 && p53[0] == 0 && p21[0] == 0 || BMI1[0] == 0 && E2F[0] == 0 && Bcatenin[0] == 1 && YAP1[0] == 1 && HNF4A[0] == 1 && NFkB[0] == 1 && SNAI1[0] == 1 && OCT4[0] == 0 && NANOG[0] == 1 && p53[0] == 0 && p21[0] == 0 && RB[0] == 0 || BMI1[0] == 0 && E2F[0] == 0 && Bcatenin[0] == 1 && YAP1[0] == 1 && HNF4A[0] == 1 && NFkB[0] == 1 && SNAI1[0] == 1 && OCT4[0] == 1 && NANOG[0] == 0 && p53[0] == 0 && p21[0] == 0 && RB[0] == 0 || BMI1[0] == 0 && E2F[0] == 0 && Bcatenin[0] == 1 && YAP1[0] == 1 && HNF4A[0] == 1 && NFkB[0] == 1 && SNAI1[0] == 1 && OCT4[0] == 1 && NANOG[0] == 1 && p53[0] == 0 && p21[0] == 0 || BMI1[0] == 0 && E2F[0] == 1 && Bcatenin[0] == 0 && YAP1[0] == 0 && HNF4A[0] == 0 && NFkB[0] == 0 && NANOG[0] == 1 && SOX2[0] == 1 && p53[0] == 0 && p21[0] == 0 && RB[0] == 0 || BMI1[0] == 0 && E2F[0] == 1 && Bcatenin[0] == 0 && YAP1[0] == 0 && HNF4A[0] == 0 && NFkB[0] == 1 && OCT4[0] == 0 && NANOG[0] == 1 && SOX2[0] == 1 && p53[0] == 0 && p21[0] == 0 && RB[0] == 0 || BMI1[0] == 0 && E2F[0] == 1 && Bcatenin[0] == 0 && YAP1[0] == 0 && HNF4A[0] == 0 && NFkB[0] == 1 && OCT4[0] == 1 && NANOG[0] == 1 && p53[0] == 0 && p21[0] == 0 && RB[0] == 0 || BMI1[0] == 0 && E2F[0] == 1 && Bcatenin[0] == 0 && YAP1[0] == 0 && HNF4A[0] == 1 && NFkB[0] == 0 && SNAI1[0] == 0 && ZEB1[0] == 0 && TGFB[0] == 0 && NANOG[0] == 1 && SOX2[0] == 1 && p53[0] == 0 && p21[0] == 0 && RB[0] == 0 || BMI1[0] == 0 && E2F[0] == 1 && Bcatenin[0] == 0 && YAP1[0] == 0 && HNF4A[0] == 1 && NFkB[0] == 0 && SNAI1[0] == 0 && ZEB1[0] == 0 && TGFB[0] == 1 && OCT4[0] == 1 && NANOG[0] == 1 && SOX2[0] == 1 && p53[0] == 0 && p21[0] == 0 && RB[0] == 0 || BMI1[0] == 0 && E2F[0] == 1 && Bcatenin[0] == 0 && YAP1[0] == 0 && HNF4A[0] == 1 && NFkB[0] == 0 && SNAI1[0] == 0 && ZEB1[0] == 1 && NANOG[0] == 1 && SOX2[0] == 1 && p53[0] == 0 && p21[0] == 0 && RB[0] == 0 || BMI1[0] == 0 && E2F[0] == 1 && Bcatenin[0] == 0 && YAP1[0] == 0 && HNF4A[0] == 1 && NFkB[0] == 0 && SNAI1[0] == 1 && NANOG[0] == 1 && SOX2[0] == 1 && p53[0] == 0 && p21[0] == 0 && RB[0] == 0 || BMI1[0] == 0 && E2F[0] == 1 && Bcatenin[0] == 0 && YAP1[0] == 0 && HNF4A[0] == 1 && NFkB[0] == 1 && OCT4[0] == 0 && NANOG[0] == 1 && SOX2[0] == 1 && p53[0] == 0 && p21[0] == 0 && RB[0] == 0 || BMI1[0] == 0 && E2F[0] == 1 && Bcatenin[0] == 0 && YAP1[0] == 0 && HNF4A[0] == 1 && NFkB[0] == 1 && OCT4[0] == 1 && NANOG[0] == 1 && p53[0] == 0 && p21[0] == 0 && RB[0] == 0 || BMI1[0] == 0 && E2F[0] == 1 && Bcatenin[0] == 0 && YAP1[0] == 1 && HNF4A[0] == 0 && OCT4[0] == 0 && NANOG[0] == 1 && p53[0] == 0 && p21[0] == 0 && RB[0] == 0 || BMI1[0] == 0 && E2F[0] == 1 && Bcatenin[0] == 0 && YAP1[0] == 1 && HNF4A[0] == 0 && OCT4[0] == 1 && p53[0] == 0 && p21[0] == 0 && RB[0] == 0 || BMI1[0] == 0 && E2F[0] == 1 && Bcatenin[0] == 0 && YAP1[0] == 1 && HNF4A[0] == 1 && NFkB[0] == 0 && SNAI1[0] == 0 && ZEB1[0] == 0 && TGFB[0] == 0 && OCT4[0] == 0 && NANOG[0] == 1 && SOX2[0] == 1 && p53[0] == 0 && p21[0] == 0 && RB[0] == 0 || BMI1[0] == 0 && E2F[0] == 1 && Bcatenin[0] == 0 && YAP1[0] == 1 && HNF4A[0] == 1 && NFkB[0] == 0 && SNAI1[0] == 0 && ZEB1[0] == 0 && TGFB[0] == 0 && OCT4[0] == 1 && NANOG[0] == 1 && p53[0] == 0 && p21[0] == 0 && RB[0] == 0 || BMI1[0] == 0 && E2F[0] == 1 && Bcatenin[0] == 0 && YAP1[0] == 1 && HNF4A[0] == 1 && NFkB[0] == 0 && SNAI1[0] == 0 && ZEB1[0] == 0 && TGFB[0] == 1 && OCT4[0] == 1 && NANOG[0] == 1 && SOX2[0] == 1 && p53[0] == 0 && p21[0] == 0 && RB[0] == 0 || BMI1[0] == 0 && E2F[0] == 1 && Bcatenin[0] == 0 && YAP1[0] == 1 && HNF4A[0] == 1 && NFkB[0] == 0 && SNAI1[0] == 0 && ZEB1[0] == 1 && OCT4[0] == 0 && NANOG[0] == 1 && p53[0] == 0 && p21[0] == 0 && RB[0] == 0 || BMI1[0] == 0 && E2F[0] == 1 && Bcatenin[0] == 0 && YAP1[0] == 1 && HNF4A[0] == 1 && NFkB[0] == 0 && SNAI1[0] == 0 && ZEB1[0] == 1 && OCT4[0] == 1 && p53[0] == 0 && p21[0] == 0 && RB[0] == 0 || BMI1[0] == 0 && E2F[0] == 1 && Bcatenin[0] == 0 && YAP1[0] == 1 && HNF4A[0] == 1 && NFkB[0] == 0 && SNAI1[0] == 1 && OCT4[0] == 0 && NANOG[0] == 1 && p53[0] == 0 && p21[0] == 0 && RB[0] == 0 || BMI1[0] == 0 && E2F[0] == 1 && Bcatenin[0] == 0 && YAP1[0] == 1 && HNF4A[0] == 1 && NFkB[0] == 0 && SNAI1[0] == 1 && OCT4[0] == 1 && p53[0] == 0 && p21[0] == 0 && RB[0] == 0 || BMI1[0] == 0 && E2F[0] == 1 && Bcatenin[0] == 0 && YAP1[0] == 1 && HNF4A[0] == 1 && NFkB[0] == 1 && SNAI1[0] == 0 && ZEB1[0] == 0 && OCT4[0] == 0 && NANOG[0] == 1 && SOX2[0] == 1 && p53[0] == 0 && p21[0] == 0 && RB[0] == 0 || BMI1[0] == 0 && E2F[0] == 1 && Bcatenin[0] == 0 && YAP1[0] == 1 && HNF4A[0] == 1 && NFkB[0] == 1 && SNAI1[0] == 0 && ZEB1[0] == 0 && OCT4[0] == 1 && NANOG[0] == 1 && p53[0] == 0 && p21[0] == 0 && RB[0] == 0 || BMI1[0] == 0 && E2F[0] == 1 && Bcatenin[0] == 0 && YAP1[0] == 1 && HNF4A[0] == 1 && NFkB[0] == 1 && SNAI1[0] == 0 && ZEB1[0] == 1 && OCT4[0] == 0 && NANOG[0] == 1 && p53[0] == 0 && p21[0] == 0 && RB[0] == 0 || BMI1[0] == 0 && E2F[0] == 1 && Bcatenin[0] == 0 && YAP1[0] == 1 && HNF4A[0] == 1 && NFkB[0] == 1 && SNAI1[0] == 0 && ZEB1[0] == 1 && OCT4[0] == 1 && p53[0] == 0 && p21[0] == 0 && RB[0] == 0 || BMI1[0] == 0 && E2F[0] == 1 && Bcatenin[0] == 0 && YAP1[0] == 1 && HNF4A[0] == 1 && NFkB[0] == 1 && SNAI1[0] == 1 && OCT4[0] == 0 && NANOG[0] == 1 && p53[0] == 0 && p21[0] == 0 && RB[0] == 0 || BMI1[0] == 0 && E2F[0] == 1 && Bcatenin[0] == 0 && YAP1[0] == 1 && HNF4A[0] == 1 && NFkB[0] == 1 && SNAI1[0] == 1 && OCT4[0] == 1 && p53[0] == 0 && p21[0] == 0 && RB[0] == 0 || BMI1[0] == 0 && E2F[0] == 1 && Bcatenin[0] == 1 && HNF4A[0] == 0 && OCT4[0] == 0 && NANOG[0] == 1 && p53[0] == 0 && p21[0] == 0 && RB[0] == 0 || BMI1[0] == 0 && E2F[0] == 1 && Bcatenin[0] == 1 && HNF4A[0] == 0 && OCT4[0] == 1 && NANOG[0] == 0 && p53[0] == 0 && p21[0] == 0 && RB[0] == 0 || BMI1[0] == 0 && E2F[0] == 1 && Bcatenin[0] == 1 && HNF4A[0] == 0 && OCT4[0] == 1 && NANOG[0] == 1 && p53[0] == 0 && p21[0] == 0 || BMI1[0] == 0 && E2F[0] == 1 && Bcatenin[0] == 1 && HNF4A[0] == 1 && NFkB[0] == 0 && SNAI1[0] == 0 && ZEB1[0] == 0 && TGFB[0] == 0 && OCT4[0] == 0 && NANOG[0] == 1 && SOX2[0] == 1 && p53[0] == 0 && p21[0] == 0 && RB[0] == 0 || BMI1[0] == 0 && E2F[0] == 1 && Bcatenin[0] == 1 && HNF4A[0] == 1 && NFkB[0] == 0 && SNAI1[0] == 0 && ZEB1[0] == 0 && TGFB[0] == 0 && OCT4[0] == 1 && NANOG[0] == 1 && p53[0] == 0 && p21[0] == 0 || BMI1[0] == 0 && E2F[0] == 1 && Bcatenin[0] == 1 && HNF4A[0] == 1 && NFkB[0] == 0 && SNAI1[0] == 0 && ZEB1[0] == 0 && TGFB[0] == 1 && OCT4[0] == 1 && NANOG[0] == 1 && p53[0] == 0 && p21[0] == 0 || BMI1[0] == 0 && E2F[0] == 1 && Bcatenin[0] == 1 && HNF4A[0] == 1 && NFkB[0] == 0 && SNAI1[0] == 0 && ZEB1[0] == 1 && OCT4[0] == 0 && NANOG[0] == 1 && p53[0] == 0 && p21[0] == 0 && RB[0] == 0 || BMI1[0] == 0 && E2F[0] == 1 && Bcatenin[0] == 1 && HNF4A[0] == 1 && NFkB[0] == 0 && SNAI1[0] == 0 && ZEB1[0] == 1 && OCT4[0] == 1 && NANOG[0] == 0 && p53[0] == 0 && p21[0] == 0 && RB[0] == 0 || BMI1[0] == 0 && E2F[0] == 1 && Bcatenin[0] == 1 && HNF4A[0] == 1 && NFkB[0] == 0 && SNAI1[0] == 0 && ZEB1[0] == 1 && OCT4[0] == 1 && NANOG[0] == 1 && p53[0] == 0 && p21[0] == 0 || BMI1[0] == 0 && E2F[0] == 1 && Bcatenin[0] == 1 && HNF4A[0] == 1 && NFkB[0] == 0 && SNAI1[0] == 1 && OCT4[0] == 0 && NANOG[0] == 1 && p53[0] == 0 && p21[0] == 0 && RB[0] == 0 || BMI1[0] == 0 && E2F[0] == 1 && Bcatenin[0] == 1 && HNF4A[0] == 1 && NFkB[0] == 0 && SNAI1[0] == 1 && OCT4[0] == 1 && NANOG[0] == 0 && p53[0] == 0 && p21[0] == 0 && RB[0] == 0 || BMI1[0] == 0 && E2F[0] == 1 && Bcatenin[0] == 1 && HNF4A[0] == 1 && NFkB[0] == 0 && SNAI1[0] == 1 && OCT4[0] == 1 && NANOG[0] == 1 && p53[0] == 0 && p21[0] == 0 || BMI1[0] == 0 && E2F[0] == 1 && Bcatenin[0] == 1 && HNF4A[0] == 1 && NFkB[0] == 1 && SNAI1[0] == 0 && ZEB1[0] == 0 && OCT4[0] == 0 && NANOG[0] == 1 && SOX2[0] == 1 && p53[0] == 0 && p21[0] == 0 && RB[0] == 0 || BMI1[0] == 0 && E2F[0] == 1 && Bcatenin[0] == 1 && HNF4A[0] == 1 && NFkB[0] == 1 && SNAI1[0] == 0 && ZEB1[0] == 0 && OCT4[0] == 1 && NANOG[0] == 1 && p53[0] == 0 && p21[0] == 0 || BMI1[0] == 0 && E2F[0] == 1 && Bcatenin[0] == 1 && HNF4A[0] == 1 && NFkB[0] == 1 && SNAI1[0] == 0 && ZEB1[0] == 1 && OCT4[0] == 0 && NANOG[0] == 1 && p53[0] == 0 && p21[0] == 0 && RB[0] == 0 || BMI1[0] == 0 && E2F[0] == 1 && Bcatenin[0] == 1 && HNF4A[0] == 1 && NFkB[0] == 1 && SNAI1[0] == 0 && ZEB1[0] == 1 && OCT4[0] == 1 && NANOG[0] == 0 && p53[0] == 0 && p21[0] == 0 && RB[0] == 0 || BMI1[0] == 0 && E2F[0] == 1 && Bcatenin[0] == 1 && HNF4A[0] == 1 && NFkB[0] == 1 && SNAI1[0] == 0 && ZEB1[0] == 1 && OCT4[0] == 1 && NANOG[0] == 1 && p53[0] == 0 && p21[0] == 0 || BMI1[0] == 0 && E2F[0] == 1 && Bcatenin[0] == 1 && HNF4A[0] == 1 && NFkB[0] == 1 && SNAI1[0] == 1 && OCT4[0] == 0 && NANOG[0] == 1 && p53[0] == 0 && p21[0] == 0 && RB[0] == 0 || BMI1[0] == 0 && E2F[0] == 1 && Bcatenin[0] == 1 && HNF4A[0] == 1 && NFkB[0] == 1 && SNAI1[0] == 1 && OCT4[0] == 1 && NANOG[0] == 0 && p53[0] == 0 && p21[0] == 0 && RB[0] == 0 || BMI1[0] == 0 && E2F[0] == 1 && Bcatenin[0] == 1 && HNF4A[0] == 1 && NFkB[0] == 1 && SNAI1[0] == 1 && OCT4[0] == 1 && NANOG[0] == 1 && p53[0] == 0 && p21[0] == 0 || BMI1[0] == 1 && E2F[0] == 0 && Bcatenin[0] == 0 && YAP1[0] == 0 && OCT4[0] == 1 && NANOG[0] == 1 && SOX2[0] == 1 && p53[0] == 0 && p21[0] == 0 && RB[0] == 0 || BMI1[0] == 1 && E2F[0] == 0 && Bcatenin[0] == 0 && YAP1[0] == 1 && OCT4[0] == 0 && NANOG[0] == 1 && p53[0] == 0 && p21[0] == 0 && RB[0] == 0 || BMI1[0] == 1 && E2F[0] == 0 && Bcatenin[0] == 0 && YAP1[0] == 1 && OCT4[0] == 1 && p53[0] == 0 && p21[0] == 0 && RB[0] == 0 || BMI1[0] == 1 && E2F[0] == 0 && Bcatenin[0] == 1 && OCT4[0] == 0 && NANOG[0] == 1 && p53[0] == 0 && p21[0] == 0 && RB[0] == 0 || BMI1[0] == 1 && E2F[0] == 0 && Bcatenin[0] == 1 && OCT4[0] == 1 && NANOG[0] == 0 && p53[0] == 0 && p21[0] == 0 && RB[0] == 0 || BMI1[0] == 1 && E2F[0] == 0 && Bcatenin[0] == 1 && OCT4[0] == 1 && NANOG[0] == 1 && p53[0] == 0 && p21[0] == 0 || BMI1[0] == 1 && E2F[0] == 1 && Bcatenin[0] == 0 && YAP1[0] == 0 && HNF4A[0] == 0 && NFkB[0] == 0 && NANOG[0] == 1 && SOX2[0] == 1 && p53[0] == 0 && p21[0] == 0 && RB[0] == 0 || BMI1[0] == 1 && E2F[0] == 1 && Bcatenin[0] == 0 && YAP1[0] == 0 && HNF4A[0] == 0 && NFkB[0] == 1 && OCT4[0] == 0 && NANOG[0] == 1 && SOX2[0] == 1 && p53[0] == 0 && p21[0] == 0 && RB[0] == 0 || BMI1[0] == 1 && E2F[0] == 1 && Bcatenin[0] == 0 && YAP1[0] == 0 && HNF4A[0] == 0 && NFkB[0] == 1 && OCT4[0] == 1 && NANOG[0] == 1 && p53[0] == 0 && p21[0] == 0 && RB[0] == 0 || BMI1[0] == 1 && E2F[0] == 1 && Bcatenin[0] == 0 && YAP1[0] == 0 && HNF4A[0] == 1 && NFkB[0] == 0 && SNAI1[0] == 0 && ZEB1[0] == 0 && TGFB[0] == 0 && NANOG[0] == 1 && SOX2[0] == 1 && p53[0] == 0 && p21[0] == 0 && RB[0] == 0 || BMI1[0] == 1 && E2F[0] == 1 && Bcatenin[0] == 0 && YAP1[0] == 0 && HNF4A[0] == 1 && NFkB[0] == 0 && SNAI1[0] == 0 && ZEB1[0] == 0 && TGFB[0] == 1 && OCT4[0] == 1 && NANOG[0] == 1 && SOX2[0] == 1 && p53[0] == 0 && p21[0] == 0 && RB[0] == 0 || BMI1[0] == 1 && E2F[0] == 1 && Bcatenin[0] == 0 && YAP1[0] == 0 && HNF4A[0] == 1 && NFkB[0] == 0 && SNAI1[0] == 0 && ZEB1[0] == 1 && NANOG[0] == 1 && SOX2[0] == 1 && p53[0] == 0 && p21[0] == 0 && RB[0] == 0 || BMI1[0] == 1 && E2F[0] == 1 && Bcatenin[0] == 0 && YAP1[0] == 0 && HNF4A[0] == 1 && NFkB[0] == 0 && SNAI1[0] == 1 && NANOG[0] == 1 && SOX2[0] == 1 && p53[0] == 0 && p21[0] == 0 && RB[0] == 0 || BMI1[0] == 1 && E2F[0] == 1 && Bcatenin[0] == 0 && YAP1[0] == 0 && HNF4A[0] == 1 && NFkB[0] == 1 && OCT4[0] == 0 && NANOG[0] == 1 && SOX2[0] == 1 && p53[0] == 0 && p21[0] == 0 && RB[0] == 0 || BMI1[0] == 1 && E2F[0] == 1 && Bcatenin[0] == 0 && YAP1[0] == 0 && HNF4A[0] == 1 && NFkB[0] == 1 && OCT4[0] == 1 && NANOG[0] == 1 && p53[0] == 0 && p21[0] == 0 && RB[0] == 0 || BMI1[0] == 1 && E2F[0] == 1 && Bcatenin[0] == 0 && YAP1[0] == 1 && OCT4[0] == 0 && NANOG[0] == 1 && p53[0] == 0 && p21[0] == 0 && RB[0] == 0 || BMI1[0] == 1 && E2F[0] == 1 && Bcatenin[0] == 0 && YAP1[0] == 1 && OCT4[0] == 1 && p53[0] == 0 && p21[0] == 0 && RB[0] == 0 || BMI1[0] == 1 && E2F[0] == 1 && Bcatenin[0] == 1 && OCT4[0] == 0 && NANOG[0] == 1 && p53[0] == 0 && p21[0] == 0 && RB[0] == 0 || BMI1[0] == 1 && E2F[0] == 1 && Bcatenin[0] == 1 && OCT4[0] == 1 && NANOG[0] == 0 && p53[0] == 0 && p21[0] == 0 && RB[0] == 0 || BMI1[0] == 1 && E2F[0] == 1 && Bcatenin[0] == 1 && OCT4[0] == 1 && NANOG[0] == 1 && p53[0] == 0 && p21[0] == 0) { SOX2[0] = 1; }

else { SOX2[0] = 0; }

//Logic rule for NANOG

if (E2F[0] == 0 && Bcatenin[0] == 0 && NFkB[0] == 0 && SNAI1[0] == 0 && TGFB[0] == 0 && OCT4[0] == 0 && NANOG[0] == 1 && SOX2[0] == 1 && p53[0] == 0 || E2F[0] == 0 && Bcatenin[0] == 0 && NFkB[0] == 0 && SNAI1[0] == 0 && TGFB[0] == 0 && OCT4[0] == 1 && NANOG[0] == 1 && p53[0] == 0 || E2F[0] == 0 && Bcatenin[0] == 0 && NFkB[0] == 0 && SNAI1[0] == 0 && TGFB[0] == 1 && OCT4[0] == 0 && NANOG[0] == 1 && SOX2[0] == 1 && p53[0] == 0 || E2F[0] == 0 && Bcatenin[0] == 0 && NFkB[0] == 0 && SNAI1[0] == 0 && TGFB[0] == 1 && OCT4[0] == 1 && NANOG[0] == 0 && SOX2[0] == 1 && p53[0] == 0 || E2F[0] == 0 && Bcatenin[0] == 0 && NFkB[0] == 0 && SNAI1[0] == 0 && TGFB[0] == 1 && OCT4[0] == 1 && NANOG[0] == 1 && p53[0] == 0 || E2F[0] == 0 && Bcatenin[0] == 0 && NFkB[0] == 0 && SNAI1[0] == 1 && TGFB[0] == 1 && OCT4[0] == 1 && SOX2[0] == 1 && p53[0] == 0 || E2F[0] == 0 && Bcatenin[0] == 0 && NFkB[0] == 1 && OCT4[0] == 0 && NANOG[0] == 1 && SOX2[0] == 1 && p53[0] == 0 || E2F[0] == 0 && Bcatenin[0] == 0 && NFkB[0] == 1 && OCT4[0] == 1 && NANOG[0] == 0 && SOX2[0] == 1 && p53[0] == 0 || E2F[0] == 0 && Bcatenin[0] == 0 && NFkB[0] == 1 && OCT4[0] == 1 && NANOG[0] == 1 && p53[0] == 0 || E2F[0] == 0 && Bcatenin[0] == 1 && HNF4A[0] == 0 && NFkB[0] == 0 && SNAI1[0] == 0 && OCT4[0] == 0 && NANOG[0] == 1 && SOX2[0] == 1 && p53[0] == 0 || E2F[0] == 0 && Bcatenin[0] == 1 && HNF4A[0] == 0 && NFkB[0] == 0 && SNAI1[0] == 0 && OCT4[0] == 1 && NANOG[0] == 0 && SOX2[0] == 1 && p53[0] == 0 || E2F[0] == 0 && Bcatenin[0] == 1 && HNF4A[0] == 0 && NFkB[0] == 0 && SNAI1[0] == 0 && OCT4[0] == 1 && NANOG[0] == 1 && p53[0] == 0 || E2F[0] == 0 && Bcatenin[0] == 1 && HNF4A[0] == 0 && NFkB[0] == 0 && SNAI1[0] == 1 && TGFB[0] == 1 && OCT4[0] == 1 && SOX2[0] == 1 && p53[0] == 0 || E2F[0] == 0 && Bcatenin[0] == 1 && HNF4A[0] == 0 && NFkB[0] == 1 && OCT4[0] == 0 && NANOG[0] == 1 && SOX2[0] == 1 && p53[0] == 0 || E2F[0] == 0 && Bcatenin[0] == 1 && HNF4A[0] == 0 && NFkB[0] == 1 && OCT4[0] == 1 && NANOG[0] == 0 && SOX2[0] == 1 && p53[0] == 0 || E2F[0] == 0 && Bcatenin[0] == 1 && HNF4A[0] == 0 && NFkB[0] == 1 && OCT4[0] == 1 && NANOG[0] == 1 && p53[0] == 0 || E2F[0] == 0 && Bcatenin[0] == 1 && HNF4A[0] == 1 && NFkB[0] == 0 && SNAI1[0] == 0 && ZEB1[0] == 0 && TGFB[0] == 0 && OCT4[0] == 0 && NANOG[0] == 1 && SOX2[0] == 1 && p53[0] == 0 || E2F[0] == 0 && Bcatenin[0] == 1 && HNF4A[0] == 1 && NFkB[0] == 0 && SNAI1[0] == 0 && ZEB1[0] == 0 && TGFB[0] == 0 && OCT4[0] == 1 && NANOG[0] == 1 && p53[0] == 0 || E2F[0] == 0 && Bcatenin[0] == 1 && HNF4A[0] == 1 && NFkB[0] == 0 && SNAI1[0] == 0 && ZEB1[0] == 0 && TGFB[0] == 1 && OCT4[0] == 0 && NANOG[0] == 1 && SOX2[0] == 1 && p53[0] == 0 || E2F[0] == 0 && Bcatenin[0] == 1 && HNF4A[0] == 1 && NFkB[0] == 0 && SNAI1[0] == 0 && ZEB1[0] == 0 && TGFB[0] == 1 && OCT4[0] == 1 && NANOG[0] == 0 && SOX2[0] == 1 && p53[0] == 0 || E2F[0] == 0 && Bcatenin[0] == 1 && HNF4A[0] == 1 && NFkB[0] == 0 && SNAI1[0] == 0 && ZEB1[0] == 0 && TGFB[0] == 1 && OCT4[0] == 1 && NANOG[0] == 1 && p53[0] == 0 || E2F[0] == 0 && Bcatenin[0] == 1 && HNF4A[0] == 1 && NFkB[0] == 0 && SNAI1[0] == 0 && ZEB1[0] == 1 && OCT4[0] == 0 && NANOG[0] == 1 && SOX2[0] == 1 && p53[0] == 0 || E2F[0] == 0 && Bcatenin[0] == 1 && HNF4A[0] == 1 && NFkB[0] == 0 && SNAI1[0] == 0 && ZEB1[0] == 1 && OCT4[0] == 1 && NANOG[0] == 0 && SOX2[0] == 1 && p53[0] == 0 || E2F[0] == 0 && Bcatenin[0] == 1 && HNF4A[0] == 1 && NFkB[0] == 0 && SNAI1[0] == 0 && ZEB1[0] == 1 && OCT4[0] == 1 && NANOG[0] == 1 && p53[0] == 0 || E2F[0] == 0 && Bcatenin[0] == 1 && HNF4A[0] == 1 && NFkB[0] == 0 && SNAI1[0] == 1 && TGFB[0] == 1 && OCT4[0] == 1 && SOX2[0] == 1 && p53[0] == 0 || E2F[0] == 0 && Bcatenin[0] == 1 && HNF4A[0] == 1 && NFkB[0] == 1 && OCT4[0] == 0 && NANOG[0] == 1 && SOX2[0] == 1 && p53[0] == 0 || E2F[0] == 0 && Bcatenin[0] == 1 && HNF4A[0] == 1 && NFkB[0] == 1 && OCT4[0] == 1 && NANOG[0] == 0 && SOX2[0] == 1 && p53[0] == 0 || E2F[0] == 0 && Bcatenin[0] == 1 && HNF4A[0] == 1 && NFkB[0] == 1 && OCT4[0] == 1 && NANOG[0] == 1 && p53[0] == 0 || E2F[0] == 1 && HNF4A[0] == 0 && NFkB[0] == 0 && SNAI1[0] == 0 && OCT4[0] == 0 && NANOG[0] == 1 && SOX2[0] == 1 && p53[0] == 0 || E2F[0] == 1 && HNF4A[0] == 0 && NFkB[0] == 0 && SNAI1[0] == 0 && OCT4[0] == 1 && NANOG[0] == 0 && SOX2[0] == 1 && p53[0] == 0 || E2F[0] == 1 && HNF4A[0] == 0 && NFkB[0] == 0 && SNAI1[0] == 0 && OCT4[0] == 1 && NANOG[0] == 1 && p53[0] == 0 || E2F[0] == 1 && HNF4A[0] == 0 && NFkB[0] == 0 && SNAI1[0] == 1 && TGFB[0] == 1 && OCT4[0] == 1 && SOX2[0] == 1 && p53[0] == 0 || E2F[0] == 1 && HNF4A[0] == 0 && NFkB[0] == 1 && OCT4[0] == 0 && NANOG[0] == 1 && SOX2[0] == 1 && p53[0] == 0 || E2F[0] == 1 && HNF4A[0] == 0 && NFkB[0] == 1 && OCT4[0] == 1 && NANOG[0] == 0 && SOX2[0] == 1 && p53[0] == 0 || E2F[0] == 1 && HNF4A[0] == 0 && NFkB[0] == 1 && OCT4[0] == 1 && NANOG[0] == 1 && p53[0] == 0 || E2F[0] == 1 && HNF4A[0] == 1 && NFkB[0] == 0 && SNAI1[0] == 0 && ZEB1[0] == 0 && TGFB[0] == 0 && OCT4[0] == 0 && NANOG[0] == 1 && SOX2[0] == 1 && p53[0] == 0 || E2F[0] == 1 && HNF4A[0] == 1 && NFkB[0] == 0 && SNAI1[0] == 0 && ZEB1[0] == 0 && TGFB[0] == 0 && OCT4[0] == 1 && NANOG[0] == 1 && p53[0] == 0 || E2F[0] == 1 && HNF4A[0] == 1 && NFkB[0] == 0 && SNAI1[0] == 0 && ZEB1[0] == 0 && TGFB[0] == 1 && OCT4[0] == 0 && NANOG[0] == 1 && SOX2[0] == 1 && p53[0] == 0 || E2F[0] == 1 && HNF4A[0] == 1 && NFkB[0] == 0 && SNAI1[0] == 0 && ZEB1[0] == 0 && TGFB[0] == 1 && OCT4[0] == 1 && NANOG[0] == 0 && SOX2[0] == 1 && p53[0] == 0 || E2F[0] == 1 && HNF4A[0] == 1 && NFkB[0] == 0 && SNAI1[0] == 0 && ZEB1[0] == 0 && TGFB[0] == 1 && OCT4[0] == 1 && NANOG[0] == 1 && p53[0] == 0 || E2F[0] == 1 && HNF4A[0] == 1 && NFkB[0] == 0 && SNAI1[0] == 0 && ZEB1[0] == 1 && OCT4[0] == 0 && NANOG[0] == 1 && SOX2[0] == 1 && p53[0] == 0 || E2F[0] == 1 && HNF4A[0] == 1 && NFkB[0] == 0 && SNAI1[0] == 0 && ZEB1[0] == 1 && OCT4[0] == 1 && NANOG[0] == 0 && SOX2[0] == 1 && p53[0] == 0 || E2F[0] == 1 && HNF4A[0] == 1 && NFkB[0] == 0 && SNAI1[0] == 0 && ZEB1[0] == 1 && OCT4[0] == 1 && NANOG[0] == 1 && p53[0] == 0 || E2F[0] == 1 && HNF4A[0] == 1 && NFkB[0] == 0 && SNAI1[0] == 1 && TGFB[0] == 1 && OCT4[0] == 1 && SOX2[0] == 1 && p53[0] == 0 || E2F[0] == 1 && HNF4A[0] == 1 && NFkB[0] == 1 && OCT4[0] == 0 && NANOG[0] == 1 && SOX2[0] == 1 && p53[0] == 0 || E2F[0] == 1 && HNF4A[0] == 1 && NFkB[0] == 1 && OCT4[0] == 1 && NANOG[0] == 0 && SOX2[0] == 1 && p53[0] == 0 || E2F[0] == 1 && HNF4A[0] == 1 && NFkB[0] == 1 && OCT4[0] == 1 && NANOG[0] == 1 && p53[0] == 0) { NANOG[0] = 1; }

else { NANOG[0] = 0; }

//Logic rule for SOX9

if (YAP1[0] == 1 && FOXA2[0] == 0 && NFkB[0] == 0 && SOX9[0] == 1 || YAP1[0] == 1 && FOXA2[0] == 0 && NFkB[0] == 1 || YAP1[0] == 1 && FOXA2[0] == 1) { SOX9[0] = 1; }

else { SOX9[0] = 0; }

//Logic rule for p16

if (EZH2[0] == 0 && BMI1[0] == 0 && E2F[0] == 0 || EZH2[0] == 0 && BMI1[0] == 0 && E2F[0] == 1 && Bcatenin[0] == 0 && YAP1[0] == 0 && HNF4A[0] == 0 && NFkB[0] == 0 && SOX2[0] == 0 || EZH2[0] == 0 && BMI1[0] == 0 && E2F[0] == 1 && Bcatenin[0] == 0 && YAP1[0] == 0 && HNF4A[0] == 0 && NFkB[0] == 0 && SOX2[0] == 1 && p53[0] == 1 || EZH2[0] == 0 && BMI1[0] == 0 && E2F[0] == 1 && Bcatenin[0] == 0 && YAP1[0] == 0 && HNF4A[0] == 0 && NFkB[0] == 1 && p53[0] == 1 || EZH2[0] == 0 && BMI1[0] == 0 && E2F[0] == 1 && Bcatenin[0] == 0 && YAP1[0] == 0 && HNF4A[0] == 1 && NFkB[0] == 0 && SNAI1[0] == 0 && ZEB1[0] == 0 && TGFB[0] == 0 && SOX2[0] == 0 || EZH2[0] == 0 && BMI1[0] == 0 && E2F[0] == 1 && Bcatenin[0] == 0 && YAP1[0] == 0 && HNF4A[0] == 1 && NFkB[0] == 0 && SNAI1[0] == 0 && ZEB1[0] == 0 && TGFB[0] == 0 && SOX2[0] == 1 && p53[0] == 1 || EZH2[0] == 0 && BMI1[0] == 0 && E2F[0] == 1 && Bcatenin[0] == 0 && YAP1[0] == 0 && HNF4A[0] == 1 && NFkB[0] == 0 && SNAI1[0] == 0 && ZEB1[0] == 0 && TGFB[0] == 1 || EZH2[0] == 0 && BMI1[0] == 0 && E2F[0] == 1 && Bcatenin[0] == 0 && YAP1[0] == 0 && HNF4A[0] == 1 && NFkB[0] == 0 && SNAI1[0] == 0 && ZEB1[0] == 1 && SOX2[0] == 0 || EZH2[0] == 0 && BMI1[0] == 0 && E2F[0] == 1 && Bcatenin[0] == 0 && YAP1[0] == 0 && HNF4A[0] == 1 && NFkB[0] == 0 && SNAI1[0] == 0 && ZEB1[0] == 1 && SOX2[0] == 1 && p53[0] == 1 || EZH2[0] == 0 && BMI1[0] == 0 && E2F[0] == 1 && Bcatenin[0] == 0 && YAP1[0] == 0 && HNF4A[0] == 1 && NFkB[0] == 0 && SNAI1[0] == 1 && SOX2[0] == 0 || EZH2[0] == 0 && BMI1[0] == 0 && E2F[0] == 1 && Bcatenin[0] == 0 && YAP1[0] == 0 && HNF4A[0] == 1 && NFkB[0] == 0 && SNAI1[0] == 1 && SOX2[0] == 1 && p53[0] == 1 || EZH2[0] == 0 && BMI1[0] == 0 && E2F[0] == 1 && Bcatenin[0] == 0 && YAP1[0] == 0 && HNF4A[0] == 1 && NFkB[0] == 1 && p53[0] == 1 || EZH2[0] == 0 && BMI1[0] == 0 && E2F[0] == 1 && Bcatenin[0] == 0 && YAP1[0] == 1 && HNF4A[0] == 0 && p53[0] == 1 || EZH2[0] == 0 && BMI1[0] == 0 && E2F[0] == 1 && Bcatenin[0] == 0 && YAP1[0] == 1 && HNF4A[0] == 1 && NFkB[0] == 0 && SNAI1[0] == 0 && ZEB1[0] == 0 && TGFB[0] == 0 && p53[0] == 1 || EZH2[0] == 0 && BMI1[0] == 0 && E2F[0] == 1 && Bcatenin[0] == 0 && YAP1[0] == 1 && HNF4A[0] == 1 && NFkB[0] == 0 && SNAI1[0] == 0 && ZEB1[0] == 0 && TGFB[0] == 1 || EZH2[0] == 0 && BMI1[0] == 0 && E2F[0] == 1 && Bcatenin[0] == 0 && YAP1[0] == 1 && HNF4A[0] == 1 && NFkB[0] == 0 && SNAI1[0] == 0 && ZEB1[0] == 1 && p53[0] == 1 || EZH2[0] == 0 && BMI1[0] == 0 && E2F[0] == 1 && Bcatenin[0] == 0 && YAP1[0] == 1 && HNF4A[0] == 1 && NFkB[0] == 0 && SNAI1[0] == 1 && p53[0] == 1 || EZH2[0] == 0 && BMI1[0] == 0 && E2F[0] == 1 && Bcatenin[0] == 0 && YAP1[0] == 1 && HNF4A[0] == 1 && NFkB[0] == 1 && p53[0] == 1 || EZH2[0] == 0 && BMI1[0] == 0 && E2F[0] == 1 && Bcatenin[0] == 1 && HNF4A[0] == 0 && p53[0] == 1 || EZH2[0] == 0 && BMI1[0] == 0 && E2F[0] == 1 && Bcatenin[0] == 1 && HNF4A[0] == 1 && NFkB[0] == 0 && SNAI1[0] == 0 && ZEB1[0] == 0 && TGFB[0] == 0 && p53[0] == 1 || EZH2[0] == 0 && BMI1[0] == 0 && E2F[0] == 1 && Bcatenin[0] == 1 && HNF4A[0] == 1 && NFkB[0] == 0 && SNAI1[0] == 0 && ZEB1[0] == 0 && TGFB[0] == 1 || EZH2[0] == 0 && BMI1[0] == 0 && E2F[0] == 1 && Bcatenin[0] == 1 && HNF4A[0] == 1 && NFkB[0] == 0 && SNAI1[0] == 0 && ZEB1[0] == 1 && p53[0] == 1 || EZH2[0] == 0 && BMI1[0] == 0 && E2F[0] == 1 && Bcatenin[0] == 1 && HNF4A[0] == 1 && NFkB[0] == 0 && SNAI1[0] == 1 && p53[0] == 1 || EZH2[0] == 0 && BMI1[0] == 0 && E2F[0] == 1 && Bcatenin[0] == 1 && HNF4A[0] == 1 && NFkB[0] == 1 && p53[0] == 1 || EZH2[0] == 0 && BMI1[0] == 1 && E2F[0] == 0 && SNAI2[0] == 0 || EZH2[0] == 0 && BMI1[0] == 1 && E2F[0] == 1 && Bcatenin[0] == 0 && YAP1[0] == 0 && HNF4A[0] == 0 && NFkB[0] == 0 && SNAI2[0] == 0 && SOX2[0] == 0 || EZH2[0] == 0 && BMI1[0] == 1 && E2F[0] == 1 && Bcatenin[0] == 0 && YAP1[0] == 0 && HNF4A[0] == 0 && NFkB[0] == 0 && SNAI2[0] == 0 && SOX2[0] == 1 && p53[0] == 1 || EZH2[0] == 0 && BMI1[0] == 1 && E2F[0] == 1 && Bcatenin[0] == 0 && YAP1[0] == 0 && HNF4A[0] == 0 && NFkB[0] == 1 && SNAI2[0] == 0 && p53[0] == 1 || EZH2[0] == 0 && BMI1[0] == 1 && E2F[0] == 1 && Bcatenin[0] == 0 && YAP1[0] == 0 && HNF4A[0] == 1 && NFkB[0] == 0 && SNAI1[0] == 0 && ZEB1[0] == 0 && SNAI2[0] == 0 && TGFB[0] == 0 && SOX2[0] == 0 || EZH2[0] == 0 && BMI1[0] == 1 && E2F[0] == 1 && Bcatenin[0] == 0 && YAP1[0] == 0 && HNF4A[0] == 1 && NFkB[0] == 0 && SNAI1[0] == 0 && ZEB1[0] == 0 && SNAI2[0] == 0 && TGFB[0] == 0 && SOX2[0] == 1 && p53[0] == 1 || EZH2[0] == 0 && BMI1[0] == 1 && E2F[0] == 1 && Bcatenin[0] == 0 && YAP1[0] == 0 && HNF4A[0] == 1 && NFkB[0] == 0 && SNAI1[0] == 0 && ZEB1[0] == 0 && SNAI2[0] == 0 && TGFB[0] == 1 || EZH2[0] == 0 && BMI1[0] == 1 && E2F[0] == 1 && Bcatenin[0] == 0 && YAP1[0] == 0 && HNF4A[0] == 1 && NFkB[0] == 0 && SNAI1[0] == 0 && ZEB1[0] == 1 && SNAI2[0] == 0 && SOX2[0] == 0 || EZH2[0] == 0 && BMI1[0] == 1 && E2F[0] == 1 && Bcatenin[0] == 0 && YAP1[0] == 0 && HNF4A[0] == 1 && NFkB[0] == 0 && SNAI1[0] == 0 && ZEB1[0] == 1 && SNAI2[0] == 0 && SOX2[0] == 1 && p53[0] == 1 || EZH2[0] == 0 && BMI1[0] == 1 && E2F[0] == 1 && Bcatenin[0] == 0 && YAP1[0] == 0 && HNF4A[0] == 1 && NFkB[0] == 0 && SNAI1[0] == 1 && SNAI2[0] == 0 && SOX2[0] == 0 || EZH2[0] == 0 && BMI1[0] == 1 && E2F[0] == 1 && Bcatenin[0] == 0 && YAP1[0] == 0 && HNF4A[0] == 1 && NFkB[0] == 0 && SNAI1[0] == 1 && SNAI2[0] == 0 && SOX2[0] == 1 && p53[0] == 1 || EZH2[0] == 0 && BMI1[0] == 1 && E2F[0] == 1 && Bcatenin[0] == 0 && YAP1[0] == 0 && HNF4A[0] == 1 && NFkB[0] == 1 && SNAI2[0] == 0 && p53[0] == 1 || EZH2[0] == 0 && BMI1[0] == 1 && E2F[0] == 1 && Bcatenin[0] == 0 && YAP1[0] == 1 && HNF4A[0] == 0 && SNAI2[0] == 0 && p53[0] == 1 || EZH2[0] == 0 && BMI1[0] == 1 && E2F[0] == 1 && Bcatenin[0] == 0 && YAP1[0] == 1 && HNF4A[0] == 1 && NFkB[0] == 0 && SNAI1[0] == 0 && ZEB1[0] == 0 && SNAI2[0] == 0 && TGFB[0] == 0 && p53[0] == 1 || EZH2[0] == 0 && BMI1[0] == 1 && E2F[0] == 1 && Bcatenin[0] == 0 && YAP1[0] == 1 && HNF4A[0] == 1 && NFkB[0] == 0 && SNAI1[0] == 0 && ZEB1[0] == 0 && SNAI2[0] == 0 && TGFB[0] == 1 || EZH2[0] == 0 && BMI1[0] == 1 && E2F[0] == 1 && Bcatenin[0] == 0 && YAP1[0] == 1 && HNF4A[0] == 1 && NFkB[0] == 0 && SNAI1[0] == 0 && ZEB1[0] == 1 && SNAI2[0] == 0 && p53[0] == 1 || EZH2[0] == 0 && BMI1[0] == 1 && E2F[0] == 1 && Bcatenin[0] == 0 && YAP1[0] == 1 && HNF4A[0] == 1 && NFkB[0] == 0 && SNAI1[0] == 1 && SNAI2[0] == 0 && p53[0] == 1 || EZH2[0] == 0 && BMI1[0] == 1 && E2F[0] == 1 && Bcatenin[0] == 0 && YAP1[0] == 1 && HNF4A[0] == 1 && NFkB[0] == 1 && SNAI2[0] == 0 && p53[0] == 1 || EZH2[0] == 0 && BMI1[0] == 1 && E2F[0] == 1 && Bcatenin[0] == 1 && HNF4A[0] == 0 && SNAI2[0] == 0 && p53[0] == 1 || EZH2[0] == 0 && BMI1[0] == 1 && E2F[0] == 1 && Bcatenin[0] == 1 && HNF4A[0] == 1 && NFkB[0] == 0 && SNAI1[0] == 0 && ZEB1[0] == 0 && SNAI2[0] == 0 && TGFB[0] == 0 && p53[0] == 1 || EZH2[0] == 0 && BMI1[0] == 1 && E2F[0] == 1 && Bcatenin[0] == 1 && HNF4A[0] == 1 && NFkB[0] == 0 && SNAI1[0] == 0 && ZEB1[0] == 0 && SNAI2[0] == 0 && TGFB[0] == 1 || EZH2[0] == 0 && BMI1[0] == 1 && E2F[0] == 1 && Bcatenin[0] == 1 && HNF4A[0] == 1 && NFkB[0] == 0 && SNAI1[0] == 0 && ZEB1[0] == 1 && SNAI2[0] == 0 && p53[0] == 1 || EZH2[0] == 0 && BMI1[0] == 1 && E2F[0] == 1 && Bcatenin[0] == 1 && HNF4A[0] == 1 && NFkB[0] == 0 && SNAI1[0] == 1 && SNAI2[0] == 0 && p53[0] == 1 || EZH2[0] == 0 && BMI1[0] == 1 && E2F[0] == 1 && Bcatenin[0] == 1 && HNF4A[0] == 1 && NFkB[0] == 1 && SNAI2[0] == 0 && p53[0] == 1) { p16[0] = 1; }

else { p16[0] = 0; }

//Logic rule for p53
[truncated: 333,759 more chars]
