# Supplementary material for: Analytical approach of synchronous and asynchronous update schemes applied to solving biological Boolean networks
Source: PLoS One. 2025 Sep 12;20(9):e0319240. doi: 10.1371/journal.pone.0319240 (PMC12431216; doi:10.1371/journal.pone.0319240)
Supplement: Attachment — Submitted filename: Response to Reviewers.docx [file pone.0319240.s002.docx]

Dear editor,

Attached to this rebuttal letter is the reviewed version of the manuscript titled “*Analytical approach of synchronous and asynchronous update schemes applied to solving biological Boolean networks*” in order to be reconsidered for its publication at *PLOS ONE*. This version of the manuscript implements all necessary changes to address the concerns expressed by the reviewers, which will be answered point-by-point below and highlighted in color blue.

**Reply to reviewer 1:**

Reviewer #1: This manuscript builds on prior work by Wang et al and by the authors to obtain the “canonical form” of a Boolean network by translating the landscape of attractors into the Cartesian plane. Unfortunately, the manuscript is not clear and understandable to the average reader. (This reviewer spent several hours trying to understand the manuscript, with limited success.) Partly due to the lack of clarity, many of the claims made in the abstract are unsupported. Most importantly, the single paragraph dedicated to solving real networks of biological systems does not describe which of the methods were actually applied, and how.

Major concerns
1. The manuscript needs a section with definitions, explanation of notations, and background information. For example, the representation of Boolean functions used in the manuscript needs to be explained. Technical terms such as “relation”, “reflexive element” need to be defined. In the statement of Theorem 3 it is not clear what is meant by c1Rc2 ^ c2Rc3 ^c3Rc1. This makes the theorem unintelligible. Moreover, the authors should not assume that every reader knows what f o f means.

Dear reviewer, please excuse the inconvenience in reading the manuscript. In attention to your comments, we improve the writing of our work by including a glossary to explain technical terms such as the concept of "relation" and the property of "reflexivity".

Briefly, the term "relation" is used to describe the mathematical association of two sets (see Figure A). On the other hand, a "function" is a special relation of sets, where for each element in the domain, there is a unique element in the codomain (see Figure B) (ISBN: 9780077418939). The concept of "reflexivity" is a property of relations, where there is at least one element related to itself (see Figure C) (ISBN: 9780077418939). Therefore, a "reflexive element" is an element related to itself (see Figure C). The condition $c_{1}Rc_{2}\wedge c_{2}Rc_{3}\wedge c_{3}Rc_{1}$ means that, in the relation that describes the periodic orbit of a cyclic attractor, the configuration c1 is related to c2; the configuration c2 is related to c3, and the configuration c3 is related to c1. The term $\left( f\circ f \right)$ is used to indicate the composition of a function $f$ with itself. Taking into account your comments, we have clarified all these aspects in the main text.

2. The point that the synchronous update scheme of a Boolean network is a particular case of Markov chains has been known for a while. For example, Ilya Shmulevich’s book on Probabilistic Boolean Networks uses Markov chains extensively.

Certainly, the connection between Markov chains and Boolean networks has been known for many years. In fact, Dr. Álvarez-Buylla, co-author of this study, was a pioneer in this field (PMID: 18978941). Regarding Dr. Shmulevich's work, his approach obtains the stable gene expression patterns (i.e., attractors) directly from the cells of interest by massive sequencing techniques, and then it uses Boolean networks to calculate transition probabilities to form Markov chains (PMID: 11847074; PMID: 36093045). This approach, although fascinating, is not what we present in this work.

Here, we found that the synchronous update scheme itself is a particular case of Markov chains. This result implies that the synchronous update scheme can be solved algebraically at a low cost compared to its traditional implementation. We admit that this idea is not entirely clear in the manuscript and, in response to your comment, we have fixed this issue in the main text.

3. The application of the theories is very limited. Instead of implementing the involved methodologies of obtaining the canonical form of the Boolean system, from the single paragraph dedicated to the application it seems that the only thing done was to determine the fixed point attractors of the systems. Determining fixed point attractors can be done by multiple means. Thus, the practical utility of the work presented in the manuscript is not clear.

In attention to your comments, we have expanded the applications section to showcase the potential of the approaches proposed in this paper.

4. The Supporting Information was not available for review.

We apologize for the inconvenience, but we sent the supplementary information through the journal's platform. We will contact the editors to verify that you can access it.

5. The paragraph of the Discussion dedicated to asynchronous update needs extensive revision. It is not factually correct that the dynamic features of asynchronous update are unexplained. The study of asynchronous Boolean systems is at least 60 years old, René Thomas being a very active pioneer. The degree of preservation of cyclic attractors of synchronous update has been well studied in the literature. Asynchronous update in which only one node may change at a time can also lead to a cyclic attractor. A necessary condition for such an attractor is the existence of a negative feedback loop in the interaction network (as conjectured by Rene Thomas and proven later). Cyclic attractor that rely on a positive feedback loop and node synchrony are not preserved. Various flavors of asynchronous and noisy update are actively researched, to establish the time implementation most suited for biological systems. There is a significant effort to find the update-independent features of Boolean systems, for example, their trap spaces.

We appreciate this observation, as learning about Dr. Thomas's scientific work was very enriching. In fact, after a thorough review, we discovered that our work extends the analytical line of research proposed by Dr. Thomas PMID: 4588055; PMID: 12779451; doi.org/10.1016/S0022-5193(05)80350-9). In this sense, our approach redefines his proposed asynchronous scheme as a composition of functions originating from the update order of the nodes. In this way, it is easy to track the effect of changing the update order, showing exactly how changes in the size of the basins of attraction occur and how the extinction of cyclic attractors occurs, observations widely reported for years (PMID: 30575543). Consequently, we mention in the main text how our work extends the analytical approach previously developed by Dr. Thomas.

More minor and specific points

6. The goal of finding the minimal necessary and sufficient components to generate a system’s dynamics is stated as a motivation for determining the canonical form. Yet, the manuscript does not establish how the canonical form may lead to the information on the necessary/sufficient components.

In mathematics, "canonical form" is by definition the standard and simplest way of representing a mathematical object (such as an equation, a matrix, or a polynomial). It facilitates their identification, comparison, and the study of their properties (PMID: 16577548). In the context of a Boolean network, the canonical form corresponds to the set of genes that determine the overall behavior of the network (doi.org/10.1080/10236198.2023.2220811). Consequently, the set of nodes in the canonical form of a Boolean network is absolutely essential to maintaining its attractor landscape.

7. “Topology of a network” is often used to refer to the graph structure of the network (the distribution of nodes and edges). “Topology of a Boolean network” can be misinterpreted to refer to the structure of the interaction network that underlies the Boolean system. The authors seem to mean the dynamics of the Boolean network, or its attractor landscape. They should state it accordingly.

In attention to your comment, we have made changes to the main text to avoid confusion with this terminology.

8. The intuition behind Lemma 1 and the function H needs to be presented in words. In particular, the reader may miss that u refers to the basin of attraction of attractor p. Example 1 needs more explanation, especially figure 1B, which illustrates the foundational concept, the function that corresponds to the landscape of attractors. The landscape of attractors is defined in line 73 as the set of attractors. It is not clear that the function also refers to the attractor basins. No description is given on how the disjunctive normal form is obtained, what its purpose is.

In attention to your observations, we have improved the presentation of Example 1 and also put into words the context of Lemma 1.

9. In line 199, what does S(u0, t=0) mean? Why does it equal u0 S(u0, t=1)? None of the equalities stated on that line make sense without definitions and explanations.

The idea behind this notation is to highlight that, starting from a fixed initial condition ($u_{0}$), at each time step, the system uses the logical rules of the network ($F$) to produce a new state. For example, at time $t = 0$, the synchronous update ($S$) is exactly the same as the initial starting configuration, i.e.: $S(u_{0}, t = 0) = u_{0}$. At the next time step ($t = 1$), then $S(u_{0}, t = 1) = F(u_{0})$. Moving to the next time step, we will say that $S(u_{0}, t = 2) = F(F(u_{0}))$, that is, the composition of $F$ with itself, i.e., $S(u_{0}, t = 2) = \left( F\circ F \right)(u_{0})$. Taking into account your observation and to improve the presentation of this idea, we explain it in detail in the main manuscript.

**Reply to reviewer 2:**

Reviewer #2: The paper addresses an important issue in modeling, especially biochemical systems - the reachability analysis of possible system states, which is still a challenging problem. The authors consider asynchronous and synchronous simulation for Boolean networks. The paper presents synchronous update schemes to characterize the topology of Boolean networks. The authors describe several theorems, which they prove and illustrate using small examples. They show a new matrix-based method to solve synchronously updated Boolean networks and derive interesting properties for Boolean networks. The authors show that the landscape of attractors of any Boolean network can be visualized using integers in the Cartesian plane, which allows one to easily obtain the canonical form of Boolean networks. They found an unexpected connection between the landscape of attractors and Markov chains. Moreover, the authors provide a simplified method for computing the canonical form of synchronously updated networks. They consider a matrix formalism of an asynchronous update scheme.
The paper is well-written and clearly explained.
I find the approach interesting, but I see some weaknesses, so I recommend a major revision.

(1) Please provide the biological models in the SBML format for reuse by the community and by other software tools

Dear reviewer, we thank you for your insightful comments and valuable observations. In attention to this request, we have converted the genetic network models to SBML and made them available in the supplementary material.

(2) Refer to related work, such as other tools, e.g., GINSIM.

In attention to your request, we worked with GINSIM and BoolNet to determine the scope of our approach. We present these results in the New Applications section of the main text.

(3) How does the approach relate to minimal transition invariants in Petri nets? Minimal transition invariants in Petri nets characterize the minimum number of nodes necessary for the functioning of the network, which seems to be similar to the concept of the canonical form of Boolean networks that should be precisely the set of minimal, necessary, and sufficient elements to generate the entire dynamics of any network.

We thank you for this question, since Petri nets are not part of our specialty and it was very interesting to learn about the topic. The definition of “canonical form” we present here, refers to a network with the minimum components and interactions necessary to obtain a given attractor landscape. For example, consider the following network:

$$\left\{ \begin{aligned} x_{1}^{+}=x_{1}+\bar{x}_{1} \\ x_{2}^{+}=x_{1}+x_{2} \end{aligned} \right.$$

From the first step of synchronous update of this network, the following function is generated:

By composing the same function several times, we can observe that its image (in red) converges to a specific attractor landscape, that is:

Consequently, the trajectories of this example network have the following form:

However, in the end all trajectories reach this equilibrium configuration:

Which is also produced by this other network:

$$\left\{ \begin{aligned} x_{1}^{+}=1 \\ x_{2}^{+}=1 \end{aligned} \right.$$

And this is the definition of canonical form we present. On the other hand, invariant Petri net transitions are characterized by not generating changes in the token distribution. Formally:

*Let* $(N,m)$ *be a marked Petri net, and let* $\sigma\in T^{*}$ *be a firing sequence such that* $\left( N:m\underset{\to}{\sigma}m \right)$*. Then* $\vec{\sigma}$ *is a transition invariant.*

In answer to your question, we note that the canonical form of Boolean networks is equivalent to constructing a network such that its image (in red) converges directly to a set of invariant transitions (blue arrows). For example, we can transform this:

To this:

We think this result, although interesting, deserves a separate paper, as we need to study Petri nets in more depth; and for this reason, we decided not to mention it in the main text.

(4) The biological examples are rather small networks. Say something about the scalability of your approach.

We appreciate your comment, because it allowed us to delve deeper into the applications of our approach. In response to your request, our method for characterizing Boolean network dynamics cannot be directly applied to large networks, as it preserves the brute force problem of characterizing $2^{n}$ initial states of an n-node network. However, by connecting our work with the results of Dr. Madalena Chaves (PMID: 29896108), our approach is useful for quickly computing attractors for large networks.

To be precise, we found that by dividing the network into small modules, as proposed by Dr. Chaves, and applying the approach developed in this paper, we were able to compute the fixed-point attractors of very large networks. In the applications section, we present the implementation of this idea on a 200-node test network. In fact, for this network, the number of initial states to be compared with our approach was 10485760, while for the brute force method approximately 1.61E+60 (i.e., $2^{60}$) are required to explore.
