# Supplementary material for: Analytical approach of synchronous and asynchronous update schemes applied to solving biological Boolean networks
Source: PLoS One. 2025 Sep 12;20(9):e0319240. doi: 10.1371/journal.pone.0319240 (PMC12431216; doi:10.1371/journal.pone.0319240)
Supplement: Attachment — Submitted filename: Response_to_Reviewers_auresp_2.docx [file pone.0319240.s003.docx]

Dear editor,

Attached to this rebuttal letter is the reviewed version of the manuscript titled “*Analytical approach of synchronous and asynchronous update schemes applied to solving biological Boolean networks*” in order to be reconsidered for its publication at *PLOS ONE*. This version of the manuscript implements all necessary changes to address the final concern expressed by Reviewer 1, which will be answered below and highlighted in color blue.

**Reply to reviewer 1:**

Reviewer #1: The revised manuscript addresses my points.

There is a single remaining sentence, on line 385-386, that is unclear

"*Furthermore, it was surprising that only fixed-point attractors were found in the synchronous and asynchronous update schemes (27*)."

What subset of Boolean models is referred to here? It certainly cannot be all Boolean dynamical systems. Why was this surprising? Please rephrase to the sentence is clear and to the point.

Dear reviewer, in attention to your request, we rewrote the sentence to clarify that we were referring to Boolean networks.

Specifically, we replaced "*Furthermore, it was surprising that only fixed-point attractors were found in the synchronous and asynchronous update schemes*" with "*These studies also showed that fixed-point attractors are invariant to the update scheme used to analyze Boolean networks*".

These changes were implemented on lines 373-374.
